# Supplementary material for: Topical chlorhexidine 0.2% versus topical natamycin 5% for fungal keratitis in Nepal: rationale and design of a randomised controlled non-inferiority trial
Source: BMJ Open. 2020 Sep 30;10(9):e038066. doi: 10.1136/bmjopen-2020-038066 (PMC7528427; doi:10.1136/bmjopen-2020-038066)
Supplement: Supplementary data [file bmjopen-2020-038066supp001.pdf]

Appendix 1: Informed Consent Material

Study Title: Randomised controlled trial of topical chlorhexidine 0.2% versus topical natamycin 5% for fungal keratitis in Nepal

Participant Information Sheet and Consent Form - No 1:
For baseline assessment and investigations

Introduction
You are being invited to take part in a medical research study. Before you decide it is important for you to understand why the research is being done and what it will involve. Please take time to read or listen to the following information carefully and to talk to others about the study, if you wish. Ask us if there is anything that is not clear or if you would like more information. Do not sign the consent form unless you are satisfied with the answers to your questions and decide that you want to be part of this study. Take time to decide whether or not you wish to take part.

Why have I been invited?
You have been invited to take part because you have a corneal infection. To guide the treatment of this it is helpful to do tests to find out the cause, to guide the choice of eye drop treatment. We are asking for your permission to carry out some tests that are listed below to find out what is causing the infection and to decide if you may be suitable for a trial comparing treatments for fungal eye infections.

What is this study about?
Infection of the clear part of the front of the eye (the cornea) is a corneal ulcer. It is an important cause of blindness in Nepal. A scratch in the cornea allows infection to enter and an ulcer to begin. These infections can be very serious with some people losing the sight in the affected eye.

Different types of infectious organisms can cause corneal ulcers. These include bacteria and fungi which need to be treated with different types of eye drop medicines. To find out what type of infection is present it is necessary to collect a small sample from the ulcer and perform various laboratory tests. The test results guide the treatment. In Nepal about half of the infections are caused by fungus.

Fungal infections can be difficult to diagnose. No single test can reliably detect it. It may need several different tests to find it. Currently, in many countries where fungal ulcers are common laboratory services are limited or not available. Tests are expensive and may not detect all of the infections. There is a need to understand which of the current test options give the most useful information.

The first purpose of this study is to carefully evaluate your eye infection using several different diagnostic tests to try to find out what type of infection you have, and to learn which tests are most useful in this process.

There is a need for alternative, affordable and more easily available eye drop treatments for fungal infections. We are also conducting a clinical trial to compare two eye drop treatments for fungal infection, to try to find out which is better.

Therefore, the second purpose of this initial Assessment and Investigation study is to identify people who have a fungal corneal infection who may be eligible and willing to enrol in the clinical trial. If we find out that you do have a fungal corneal infection, we will then ask if you would be interested in hearing about the clinical trial. If you are interested, then at that stage we will explain about the treatment trial in detail, provide you with a second information sheet and go throw a second consent process.

Do I have to take part?
No. Your involvement is entirely voluntary. If you agree to take part, we will then ask you to sign a consent form. If you decide to join and change your mind, you are free to withdraw at any time without giving a reason. This will not affect the standard of care you receive.
Why have I been invited?
You have been invited to take part because you have a corneal infection. To guide the treatment of this it is helpful to do tests to find out the cause, to guide the choice of eye drop treatment. We are asking for your permission to carry out some tests that are listed below to find out what is causing the infection and to decide if you may be suitable for a trial comparing treatments for fungal eye infections.

What will happen to me if I take part?
If you agree to be part of this study, the following will happen:

- We will ask you a series of questions. This will include basic demographic information, the history of your current eye problem, and treatment you have had before arriving at the hospital.

- We will then carefully examine both eyes using a special microscope.

- Your eyes will be photographed with a camera. This is additional to standard care, and will help us to monitor the infection and the response to treatment.

- We will use a special microscope to look at the cornea to see if we can find a fungal infection. This involves putting anaesthetic drops on the eye. A soft plastic device then gently touches the eye so to see if you have a fungal infection. This is additional to standard care, and will help us to find out the type of infection you have more rapidly so that we can offer you the most appropriate treatment.

- We will collect samples form the corneal ulcer to test in the laboratory to try to identify what is causing the infection. Anaesthetic eye drops will be used numb the eye so you will not feel anything while we collect the sample by brushing the surface.
-
- We will check your blood sugar level for diabetes. This is done using a finger-prick blood sample. If this is raised, we will refer you to a separate group of doctors to help you with this.

- You will be offered a test for HIV infection. This will be done through the hospitals counselling and testing services. If you choose to accept this, then you will be separately counselled about the test and the implications of the results. This would involve the collection of a blood test sample from your arm. If the HIV test is positive, then you will be referred to the appropriate team for ongoing care. The result of this test will be shared with the us (cornea infection study team), with your consent, as it is potentially relevant to the treatment of your cornea infection.

- We will collect a sample of the cells from the inside of your cheek by gently rubbing a swab for a few seconds. This is additional to the standard of care. The purpose is to try to understand how the body fights the infection and why some people develop this eye problem and others do not.

- You will be asked to complete three short questionnaires to assess your quality of life and vision function. This is additional to standard care, and will help us to better understand the impact corneal ulcers can have on people's lives.

If we find evidence of fungus infecting your eye, then we will discuss the details of the trial with you further. At that point we will give you a second information sheet to read (or read to you), and if you meet all the eligibility criteria for the trial, to then go through a second consent process with you.

If you do not have a fungal infection, for example if the infection is caused by bacteria, then your will receive standard ongoing treatment for this by the same team at the hospital.

You may be withdrawn from the study without your consent if the researchers believe it is in your best interest or if you fail to follow study procedures.

What are the side effects or risks of taking part?
- The anaesthetic eye drops usually sting for a few seconds and then your eye should feel more comfortable. There is a very low risk of allergic reaction from the anaesthetic eye drops.
- The procedures including the history taking, examination, photography, in vivo confocal microscopy and sample collection are part of the standard of care for assessing a corneal infection. The risk associated with these are very low and no different from routine care. To minimise discomfort, topical anaesthetic will be given before examinations and sample collection.
- You will experience a little pain when a blood sample is collected. The people collecting the blood sample will ensure that this is done with as little discomfort as possible.
- The questions, tests and examinations will add about 15 minutes to your hospital visit above what we would usually expect in assessing a patient with this problem.

What are the benefits of taking part?
- The study will involve tests for the type of infection. This helps the doctor looking after you to choose the best type of treatment for your eyes
- The costs for the clinical assessment and the tests will be paid for by the study.
- By participating in this study, you will be helping to further research into this field so more informed decisions can be made when treating people with corneal infection.

What will happen to the clinical records, photographs and test results?
Your records will remain strictly confidential at all times. The information will be held in a secure office at your treating hospital. Only the people organizing or supervising the trial and regulatory authority auditors will have access to it. These include officials delegated by the Sponsor (London School of Hygiene and Tropical Medicine), the local National Ethics Committee, The local National Drug Regulatory Authority and trial Data Safety Monitoring Body (DSMB).

Your name will not be passed to anyone else outside the research team, unless we have your direct instruction to do so, for example to make a medical referral.

The photographs of the eye and the result of the laboratory test for infection will be shared with computer engineers to help develop a programme that can automatically analyse the image to see if this can provide some indication of the cause of the infection. Images of corneal infection may also be used for educational and teaching purposes, including in publications. All personal identifying information will be removed before sharing images.

What tests will we do on the sample?
The samples collected from the surface of your eye will be tested in several different ways to determine what is causing the infection. This work will be done in the hospital microbiology laboratory, where you are being treated. A portion of the infection sample will be transferred for additional special tests at KCMC Hospital Biotechnology (Tanzania), the London School of Hygiene and Tropical Medicine (UK) and Radboud University Nijmegen Medical Center (The Netherlands), as some of the tests will require additional special equipment that is not available at all sites.

- We will look for the type of infection using a microscope and by growing the organisms in the laboratory. We will test the organisms that grow to see which medicines work best to kill the infection, which is helpful in guiding the choice of treatment to be used.

- The swab samples from the ulcer will be used to test for infection using molecular diagnostic tests and to evaluate new tests that may be used to find the cause rapidly in the clinic.

- We will use study genetic material of the organism causing the infection to find out the exact type of infection and its ability to resist treatments. Samples from the ulcer may also be used to investigate your immune response to the infection, so that we can better understand what causes corneal scarring

- We will store a sample of the infection causing organism indefinitely for additional testing.

- The genetic material from the cells from your cheek will be sent to the UK or to KCMC Hospital in Tanzania. We would like to store it until a later time when a sufficiently large number of samples has been collected to conduct an analysis of this genetic material. The purpose would be to try to better understand how the human immune system fights the infection and why some people develop this eye problem and others do not. By doing these tests we hope that it will help us to develop approaches that will help to prevent or improve outcomes from this condition.

- As part of this consent we are asking that you give us permission to store this material to be able to test it at a later date as mentioned above. We do not know exactly for how long we shall store the genetic material before we have assembled a sufficiently large collection during the course of several planned studies to be able to proceed to the sample analysis, however, we anticipate a period of at least five years.

What will happen to the results of the research study?
The results of the study will be available after it finishes and will be included in peer reviewed medical and scientific journals and may be presented at medical meetings. Results will also be published on a publicly accessible trials database. The data will be anonymous and none of the patients involved in the trial will be identified in any report or publication. Should you wish to see the results, or the publication, please ask your study doctor.

Who is funding the research?
The research is being funded as part of a grant from the Wellcome Trust, UK.

Who is organising the research?
It is being organised through a research partnership between the London School of Hygiene and Tropical Medicine and Sagarmatha Choudhary Eye Hospital in Nepal, Mbarara University of Science and Technology in Uganda and Kilimanjaro Christian Medical Centre in Tanzania.

What if relevant new information becomes available?
It is not anticipated that new information will become available during the course of this short study. The information from the microbiology tests will be available to the doctors to help select the most appropriate treatment for you.

What if something goes wrong?
If you have a concern about any aspect of this study, you should ask to speak to the researchers who will do their best to answer your questions. If you remain unhappy and wish to complain formally, you can do this through the head of the hospital eye department or the named person on the following page. The London School of Hygiene and Tropical Medicine holds insurance policies which apply to this study. If you experience harm or injury as a result of taking part in this study, you may be eligible to claim compensation.

Who has reviewed the study?
Prospective research such as this is looked at by an independent group of people, called a Research Ethics Committee, to protect your interests. This study has been reviewed and approved by (1) the London School of Hygiene and Tropical Medicine Research Ethics Committee; (2) Nepal Health Research Council.

What will happen if I don't want to carry on with the study?
Your participation in this study is entirely voluntary. You may refuse to participate or may withdraw from this study at any time without penalty or loss of any rights or benefits to which you are otherwise entitled. The study doctor may also stop your participation in the study at any time for safety reasons. If you decide to withdraw from the study you should contact a member of the study team immediately. You do not have to give a reason when stopping, however for safety reasons, it is suggested that you tell the study doctor if you decide to stop because of an unwanted side effect. If you withdraw from the study, we will only use data collected before this decision, unless you request this to also be withdrawn. If you withdraw from the study, researchers, authorized persons from the Sponsor and the regulatory authorities will still require access to your medical notes to verify the data collected up to the date of your withdrawal.

Contact Details
Nepal Study Site: Dr Reena Yadav, Sagarmatha Choudhary Eye Hospital, Lahan. T: +977 9807799958

Study Coordinator: Dr Jeremy Hoffman: email: Jeremy.hoffman@lshtm.ac.uk
Chief Investigator: Prof. Matthew Burton: email: matthew.burton@lshtm.ac.uk

You will be given a copy of the information sheet and a signed consent form to keep.
Thank you for considering taking the time to read this sheet.

Randomised controlled trial of topical chlorhexidine 0.2% versus topical natamycin 5% for fungal keratitis

Consent Form No 1: Baseline assessment and investigations

Participant Name___________________________ **Study ID Number:** ____________________

	**Please initial box**
1. I confirm that I have read and understand the participant information sheet dated .... (version ....) for the above study. I have had the opportunity to consider the information, ask questions and have had these answered fully.	
2. I understand that my participation is voluntary and I am free to withdraw at any time, without giving any reason, without my medical care or legal rights being affected.	
3. I understand that sections of my medical notes and data collected during the study may be looked at by responsible individuals from the London School of Hygiene & Tropical Medicine, from national regulatory authorities or from this hospital, where it is relevant to my taking part in this research. I give permission for these individuals to access my records.	
4. I agree to take part in the above study.	
5. I agree to the collection, laboratory tests and storage for future analysis of the samples from the surface my eye infection to understand the disease as described above.	
6. I agree to the collection and storage for future analysis of the samples from the inside of my mouth to investigate how human genes affect this disease.	
7. I agree to having a blood sample collected to measure sugar levels	
8. I agree to being referred for a HIV pre-test counselling, testing and for the results to be shared with the research team.	
9. I agree for the photographs of the front of my eye to be used in the publication or report released on the study, and for teaching purposes, including on the internet.	
10. I understand that data about/from me/the participant may be shared via a public data repository or by sharing directly with other researchers, and that I will not be identifiable from this information.	

_________________________ _________________________ _________________________

Name of Participant *(printed)* Signature/Thumbprint Date

_________________________ _________________________ _________________________

Name of Person taking consent Signature Date

The participant is unable to sign. As a witness, I confirm that all the information about the study was given and the participant consented to taking part.

_________________________ _________________________ _________________________

Name of Impartial Witness *(if required)* Signature Date

1 copy for participant; 1 copy for Principal Investigator; 1 copy to be kept with hospital notes

Study Title: Randomised controlled trial of topical chlorhexidine 0.2% versus topical natamycin 5% for fungal keratitis in Nepal

Participant Information Sheet and Consent Form – No 2:
For enrolment into the clinical trial

Introduction
Thank you for earlier participating in the first stage of the study. You are now being invited to take part in the clinical treatment trial part of this medical research study. Before you decide it is important for you to understand why the research is being done and what it will involve. Please take time to read or listen to the following information carefully and to talk to others about the study, if you wish. Ask us if there is anything that is not clear or if you would like more information. Do not sign the consent form unless you are satisfied with the answers to your questions and decide that you want to be part of this study. Take time to decide whether or not you wish to take part.

Why have I been invited?
You have been invited to take part because you have a corneal infection which we have found out from tests is caused by fungus.

What is this study about?
Infection of the clear part of the front of the eye (the cornea) is a corneal ulcer. It is an important cause of blindness in Nepal. A scratch in the cornea allows infection to enter and an ulcer to begin. These infections can be very serious with some people losing the sight in the affected eye.

Different types of infectious organisms can cause corneal ulcers. These include bacteria and fungi. In tropical regions about half of all corneal ulcers are caused by fungi. Bacteria and fungi need to be treated with different types of eye drop medicines.

Treatments for fungal eye infections are frequently not very effective, in addition access to these treatments in many countries is very limited and can be expensive. In some countries they are simply not available. Currently the most commonly used treatment for fungal corneal ulcers is an eye drop called Natamycin. There is a need for additional, alternative, affordable and more easily available eye drop treatments for fungal infections.

There is an antiseptic solution called Chlorhexidine. This is very effective at killing bacteria, fungi and other types of infectious organisms. It is used in medical care worldwide in several different ways. For example, it is used to clean skin before surgical operations, in antiseptic creams for skin cuts and as a mouth wash to prevent and treat mouth infections. It has been used in eye care for more than thirty years as an eye-drop preservative, for sterilizing contact lenses, for pre-operative topical antiseptic and for treating *Acanthamoeba* and fungal corneal infections.

About twenty years ago chlorhexidine eye drops were tested in two small clinical trials conducted in India and Bangladesh for the treatment of fungal corneal infections. The results of these studies suggested that chlorhexidine was as good as and possibly better than natamycin at controlling the infection. Neither eye drop had any serious side effect. However, the studies were not large enough to be certain.

Chlorhexidine is currently used in several countries for the treatment of fungal corneal infections when natamycin or alternative treatment is not working or is not available. We would like to conduct a large clinical to find out whether chlorhexidine 0.2% eye drops are as effective as or more effective than natamycin 5% eye drops for treating fungal corneal infections

Do I have to take part?
No. Your involvement is entirely voluntary. If you agree to take part, we will then ask you to sign a consent form. If you decide to join and change your mind, you are free to withdraw at any time without giving a reason. This will not affect the standard of care you receive. If you decide not to participate in the study, then you will be offered the standard treatment for fungal keratitis using natamycin.

What will happen to me if I take part?
If you agree to be part of this study, the following will happen:

1) Baseline Assessment:
As part of the initial assessment that has already taken place we have carefully examined yours eyes, taken photographs, performed a scan for infection and collected samples. The tests have found that you have a fungal infection in your cornea. The risks to an unborn or breast-fed baby from antifungal eye drops use are unknown. Therefore, pregnant and breastfeeding women are excluded from participating in this study. Pregnancy testing will be offered to potential female participants to confirm pregnancy status.

2) Randomisation:
We will randomly allocate you to one of the two treatment option: either chlorhexidine eye drops or natamycin eye drops. Sometimes we don't know which way of treating patients is best. To find out, we need to make comparisons between the different treatments. We put people into groups and give each group one of the two alternative treatments; the results are compared after some time to see if one is better. To try to make sure the groups are the same to start with, each patient is put into a group by chance (randomly). You have an equal chance of being put into the chlorhexidine or natamycin treatment group. Neither you nor the people examining your eyes will be told which treatment group you are in. It is important that neither you nor we know which of the two you are given. This information would be in our files, but we would not look at these files until after the research is finished. This is the best way we have for testing without being influenced by what we think might happen. We would then compare which of the two treatments has the best results.

3) Treatment:
Once you are allocated to one of the treatment groups, you will receive clear instruction on how to take the eye drops. For the first week we will ask you to take one drop every hour. For the second and the third weeks the frequency of the eye drops will be reduced to every other hour (2-hourly). After that the frequency and duration of treatment will depend on the severity of the infection and how it is responding. You will be given clear guidance on this by the eye doctor who is looking after you.

4) Follow-up Assessment:
Initially, most people with corneal infections stay in hospital for several days so that the clinical team can monitor the response of the infection to the treatment. For the purposes of the study, we would like to review the response to treatment and document the clinical findings at the following times after you start treatment: two days, 1 week, 2 weeks, 3 weeks, 2 months and 3 months.

On each occasion we will ask you a few questions about your eye and the treatment. We will measure your eye sight. We will examine the eye with a microscope and photograph it with a camera.

At 1 week, 2 weeks and 3 weeks we will repeat the *in vivo* confocal microscopy test that was done at your first assessment. This is done to see how the infection is responding to treatment. This involves putting anaesthetic drops on the eye so that you do not feel any discomfort. A soft plastic device then gently touches the surface of the eye so that we can take special photographs of the front of your eye ("scan")

At 1 week if you still have an open ulcer on the cornea we will repeat the sample collection to test for the ongoing presence of the infection. This involves first putting anaesthetic eye drops on the surface of the eye. Then gently scraping the surface of the corneal ulcer and testing for the presence of fungus and bacteria in the microbiology laboratory.

Sometimes fungal infections do not respond to the treatment. In such cases it may be necessary to alter the treatment or perform an operation. The eye doctors who will be looking after you will monitor your progress closely and advise you about further treatment might be needed.

At the three month review you will be asked to complete three short questionnaires to assess your quality of life and vision function. These are the same standardised questions used in the baseline assessment to try to understand the impact that different medical problems can have on people's lives.

You may be withdrawn from the study without your consent if the researchers believe it is in your best interest or if you fail to follow study procedures.

What are the side effects or risks of taking part?

1. **Random Allocation and Treatment Failure**: You will be randomly allocated to a treatment. The treatment you are allocated to may prove to be less effective or to have more side effects than the other study treatment or other available treatments.

   It is important to recognise that corneal infection is a serious, sight threatening condition. Many patients, whatever the treatment used, have reduced vision in the affected eye after it has resolved. In some people the affected eye will become blind. Sometimes the infection, despite lots of treatment, can progress to cause a hole to develop in the cornea (Perforation) and sometimes it is so severe it is necessary to perform an operation to remove the eye content.

2. **Local Irritation:** As with most eye drops, there is the risk of local irritation or stinging from either chlorhexidine or natamycin. This usually only lasts for a short time.

3. **Allergic Response**: Very rarely, either chlorhexidine or natamycin eye drops can provoke a local allergic reaction on the surface of the eye or the eyelids.

4. **Pregnancy and Breast Feeding:** The risks to an unborn or breast-fed baby from antifungal eye drops use are unknown. Therefore, pregnant and breastfeeding women are excluded from participating in this study.

5. **Natamycin 5% eye drops**: Natamycin is an approved antifungal medication that is currently being used for the treatment of fungal corneal ulcer. It is on the World Health Organisation Essential Medicines List for the treatment of fungal corneal infections. There are no known serious side effects with this medication. It may cause mild irritation and very rarely a local allergic response.

6. **Chlorhexidine 0.2% eye drops:** Chlorhexidine eye drops are used on the surface of the eye as an antiseptic before procedures and also in the treatment of fungal and other eye infections. It has not been associated we any serious side effects. It may cause mild irritation and very rarely a local allergic response. This concentration of chlorhexidine is approved to be used in much larger volumes as a mouth wash. It is considered to be safe and is not associated with any systemic side effects.

7. **Procedures:** including examinations, confocal microscopy, corneal sample collection and checking for the best glasses or contact lenses carry the same very small risk whether they are performed as part of this study or of usual care outside the study. To minimise discomfort, topical anaesthetic will be given before examinations and sample collection.

8. **Unknown Risks:** The treatments in this study may have rare side effects that are currently not known. If during the course of the study new information becomes available, the researchers will share this with.

What are the possible benefits of taking part?

- The study will involve tests for the type of infection. This helps the doctor looking after you to choose the best type of treatment for your eyes
- The costs for your clinical assessment, tests, treatment and transport will be paid for by the study.
- By participating in this study, you will be helping to answer the question about whether or not chlorhexidine is a suitable alternative treatment for fungal corneal infections.

What will happen to the clinical records, photographs and test results?
Your records will remain strictly confidential at all times. The information will be held in a secure office at your treating hospital. Only the people organizing or supervising the trial and regulatory authority auditors will have access to it. These include officials delegated by the Sponsor (London School of Hygiene and Tropical Medicine), the local National Ethics Committee, The local National Drug Regulatory Authority and trial Data Safety Monitoring Body (DSMB).

A study number rather than your name will be used on study records or the database wherever possible. Your name and other facts that might identify you will not appear when we present this study or publish its results.

Your name will not be passed to anyone else outside the research team, unless we have your direct instruction to do so, for example to make a medical referral.

Images of corneal infection may be used for educational and teaching purposes, including in publications. All personal identifying information will be removed before sharing images.

What tests will we do on the sample?
The samples collected from the surface of your eye will be tested in several different ways to determine what is causing the infection. This work will be done in the hospital microbiology laboratory, where you are being treated. A portion of the infection sample will be transferred for additional special tests at KCMC Hospital Biotechnology (Tanzania), the London School of Hygiene and Tropical Medicine (UK) and Radboud University Nijmegen Medical Center (The Netherlands).

We will look for the type of infection using a microscope and by growing the organisms in the laboratory. We will test the organisms that grow to see which medicines work best to kill the infection, which is helpful in guiding the choice of treatment to be used. The swab samples from the ulcer will be used to test for infection using molecular diagnostic tests and to evaluate new tests that may be used to find the cause rapidly in the clinic. We will use the genetic material of the organism causing the infection to sequence its genetic code, which helps us to find out the exact type of infection and its ability to resist treatments. We will store a sample of the infection causing organism indefinitely for additional testing.

What will happen to the results of the research study?
The results of the study will be available after it finishes and will be included in peer reviewed medical and scientific journals and may be presented at medical meetings. Results will also be published on a publicly accessible trials database. The data will be anonymous and none of the patients involved in the trial will be identified in any report or publication. Should you wish to see the results, or the publication, please ask your study doctor.

Who is funding the research?

The research is being funded as part of a grant from the Wellcome Trust, UK.

Who is organising the research?
It is being organised through a research partnership between the London School of Hygiene and Tropical Medicine and Sagarmatha Choudhary Eye Hospital in Nepal, Mbarara University of Science and Technology in Uganda and Kilimanjaro Christian Medical Centre in Tanzania.

What if relevant new information becomes available?
It is not anticipated that new information will become available during the course of this study. However, if it does, this will be shared with you by the researchers in case this affects whether you wish to continue in the study.

What if something goes wrong?
If you have a concern about any aspect of this study, you should ask to speak to the researchers who will do their best to answer your questions. If you remain unhappy and wish to complain formally, you can do this through the head of the hospital eye department or the named person on the following page. The London School of Hygiene and Tropical Medicine holds insurance policies which apply to this study. If you experience harm or injury as a result of taking part in this study, you may be eligible to claim compensation.

Who has reviewed the study?
Prospective research such as this is looked at by an independent group of people, called a Research Ethics Committee, to protect your interests. This study has been reviewed and approved by (1) the London School of Hygiene and Tropical Medicine Research Ethics Committee; (2) Nepal Health Research Council.

What will happen if I don’t want to carry on with the study?
Your participation in this study is entirely voluntary. You may refuse to participate or may withdraw from this study at any time without penalty or loss of any rights or benefits to which you are otherwise entitled. The study doctor may also stop your participation in the study at any time for safety reasons. If you decide to withdraw from the study you should contact a member of the study team immediately. You do not have to give a reason when stopping, however for safety reasons, it is suggested that you tell the study doctor if you decide to stop because of an unwanted side effect. If you withdraw from the study, we will only use data collected before this decision, unless you request this to also be withdrawn. If you withdraw from the study, researchers, authorized persons from the Sponsor and the regulatory authorities will still require access to your medical notes to verify the data collected up to the date of your withdrawal.

Contact Details
Nepal Study Site: Dr Reena Yadav, Sagarmatha Choudhary Eye Hospital, Lahan. T: +977 9807799958

Study Coordinator: Dr Jeremy Hoffman: email: Jeremy.hoffman@lshtm.ac.uk
Chief Investigator: Prof. Matthew Burton: email: matthew.burton@lshtm.ac.uk

You will be given a copy of the information sheet and a signed consent form to keep.
Thank you for considering taking the time to read this sheet.

Randomised controlled trial of topical chlorhexidine 0.2% versus topical natamycin 5% for fungal keratitis

Consent Form No 2: Enrolment into the clinical trial

Participant Name____________________________ **Study ID Number:** ____________________

	**Please initial box**
1. I confirm that I have read and understand the participant information sheet dated .... (version ....) for the above study. I have had the opportunity to consider the information, ask questions and have had these answered fully.	
2. I understand that my participation is voluntary and I am free to withdraw at any time, without giving any reason, without my medical care or legal rights being affected.	
3. I understand that sections of my medical notes and data collected during the study may be looked at by responsible individuals from the London School of Hygiene & Tropical Medicine, from national regulatory authorities or from this hospital, where it is relevant to my taking part in this research. I give permission for these individuals to access my records.	
4. I agree to take part in this clinical treatment trial.	
5. I agree to the collection, laboratory tests and storage for future analysis of the samples from the surface my eye infection to understand the disease as described above.	
6. I agree for the photographs of the front of my eye to be used in the publication or report released on the study, and for teaching purposes, including on the internet.	
7. I understand that data about/from me/the participant may be shared via a public data repository or by sharing directly with other researchers, and that I will not be identifiable from this information.	

____________________ ____________________ ________________

Name of Participant *(printed)* Signature/Thumbprint Date

____________________ ____________________ ________________

Name of Person taking consent Signature Date

The participant is unable to sign. As a witness, I confirm that all the information about the study was given and the participant consented to taking part.

____________________ ____________________ ________________

Name of Impartial Witness *(if required)* Signature Date

1 copy for participant; 1 copy for Principal Investigator; 1 copy to be kept with hospital notes
