# Supplementary material for: Topical chlorhexidine 0.2% versus topical natamycin 5% for fungal keratitis in Nepal: rationale and design of a randomised controlled non-inferiority trial
Source: BMJ Open. 2020 Sep 30;10(9):e038066. doi: 10.1136/bmjopen-2020-038066 (PMC7528427; doi:10.1136/bmjopen-2020-038066)
Supplement: Supplementary data [file bmjopen-2020-038066supp002.pdf]

Appendix 2: Monitoring for Harm

Definitions

Adverse Event (AE): any untoward medical occurrence in a patient or clinical trial subject administered a medicinal product and which does not necessarily have a causal relationship with this treatment. *An AE can therefore be any unfavourable and unintended sign (including an abnormal laboratory finding), symptom, or disease temporally associated with the use of an investigational medicinal product (IMP), whether or not considered related to the IMP.*

Adverse Reaction (AR): all untoward and unintended responses to an IMP related to any dose administered. *All AEs judged by either the reporting investigator or the sponsor as having reasonable causal relationship to a medicinal product qualify as adverse reactions. The expression reasonable causal relationship means to convey in general that there is evidence or argument to suggest a causal relationship.*

Unexpected Adverse Reaction: an AR, the nature or severity of which is not consistent with the applicable product information (e.g. investigator's brochure for an unapproved investigational product or summary of product characteristics (SmPC) for an authorised product*). When the outcome of the adverse reaction is not consistent with the applicable product information this adverse reaction should be considered as unexpected. Side effects documented in the SmPC which occur in a more severe form than anticipated are also considered to be unexpected.*

Serious Adverse Event (SAE) or **Serious Adverse Reaction:** any untoward medical occurrence or effect that at any dose

- **Results in death**
- **Is life-threatening** – *refers to an event in which the subject was at risk of death at the time of the event; it does not refer to an event which hypothetically might have caused death if it were more severe*
- **Requires hospitalisation, or prolongation of existing inpatients' hospitalisation**
- **Results in persistent or significant disability or incapacity**
- **Is a congenital anomaly or birth defect**

Medical judgement should be exercised in deciding whether an AE/AR is serious in other situations. Important AE/ARs that are not immediately life-threatening or do not result in death or hospitalisation but may jeopardise the subject or may require intervention to prevent one of the other outcomes listed in the definition above, should also be considered serious.

Suspected Unexpected Serious Adverse Reaction (SUSAR): any suspected adverse reaction related to an IMP that is both unexpected and serious.

Causality

Most adverse events and adverse drug reactions that occur in this study, whether they are serious or not, will be expected treatment-related toxicities due to the drugs used in this study. The assignment of the causality should be made by the investigator responsible for the care of the participant using the definitions in the table below.

Relationship	Description
**Unrelated**	There is no evidence of any causal relationship
**Unlikely**	There is little evidence to suggest there is a causal relationship (e.g. the event did not occur within a reasonable time after administration of the trial medication). There is another reasonable explanation for the event (e.g. the participant's clinical condition, other concomitant treatment).
**Possible**	There is some evidence to suggest a causal relationship (e.g. because the event occurs within a reasonable time after administration of the trial medication). However, the influence of other factors may have contributed to the event (e.g. the participant's clinical condition, other concomitant treatments).
**Probable**	There is evidence to suggest a causal relationship and the influence of other factors is unlikely.

**Definitely**	There is clear evidence to suggest a causal relationship and other possible contributing factors can be ruled out.
**Not assessable**	There is insufficient or incomplete evidence to make a clinical judgement of the causal relationship.

If any doubt about the causality exists the local investigator should inform the study coordination centre who will notify the Chief Investigators. The pharmaceutical companies and/or other clinicians may be asked to advise in some cases.

In the case of discrepant views on causality between the investigator and others, all parties will discuss the case. In the event that no agreement is made, the MHRA will be informed of both points of view.

Reporting Procedures

All adverse events should be reported. Depending on the nature of the event the reporting procedures below should be followed. Any questions concerning adverse event reporting should be directed to the study coordination centre in the first instance. A flowchart is given below (Figure 5) to aid in the reporting procedures and the reporting form is shown in (Data Collection Appendix).

Figure 5: Adverse event reporting flow-chart

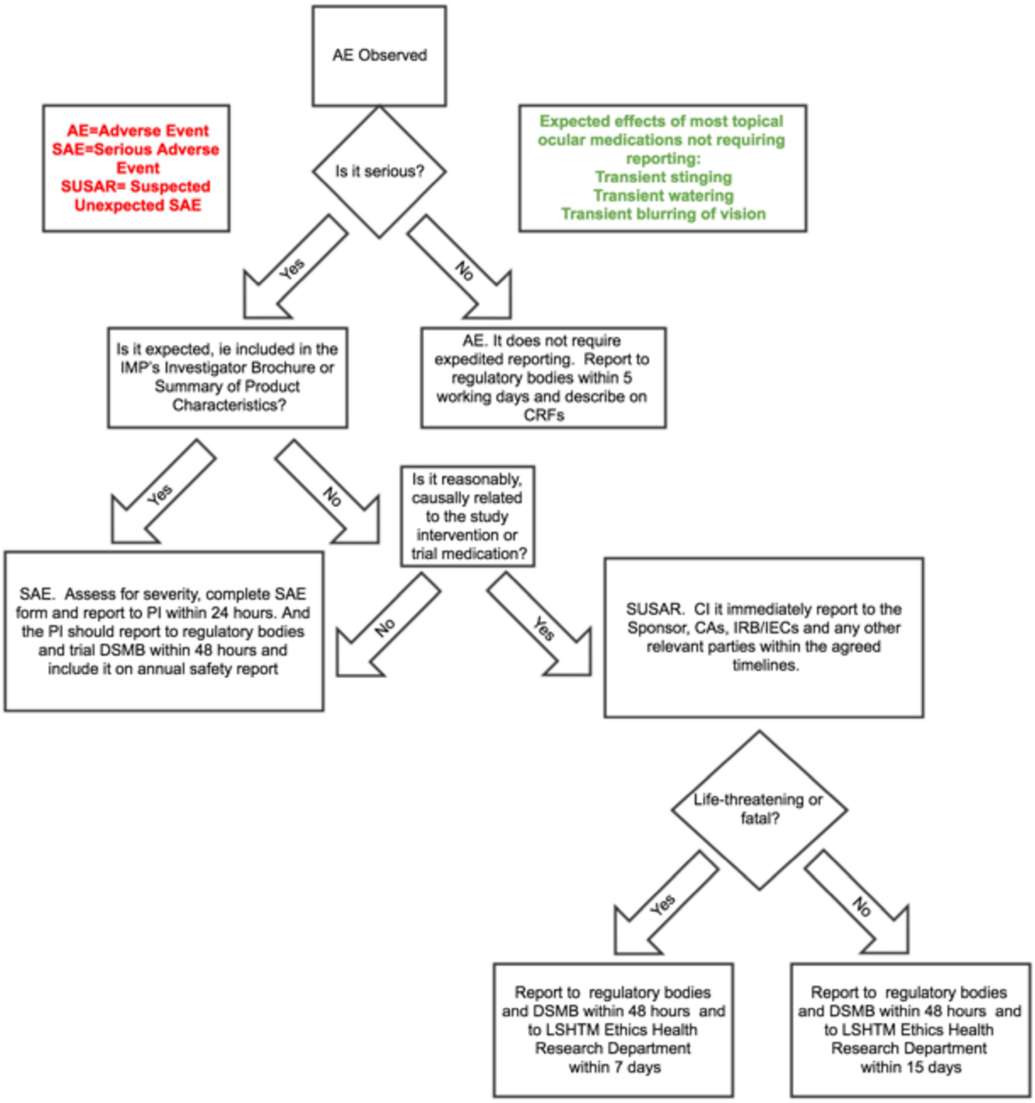

Non-serious Adverse Reactions (ARs)/Adverse Events (AEs)
All such events, whether expected or not, should be recorded in the toxicity section of the relevant case report form and sent to the study coordination centre, regulatory bodies and the trial DSMB within 5 working days from the day of occurrence.

Serious Adverse Reactions (SARs)/Serious Adverse Events (SAEs)
Fatal or life threatening Serious Adverse Events (SAEs), Serious Adverse Reactions (SARs) and Suspected Unexpected Serious Adverse Reactions (SUSARs) should be reported to the PI on the day that the local site is aware of the event. The SAE form asks for nature of event, date of onset, severity, corrective therapies given, outcome and causality (i.e. unrelated, unlikely, possible, probably, definitely). All SAEs or SARs whether expected, suspected or unexpected should be reported to regulatory bodies and the trial DSMB within 48 hours of occurrence. The responsible investigator should sign the causality of the event. Additional information should be sent within 5 days if the reaction has not resolved at the time of reporting. All investigators will be informed of all SARs occurring throughout the study.

SAEs
A SAE form should be completed and faxed to the study coordination centre for all SAEs within 24 hours. However, relapse and death due to a known pre-existing condition and hospitalisations for elective treatment of a pre-existing condition do not need reporting as SAEs.

SUSARs
In the case of serious, unexpected and related adverse events, the staff at the site should:

Complete the SAE case report form & send it immediately (within 24 hours, preferably by fax), signed and dated to the study coordination centre together with relevant treatment forms and anonymised copies of all relevant investigations.

Or

Contact the study coordination centre by phone and then send the completed SAE form to the study coordination centre within the following 24 hours as above.

The study coordination centre will notify all SUSARs occurring during the study within 48 hours to the Nepal Department of Drug Administration, and the trial DSMB. SUSARs will be reported to LSHTM Ethics Committee according to the following timelines; fatal and life-threatening within 7 days of notification and non-life threatening within 15 days. All investigators will be informed of all SUSARs occurring throughout the study.

Local investigators should report any SUSARs and /or SAEs as required by their Local Research Ethics Committee and/or Research & Development Office.

Adverse Event Reporting for this Trial
Non-serious Ocular: narrative form not required
- ☐ Local allergic reaction (conjunctiva, lid)
- ☐ Intraocular pressure ≥35 mmHg for 1 week despite therapy
- ☐ Progressive corneal thinning, ≤50% of enrolment thickness
- ☐ Other (specify)

Serious Ocular: (1) report within 24hrs (2) Serious Adverse Event (SAE) Form required
- ☐ Endophthalmitis
- ☐ Other serious ocular event thought to be related to the study drug, requiring non-elective hospitalization, loss of function, or systemic treatment (specify)

Serious Non-ocular: (1) report within 24hrs (2) Serious Adverse Event (SAE) Form required
- ☐ Death
- ☐ Non-elective surgery, hospitalization, or loss of function
- ☐ Myocardial infarction or stroke
- ☐ Systemic allergic reaction
