# Supplementary material for: Topical chlorhexidine 0.2% versus topical natamycin 5% for fungal keratitis in Nepal: rationale and design of a randomised controlled non-inferiority trial
Source: BMJ Open. 2020 Sep 30;10(9):e038066. doi: 10.1136/bmjopen-2020-038066 (PMC7528427; doi:10.1136/bmjopen-2020-038066)
Supplement: Supplementary data [file bmjopen-2020-038066supp003.pdf]

Appendix 3: Biological Specimens

The project will equip and staff an ocular microbiology laboratory at the hospital. The microbiology analysis will involve the following investigations:

1) **Microscopy** will be performed of the slides using Gram stain, KOH and calcofluor white.

2) **Culture** will be performed using a variety of media inoculated with corneal scrape samples in the clinic: blood agar, chocolate agar, brain-heart infusion broth (incubated for 2-7 days at 37°C) and Sabouraud agar (incubated for 7 days at 30°C). Culture isolates will be identified using standard microbiological techniques. Fungal and bacterial isolates will be stored frozen for long-term reference and subsequent additional analysis.

3) **Antibiotic susceptibility testing** of bacteria culture isolates will be performed using standard disc diffusion techniques in each of the laboratories.

4) **Antifungal susceptibility testing** of fungal isolates will be performed at KCMC, Tanzania . Isolates from Uganda and Nepal will be periodically transferred to KCMC, under a Material Transfer Agreement. They will be re-cultured and tested using a dilution series method for four drugs: Natamycin, Chlorhexidine, Voriconazole and Amphotericin-B. The technique has been developed at Radboud University, The Netherlands.

PCR for microorganisms

DNA from the dry swab will be extracted using the QIAamp mini kit (Qiagen) with bead-beating. Samples will be tested for the presence of bacterial (16rRNA), fungal (ITS1) and specific viral (HSV1&2) DNA by quantitative PCR. This PCR work will be performed at KCMC, Tanzania. Samples from Uganda and Nepal will be periodically transferred to KCMC for testing, under a Material Transfer Agreement. The performance of PCR as a diagnostic tool will be evaluated against a composite reference standard for positive cases. Cases will be considered positive if the pathogen is detected by either smear microscopy, IVCM or culture.

Point of care tests for fungal infection

Point-of-care tests for fungal keratitis would be very helpful. Molecular tests are generally more sensitive than antigen-based assays. Isothermal nucleic amplification techniques offer the sensitivity of PCR amplification without the need for specialized equipment. A result may be obtained within 30 minutes. Promising molecular approaches include isothermal amplification (Loop-mediated isothermal amplification (LAMP), Recombinase polymerase amplification (RPA)), associated with lateral flow assays for amplicon detection. Several assays have been developed that detect fungi, however, these have not been evaluated in fungal keratitis. The second ulcer swab will be used to test and develop isothermal molecular assays in comparison with the standard microbiological approaches described above. Results will be evaluated against a composite reference standard for fungal diagnosis as described above.

Fungal DNA sequencing

It is not always possible to determine the species of a fungus from classical microbiological techniques. It is now common to use fungal DNA sequencing for species-level diagnosis for some genera, including *Fusarium spp.*, which is the commonest fungal genus reported for MK in the study settings. We will use sequencing of the fungal ITS and TEF1α regions for this.[1] DNA will be extracted from the cultured fungal isolate when available. If there no fungus is cultured but the PCR is positive, then this PCR product will be used for sequencing. The DNA will be extracted from the isolates in KCMC, Tanzania. The extracted DNA will be sent under a Material Transfer Agreement to the Center of Expertise in Mycology Radboudumc/CWZ, Radboud University Nijmegen Medical Center and the CBS Fungal Biodiversity Centre, Utrecht, the Netherlands for the specialised sequencing and identification work.

Corneal gene expression

Three prime RNAseq (3'RNAseq) generates one read per transcript and enables strand-specific, highly multiplexed sequencing of poor concentration or quality samples (as low as 100pg total RNA input) for gene expression analysis. We have previously used this method for gene expression profiling of human conjunctival swabs from a trachoma-endemic area. RNA will be extracted from corneal swabs or scrapes using Norgen RNA purification kits. 3'RNAseq (Lexogen) will be performed on normalised RNA extracts from 500 Tanzanian and Ugandan participants with confirmed fungal keratitis. Pooled libraries will be sequenced on a HiSeq (Illumina). Transcriptomic profiles will be analysed for differences between controls and mild, moderate and severe FK cases using DEseq2, and between participants with good versus poor visual outcomes. A small number of healthy corneal tissue controls will be derived from the residual ring of tissue remaining after corneal graft surgery (ethics application for control tissue to be submitted separately).

Human Genetics

DNA will be extracted from buccal brush samples using QIAmp DNA mini kits (Qiagen). Samples from 500 confirmed fungal keratitis participants from Nepal and 500 matched controls will be run on immune-focussed Infinium ImmunoArray-24 BeadChips (Illumina) covering >240,000 genetic polymorphisms (including copy number variants, germline variants, insertions-deletions, single nucleotide polymorphisms (SNPs) and structural variants) at UCL genomics centre. In addition to SNP testing, two-field resolution Human Leukocyte Antigen (HLA) allele typing will be performed in all 1000 samples. Allele level HLA typing cannot currently be determined from SNP data but the HLA genes are primary immune response genes that have a role in many diseases and warrant independent study. Data will be analysed to determine sources of immunogenetic variation associated with fungal keratitis. These data will be linked to gene expression data from the same individuals to determine functional effects of genetic variation associated with fungal keratitis.

HIV testing

As outlined above, patients with microbial keratitis will be offered counselling and testing for HIV infection. This will be done through the established services in each of the hospitals. Appropriate onward referrals will be made if a test is found to be positive.

Blood glucose testing

Patients will have capillary blood glucose (CBG) testing done at presentation. Patients with an elevated CBG (>6.1mmol) will be referred to the hospital physicians for assessment and formal diagnosis of impaired glucose tolerance or diabetes mellitus. This level is considered a suitable cut-off to detect individuals with diabetes and has been validated in a south-Asian population.[2]

References:

1 Schoch CL, Seifert KA, Huhndorf S, *et al.* Nuclear ribosomal internal transcribed spacer (ITS) region as a universal DNA barcode marker for Fungi. *Proc Natl Acad Sci USA* 2012;**109**:6241–6. doi:10.1073/pnas.1117018109

2 Somannavar S, Ganesan A, Deepa M, *et al.* Random capillary blood glucose cut points for diabetes and pre-diabetes derived from community-based opportunistic screening in India. *Diabetes Care* 2009;**32**:641–3. doi:10.2337/dc08-0403
