# Supplementary material for: Topical chlorhexidine 0.2% versus topical natamycin 5% for fungal keratitis in Nepal: rationale and design of a randomised controlled non-inferiority trial
Source: BMJ Open. 2020 Sep 30;10(9):e038066. doi: 10.1136/bmjopen-2020-038066 (PMC7528427; doi:10.1136/bmjopen-2020-038066)
Supplement: Supplementary data [file bmjopen-2020-038066supp004.pdf]

Appendix 4: SPIRIT Checklist for Topical chlorhexidine 0.2% versus topical natamycin 5% for fungal keratitis in Nepal randomised clinical trial

Section / Item	Item No.	Description	Location Reference
**Administrative information**			
Title	1	Descriptive title identifying the study design, population, interventions, and, if applicable, trial acronym	Page 1 of study protocol
Trial registration	2a	Trial identifier and registry name. If not yet registered, name of intended registry	ISRCTN14332621; pre-results
	2b	All items from the World Health Organization Trial Registration Data Set	ISRCTN14332621 Study Registry
Protocol Version	3	Date and version identifier	Every page of study protocol
Funding	4	Sources and types of financial, material, and other support 5a Names, affiliations, and roles of protocol contributors	Section 5.3 of study protocol, page 25<br>Page 17 of Manuscript
Roles and Responsibilities	5a	Names, affiliations, and roles of protocol contributors	Page 1 of study protocol<br>Page 1 of Manuscript
	5b	Name and contact information for the trial sponsor	Page 1 of study protocol<br>Page 1 of Manuscript
	5c	Role of study sponsor and funders, if any, in study design; collection, management, analysis, and interpretation of data; writing of the report; and the decision to submit the report for publication, including whether they will have ultimate authority over any of these activities	Page 1 of study protocol and Section 5.2 of study protocol, page 25.<br><br>Page 17 of Manuscript
	5d	Composition, roles, and responsibilities of the coordinating centre, steering committee, endpoint adjudication committee, data management team, and other individuals or groups overseeing the trial, if applicable (see Item 21a for data monitoring committee)	Section 5.1, page 24 of study protocol<br><br>Section 6, page 25 of study protocol<br><br>Page 11 of Manuscript
**Introduction**			
Background and rationale	6a	Description of research question and justification for undertaking the trial, including summary of relevant studies (published and unpublished) examining benefits and harms for each intervention	Section 1, pages 6-9 of study protocol<br><br>Pages 4-5 of Manuscript
	6b	Explanation for choice of comparators	Sections 1.3 – 1.4, pages 7-9 of study protocol<br>Pages 4-5 of Manuscript

Objectives	7	Specific objectives or hypotheses	Section 3, page 11 of study protocol<br><br>Page 5 of Manuscript
Trial design	8	Description of trial design including type of trial (eg, parallel group, crossover, factorial, single group), allocation ratio, and framework (eg, superiority, equivalence, noninferiority, exploratory)	Section 4, pages 12-24 of study protocol<br><br>Page 6 of Manuscript
**Methods: Participants, interventions, and outcomes**			
Study setting	9	Description of study settings (eg, community clinic, academic hospital) and list of countries where data will be collected. Reference to where list of study sites can be obtained	Section 4.3, page 13 of study protocol<br><br>Page 6 of Manuscript
Eligibility criteria	10	Inclusion and exclusion criteria for participants. If applicable, eligibility criteria for study centres and individuals who will perform the interventions (eg, surgeons, psychotherapists)	Section 4.4, page 13 of study protocol<br><br>Page 7 of Manuscript and Table 1 of Manuscript
Interventions	11a	Interventions for each group with sufficient detail to allow replication, including how and when they will be administered	Section 4.7, page 17 of study protocol<br><br>Pages 8-9 of Manuscript
	11b	Criteria for discontinuing or modifying allocated interventions for a given trial participant (eg, drug dose change in response to harms, participant request, or improving/worsening disease)	Page 11 of Manuscript
	11c	Strategies to improve adherence to intervention protocols, and any procedures for monitoring adherence (eg, drug tablet return, laboratory tests)	Section 4.9, page 19 of study protocol<br><br>Pages 10-11 of Manuscript
	11d	Relevant concomitant care and interventions that are permitted or prohibited during the trial	Section 4.7, pages 17-18 of study protocol<br>Pages 9-10 of Manuscript
Outcomes	12	Primary, secondary, and other outcomes, including the specific measurement variable (eg, systolic blood pressure), analysis metric (eg, change from baseline, final value, time to event), method of aggregation (eg, median, proportion), and time point for each outcome. Explanation of the clinical relevance of chosen efficacy and harm outcomes is strongly recommended	Section 4.11, page 22 of study protocol<br><br>Pages 10-11 of Manuscript

Participant timeline	13	Time schedule of enrolment, interventions (including any run-ins and washouts), assessments, and visits for participants. A schematic diagram is highly recommended (see Figure)	Table 3, page 19 of study protocol<br><br>Table 4 of Manuscript
Sample size	14	Estimated number of participants needed to achieve study objectives and how it was determined, including clinical and statistical assumptions supporting any sample size calculations	Section 4.12, page 22 of study protocol<br><br>Pages 12 -13 of Manuscript
Recruitment	15	Strategies for achieving adequate participant enrolment to reach target sample size	Section 4.5, page 14 of study protocol<br><br>Page 7 of Manuscript
**Methods: Assignment of interventions (for controlled trials)**			
Allocation: sequence generation	16a	Method of generating the allocation sequence (eg, computer- generated random numbers), and list of any factors for stratification. To reduce predictability of a random sequence, details of any planned restriction (eg, blocking) should be provided in a separate document that is unavailable to those who enrol participants or assign interventions	Section 4.8, pages 18-19 of study protocol<br><br>Pages 7-8 of Manuscript
Allocation: concealment mechanism	16b	Mechanism of implementing the allocation sequence (eg, central telephone; sequentially numbered, opaque, sealed envelopes), describing any steps to conceal the sequence until interventions are assigned	
Allocation: Implementation	16c	Who will generate the allocation sequence, who will enrol participants, and who will assign participants to interventions	
Blinding (masking)	17a	Who will be blinded after assignment to interventions (eg, trial participants, care providers, outcome assessors, data analysts), and how	Section 4.8, pages 18-19 of study protocol<br><br>Page 8 of Manuscript
	17b	If blinded, circumstances under which unblinding is permissible, and procedure for revealing a participant's allocated intervention during the trial	Page 8 of Manuscript
**Methods: Data collection, management, and analysis**			
	18a	Plans for assessment and collection of outcome, baseline, and other trial data, including any	Section 4.14, page 23 of study protocol

Data collection methods		related processes to promote data quality (eg, duplicate measurements, training of assessors) and a description of study instruments (eg, questionnaires, laboratory tests) along with their reliability and validity, if known. Reference to where data collection forms can be found, if not in the protocol	Page 12 of Manuscript
	18b	Plans to promote participant retention and complete follow-up, including list of any outcome data to be collected for participants who discontinue or deviate from intervention protocols	Page 12 of Manuscript
Data management	19	Plans for data entry, coding, security, and storage, including any related processes to promote data quality (eg, double data entry; range checks for data values). Reference to where details of data management procedures can be found, if not in the protocol	Section 4.14, page 23 of study protocol<br><br>Page 12 of Manuscript
Statistical methods	20a	Statistical methods for analysing primary and secondary outcomes. Reference to where other details of the statistical analysis plan can be found, if not in the protocol	Section 4.13, pages 22-23 of study protocol<br><br>Pages 12-15 of Manuscript
	20b	Methods for any additional analyses (eg, subgroup and adjusted analyses)	
	20c	Definition of analysis population relating to protocol non-adherence (eg, as randomised analysis), and any statistical methods to handle missing data (eg, multiple imputation)	
**Methods: Monitoring**			
Data Monitoring	21a	Composition of data monitoring committee (DMC); summary of its role and reporting structure; statement of whether it is independent from the sponsor and competing interests; and reference to where further details about its charter can be found, if not in the protocol. Alternatively, an explanation of why a DMC is not needed	Section 6, page 25 of study protocol<br><br>Page 12 of Manuscript
	21b	Description of any interim analyses and stopping guidelines, including who will have access to	Section 4.13, page 23 of study protocol

		these interim results and make the final decision to terminate the trial	Page 15 of Manuscript
Harms	22	Plans for collecting, assessing, reporting, and managing solicited and spontaneously reported adverse events and other unintended effects of trial interventions or trial conduct	Section 7, pages 26-28 of study protocol<br><br>Page 12 of Manuscript<br><br>Annex 2
Auditing	23	Frequency and procedures for auditing trial conduct, if any, and whether the process will be independent from investigators and the sponsor	Section 6, page 25 of study protocol<br><br>Page 12 of Manuscript
**Ethics and Dissemination**			
Research ethics approval	24	Plans for seeking research ethics committee/institutional review board (REC/IRB) approval	Section 4.15, page 24 of study protocol<br>Page 15 of Manuscript
Protocol amendments	25	Plans for communicating important protocol modifications (eg, changes to eligibility criteria, outcomes, analyses) to relevant parties (eg, investigators, REC/IRBs, trial participants, trial registries, journals, regulators)	Section 4.15, page 24 of study protocol<br>Page 15 of Manuscript
Consent or assent	26a	Who will obtain informed consent or assent from potential trial participants or authorised surrogates, and how (see Item 32)	Section 4.5, page 14 of study protocol<br><br>Page 7 of Manuscript<br><br>Appendix 1: Consent Forms
	26b	Additional consent provisions for collection and use of participant data and biological specimens in ancillary studies, if applicable	
Confidentiality	27	How personal information about potential and enrolled participants will be collected, shared, and maintained in order to protect confidentiality before, during, and after the trial	Page 12 of Manuscript
Declaration of interests	28	Financial and other competing interests for principal investigators for the overall trial and each study site	Page 18 of submitted manuscript
Access to data	29	Statement of who will have access to the final trial dataset, and disclosure of contractual agreements that limit such access for investigators	Page 12 of Manuscript
Ancillary and post-trial care	30	Provisions, if any, for ancillary and post-trial care, and for compensation to those who suffer harm from trial participation	Page 9 of Manuscript
Dissemination policy	31a	Plans for investigators and sponsor to communicate trial results to participants, healthcare	Section 10, page 33 of study protocol

		professionals, the public, and other relevant groups (eg, via publication, reporting in results databases, or other data sharing arrangements), including any publication restrictions	Pages 15-16 of Manuscript
	31b	Authorship eligibility guidelines and any intended use of professional writers	Page 24 of Manuscript
	31c	Plans, if any, for granting public access to the full protocol, participant- level dataset, and statistical code	Page 24 of Manuscript
**Appendices**			
Informed consent materials	32	Model consent form and other related documentation given to participants and authorised surrogates	Appendix 1 of Manuscript
Biological specimens	33	Plans for collection, laboratory evaluation, and storage of biological specimens for genetic or molecular analysis in the current trial and for future use in ancillary studies, if applicable	Appendix 3 of Manuscript
