# Supplementary material for: Topical chlorhexidine 0.2% versus topical natamycin 5% for fungal keratitis in Nepal: rationale and design of a randomised controlled non-inferiority trial
Source: BMJ Open. 2020 Sep 30;10(9):e038066. doi: 10.1136/bmjopen-2020-038066 (PMC7528427; doi:10.1136/bmjopen-2020-038066)
Supplement: Author's manuscript [file bmjopen-2020-038066.draft_revisions.pdf]

BMJ Open

BMJ Open is committed to open peer review. As part of this commitment we make the peer review history of every article we publish publicly available.

When an article is published we post the peer reviewers' comments and the authors' responses online. We also post the versions of the paper that were used during peer review. These are the versions that the peer review comments apply to.

The versions of the paper that follow are the versions that were submitted during the peer review process. They are not the versions of record or the final published versions. They should not be cited or distributed as the published version of this manuscript.

BMJ Open is an open access journal and the full, final, typeset and author-corrected version of record of the manuscript is available on our site with no access controls, subscription charges or pay-per-view fees (http://bmjopen.bmj.com).

If you have any questions on BMJ Open's open peer review process please email info.bmjopen@bmj.com

BMJ Open

# Topical chlorhexidine 0.2% versus topical natamycin 5% for fungal keratitis in Nepal: rationale and design of a randomised controlled non-inferiority trial

| | |
|---|---|
| Journal: | *BMJ Open* |
| Manuscript ID | bmjopen-2020-038066 |
| Article Type: | Protocol |
| Date Submitted by the Author: | 26-Feb-2020 |
| Complete List of Authors: | Hoffman, Jeremy; London School of Hygiene and Tropical Medicine International Centre for Eye Health; Sagarmatha Choudhary Eye Hospital<br>Yadav, Reena; Sagarmatha Choudhary Eye Hospital<br>Das Sanyam, Sandip; Sagarmatha Choudhary Eye Hospital<br>Chaudhary, Pankaj; Sagarmatha Choudhary Eye Hospital<br>Roshan, Abhishek; Sagarmatha Choudhary Eye Hospital<br>Singh, Sanjay; Eastern Region Eye Care Programme<br>Arunga, Simon; London School of Hygiene and Tropical Medicine International Centre for Eye Health, International Centre for Eye Health; Mbarara University of Science and Technology Faculty of Medicine<br>Matayan, Einoti; Kilimanjaro Christian Medical Centre, Department of Ophthalmology<br>Macleod, David; London School of Hygiene & Tropical Medicine, MRC Tropical Epidemiology Group<br>Weiss, Helen; London School of Hygiene & Tropical Medicine, MRC Tropical Epidemiology Group<br>Leck, Astrid; London School of Hygiene and Tropical Medicine International Centre for Eye Health<br>Hu, Victor; London School of Hygiene and Tropical Medicine International Centre for Eye Health<br>Burton, Matthew J; London School of Hygiene and Tropical Medicine International Centre for Eye Health; Moorfields Eye Hospital NHS Foundation Trust |
| Keywords: | Corneal and external diseases < OPHTHALMOLOGY, OPHTHALMOLOGY, Clinical trials < THERAPEUTICS, MYCOLOGY |
| | |

SCHOLARONE™
Manuscripts

*I, the Submitting Author has the right to grant and does grant on behalf of all authors of the Work (as defined in the below author licence), an exclusive licence and/or a non-exclusive licence for contributions from authors who are: i) UK Crown employees; ii) where BMJ has agreed a CC-BY licence shall apply, and/or iii) in accordance with the terms applicable for US Federal Government officers or employees acting as part of their official duties; on a worldwide, perpetual, irrevocable, royalty-free basis to BMJ Publishing Group Ltd ("BMJ") its licensees and where the relevant Journal is co-owned by BMJ to the co-owners of the Journal, to publish the Work in this journal and any other BMJ products and to exploit all rights, as set out in our licence.*

*The Submitting Author accepts and understands that any supply made under these terms is made by BMJ to the Submitting Author unless you are acting as an employee on behalf of your employer or a postgraduate student of an affiliated institution which is paying any applicable article publishing charge ("APC") for Open Access articles. Where the Submitting Author wishes to make the Work available on an Open Access basis (and intends to pay the relevant APC), the terms of reuse of such Open Access shall be governed by a Creative Commons licence – details of these licences and which Creative Commons licence will apply to this Work are set out in our licence referred to above.*

*Other than as permitted in any relevant BMJ Author's Self Archiving Policies, I confirm this Work has not been accepted for publication elsewhere, is not being considered for publication elsewhere and does not duplicate material already published. I confirm all authors consent to publication of this Work and authorise the granting of this licence.*

For peer review only

# Topical chlorhexidine 0.2% versus topical natamycin 5% for fungal keratitis in Nepal: rationale and design of a randomised controlled non-inferiority trial

**Authors:**

Jeremy J Hoffman FRCOphth (1, 2, 5), Reena Yadav MD (2), Sandip Das Sanyam (2), Pankaj Chaudhary (2), Abhishek Roshan (2), Sanjay K Singh MD (3), Simon Arunga (1, 4), Einoti Matayan (5), David Macleod PhD (6), Helen A Weiss PhD (6), Astrid Leck PhD (1), Victor H Hu PhD FRCOphth (1), Matthew J Burton PhD FRCOphth (1, 7).

**Affiliations:**

1. International Centre for Eye Health, London School of Hygiene and Tropical Medicine, London, UK
2. Sagarmatha Choudhary Eye Hospital, Lahan, Nepal
3. Eastern Region Eye Care Programme, Biratnagar, Nepal
4. Mbarara University of Science and Technology, Mbarara, Uganda
5. Kilimanjaro Christian Medical Centre, Moshi, Tanzania
6. MRC Tropical Epidemiology Group, London School of Hygiene & Tropical Medicine, London, UK
7. Moorfields Eye Hospital, London, UK

**Corresponding author:**

Jeremy J Hoffman FRCOphth

International Centre for Eye Health

London School of Hygiene and Tropical Medicine

Keppel Street

London

WC1E 7HT

United Kingdom

jeremy.hoffman@lshtm.ac.uk

**Key words:** Clinical trial; fungal keratitis; chlorhexidine; natamycin; corneal ulcer, Nepal.

**Word count:** 5138 (excluding abstract, titles, tables, figures and references)

For peer review only

## ABSTRACT

### Introduction

Fungal infections of the cornea, fungal keratitis (FK), are challenging to treat. Current topical antifungals are not always effective and are often unavailable, particularly in low and middle-income countries where most cases occur. G-natamycin 5% is usually first-line treatment, however, even when treated intensively, infections may progress to perforation of the eye in around a quarter of cases. Alternative anti fungal medications are needed to treat this blinding disease.

Chlorhexidine is an antiseptic agent with antibacterial and antifungal properties. Previous pilot studies suggest that g-chlorhexidine 0.2% compares favourably with g-natamycin. Full-scale randomised controlled trials (RCT) of g-chlorhexidine 0.2% are warranted to answer this question definitively.

### Methods and analysis

We will test the hypothesis that g-chlorhexidine 0.2% is non-inferior to g-natamycin 5% in a two-arm, single-masked RCT. Participants are adults with FK presenting to a tertiary ophthalmic hospital in Nepal. Baseline assessment includes history, examination, photography, *in vivo* confocal microscopy and cornea scrapes for microbiology. Participants will be randomised to alternative topical antifungal treatments (g-chlorhexidine 0.2% and g-natamycin 5%; 1:1 ratio, 2-6 random block size). Patients are reviewed at day 2, day 7 (with re-culture), day 14, day 21, month 2 and month 3. The primary outcome is best spectacle corrected visual acuity (BSCVA) at three months. Primary analysis (intention-to-treat) will be by linear regression, with treatment arm and baseline BSCVA pre-specified covariates. Secondary outcomes include epithelial healing time, scar/infiltrate size, ulcer depth, hypopyon size, perforation and/or therapeutic penetrating keratoplasty (TPK, corneal transplant), positive re-culture rate (day-7) and quality of life (EQ-5D, WHO/PBD-VF20, WHOQOL-BREF).

### Ethics and dissemination

The Nepal Health Research Council, the Nepal Department of Drug Administration and the London School of Hygiene and Tropical Medicine ethics committee have approved the trial. The results will be presented at local and international meetings and submitted to peer-reviewed journals for publication.

### Trial Registration

ISRCTN14332621; pre-results.

### Article summary

For peer review only

- First large-scale randomised controlled clinical trial comparing chlorhexidine 0.2% to natamycin 5% for the treatment of fungal keratitis.
- This study benefits from a pragmatic design: as a non-inferiority trial, if chlorhexidine is found to be within a pre-defined non-inferiority margin of 0.15 logMAR of natamycin at 3 months a recommendation to use chlorhexidine 0.2% can be made; this is a far cheaper, easy to formulate medication that could significantly increase access to anti fungal treatment for the target population.
- Clinicians are masked to the treatment allocation, however, due to different physical appearance it is not possible to mask patients to their allocated treatment.
- This study will also assess the superiority of either medication as well as a number of key secondary outcome measures, analysed by arm.
- First randomised controlled trial investigating fungal keratitis to use *in vivo* confocal microscopy as a diagnostic tool for the detection of fungal elements

For peer review only

## INTRODUCTION

Fungal keratitis (FK) is a severe and potentially blinding corneal infection (Figure 1). [1,2] The burden is greatest in tropical and subtropical countries, probably due to a combination of climate (higher temperatures and humidity) and frequent agriculture related eye injuries. [3] It is one of the causes of microbial keratitis and accounts for between 20 – 60% of corneal infections diagnosed in tropical regions. [4] It is often inadequately treated with significant barriers to receiving appropriate, timely intervention, compounded by indiscriminate and inappropriate use of conventional medicines such as topical corticosteroids or harmful traditional eye medicines. [1,2,5] Furthermore, when appropriate treatment is available, up to 30% of patients receiving current "gold-standard" therapy progress to corneal perforation and / or eye-loss (Figure 2). [1,6,7]

Treatment for fungal keratitis almost always involves topical antifungal agents. Surgical intervention, usually in the form of therapeutic penetrating keratoplasty (TPK), is generally reserved for cases of corneal perforation or progressive infection refractory to medical therapy. Corneal transplantation is also performed for visual rehabilitation after the acute infection has resolved. There are a limited number of antifungals available for treating FK, which fall into four main groups: imidazoles, triazoles, polyenes and fluorinated pyrimidines. These may be available topically, orally or by intravenous injection. Subconjunctival, corneal stromal or intracameral injections may also be given. The treatment of yeasts (*Candida spp.*) is often different to filamentous fungi, with the former being more common in temperate climates and the latter in hot and humid locations.[8,9]

There have been several clinical trials comparing treatment options for FK, which have been systematically reviewed.[10,11] Natamycin, which was approved in the 1960s by the FDA for FK, has been compared to a number of newer agents, including voriconazole. Natamycin and voriconazole have been compared in four trials, with the meta-analysis favouring natamycin. [6,10,12-14]

As a result, first line management of filamentous FK is usually with topical natamycin 5% when this is available. This was added to the WHO Essential Medicines List in 2017 for this indication. However, even when intensive topical natamycin is initiated, infections frequently progress relentlessly to perforation and loss of the eye in about a quarter of cases, Figure 2. [1,6,7] Moreover, in many countries antifungal eye drop treatments are simply not available. This includes most countries in sub-Saharan Africa, some Asian countries and some countries in Europe.[1,2] Natamycin is relatively expensive even if it is available. Alternative affordable antifungal therapies are clearly needed if the outcome of these infections is to improve.


For peer review only - http://bmjopen.bmj.com/site/about/guidelines.xhtml

Chlorhexidine (CHX) is an antiseptic agent, with both antibacterial and antifungal properties. It is a widely-used broad-spectrum biocide, killing microorganisms through cell membrane disruption. [15-17] CHX has been used in ophthalmology for over 30 years as an eye-drop preservative and for sterilizing contact lenses, and has also been used to treat *Acanthamoeba* and fungal keratitis. [9,18-22] In a study of potential anti fungal treatments, CHX was effective both *in vitro* against MK fungal isolates from India and Ghana, as well as in an Indian case series.[23] Subsequently two pilot RCTs of CHX for fungal keratitis were conducted. In the first, three CHX concentrations (0.05%, 0.1%, 0.2%) were compared to each other and natamycin 5%; this concluded CHX 0.2% had reliable antifungal action. [19] In the second trial, CHX 0.2% was compared to topical natamycin 2.5% (half standard concentration). There was evidence CHX produced a more favourable response by 5 days (RR=0.23, 95%CI 0.09-0.63). [18] A systematic review found a trend favouring CHX over natamycin in "curing" by 21-days (RR=0.70, 95%CI 0.45-1.09), suggesting CHX might prove superior in adequately powered trials.[10] CHX is safe and well-tolerated at these concentrations. [18,19,22,24] Based on this, CHX is used for treating fungal MK in several countries. [1,9,11] However, the combined size of these two pilot trials comparing chlorhexidine and natamycin is not sufficient to reach firm conclusions. Currently, the meta-analysis indicates equipoise in terms of which treatment is best for treating fungal keratitis.

**Objectives**

The primary objective of this study is to determine if topical chlorhexidine 0.2% is non-inferior to topical natamycin 5% for treating fungal keratitis, in terms of vision at three months. The secondary objectives are: (1) to determine whether either treatment (chlorhexidine 0.2% or natamycin 5%) is superior to the other, in terms of vision at three months; and (2) to determine whether there is a difference between chlorhexidine 0.2% and natamycin 5% in terms of secondary clinical outcomes including infiltrate / scar size, time to re-epithelialisation, re-culture rates at one week and the effect of the alternative treatments on the Quality of Life of participants.

Chlorhexidine is cheap, stable and easily prepared by aqueous dilution. If chlorhexidine is found to be non-inferior (or even superior) to natamycin this offers the potential of an effective, affordable and accessible treatment for fungal keratitis, which could benefit millions of people each year who currently have no treatment options. This trial is a response to this expressed need from both clinicians and patients for a readily available and affordable medication for fungal infection.



For peer review only - http://bmjopen.bmj.com/site/about/guidelines.xhtml

## METHODS AND ANALYSIS

### Trial Design

We are conducting a single-masked, non-inferiority randomised controlled trial comparing chlorhexidine 0.2% to natamycin 5% for the treatment of fungal keratitis. The non-inferiority design of this trial offers a clinically pragmatic way to address this important question: if CHX is found to be within the pre-specified non-inferiority margin of 0.15 logMAR (about 1.5 Snellen lines) then CHX may prove to be a sustainable solution for this aspect of the complex problem of fungal keratitis.

### Trial Summary

This RCT follows a two-stage recruitment process (Figure 3). All patients presenting with acute microbial keratitis are reviewed and enrolled into Stage 1, following written, informed consent. This involves history, examination and investigations (corneal scrapes for microbiological assessment and *in vivo* confocal microscopy). If there is evidence of fungal hyphae on smear or confocal microscopy, patients then proceed to Stage 2. A trial eligibility checklist is completed and Stage 2 written informed consent is conducted. We will recruit 500 patients into Stage 2. Eligible FK patients are then randomised 1:1 to receive either natamycin 5% or chlorhexidine 0.2% topical treatments hourly for the first week, then two-hourly for the subsequent two weeks. Ongoing treatment duration will then be tailored to clinical response. Study personnel are masked to the treatment allocation. Patients are usually initially admitted and followed up on day-2, day-7 (with re-culture), day-14, day-21, month-2 and month-3. The primary outcome is best spectacle corrected visual acuity (BSCVA) at three months.

### Trial Setting

Low-land Nepal, a region with a high burden of fungal keratitis, provides a suitable location to conduct a clinical trial that requires a relatively large sample size. This trial will be conducted in Sagarmatha Choudhary Eye Hospital (SCEH), Lahan, Siraha District in south eastern Nepal. SCEH treats approximately 800 cases of keratitis per month, half of which is attributable to fungal infection. [1,25] SCEH is a tertiary level ophthalmic hospital, with several satellite Eye Care Clinics (ECCs) that refer patients to SCEH directly as required. SCEH and its ECCs serve an estimated population of about 5 million people. Due to its proximity to the border, approximately 50% of the outpatients are Indian nationals. It is anticipated that the study participants will present to the hospital from multiple districts within the region. Potentially eligible individuals will be recruited from outpatient clinics or referred directly by the ECCs to the study team. Based on the numbers of patients attending, it should be possible to complete recruitment of 500 participants within six to twelve months. A second separate trial in East Africa (Tanzania and Uganda) will also be conducted to compare chlorhexidine 0.2% to natamycin 5% following a very similar protocol to the one described here. This will be

For peer review only

registered as a separate trial. This will enable us to assess the generalisability of the findings in two geographically distinct regions with potentially different fungal aetiologies and susceptibility patterns.

**Eligibility Criteria**

Potential participants need to meet all the inclusion criteria and have none of the exclusion criteria listed in Table 1. In summary, they need to have an active fungal keratitis defined as acute microbial keratitis characterised by corneal epithelial ulceration (>1mm in diameter), corneal stromal infiltrate and signs of acute inflammation (e.g. conjunctival injection, anterior chamber inflammatory cells, hypopyon) in conjunction with evidence of a filamentous fungal infection on smear microscopy and/or *in vivo* confocal microscopy.

**Table 1: Inclusion and exclusion criteria for enrolment in Stage 1 (microbial keratitis cases) and Stage 2 (the randomised controlled trial)**

| **Stage 1:** | |
|---|---|
| **Inclusion Criteria (all must be met):** | **Exclusion Criteria (any of the following):** |
| 1. Acute MK characterised by:<br>• Corneal epithelial ulceration >1mm diameter<br>• Corneal stromal infiltrate<br>• Acute inflammation: e.g. conjunctival injection, anterior chamber inflammatory cells, hypopyon.<br>2. Adults (18 years and older)<br>3. Able to provide informed consent | 1. Patients aged less than 18 years<br>2. Patients unable or unwilling to provide informed consent<br>3. Patients who do not have acute microbial keratitis or where there is a more likely alternative diagnosis |
| **Stage 2:** | |
| **Inclusion Criteria (all must be met):** | **Exclusion Criteria (any of the following):** |
| 1. Acute MK characterised by:<br>• Corneal epithelial ulceration >1mm diameter<br>• Corneal stromal infiltrate<br>• Acute inflammation: e.g. conjunctival injection, anterior chamber inflammatory cells, hypopyon.<br>2. Filamentous fungal hyphae visualised on smear microscopy and/or *in vivo* confocal microscopy.<br>3. Agree to be randomised to either treatment arm and are able to give informed consent<br>4. Agree to be followed up at 2 days, 1 week, 2 weeks, 3 weeks, 2 months and 3 months<br>5. Adults (18 years and older) | 1. Unwilling/unable to participate in trial and/or attend follow-up<br>2. Aged less than 18 years<br>3. Pregnancy: self-reported, or by urine hCG pregnancy test if uncertain.<br>4. Breast feeding: self-reported<br>5. Prior topical anti fungal treatment<br>6. No light perception in the affected eye<br>7. Fellow eye visual acuity <6/60<br>8. Acanthamoebic infection visualised by smear microscopy or IVCM<br>9. Clinical evidence of herpetic keratitis<br>10. Known allergy to study medication (including preservatives)<br>11. Previous keratoplasty in the affected eye<br>12. Bilateral corneal ulcers<br>13. Very severe ulcers warranting immediate evisceration or conjunctival flap<br>14. Endophthalmitis |



For peer review only - http://bmjopen.bmj.com/site/about/guidelines.xhtml

**Consent Procedures**

There are two independent consent stages in this trial: Stage 1, where all adult patients with microbial keratitis are eligible; and Stage 2, only for fungal keratitis patients meeting the eligibility criteria. The two-stage process enables data collection on all potential patients at baseline before a diagnosis of fungal keratitis is confirmed. All patients who are eligible to participate will be given a participant information sheet in Nepali and its contents read to them. There will be an opportunity to discuss any questions that they might have. If the patient would like to participate, they will be asked to read and sign or place a thumb print on the study consent form. The consent will be witnessed by the eye health worker, confirmed by a signature on the form. For patients who are unable to read the documentation a second witness who is unrelated to the study is required. Consent Forms are given in Appendix 1.

**Baseline Assessment**

The detailed baseline assessment is described in Table 2. This includes clinical examination, corneal photography, *in vivo* confocal microscopy and the collection of microbiology samples. Quality of life questionnaires will also be completed (EQ-5D, WHO/PBD-VF20, WHOQOL-BREF).

**Table 2: Baseline assessment.** Assessment performed at baseline with details of how they are made.

| Assessment | Details |
|---|---|
| Visual Acuity | Presenting, Pin-Hole and Best Spectacle Corrected Visual Acuity (BSCVA) will be measured using an ETDRS Tumbling-E logMAR 3m chart (Good-Lite Inc., USA) mounted on an ESC 2000 ETDRS LED Cabinet, (Good-Lite Inc, USA) by a trial-certified optometrist, for each eye separately |
| Contrast Sensitivity | Measured using the Peek Contrast Sensitivity [26] smartphone application running on Android OS with a Sony Xperia Z3 Compact smartphone (Sony, Japan). |
| Clinical Photographs | Photographs will be taken separately of both corneas using a Nikon D7500 camera with an AF-S Micro Nikkor 105mm lens and lens mounted SB-200 flash units (Nikon, Japan). A standardised photography protocol is used to ensure images can be compared between time points. Standardised magnification will be used to allow epithelial defect and stromal infiltrate size measurements to be made. |
| Slit-lamp examination | Both eyes will be examined using a slit-lamp biomicroscope (standard ophthalmology examination) to assess the anterior segment of the eye. This examination will be performed by an ophthalmic clinician experienced in managing microbial keratitis. Particular attention will be paid to the following features:<br>1. Eyelids: trichiasis, lagophthalmos, facial weakness, Bell's reflex<br>2. Suppuration<br>3. Conjunctival inflammation<br>4. Corneal sensation<br>5. Cornea epithelial defect (measuring the longest dimension and the longest perpendicular) and ulcer depth<br>6. Corneal inflammatory infiltrate depth, size, profile, colour, edge pattern, texture, satellites.<br>7. Anterior chamber inflammatory cells, hypopyon, endothelial plaque<br>8. Relative afferent pupillary defect. |
| *In vivo* confocal microscopy (IVCM) | The Heidelberg Retinal Tomograph 3 (HRT3) *in vivo* confocal microscope enables the clinician to examine the cornea down to the cellular level. It is able to detect the presence of fungal hyphae.[27,28] A sterile, single-use disposable cap covers the objective lens and changed between patients. Volume scans will be performed which provide a series of 400 x 400µm |



For peer review only - http://bmjopen.bmj.com/site/about/guidelines.xhtml

| | |
|---|---|
| | images over a depth range of 80µm. The resolution of the corneal scanning module is 7.6µm. IVCM images will be collected in a systematic way, starting at the centre of the ulcer, then at the superior, inferior, nasal and temporal borders of the ulcer. Volume scans will be performed in all of these locations, starting at the level of the corneal epithelium, and ending at the deepest affected aspect of the cornea assessed from IVCM images. Images will be assessed during the examination. |
| Ocular Sample Collection | The following samples will be collected from the corneal ulcer of each patient at the baseline assessment:<br>1. Corneal scrape specimens for microscopy and microbiological culture. A corneal scrape will be collected from the corneal ulcer after application of preservative free proxymetacaine local anaesthetic eye drops (Minims). Sterile needles are used to take corneal scrape specimens and then place on to glass slides for immediate gram stain, 10% KOH, lactophenol cotton blue and calcofluor white. Samples will be directly inoculated onto blood, chocolate, Sabouraud dextrose agar and brain heart infusion broth for culture.<br>2. Corneal specimen collection for PCR. Two sterile swabs will be gently swept over the surface of the corneal ulcer and placed into a 2ml tube. The swabs will be for pathogen detection by PCR, fungal sequencing and assessment of point of care tests for fungal infections. Swabs will be stored dry at -80°C. If swab yields are found to be too low for analysis an additional corneal scrape will be collected for PCR. The analysis of the PCR samples will not form part of the RCT work up and report. |
| HIV Testing | All individuals presenting with MK would be offered counselling and testing services. If this is found to be positive and the patient is unknown to the HIV care services and appropriate referral will be made. HIV testing is performed using HIV Tri-Dot rapid diagnostic test (J. Mitra & Co. Pvt. Ltd., India) |
| Random Blood Glucose | There is a suggestion that individuals with diabetes may be more susceptible to fungal keratitis. Participants will be offered a random blood glucose test, on a finger prick sample, analysed using HumaLyzer Primus (HUMAN Gesellschaft für Biochemica und Diagnostica mbH, Germany). If this is above 6.1 mmol/l they will be referred to the hospital physicians for assessment and formal diagnosis of impaired glucose tolerance or diabetes mellitus. This level is considered a suitable cut-off to detect individuals with diabetes and has been validated in a south-Asian population.[29] |
| Quality of Life Questionnaires | For those with confirmed fungal keratitis and who are enrolled in the trial, there will be several additional baseline assessments to evaluate the impact of MK on quality of life.<br><br>Vision Related Quality of Life (VRQoL): will be assessed by a vision disease specific tool the WHO/PBD-VF20. [30] This tool measures the impact of visual impairment in the person's life including mental wellbeing, dependency and social functioning. These have been used in a number of other vision related studies to show a difference in QoL.[31,32] This instrument consists of 20 questions divided into three sub-scales: visual symptom, general functioning and psychosocial. It begins by asking the patient *"Overall, how would you rate your eyesight using both eyes?"*; and uses a five point scale answer option such as *"very good"*, *"good"*, *"moderate"*, *"bad"*, *"very bad"*. The test is scored out of 100, with higher scores reflecting a better VRQoL.<br><br>General Health Related Quality of Life (HRQoL): We will use the EQ-5D questionnaire and EQ-Visual Analogue Scale. Patients will also be assessed using the WHOQOL-BREF. This has good applicability in low and middle-income countries (LMIC) as it was developed simultaneously from concept across 18 countries in Africa, Asia and Latin America. It measures 4 domains of health: Physical Health, Psychological Health, Social Relationships, and Environment. It asks respondents 26 questions how much (frequency) they have experienced and/or were able to do things (e.g. feel safe, able to concentrate, enjoy life) in the past 4 weeks and how satisfied they are with certain aspects of their lives (e.g. sleep, capacity for work).[33] |


For peer review only - http://bmjopen.bmj.com/site/about/guidelines.xhtml

**Randomisation and Masking**

***Sequence generation***: a computer-generated randomisation list will be prepared by an independent statistician at LSHTM, who will hold the sequence, will not be masked, and will not be involved in any other aspect of the trial. The sequence is in a 1:1 allocation ratio of CHX to NATA, with a random block size (2, 4 or 6).

***Allocation concealment and implementation:*** the randomisation sequences will be concealed in sequentially numbered, opaque envelopes. The envelopes will be prepared by a person independent of all other aspects of the trial. The randomisation administrator (nurse or pharmacist) will conduct the random allocation procedure. The investigational products will be stored in the trial coordination office in a locked and separate drug cabinet dedicated for this clinical trial only. The cabinet will only be used for drug storage and will only be accessed by the randomisation administrator. The randomisation administrator will work in a separate room in the clinic. After the patient has been recruited and assessed, they will be guided to this separate room where the randomisation allocation will be conducted.

***Masking:*** The topical treatments being compared in this trial have a different appearance: the chlorhexidine 0.2% is a clear, colourless solution and the natamycin 5% is an opaque, white suspension. Therefore, it is not possible to mask the participants to this difference in visual appearance. However, the patients will not be told which treatment they have been allocated. Prior to any follow-up clinical examinations, a nurse, otherwise uninvolved in the study, will wipe away any white natamycin residue from the patient's eyes to avoid unmasking the clinical assessor. This procedure was used successfully in other trials.[6] All clinicians involved in the clinical assessment of the patients will be masked to the allocation. The statistician who will perform the primary analysis will be masked to the allocation and only receive the actual allocation code sequence from the independent statistician after the analysis code has been prepared and pre-tested with a test sequence. The primary outcome is visual acuity at 3-months assessed by an optometrist who will not have been involved in any other aspects of the trial and masked to the allocation. By three months the treatment courses are likely to be completed. There will be masked grading of the photographs to independently confirm outcome measures and assess for any systematic bias on the part of the clinical examiners.

***Unmasking:*** Unmasking is a serious action and should only be performed if necessary to ensure the safety of a study participant. Anyone unmasked to randomised treatment for the purpose of creating analyses for independent data and safety monitoring committees will not be involved in any other aspects of the conduct or final analysis of the study. A list will be maintained of all unmasked members of staff and will be approved by the Chief Investigator. Such staff members must sign a document to indicate that they are aware of their



For peer review only - http://bmjopen.bmj.com/site/about/guidelines.xhtml

responsibilities with respect to confidentiality. The processes used to provide access to unmasked treatment codes and reports relating to these codes shall be documented.

**Intervention and Treatment**

The standard of care in this hospital is for cases of fungal keratitis to be admitted. If willing, patients will be admitted for close observation and supervised treatment, until there are signs of improvement and the supervising clinician considers it safe to provide ongoing management on an outpatient basis.

***Trial Treatment Arms***

A) *Chlorhexidine 0.2% w/v eye drops*

Chlorhexidine 0.2% w/v solution, as eye drops, will be applied to the surface of the infected eye (one drop per application). The chlorhexidine 0.2% w/v eye drops used in these studies is produced by Mandeville Medicines, UK.

B) *Natamycin 5% eye drops*

Natamycin 5% w/v suspension, as eye drops, will be applied to the surface of the infected eye (one drop per application) Topical natamycin 5% is produced by FDC Pharmaceuticals, India.

***Dosing Schedule:*** Both trial treatment arms will follow the same dosing schedule. Eye drops will be given hourly day and night for 48 hours, then hourly whilst awake for five days, and then two-hourly whilst awake for two further weeks. If the ulcer has healed (epithelial defect less than 1mm, infiltrate resolved, with or without corneal scarring), then treatment is stopped. If resolving stromal infiltration and/or epithelial defect >1mm but < 5mm, treatment is reduced to four times daily. If resolving but with epithelial defect > 5mm and/or stromal infiltration / hypopyon, treatment is reduced to six times daily. Treatment duration will be tailored to clinical response, with patients reviewed regularly whilst on treatment in addition to their scheduled study visits.

***Topical treatments:*** Several other topical medicines are used in the standard care of people with corneal infections:

1. **Fluorescein sodium ophthalmic strips** (Contacare Ophthalmics and Diagnostics, India) to highlight the area of cornea epithelial defect.
2. **Anaesthetic eye drops** to anaesthetise the cornea before for procedures such as microbiology samples: Proxymetacaine 0.5% eye drop Minims (Bausch and Lomb, UK).
3. **Antibiotic eye drops** may be used as per the judgement of the treating ophthalmologist if there is a suspicion of mixed bacterial and fungal keratitis: Moxifloxacin 0.5% eye drops (Centaur Pharmaceuticals, India).


For peer review only - http://bmjopen.bmj.com/site/about/guidelines.xhtml

4. **Mydriatic eye drops** for pupil dilation to reduce discomfort from the infection: Cyclopentolate 2% eye drops (Aurolab, India), twice daily.
5. **Ocular hypotensive eye drops** if the intra-ocular pressure is elevated >25 mmHg. This will be at the discretion of the supervising clinician; usual first line treatment is timolol 0.5% eye drops (Allergan, India). Alternative treatment is betaxolol 0.5% (FDC, India).

***Ancillary treatment in patients refractory to trial medication:*** In patients with progressive fungal keratitis despite 7 days or more of trial medication, additional treatment options will be considered and offered. After repeating microbiological tests to rule out a mixed bacterial infection, deteriorating patients are started on topical voriconazole 1% hourly if the ulcer is superficial only. For ulcers that are deeper than 75% of the corneal thickness, oral ketoconazole 200mg twice-daily is added (with monitoring of liver function). The choice of ancillary treatment is due to local availability. If, following 7 days of additional treatment, despite these measures there is ongoing progression, surgical management such as a TPK can be considered.

***Non-pharmacological treatment:*** It is sometimes necessary to perform surgical procedures during the management of corneal infections. In the context of the trial this will be performed by the supervising consultant ophthalmologist. These can include:

1. Insertion of a bandage contact lens for very small perforations.
2. Tissue glue and patch for small perforations
3. Corneal transplant (TPK) for progressive fungal infections that are refractory to medical management or large perforations.
4. Conjunctival flaps to cover corneas in non-healing ulcers.

**Outcome Measures**

***Primary Outcome Measure:***

The primary outcome will be best spectacle corrected visual acuity (BSCVA) in logMAR units at three months. This will be measured by a trial-certified optometrist, independent of all other aspects of the study and masked to allocation. This has been the primary outcome of several other trials and will therefore facilitate comparison. Three months is the time at which clinical experience suggests most corneal ulcers have usually healed. BSCVA is chosen as it is easy to measure and is of functional significance. We will measure the BSCVA using an LED-backlit, Tumbling-E LogMAR chart (Good-Lite Co., IL, USA) under controlled conditions. We will also measure vision using Peek Acuity, a validated smartphone application, in situations where the patient is unable to attend the hospital for follow-up and outcome data is only available through a domiciliary visit. [34] For patients who have CF vision or less, pre-defined logMAR values will be assigned based on previous clinical trials. [35,36] Similarly, for patients who undergo a corneal transplant (therapeutic penetrating keratoplasty, TPK), a pre-defined visual acuity of 1.9 logMAR will be given, or last observation carried forward (LOCF, whichever is the better vision), as per previous studies. [6]



For peer review only - http://bmjopen.bmj.com/site/about/guidelines.xhtml

For peer review only

***Secondary Outcome Measures:***

We will be assessing a number of secondary outcome measures that relate to different measures of visual acuity such as Peek Acuity; clinical signs of healing such as reduction of epithelial defect; microbiological culture rates; and other clinical outcome measures such as scar size or perforation rate. These secondary outcomes are given in Table 3 below.

**Table 3: Secondary Outcome Measures that will be investigated as part of the trial, together with analysis details**

| Secondary outcome measure | Details |
|---|---|
| Three-week BSCVA | We will analyse the secondary outcome of three-week BSCVA in logMAR in the same manner as the primary analysis of the primary outcome described above. The three-week BSCVA will include values taken between 18 days and 5 weeks, with the value closest to 3 weeks used. |
| Presenting VA by Peek | We will analyse the presenting VA by Peek Acuity with and without pinhole at three months as a secondary outcome. This will be of interest if we are unable to get reliable BSCVA measurements at 3 months (i.e. if patients fail to attend and we need to attend their houses for visual acuity testing). This will be performed in the same way as the primary analysis of the primary outcome. We will also perform a sensitivity analysis including those lost to follow-up, by using the most recent observation of this variable. |
| Scar/infiltrate size at 1 week, 3 weeks and 3 months by slit lamp examination. | The geometric mean of the two principle axes in mm of the scar or infiltrate at 1 week, 3 weeks and 3 months will be used as a secondary outcome variable. The slit-lamp scar size will be compared at each of these time points between treatment arm in the same manner as described above. This will be by linear regression, with treatment arm and baseline infiltrate / scar size as pre-specified covariates. This controls for the baseline infiltrate / scar size. |
| Time to full epithelial healing (slit lamp examination by ophthalmic clinician) | Time of re-epithelialisation will be defined as the midpoint between the last review where an epithelial defect (ED) was present and the subsequent review where there was no ED. An area of fluorescein staining of less than 0.5mm will be considered as a resolved ED due to the difficulty in differentiating a smaller defect from a small amount of fluorescein pooling observed in a healed defect.<br><br>Analysis of time to healing will use Cox proportional hazards regression with treatment group as the primary predictor and with predictors of baseline ED size (using the geometric mean in mm as outlined above). Survival curves will be plotted using Kaplan-Meier analysis for both treatment arms up to the final visit at three months. The proportional hazards assumption will be checked by stratifying on quartiles of the baseline ED size and if the assumption does not hold, the stratified results will be the ones reported. Additionally, treatment failure (defined as persisting epithelial defect greater than 0.5mm at the three-month review) will be compared between treatment groups using Fisher's exact test. |
| Rate of healing | We will assess how quickly the area of ulceration reduces over time. The rate will be calculated between the 1 week, 3 week and 3-month review by taking the difference in ED size between the two time points, in mm, and diving by the number of days to give a rate of mm/day. Analysis will be performed using Cox regression. |
| Microbiological cure | Patients who have a persisting corneal ulcer (as defined by the presence of an ED) at day 7 will undergo a repeat corneal scrape and microbiological investigations. Microbiological cure at 7 days will be defined as the absence of any micro-organisms as no significant growth on culture. The number of patients with microbiological cure at day 7 will be compared between the two treatment arms using logistic regression with treatment group and organism (Aspergillus spp., Fusarium spp., or other) as covariates. |


For peer review only - http://bmjopen.bmj.com/site/about/guidelines.xhtml

| | |
|---|---|
| Ulcer depth at 1 week and 3 weeks (slit lamp examination by ophthalmic clinician). | The depth of ulcer in terms of percentage of healthy cornea will be compared at 1 week and 3 weeks between treatment arms, adjusting for baseline depth in the same manner with analysis performed by linear regression |
| Hypopyon height at 1 and 3 weeks, (slit lamp examination by ophthalmic clinician) | The hypopyon height in mm will be compared at 1 week and 3 weeks between treatment arms, adjusting for baseline hypopyon height in the same manner with analysis performed in the same way (linear regression) as outlined in 6.3 above |
| Perforation and/or TPK and/or conjunctival advancement by three months (slit lamp examination by ophthalmic clinician) | The number of patients who undergo perforation and/or require TPK and/or have undergone conjunctival advancement by three months will be reported using confidence intervals and descriptive statistics. The study is not powered to detect a difference in perforation rate or TPK between treatment groups. Therefore not reporting a significant difference may be wrongly interpreted as there being no difference between groups. However, exploratory analysis will be performed to compare TPK or perforation rate between treatment groups. This will be by logistic regression to compute an odds ratio by arm. However, this analysis will be exploratory to find risk factors for perforation and/or transplantation. |
| Loss of Eye | The number of patients who have their eye surgically removed (evisceration or enucleation) during the 3 month follow-up period will be reported using confidence intervals and descriptive statistics in the same way as for TPK / perforation rate above, along with exploratory analysis using logistic regression to find risk factors for eye loss and odds ratio for this by arm. |
| Ocular adverse effects, slit lamp examination by ophthalmic clinician. | The proportion of patients with one or more adverse events will be compared using Fisher's Exact test. Additional analysis to compare the rate of adverse events during the three month follow-up will be by Poisson regression as this can take into account multiple instances within one participant. |
| Quality of life assessed using: EQ-5D, WHO/PBD-VF20, WHOQOL-BREF | QoL can be assessed quantitatively using different tools depending on what is of interest. For example, disease-related QoL can be assessed (e.g. vision related QoL, VRQoL) or more general health-related issues irrespective of the disease can be investigated (health-related QoL, HRQoL).[37]<br><br>We will use the WHO/PBD-VF20 (World Health Organisation/ Prevention of Blindness and Deafness—Visual Functioning 20 item questionnaire) VRQoL tool. This tool measures the impact of visual impairment in the person's life including mental wellbeing, dependency and social functioning. These have been used in a number of other visual related studies to show a difference in QoL. [31,32]<br><br>For HRQoL, we will use the EQ-5D questionnaire, EQ-Visual Analogue Scale and the WHOQOL-BREF. The EQ-5D is a standardised tool to measure health outcomes.[33] The WHOQOL-BREF has good applicability in (LMIC as it was developed simultaneously from concept across 18 countries in Africa, Asia and Latin America. It measures 4 domains of health: Physical Health, Psychological Health, Social Relationships, and Environment.<br><br>Analysis will be by comparing the scores obtained for each QoL assessment for the two treatment arms to estimate the effect of CHX and NATA on patients' quality of life. We propose to perform linear regression |
| Cost effectiveness analysis, using EQ-5D data form 3 months and direct cost data | Mean direct costs incurred by patients will be compared between interventional arms using the t-test for significance. The difference from the baseline EQ-5D and the three month EQ-5D mean scores will also be compared in a similar fashion. |
| Drug adherence | The rate of drug adherence will be compared between the two treatment groups using descriptive statistics. |

**Outcome Assessments**

Participants will be re-assessed at 2-days, 1-week, 2-weeks, 3-weeks, 2-months and 3-months following enrolment. Additional examinations, outside the trial protocol schedule may be conducted by the supervising clinician as indicated. The specific assessments to be carried out at each visit are indicated in Table 4. These


For peer review only - http://bmjopen.bmj.com/site/about/guidelines.xhtml

will be conducted in the same way described for the baseline assessment (Table 2). At each follow-up assessment the participant will be asked about adherence to treatment and symptoms, including side effects, if still receiving trial treatment. To monitor trial medication adherence, the study participants will be asked to bring their eye drop bottles to the 1, 2- and 3-week follow-ups. The amount of remaining medication will be measured by weighing the bottle and will be compared with the anticipated remaining amount provided the drops were being used as instructed. Participants will be resupplied with medication as needed. The presenting visual acuity will be measured on each visit. In addition, the BSCVA will also be measured at the three-month follow-up (primary outcome measure). At the final 3-month follow-up the three quality of life questionnaires will be repeated. On each occasion the eyes will be examined using a slit-lamp and the cornea photographed. The *in vivo* confocal microscopy will be repeated at 1, 2 and 3 weeks to determine whether there is evidence of fungal hyphae resolution with the fragmentation of linear elements. At the 1-week follow-up if the ulcer has not healed it will be re-scraped for repeat culture to determine whether the keratitis is now culture-positive or culture-negative. We will give the study participants appointment cards for the next follow-up and they will be reminded about the follow-up a week prior to the date. Public transport costs will be paid for participants who are outpatients.

**Table 4: Baseline and follow-up assessment components.**

| Assessment Item | Baseline | Day 2 | Day 7 | Day 14 | Day 21 | Day 60 | Day 90 |
|---|---|---|---|---|---|---|---|
| History / Baseline questionnaire | X | | | | | | |
| Check treatment adherence | | X | X | X | X | X | X |
| Check for side effects | | X | X | X | X | X | X |
| Visual Acuity – Presenting | X | X | X | X | X | X | X |
| Visual Acuity – BSCVA | X | | | | | | X |
| Contrast Sensitivity | X | | | | | | X |
| Slit-lamp Examination | X | X | X | X | X | X | X |
| Cornea Photography | X | X | X | X | X | X | X |
| *In vivo* confocal microscopy | X | | X | X | X | | |
| Cornea samples (Microbiology/PCR) | X | | X | | | | |
| Quality of Life Tools | X | | | | | | X |

**Treatment Review**

At each follow-up visit participants will be reviewed by an ophthalmic clinician experienced in the management of microbial keratitis. Clinical responses to anti fungal therapy tend to be relatively slow (compared to bacterial infections). Prolonged topical treatment courses of 4-6 weeks are usually needed. Therefore, a change in therapy is usually not made for at least the first week. Other interventions may be indicated such as application of glue to corneal perforations, conjunctival flaps or therapeutic penetrating keratoplasty (corneal transplant).


For peer review only - http://bmjopen.bmj.com/site/about/guidelines.xhtml

**Stopping Rules**

If the study eye develops any serious adverse outcomes, the antifungal study medication may be discontinued if it is considered to be responsible for the adverse event. The patient will then be treated at the discretion of the supervising ophthalmologist, without breaking the randomisation code. If the medication is stopped, the patient will continue with the scheduled follow-up examinations.

**Loss to Follow-up**

Loss to follow-up rates are expected to be low, based on clinical experience. Participants who do not present for their follow-up visit will be contacted by telephone. Reasons for the loss to follow-up will be identified. Patients will be counselled about the importance of attending for ongoing treatment and monitoring. If they are unwell or unable to attend the hospital for some specific reason the study team will arrange to visit them in their home. Reasons for loss to follow-up will be recorded and reported.

**Data collection, management, confidentiality and access to data**

Data will be collected using paper Clinical Record Forms (CRFs). These will be stored securely at SCEH and scanned electronic copies taken at the end of the day, stored on an encrypted drive with encrypted backups made daily both on and off-site. These data will be double entered into two separate MS Access databases. After double data entry has been completed data will be cleaned using EpiData version 3.1 software. Data entry is supervised by the local study co-ordinator on a daily basis, with data collection and data entry progress being reviewed by the study co-ordinator and chief investigator at LSHTM on a weekly basis. Data protection and confidentiality is maintained through restricted access to the database system and cupboard where the paper documents are kept. Only authorised users will have access to the locked filing cabinet. The database will be password protected, with each data entry staff member having their own password. Data exports for further analysis will be anonymized.

**Data and Safety Monitoring Board**

The Data and Safety Monitoring Board (DSMB) for this trial includes independent experts in bioethics, biostatistics, epidemiology and ophthalmology appointed by the Trial Steering Committee and approved by the national regulatory authorities, before the start of the study. The DSMB meets at least twice each year and organises teleconferences as needed for progress reporting. The study protocol and modifications are subject to review and approval by ethics committees in Nepal and LSHTM, and by the DSMB. The DSMB will monitor any severe or unexpected events and oversees the data collected. The DSMB will be responsible for reviewing the results of the interim analysis and determining whether or not the trial should continue, with or without modifications.

**Monitoring for Harm**


For peer review only - http://bmjopen.bmj.com/site/about/guidelines.xhtml

Patients will be monitored at each visit for adverse events or reactions. We will follow standardised definitions for adverse events, adverse reactions, unexpected adverse reactions, serious adverse events or reactions, and suspected unexpected serious adverse reaction. These, along with the reporting scheme, are given in Appendix 2.

**Sample Size Considerations**

The study is powered to test the hypothesis that CHX is non-inferior to NATA in terms of the primary outcome (BSCVA at 3 months) at a pre-specified non-inferiority margin (Delta) of 0.15 logMAR units. It is possible that CHX is non-inferior to NATA, given pilot RCT data that showed no statistically significant difference between CHX and NATA, with a Cochrane review finding a non-significant trend favouring CHX over NATA.[10,18,19] Despite this trend favouring CHX, we have chosen to carry out a non-inferiority trial rather than a superiority trial as this is a more clinically pragmatic approach. It would be counter-productive to conduct a superiority trial and find no statistically significant difference between the two treatments leading clinicians to potentially disregard CHX as a treatment, when in fact it may be 'non-inferior" to NATA, and be the only available or most cost-effective treatment.

The choice of Delta: This clinically meaningful difference of 0.15 logMAR was chosen as a difference of 0.15 logMAR corresponds to approximately 1.5 lines on a Snellen chart; any difference greater than this is clinically significant, as a difference of less than 0.15 log MAR could potentially be accounted for by testing / re-testing error.[34] Furthermore, it was used in the MUTT1 trial, providing methodological consistency between studies.[6] In addition, previous studies have suggested treated ulcers improve at a mean of four Snellen lines from baseline.

Sample size was calculated for 90% power and adjusted final alpha of 0.0492, taking account of a single interim analysis using O'Brien-Fleming approach to maintain type-1 error rate of 5%. A sample size of 452 is required to detect a non-inferiority margin of 0.15-logMAR in BSCVA 3-months after enrolment between arms, assuming 0.5 SD for 3-month BSCVA and 15% dropout. However, given approximately 10% of infections are mixed and these will be excluded from the primary analysis, 500 patients will be recruited. This sample size provides 90% power to detect superiority in BSCVA at 3-months if there is ≥0.17 LogMAR units difference as a secondary analysis.

**Analysis Plan**

The analysis will be by intention to treat. All patient data will be analysed according to their randomisation allocation irrespective of whether or not the patient received or adhered to the allocated treatment. CONSORT guidelines for analysing/reporting non-inferiority RCTs will be followed including a flowchart


For peer review only - http://bmjopen.bmj.com/site/about/guidelines.xhtml

showing cases assessed, recruited and followed-up by arm will be prepared.[38] Baseline characteristics will be summarised by arm.

***Primary Outcome Analysis – unadjusted analysis:*** The primary analysis will be conducted using all available data, missing data due to loss to follow up will be excluded. The primary analysis of the primary outcome (BSCVA at 3 month) will be by linear regression, with treatment arm and baseline BSCVA as pre-specified covariates. This controls for the baseline BSCVA. The treatment group is the primary predictor. Primary analysis will exclude mixed fungal and bacterial infection (isolated in the baseline sample).

We will use our alpha of 0.0492 to test the null hypotheses at 0.0492 significance. The null hypothesis for non-inferiority is that the mean BSCVA at three months for chlorhexidine is greater than or equal to 0.15 logMAR worse than the BSCVA when natamycin is used. Significance will be assessed using a two-tailed test at 0.0492 level for assessing non-inferiority. CHX would be found non-inferior if the upper one-sided 95% confidence level for this regression coefficient (i.e. the effect of CHX controlling for baseline BSCVA) exceeds 0.15 logMAR.

***Primary Outcome Analysis – adjusted analysis:*** In the event that there is a baseline imbalance between the treatment groups in a baseline covariate due to chance, we will perform an adjusted (sensitivity) analysis (see below). This is particularly important if CHX has a better outcome than NATA, as the adjusted treatment effects may account for this observed imbalance whilst the unadjusted analyses may not. Sensitivity analyses will allow us to show that any observed positive treatment effect is not solely explained by imbalances at baseline in any of the covariates.

***Secondary Analyses of the Primary Outcome***

<u>Per-protocol analysis</u>

Repeat analysis of the primary outcome will be done as per the protocol, based on what the participants actually took. The per-protocol population would include all the individuals included in the primary intention-to-treat (ITT) analysis, excluding individuals who showed poor compliance with the medications (defined as taking less than 50% through self-reporting or bottle weighing, whichever is the lower); individuals where there have been major protocol deviations; and non-fungal or mixed corneal infections (should these patients happened to have been randomised). All analyses that are performed as ITT will be repeated as per-protocol and labelled as such.

For peer review only

Mixed infections

Secondary analysis of the primary outcome (by intention to treat) will include mixed infections and will be carried out in the same way as in primary analysis of the primary outcome above.

Sensitivity analyses

For the primary analysis those individuals with missing outcome data (i.e. lost to follow up) will be excluded. Sensitivity analyses will be performed by imputing a range of scenarios to demonstrate a range of potential results, where there is missing outcome data. In the case of substantial missing data in the trial, the primary analysis will be carried out as previously stated excluding missing observations. This however assumes data are missing completely at random. As a sensitivity analysis, we will then apply a multiple imputation approach to the missing data, if we consider the data is randomly missing conditional on other observed covariates.

In the case that there is a systematic (i.e. non-random) reason for a difference in the follow-up rates between the two groups, we will explore models in which these missing outcome data are assumed to be non-random i.e. dependent on the outcome being regressed, BSCVA or treatment group.

We will also perform sensitivity analyses on patients whose vision is CF or worse, those patients who have undergone a TPK or those with corneal perforation, to see if there is any change in the effect size or conclusions drawn.

Analysis of Other Potential Determinants for Success

Logistic regression random effects models will be used to analyse potential factors that may be associated with a poor primary outcome, BSCVA at 3 months, defined as >1.0 logMAR. Individual baseline characteristics will be used separately as an exposure variable with BSCVA at 3 months as the outcome, with the model adjusted for trial arm. A multivariate model will be built using parameters with a p-value of $< 0.2$ in the log likelihood ratio test. Variables will be removed one by one, by omitting the variable with the largest p-value each time, until all predictors in the model have a $p<0.05$.

***Secondary Outcome Analysis:*** The secondary outcomes outlined above will be analysed by arm. Additional adjustment for factors imbalanced between arms at baseline will be introduced as appropriate. Continuous outcomes will be analysed using linear regression. Binary and ordinal outcomes will analysed using logistic regression. Details of these are given in Table 3.

***Interim Analysis:*** An interim analysis will be conducted for the DSMB by an independent statistician after 1/3 of patients recruited and completed follow-up.



For peer review only - http://bmjopen.bmj.com/site/about/guidelines.xhtml

**Patient and public involvement**

Mixed methods descriptive cross-sectional studies with semi-structured interviews and focus group discussions with patients and eye health providers were carried out in Sagarmatha zone, Eastern Nepal at various points in 2018.[39] These conversations highlighted the delayed presentation often seen with microbial keratitis combined with the often prohibitively high costs of treatment. Furthermore, treatment is not always felt to be effective. Eye health workers were keen to receive further training and highlighted the need for greater government support in the provision of eye care services in the community.

**ETHICS AND DISSEMINATION**

Ethics committee and regulatory review and approval has been obtained from the Nepal Health Research Council (NHRC) Ethics Committee, Kathmandu, Nepal; the Department of Drug Administration (DDA), Kathmandu, Nepal; and the London School of Hygiene and Tropical Medicine Ethics Committee, UK. The trial is registered with the ISRCTN clinical trials registry (ISRCTN14332621). Protocol modifications are submitted to the relevant parties for review and/or approval. Table 5 summarises the study protocol and trial registration information. At the end of the study period, patients who still require treatment or follow-up will continue to be treated at SCEH as per routine clinical practice. The trial Sponsor is the London School of Hygiene and Tropical Medicine. The results of this trial will be presented at local and international meetings and submitted to peer-reviewed journals for publication.

For peer review only



For peer review only - http://bmjopen.bmj.com/site/about/guidelines.xhtml

**Table 5: Registration Data and Protocol Summary**

| Data category | Information |
|---|---|
| Primary registry and trial identifying number | ISRCTN Registry; ISRCTN14332621 |
| Date of registration in primary registry | 15 May 2019 |
| Secondary identifying numbers | |
| Source(s) of monetary or material support | Wellcome Trust |
| Primary sponsor | London School of Hygiene and Tropical Medicine (LSHTM) |
| Secondary sponsor(s) | |
| Contact for queries | Jeremy Hoffman FRCOphth (Jeremy.hoffman@lshtm.ac.uk) |
| Title | Chlorhexidine 0.2% vs Natamycin 5% for the treatment of fungal corneal infections |
| Countries of recruitment | Nepal |
| Health condition(s) or problem(s) studied | Fungal keratitis |
| Intervention(s) | Participants will be randomised to either topical chlorhexidine 0.2% or topical natamycin 5% |
| Key eligibility criteria | 1. Acute MK characterised by:<br>• Corneal epithelial ulceration >1mm diameter<br>• Corneal stromal infiltrate<br>• Acute inflammation: e.g. conjunctival injection, anterior chamber inflammatory cells, hypopyon.<br>2. Filamentous fungal hyphae visualised on smear microscopy and/or *in vivo* confocal microscopy.<br>3. Agree to be randomised to either treatment arm and able to give informed consent<br>4. Agree to be followed up at 2 days, 1 week, 2 weeks, 3 weeks, 2 months and 3 months<br>5. Adults (18 years and older) |
| Study type | Randomised controlled trial |
| Date of first enrolment | 1 June 2019 |
| Target sample size | 500 |
| Recruitment status | Recruiting |
| Primary outcome(s) | Best Spectacle Corrected Visual Acuity (BSCVA) at 3 months by a trial certified optometrist |
| Key secondary outcomes | 1. Time to full epithelial healing (slit lamp examination by ophthalmic clinician).<br>2. Pin-hole visual acuity in logMAR at 3 months, trial-certified optometrist<br>3. Scar/infiltrate size at 1 week, 3 weeks and 3 months (slit lamp examination by ophthalmic clinician).<br>4. Ulcer depth at 1 week and 3 weeks (slit lamp examination by ophthalmic clinician).<br>5. Hypopyon height at 1 and 3 weeks, (slit lamp examination by ophthalmic clinician).<br>6. Perforation and/or TPK by three months (slit lamp examination by ophthalmic clinician).<br>7. Positive culture rate at 1 week<br>8. Ocular adverse effects at each follow up visit (Day 2, Day 7, Day 14, 3 weeks, 2 months, 3 months), slit lamp examination by ophthalmic clinician.<br>9. Quality of life (QoL) assessed using: EQ-5D, WHO/PBD-VF20, WHOQOL-BREF (comparison between baseline and QoL measures at 3 months)<br>10. Cost-effectiveness analysis, using EQ-5D data form 3 months and direct cost data.<br>11. Drug adherence at each follow up visit (Day 2, Day 7, Day 14, 3 weeks, 2 months, 3 months) whilst the patient is using study medications. |


For peer review only - http://bmjopen.bmj.com/site/about/guidelines.xhtml

## CONCLUSION

This is the first large-scale randomised controlled clinical trial comparing topical chlorhexidine 0.2% to natamycin 5% for the treatment of fungal keratitis. It benefits from a pragmatic design: as a non-inferiority designed trial, if chlorhexidine is found to be within a pre-defined non-inferiority margin of 0.15 logMAR of natamycin at 3 months a recommendation to use chlorhexidine 0.2% can be made. This is a far cheaper, easy to formulate medication that could significantly increase access to anti fungal treatment for the population most in need.

## ACKNOWLEDGEMENTS

The authors would like to thank the Eastern Region Eye Care Programme (EREC-P), Nepal Netra Jyoti Sangh (NNJS) and the Nepal Health Research Council (NHRC) for helping with study co-ordination and implementation. The authors would like to thank the staff and management board at Sagarmatha Choudhary Eye Hospital (SCEH) for their continued support, co-ordination and implementation of the study. The authors are grateful to the guidance of the Data Safety Monitoring Board including Reeta Gurung (chair), Salma KC Rai, Sabina Shrestha and Meenu Chaudhary.

For peer review only

## COLLABORATORS

SCEH:

Abhishek Roshan (Hospital Manager); Sanjay Kumar Singh (Medical Superintendent); Reena Yadav (Primary Investigator); Sandip Das Sanyam (Study Co-Ordinator); Pankaj Chaudhary (Microbiologist); Rabi Shankar Sah, Kamlesh Yadav (Investigators); Ram Narayan Bhandari, Aasha Chaudhary, Sharban Mandal (Eye Health Workers); Raja Ram Mahato (Randomisation Administrator and Logistics); Lalita Rajbanshi (Laboratory Assistant); Ramesh Sah, Arvind Ray, Sachindra Kamti (Optometrists); Avinash Chaudhary (Ophthalmic Assistant); Rabindra Lal Chaudhary (Hospital Chairman); Suresh Singh, Ravi Pant, Rakesh Singh (Hospital Management); Ram Kumar Jha (Ophthalmic Assistant, Rajbiraj ECC)

NNJS:

Sailesh Kumar Mishra (Executive Director); Sabita KC (Board Secretary); Ranjan Shah (Programme Associate); Jaganath Dhital (Assistant)

EREC-P:

Sanjay Kumar Singh (Programme Director)

JEH:

Hemchandra Jha (Medical Superintendent); Mahesh Yadav (Investigator); Rudal Prasad Sah (Ophthalmic Assistant)

LSHTM:

Jeremy Hoffman (Primary Investigator); Matthew Burton (Chief Investigator); Astrid Leck (Microbiologist); David Macleod, Helen Weiss (Statisticians); Victor Hu (Investigator); Sarah O'Regan (Administrator)



For peer review only - http://bmjopen.bmj.com/site/about/guidelines.xhtml

**FUNDING STATEMENT**

This research was funded through a Senior Research Fellowship to MJB from the Wellcome Trust (207472/Z/17/Z). The funders had no role in study design, data collection and analysis, decision to publish, or preparation of the manuscript.

**COMPETING INTERESTS STATEMENT**

All authors declare there are no conflict of interests.

**DATA STATEMENT**

The datasets generated during and/or analysed during the current study will be available upon request from Prof Matthew Burton (matthew.burton@lshtm.ac.uk). The full data set will be available with all patient identifiable details removed. Data will be available after formal reporting of the study findings in a peer-reviewed scientific publication. Datasets will only be available to bona fide scientific investigators. Requests should be made to the Chief Investigator in writing detailing the scientific investigators background and intended use for the data. Consideration will be given to all proposed analyses, with likely envisaged uses including investigators planning on conducting meta-analyses for example. Patient Information Sheets and consent forms specifically referenced making anonymised data available and this has been approved by the relevant ethic committees.

**AUTHOR CONTRIBUTIONS**

Searched the literature: JJH, MJB

Drafted initial protocol: JJH

Contributed to protocol development and revision: JJH, RY, SD, PC, AR, SKS, SA, EM, DM, HAW, AL, VHH, MJB

Drafted this manuscript: JJH

Critically revised this manuscript: JJH, RY, SD, PC, AR, SKS, SA, EM, DM, HAW, AL, VHH, MJB

Conceptualization: MJB

Funding acquisition: MJB

For peer review only

**APPENDICES:**

Appendix 1 - Informed consent material

Appendix 2 – Monitoring for Harm

Appendix 3 - Biological specimens

Appendix 4 – SPIRIT Checklist

For peer review only



For peer review only - http://bmjopen.bmj.com/site/about/guidelines.xhtml

## LEGENDS FOR FIGURES

**Figure 1: Fungal keratitis and corneal scarring.** (A) Active fungal keratitis with signs of acute inflammation and corneal ulceration. Photograph taken at presentation to SCEH. (B) Corneal Scar, the blinding sequela of a resolved episode of microbial keratitis. Photograph taken at two months following presentation (same patient as (A)).

**Figure 2: Progressive fungal keratitis.** (A) Early filamentous fungal keratitis; started immediately on intensive topical antifungal treatment (Natamycin 5%). (B) The same case one week later, unresponsive to intense Natamycin 5% treatment, with progression of the infection.

**Figure 3: Overview of the clinical trial.** Microbial keratitis is defined as presence of corneal epithelial ulceration (>1mm in diameter), corneal stromal infiltrate and signs of acute inflammation (e.g. conjunctival injection, anterior chamber inflammatory cells, hypopyon). Fungal elements to be detected by smear microscopy and/or confocal microscopy. Those eligible will be randomised 1:1 to CHX or NATA (n = 500). BSCVA: Best Spectacle Corrected Visual Acuity; TPK: Therapeutic Penetrating Keratoplasty.

For peer review only

## REFERENCES


1 Burton MJ, Pithuwa J, Okello E, *et al.* Microbial Keratitis in East Africa: Why are the Outcomes so Poor? *Ophthalmic Epidemiol* 2011;**18**:158–63. doi:10.3109/09286586.2011.595041

2 Thomas PA, Leck AK, Myatt M. Characteristic clinical features as an aid to the diagnosis of suppurative keratitis caused by filamentous fungi. *British Journal of Ophthalmology* 2005;**89**:1554–8. doi:10.1136/bjo.2005.076315

3 Leck AK, Thomas PA, Hagan M, *et al.* Aetiology of suppurative corneal ulcers in Ghana and south India, and epidemiology of fungal keratitis. *British Journal of Ophthalmology* 2002;**86**:1211–5. doi:10.1136/bjo.86.11.1211

4 Bongomin F, Gago S, Oladele RO, *et al.* Global and Multi-National Prevalence of Fungal Diseases-Estimate Precision. *J Fungi (Basel)* 2017;**3**:57. doi:10.3390/jof3040057

5 Ali Shah SI, Shah SA, Rai P, *et al.* Visual outcome in patients of keratomycosis, at a tertiary care centre in Larkana, Pakistan. *J Pak Med Assoc* 2017;**67**:1035–8.

6 Prajna NV, Krishnan T, Mascarenhas J, *et al.* The mycotic ulcer treatment trial: a randomized trial comparing natamycin vs voriconazole. *JAMA Ophthalmol* 2013;**131**:422–9.

7 Prajna NV, Krishnan T, Rajaraman R, *et al.* Effect of Oral Voriconazole on Fungal Keratitis in the Mycotic Ulcer Treatment Trial II (MUTT II): A Randomized Clinical Trial. *JAMA Ophthalmol* 2016;**134**:1365–72. doi:10.1001/jamaophthalmol.2016.4096

8 Galarreta DJ, Tuft SJ, Ramsay A, *et al.* Fungal keratitis in London: microbiological and clinical evaluation. *Cornea* 2007;**26**:1082–6. doi:10.1097/ICO.0b013e318142bff3

9 Ong HS, Fung SSM, Macleod D, *et al.* Altered Patterns of Fungal Keratitis at a London Ophthalmic Referral Hospital: An Eight-Year Retrospective Observational Study. *Am J Ophthalmol* 2016;**168**:227–36. doi:10.1016/j.ajo.2016.05.021

10 FlorCruz NV, Evans JR. Medical interventions for fungal keratitis. *Cochrane Database Syst Rev* 2015;**4**:CD004241. doi:10.1002/14651858.CD004241.pub4

11 Schein OD. Evidence-Based Treatment of Fungal Keratitis. *JAMA Ophthalmol* 2016;**134**:1372–3. doi:10.1001/jamaophthalmol.2016.4167

12 Arora R, Gupta D, Goyal J, *et al.* Voriconazole versus natamycin as primary treatment in fungal corneal ulcers. *Clin Experiment Ophthalmol* 2011;**39**:434–40. doi:10.1111/j.1442-9071.2010.02473.x

13 Prajna NV, Mascarenhas J, Krishnan T, *et al.* Comparison of natamycin and voriconazole for the treatment of fungal keratitis. *Arch Ophthalmol* 2010;**128**:672–8. doi:10.1001/archophthalmol.2010.102

14 Sharma S, Das S, Virdi A, *et al.* Re-appraisal of topical 1% voriconazole and 5% natamycin in the treatment of fungal keratitis in a randomised trial. *Br J Ophthalmol* 2015;**99**:1190–5. doi:10.1136/bjophthalmol-2014-306485

15 McDonnell G, Russell AD. Antiseptics and disinfectants: activity, action, and resistance. *Clinical Microbiology Reviews* 1999;**12**:147–79.


For peer review only



For peer review only - http://bmjopen.bmj.com/site/about/guidelines.xhtml

16 Shariff JA, Lee KC, Leyton A, *et al.* Neonatal mortality and topical application of chlorhexidine on umbilical cord stump: a meta-analysis of randomized control trials. *Public Health* 2016;**139**:27–35. doi:10.1016/j.puhe.2016.05.006

17 Zhou J, Hu B, Liu Y, *et al.* The efficacy of intra-alveolar 0.2% chlorhexidine gel on alveolar osteitis: a meta-analysis. *Oral Dis* 2017;**23**:598–608. doi:10.1111/odi.12553

18 Rahman MR, Johnson GJ, Husain R, *et al.* Randomised trial of 0.2% chlorhexidine gluconate and 2.5% natamycin for fungal keratitis in Bangladesh. *British Journal of Ophthalmology* 1998;**82**:919–25. doi:10.1136/bjo.82.8.919

19 Rahman MR, Minassian DC, Srinivasan M, *et al.* Trial of chlorhexidine gluconate for fungal corneal ulcers. *Ophthalmic Epidemiol* 1997;**4**:141–9. doi:10.3109/09286589709115721

20 Dart JKG, Saw VPJ, Kilvington S. Acanthamoeba keratitis: diagnosis and treatment update 2009. *Am J Ophthalmol* 2009;**148**:487–499.e2. doi:10.1016/j.ajo.2009.06.009

21 Kosrirukvongs P, Wanachiwanawin D, Visvesvara GS. Treatment of acanthamoeba keratitis with chlorhexidine. *Ophthalmology* 1999;**106**:798–802. doi:10.1016/S0161-6420(99)90169-0

22 Seal D, Hay J, Kirkness C, *et al.* Successful medical therapy of Acanthamoeba keratitis with topical chlorhexidine and propamidine. *Eye* 1996;**10 ( Pt 4)**:413–21. doi:10.1038/eye.1996.92

23 Martin MJ, Rahman MR, Johnson GJ, *et al.* Mycotic keratitis: susceptibility to antiseptic agents. *Int Ophthalmol* 1995;**19**:299–302.

24 Geffen N, Norman G, Kheradiya NS, *et al.* Chlorhexidine gluconate 0.02% as adjunct to primary treatment for corneal bacterial ulcers. *Isr Med Assoc J* 2009;**11**:664–8.

25 Upadhyay MP, Karmacharya PC, Koirala S, *et al.* The Bhaktapur eye study: ocular trauma and antibiotic prophylaxis for the prevention of corneal ulceration in Nepal. *British Journal of Ophthalmology* 2001;**85**:388–92.

26 Habtamu E, Bastawrous A, Bolster NM, *et al.* Development and Validation of a Smartphone-based Contrast Sensitivity Test. *Transl Vis Sci Technol* 2019;**8**:13. doi:10.1167/tvst.8.5.13

27 Chidambaram JD, Prajna NV, Larke N, *et al.* In vivo confocal microscopy appearance of Fusarium and Aspergillus species in fungal keratitis. *Br J Ophthalmol* 2017;**101**:1119–23. doi:10.1136/bjophthalmol-2016-309656

28 Chidambaram JD, Prajna NV, Larke NL, *et al.* Prospective Study of the Diagnostic Accuracy of the In Vivo Laser Scanning Confocal Microscope for Severe Microbial Keratitis. *Ophthalmology* 2016;**123**:2285–93. doi:10.1016/j.ophtha.2016.07.009

29 Somannavar S, Ganesan A, Deepa M, *et al.* Random capillary blood glucose cut points for diabetes and pre-diabetes derived from community-based opportunistic screening in India. *Diabetes Care* 2009;**32**:641–3. doi:10.2337/dc08-0403

30 World Health Organization. Prevention of Blindness & Deafness. Consultation on Development of Standards for Characterization of Vision Loss and Visual Functioning. In: *WHO / PBL*. Geneva:  2003.

31 Polack S, Kuper H, Mathenge W, *et al.* Cataract visual impairment and quality of life in a Kenyan population. *British Journal of Ophthalmology* 2007;**91**:927–32. doi:10.1136/bjo.2006.110973

For peer review only

32 Polack S, Kuper H, Wadud Z, *et al.* Quality of life and visual impairment from cataract in Satkhira district, Bangladesh. *Br J Ophthalmol* 2008;**92**:1026–30. doi:10.1136/bjo.2007.134791

33 EuroQol Research Foundation. EQ-5D-3L User Guide. 2018. https://euroqol.org/publications/user-guides

34 Bastawrous A, Rono HK, Livingstone IAT, *et al.* Development and Validation of a Smartphone-Based Visual Acuity Test (Peek Acuity) for Clinical Practice and Community-Based Fieldwork. *JAMA Ophthalmol* 2015;**133**:930–8. doi:10.1001/jamaophthalmol.2015.1468

35 Barron BA, Gee L, Hauck WW, *et al.* Herpetic Eye Disease Study. A controlled trial of oral acyclovir for herpes simplex stromal keratitis. *Ophthalmology* 1994;**101**:1871–82.

36 Wilhelmus KR, Beck RW, Moke PS, *et al.* Acyclovir for the prevention of recurrent herpes simplex virus eye disease. Herpetic Eye Disease Study Group. *N Engl J Med* 1998;**339**:300–6. doi:10.1056/NEJM199807303390503

37 Habtamu E, Wondie T, Aweke S, *et al.* The Impact of Trachomatous Trichiasis on Quality of Life: A Case Control Study. *PLoS Negl Trop Dis* 2015;**9**:e0004254. doi:10.1371/journal.pntd.0004254

38 Schulz KF, Altman DG, Moher D, *et al.* CONSORT 2010 statement: updated guidelines for reporting parallel group randomised trials. BMJ. 2010;**340**:c332–2. doi:10.1136/bmj.c332

39 Burn H, Puri L, Roshan A, *et al.* Primary Eye Care in Eastern Nepal. *Ophthalmic Epidemiol* 2019;**22**:1–12. doi:10.1080/09286586.2019.1702217

For peer review only

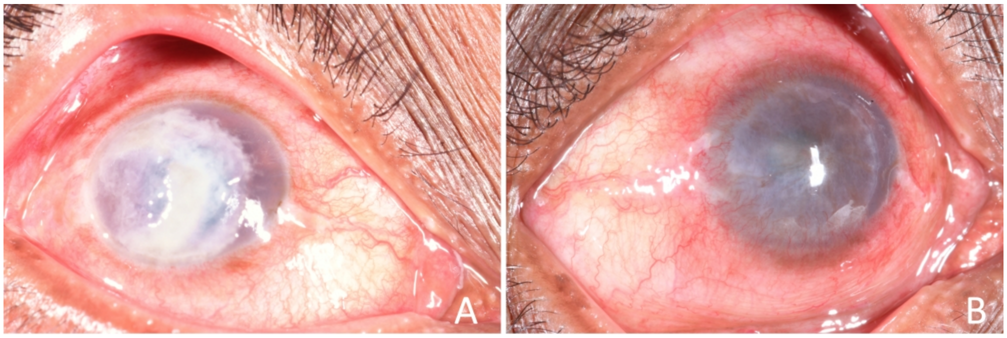


Figure 1: Fungal keratitis and corneal scarring. (A) Active fungal keratitis with signs of acute inflammation and corneal ulceration. Photograph taken at presentation to SCEH. (B) Corneal Scar, the blinding sequela of a resolved episode of microbial keratitis. Photograph taken at two months following presentation (same patient as (A)).

325x107mm (300 x 300 DPI)

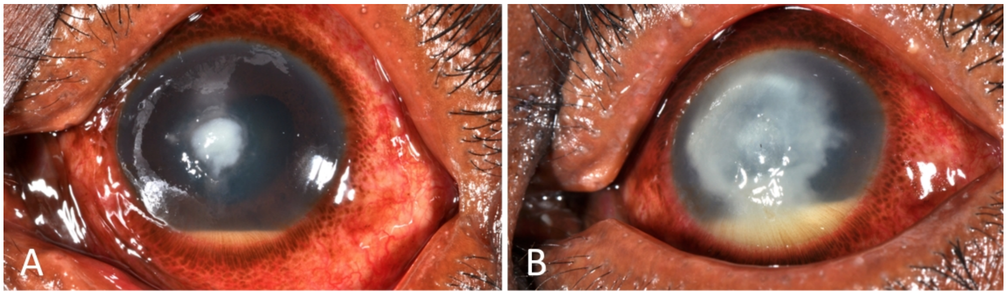


Figure 2: Progressive fungal keratitis. (A) Early filamentous fungal keratitis; started immediately on intensive topical antifungal treatment (Natamycin 5%). (B) The same case one week later, unresponsive to intense Natamycin 5% treatment, with progression of the infection.

325x93mm (300 x 300 DPI)

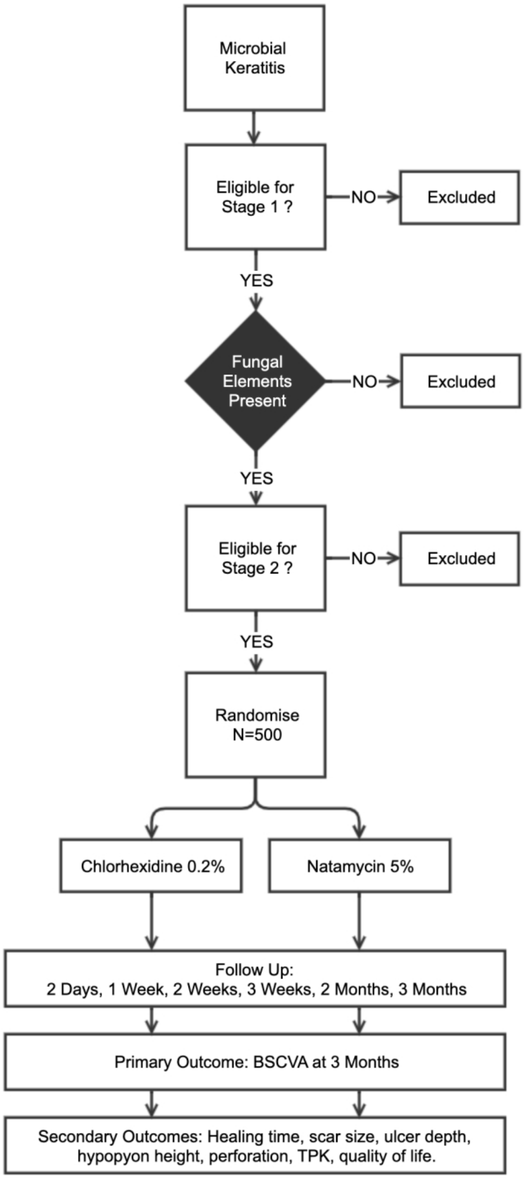


Figure 3: Overview of the clinical trial. Microbial keratitis is defined as presence of corneal epithelial ulceration (>1mm in diameter), corneal stromal infiltrate and signs ofacute inflammation (e.g. conjunctival injection, anterior chamberinflammatory cells, hypopyon). Fungal elements to be detected by smear microscopy and/or confocal microscopy. Those eligible will be randomised 1:1 to CHX or NATA (n = 500). BSCVA: Best Spectacle Corrected Visual Acuity; TPK: Therapeutic Penetrating Keratoplasty.

87x182mm (300 x 300 DPI)

# Appendix 1: Informed Consent Material

## Study Title: Randomised controlled trial of topical chlorhexidine 0.2% versus topical natamycin 5% for fungal keratitis in Nepal

*Participant Information Sheet and Consent Form - No 1:*
*For baseline assessment and investigations*

**Introduction**
You are being invited to take part in a medical research study. Before you decide it is important for you to understand why the research is being done and what it will involve. Please take time to read or listen to the following information carefully and to talk to others about the study, if you wish. Ask us if there is anything that is not clear or if you would like more information. Do not sign the consent form unless you are satisfied with the answers to your questions and decide that you want to be part of this study. Take time to decide whether or not you wish to take part.

**Why have I been invited?**
You have been invited to take part because you have a corneal infection. To guide the treatment of this it is helpful to do tests to find out the cause, to guide the choice of eye drop treatment. We are asking for your permission to carry out some tests that are listed below to find out what is causing the infection and to decide if you may be suitable for a trial comparing treatments for fungal eye infections.

**What is this study about?**
Infection of the clear part of the front of the eye (the cornea) is a corneal ulcer. It is an important cause of blindness in Nepal. A scratch in the cornea allows infection to enter and an ulcer to begin. These infections can be very serious with some people losing the sight in the affected eye.

Different types of infectious organisms can cause corneal ulcers. These include bacteria and fungi which need to be treated with different types of eye drop medicines. To find out what type of infection is present it is necessary to collect a small sample from the ulcer and perform various laboratory tests. The test results guide the treatment. In Nepal about half of the infections are caused by fungus.

Fungal infections can be difficult to diagnose. No single test can reliably detect it. It may need several different tests to find it. Currently, in many countries where fungal ulcers are common laboratory services are limited or not available. Tests are expensive and may not detect all of the infections. There is a need to understand which of the current test options give the most useful information.

The first purpose of this study is to carefully evaluate your eye infection using several different diagnostic tests to try to find out what type of infection you have, and to learn which tests are most useful in this process.

There is a need for alternative, affordable and more easily available eye drop treatments for fungal infections. We are also conducting a clinical trial to compare two eye drop treatments for fungal infection, to try to find out which is better.

Therefore, the second purpose of this initial Assessment and Investigation study is to identify people who have a fungal corneal infection who may be eligible and willing to enrol in the clinical trial. If we find out that you do have a fungal corneal infection, we will then ask if you would be interested in hearing about the clinical trial. If you are interested, then at that stage we will explain about the treatment trial in detail, provide you with a second information sheet and go throw a second consent process.

**Do I have to take part?**
No. Your involvement is entirely voluntary. If you agree to take part, we will then ask you to sign a consent form. If you decide to join and change your mind, you are free to withdraw at any time without giving a reason. This will not affect the standard of care you receive.
Why have I been invited?
You have been invited to take part because you have a corneal infection. To guide the treatment of this it is helpful to do tests to find out the cause, to guide the choice of eye drop treatment. We are asking for your permission to carry out some tests that are listed below to find out what is causing the infection and to decide if you may be suitable for a trial comparing treatments for fungal eye infections.

For peer review only

**What will happen to me if I take part?**
If you agree to be part of this study, the following will happen:

- We will ask you a series of questions. This will include basic demographic information, the history of your current eye problem, and treatment you have had before arriving at the hospital.
- We will then carefully examine both eyes using a special microscope.
- Your eyes will be photographed with a camera. This is additional to standard care, and will help us to monitor the infection and the response to treatment.
- We will use a special microscope to look at the cornea to see if we can find a fungal infection. This involves putting anaesthetic drops on the eye. A soft plastic device then gently touches the eye so to see if you have a fungal infection. This is additional to standard care, and will help us to find out the type of infection you have more rapidly so that we can offer you the most appropriate treatment.
- We will collect samples form the corneal ulcer to test in the laboratory to try to identify what is causing the infection. Anaesthetic eye drops will be used numb the eye so you will not feel anything while we collect the sample by brushing the surface.
-
- We will check your blood sugar level for diabetes. This is done using a finger-prick blood sample. If this is raised, we will refer you to a separate group of doctors to help you with this.
- You will be offered a test for HIV infection. This will be done through the hospitals counselling and testing services. If you choose to accept this, then you will be separately counselled about the test and the implications of the results. This would involve the collection of a blood test sample from your arm. If the HIV test is positive, then you will be referred to the appropriate team for ongoing care. The result of this test will be shared with the us (cornea infection study team), with your consent, as it is potentially relevant to the treatment of your cornea infection.
- We will collect a sample of the cells from the inside of your cheek by gently rubbing a swab for a few seconds. This is additional to the standard of care. The purpose is to try to understand how the body fights the infection and why some people develop this eye problem and others do not.
- You will be asked to complete three short questionnaires to assess your quality of life and vision function. This is additional to standard care, and will help us to better understand the impact corneal ulcers can have on people's lives.

If we find evidence of fungus infecting your eye, then we will discuss the details of the trial with you further. At that point we will give you a second information sheet to read (or read to you), and if you meet all the eligibility criteria for the trial, to then go through a second consent process with you.

If you do not have a fungal infection, for example if the infection is caused by bacteria, then your will receive standard ongoing treatment for this by the same team at the hospital.

You may be withdrawn from the study without your consent if the researchers believe it is in your best interest or if you fail to follow study procedures.

**What are the side effects or risks of taking part?**
- The anaesthetic eye drops usually sting for a few seconds and then your eye should feel more comfortable. There is a very low risk of allergic reaction from the anaesthetic eye drops.
- The procedures including the history taking, examination, photography, in vivo confocal microscopy and sample collection are part of the standard of care for assessing a corneal infection. The risk associated with these are very low and no different from routine care. To minimise discomfort, topical anaesthetic will be given before examinations and sample collection.
- You will experience a little pain when a blood sample is collected. The people collecting the blood sample will ensure that this is done with as little discomfort as possible.
- The questions, tests and examinations will add about 15 minutes to your hospital visit above what we would usually expect in assessing a patient with this problem.

**What are the benefits of taking part?**
- The study will involve tests for the type of infection. This helps the doctor looking after you to choose the best type of treatment for your eyes
- The costs for the clinical assessment and the tests will be paid for by the study.
- By participating in this study, you will be helping to further research into this field so more informed decisions can be made when treating people with corneal infection.

For peer review only

**What will happen to the clinical records, photographs and test results?**
Your records will remain strictly confidential at all times. The information will be held in a secure office at your treating hospital. Only the people organizing or supervising the trial and regulatory authority auditors will have access to it. These include officials delegated by the Sponsor (London School of Hygiene and Tropical Medicine), the local National Ethics Committee, The local National Drug Regulatory Authority and trial Data Safety Monitoring Body (DSMB).

Your name will not be passed to anyone else outside the research team, unless we have your direct instruction to do so, for example to make a medical referral.

The photographs of the eye and the result of the laboratory test for infection will be shared with computer engineers to help develop a programme that can automatically analyse the image to see if this can provide some indication of the cause of the infection. Images of corneal infection may also be used for educational and teaching purposes, including in publications. All personal identifying information will be removed before sharing images.

**What tests will we do on the sample?**
The samples collected from the surface of your eye will be tested in several different ways to determine what is causing the infection. This work will be done in the hospital microbiology laboratory, where you are being treated. A portion of the infection sample will be transferred for additional special tests at KCMC Hospital Biotechnology (Tanzania), the London School of Hygiene and Tropical Medicine (UK) and Radboud University Nijmegen Medical Center (The Netherlands), as some of the tests will require additional special equipment that is not available at all sites.

- We will look for the type of infection using a microscope and by growing the organisms in the laboratory. We will test the organisms that grow to see which medicines work best to kill the infection, which is helpful in guiding the choice of treatment to be used.

- The swab samples from the ulcer will be used to test for infection using molecular diagnostic tests and to evaluate new tests that may be used to find the cause rapidly in the clinic.

- We will use study genetic material of the organism causing the infection to find out the exact type of infection and its ability to resist treatments. Samples from the ulcer may also be used to investigate your immune response to the infection, so that we can better understand what causes corneal scarring

- We will store a sample of the infection causing organism indefinitely for additional testing.

- The genetic material from the cells from your cheek will be sent to the UK or to KCMC Hospital in Tanzania. We would like to store it until a later time when a sufficiently large number of samples has been collected to conduct an analysis of this genetic material. The purpose would be to try to better understand how the human immune system fights the infection and why some people develop this eye problem and others do not. By doing these tests we hope that it will help us to develop approaches that will help to prevent or improve outcomes from this condition.

- As part of this consent we are asking that you give us permission to store this material to be able to test it at a later date as mentioned above. We do not know exactly for how long we shall store the genetic material before we have assembled a sufficiently large collection during the course of several planned studies to be able to proceed to the sample analysis, however, we anticipate a period of at least five years.

**What will happen to the results of the research study?**
The results of the study will be available after it finishes and will be included in peer reviewed medical and scientific journals and may be presented at medical meetings. Results will also be published on a publicly accessible trials database. The data will be anonymous and none of the patients involved in the trial will be identified in any report or publication. Should you wish to see the results, or the publication, please ask your study doctor.

**Who is funding the research?**
The research is being funded as part of a grant from the Wellcome Trust, UK.

**Who is organising the research?**
It is being organised through a research partnership between the London School of Hygiene and Tropical Medicine and Sagarmatha Choudhary Eye Hospital in Nepal, Mbarara University of Science and Technology in Uganda and Kilimanjaro Christian Medical Centre in Tanzania.

**What if relevant new information becomes available?**
It is not anticipated that new information will become available during the course of this short study. The information from the microbiology tests will be available to the doctors to help select the most appropriate treatment for you.

For peer review only

**What if something goes wrong?**
If you have a concern about any aspect of this study, you should ask to speak to the researchers who will do their best to answer your questions. If you remain unhappy and wish to complain formally, you can do this through the head of the hospital eye department or the named person on the following page. The London School of Hygiene and Tropical Medicine holds insurance policies which apply to this study. If you experience harm or injury as a result of taking part in this study, you may be eligible to claim compensation.

**Who has reviewed the study?**
Prospective research such as this is looked at by an independent group of people, called a Research Ethics Committee, to protect your interests. This study has been reviewed and approved by (1) the London School of Hygiene and Tropical Medicine Research Ethics Committee; (2) Nepal Health Research Council.

**What will happen if I don't want to carry on with the study?**
Your participation in this study is entirely voluntary. You may refuse to participate or may withdraw from this study at any time without penalty or loss of any rights or benefits to which you are otherwise entitled. The study doctor may also stop your participation in the study at any time for safety reasons. If you decide to withdraw from the study you should contact a member of the study team immediately. You do not have to give a reason when stopping, however for safety reasons, it is suggested that you tell the study doctor if you decide to stop because of an unwanted side effect. If you withdraw from the study, we will only use data collected before this decision, unless you request this to also be withdrawn. If you withdraw from the study, researchers, authorized persons from the Sponsor and the regulatory authorities will still require access to your medical notes to verify the data collected up to the date of your withdrawal.

**Contact Details**
Nepal Study Site: Dr Reena Yadav, Sagarmatha Choudhary Eye Hospital, Lahan. T: +977 9807799958

Study Coordinator: Dr Jeremy Hoffman: email: Jeremy.hoffman@lshtm.ac.uk
Chief Investigator: Prof. Matthew Burton: email: matthew.burton@lshtm.ac.uk

**You will be given a copy of the information sheet and a signed consent form to keep.**
**Thank you for considering taking the time to read this sheet.**

For peer review only

**Randomised controlled trial of topical chlorhexidine 0.2% versus topical natamycin 5% for fungal keratitis**

## Consent Form No 1: Baseline assessment and investigations

**Participant Name**___________________________ **Study ID Number:** ____________________

| | Please initial box |
|---|---|
| 1. I confirm that I have read and understand the participant information sheet dated ……….. (version ………) for the above study. I have had the opportunity to consider the information, ask questions and have had these answered fully. | |
| 2. I understand that my participation is voluntary and I am free to withdraw at any time, without giving any reason, without my medical care or legal rights being affected. | |
| 3. I understand that sections of my medical notes and data collected during the study may be looked at by responsible individuals from the London School of Hygiene & Tropical Medicine, from national regulatory authorities or from this hospital, where it is relevant to my taking part in this research. I give permission for these individuals to access my records. | |
| 4. I agree to take part in the above study. | |
| 5. I agree to the collection, laboratory tests and storage for future analysis of the samples from the surface my eye infection to understand the disease as described above. | |
| 6. I agree to the collection and storage for future analysis of the samples from the inside of my mouth to investigate how human genes affect this disease. | |
| 7. I agree to having a blood sample collected to measure sugar levels | |
| 8. I agree to being referred for a HIV pre-test counselling, testing and for the results to be shared with the research team. | |
| 9. I agree for the photographs of the front of my eye to be used in the publication or report released on the study, and for teaching purposes, including on the internet. | |
| 10. I understand that data about/from me/the participant may be shared via a public data repository or by sharing directly with other researchers, and that I will not be identifiable from this information. | |

| Name of Participant *(printed)* | Signature/Thumbprint | Date |
|---|---|---|
| Name of Person taking consent | Signature | Date |

The participant is unable to sign. As a witness, I confirm that all the information about the study was given and the participant consented to taking part.

| Name of Impartial Witness *(if required)* | Signature | Date |
|---|---|---|

*1 copy for participant; 1 copy for Principal Investigator; 1 copy to be kept with hospital notes*

For peer review only

**Study Title: Randomised controlled trial of topical chlorhexidine 0.2% versus topical natamycin 5% for fungal keratitis in Nepal**

*Participant Information Sheet and Consent Form – No 2:*
*For enrolment into the clinical trial*

**Introduction**
Thank you for earlier participating in the first stage of the study. You are now being invited to take part in the clinical treatment trial part of this medical research study. Before you decide it is important for you to understand why the research is being done and what it will involve. Please take time to read or listen to the following information carefully and to talk to others about the study, if you wish. Ask us if there is anything that is not clear or if you would like more information. Do not sign the consent form unless you are satisfied with the answers to your questions and decide that you want to be part of this study. Take time to decide whether or not you wish to take part.

**Why have I been invited?**
You have been invited to take part because you have a corneal infection which we have found out from tests is caused by fungus.

**What is this study about?**
Infection of the clear part of the front of the eye (the cornea) is a corneal ulcer. It is an important cause of blindness in Nepal. A scratch in the cornea allows infection to enter and an ulcer to begin. These infections can be very serious with some people losing the sight in the affected eye.

Different types of infectious organisms can cause corneal ulcers. These include bacteria and fungi. In tropical regions about half of all corneal ulcers are caused by fungi. Bacteria and fungi need to be treated with different types of eye drop medicines.

Treatments for fungal eye infections are frequently not very effective, in addition access to these treatments in many countries is very limited and can be expensive. In some countries they are simply not available. Currently the most commonly used treatment for fungal corneal ulcers is an eye drop called Natamycin. There is a need for additional, alternative, affordable and more easily available eye drop treatments for fungal infections.

There is an antiseptic solution called Chlorhexidine. This is very effective at killing bacteria, fungi and other types of infectious organisms. It is used in medical care worldwide in several different ways. For example, it is used to clean skin before surgical operations, in antiseptic creams for skin cuts and as a mouth wash to prevent and treat mouth infections. It has been used in eye care for more than thirty years as an eye-drop preservative, for sterilizing contact lenses, for pre-operative topical antiseptic and for treating *Acanthamoeba* and fungal corneal infections.

About twenty years ago chlorhexidine eye drops were tested in two small clinical trials conducted in India and Bangladesh for the treatment of fungal corneal infections. The results of these studies suggested that chlorhexidine was as good as and possibly better than natamycin at controlling the infection. Neither eye drop had any serious side effect. However, the studies were not large enough to be certain.

Chlorhexidine is currently used in several countries for the treatment of fungal corneal infections when natamycin or alternative treatment is not working or is not available. We would like to conduct a large clinical to find out whether chlorhexidine 0.2% eye drops are as effective as or more effective than natamycin 5% eye drops for treating fungal corneal infections

**Do I have to take part?**
No. Your involvement is entirely voluntary. If you agree to take part, we will then ask you to sign a consent form. If you decide to join and change your mind, you are free to withdraw at any time without giving a reason. This will not affect the standard of care you receive. If you decide not to participate in the study, then you will be offered the standard treatment for fungal keratitis using natamycin.

**What will happen to me if I take part?**
If you agree to be part of this study, the following will happen:

***1) Baseline Assessment:***
As part of the initial assessment that has already taken place we have carefully examined yours eyes, taken photographs, performed a scan for infection and collected samples. The tests have found that you have a fungal infection in your cornea. The risks to an unborn or breast-fed baby from antifungal eye drops use are unknown. Therefore, pregnant and breastfeeding women are excluded from participating in this study. Pregnancy testing will be offered to potential female participants to confirm pregnancy status.

For peer review only

***2) Randomisation:***

We will randomly allocate you to one of the two treatment option: either chlorhexidine eye drops or natamycin eye drops. Sometimes we don't know which way of treating patients is best. To find out, we need to make comparisons between the different treatments. We put people into groups and give each group one of the two alternative treatments; the results are compared after some time to see if one is better. To try to make sure the groups are the same to start with, each patient is put into a group by chance (randomly). You have an equal chance of being put into the chlorhexidine or natamycin treatment group. Neither you nor the people examining your eyes will be told which treatment group you are in. It is important that neither you nor we know which of the two you are given. This information would be in our files, but we would not look at these files until after the research is finished. This is the best way we have for testing without being influenced by what we think might happen. We would then compare which of the two treatments has the best results.

***3) Treatment:***

Once you are allocated to one of the treatment groups, you will receive clear instruction on how to take the eye drops. For the first week we will ask you to take one drop every hour. For the second and the third weeks the frequency of the eye drops will be reduced to every other hour (2-hourly). After that the frequency and duration of treatment will depend on the severity of the infection and how it is responding. You will be given clear guidance on this by the eye doctor who is looking after you.

***4) Follow-up Assessment:***

Initially, most people with corneal infections stay in hospital for several days so that the clinical team can monitor the response of the infection to the treatment. For the purposes of the study, we would like to review the response to treatment and document the clinical findings at the following times after you start treatment: two days, 1 week, 2 weeks, 3 weeks, 2 months and 3 months.

On each occasion we will ask you a few questions about your eye and the treatment. We will measure your eye sight. We will examine the eye with a microscope and photograph it with a camera.

At 1 week, 2 weeks and 3 weeks we will repeat the *in vivo* confocal microscopy test that was done at your first assessment. This is done to see how the infection is responding to treatment. This involves putting anaesthetic drops on the eye so that you do not feel any discomfort. A soft plastic device then gently touches the surface of the eye so that we can take special photographs of the front of your eye ("scan")

At 1 week if you still have an open ulcer on the cornea we will repeat the sample collection to test for the ongoing presence of the infection. This involves first putting anaesthetic eye drops on the surface of the eye. Then gently scraping the surface of the corneal ulcer and testing for the presence of fungus and bacteria in the microbiology laboratory.

Sometimes fungal infections do not respond to the treatment. In such cases it may be necessary to alter the treatment or perform an operation. The eye doctors who will be looking after you will monitor your progress closely and advise you about further treatment might be needed.

At the three month review you will be asked to complete three short questionnaires to assess your quality of life and vision function. These are the same standardised questions used in the baseline assessment to try to understand the impact that different medical problems can have on people's lives.

You may be withdrawn from the study without your consent if the researchers believe it is in your best interest or if you fail to follow study procedures.

**What are the side effects or risks of taking part?**

1. **Random Allocation and Treatment Failure**: You will be randomly allocated to a treatment. The treatment you are allocated to may prove to be less effective or to have more side effects than the other study treatment or other available treatments.

   **It is important to recognise that corneal infection is a serious, sight threatening condition.** Many patients, whatever the treatment used, have reduced vision in the affected eye after it has resolved. In some people the affected eye will become blind. Sometimes the infection, despite lots of treatment, can progress to cause a hole to develop in the cornea (Perforation) and sometimes it is so severe it is necessary to perform an operation to remove the eye content.

2. **Local Irritation:** As with most eye drops, there is the risk of local irritation or stinging from either chlorhexidine or natamycin. This usually only lasts for a short time.

3. **Allergic Response**: Very rarely, either chlorhexidine or natamycin eye drops can provoke a local allergic reaction on the surface of the eye or the eyelids.

For peer review only

4. **Pregnancy and Breast Feeding:** The risks to an unborn or breast-fed baby from antifungal eye drops use are unknown. Therefore, pregnant and breastfeeding women are excluded from participating in this study.

5. **Natamycin 5% eye drops**: Natamycin is an approved antifungal medication that is currently being used for the treatment of fungal corneal ulcer. It is on the World Health Organisation Essential Medicines List for the treatment of fungal corneal infections. There are no known serious side effects with this medication. It may cause mild irritation and very rarely a local allergic response.

6. **Chlorhexidine 0.2% eye drops:** Chlorhexidine eye drops are used on the surface of the eye as an antiseptic before procedures and also in the treatment of fungal and other eye infections. It has not been associated we any serious side effects. It may cause mild irritation and very rarely a local allergic response. This concentration of chlorhexidine is approved to be used in much larger volumes as a mouth wash. It is considered to be safe and is not associated with any systemic side effects.

7. **Procedures:** including examinations, confocal microscopy, corneal sample collection and checking for the best glasses or contact lenses carry the same very small risk whether they are performed as part of this study or of usual care outside the study. To minimise discomfort, topical anaesthetic will be given before examinations and sample collection.

8. **Unknown Risks:** The treatments in this study may have rare side effects that are currently not known. If during the course of the study new information becomes available, the researchers will share this with.

**What are the possible benefits of taking part?**

- The study will involve tests for the type of infection. This helps the doctor looking after you to choose the best type of treatment for your eyes
- The costs for your clinical assessment, tests, treatment and transport will be paid for by the study.
- By participating in this study, you will be helping to answer the question about whether or not chlorhexidine is a suitable alternative treatment for fungal corneal infections.

**What will happen to the clinical records, photographs and test results?**

Your records will remain strictly confidential at all times. The information will be held in a secure office at your treating hospital. Only the people organizing or supervising the trial and regulatory authority auditors will have access to it. These include officials delegated by the Sponsor (London School of Hygiene and Tropical Medicine), the local National Ethics Committee, The local National Drug Regulatory Authority and trial Data Safety Monitoring Body (DSMB).

A study number rather than your name will be used on study records or the database wherever possible. Your name and other facts that might identify you will not appear when we present this study or publish its results.

Your name will not be passed to anyone else outside the research team, unless we have your direct instruction to do so, for example to make a medical referral.

Images of corneal infection may be used for educational and teaching purposes, including in publications. All personal identifying information will be removed before sharing images.

**What tests will we do on the sample?**

The samples collected from the surface of your eye will be tested in several different ways to determine what is causing the infection. This work will be done in the hospital microbiology laboratory, where you are being treated. A portion of the infection sample will be transferred for additional special tests at KCMC Hospital Biotechnology (Tanzania), the London School of Hygiene and Tropical Medicine (UK) and Radboud University Nijmegen Medical Center (The Netherlands).

We will look for the type of infection using a microscope and by growing the organisms in the laboratory. We will test the organisms that grow to see which medicines work best to kill the infection, which is helpful in guiding the choice of treatment to be used. The swab samples from the ulcer will be used to test for infection using molecular diagnostic tests and to evaluate new tests that may be used to find the cause rapidly in the clinic. We will use the genetic material of the organism causing the infection to sequence its genetic code, which helps us to find out the exact type of infection and its ability to resist treatments. We will store a sample of the infection causing organism indefinitely for additional testing.

**What will happen to the results of the research study?**

The results of the study will be available after it finishes and will be included in peer reviewed medical and scientific journals and may be presented at medical meetings. Results will also be published on a publicly accessible trials database. The data will be anonymous and none of the patients involved in the trial will be identified in any report or publication. Should you wish to see the results, or the publication, please ask your study doctor.

**Who is funding the research?**

For peer review only

The research is being funded as part of a grant from the Wellcome Trust, UK.

**Who is organising the research?**
It is being organised through a research partnership between the London School of Hygiene and Tropical Medicine and Sagarmatha Choudhary Eye Hospital in Nepal, Mbarara University of Science and Technology in Uganda and Kilimanjaro Christian Medical Centre in Tanzania.

**What if relevant new information becomes available?**
It is not anticipated that new information will become available during the course of this study. However, if it does, this will be shared with you by the researchers in case this affects whether you wish to continue in the study.

**What if something goes wrong?**
If you have a concern about any aspect of this study, you should ask to speak to the researchers who will do their best to answer your questions. If you remain unhappy and wish to complain formally, you can do this through the head of the hospital eye department or the named person on the following page. The London School of Hygiene and Tropical Medicine holds insurance policies which apply to this study. If you experience harm or injury as a result of taking part in this study, you may be eligible to claim compensation.

**Who has reviewed the study?**
Prospective research such as this is looked at by an independent group of people, called a Research Ethics Committee, to protect your interests. This study has been reviewed and approved by (1) the London School of Hygiene and Tropical Medicine Research Ethics Committee; (2) Nepal Health Research Council.

What will happen if I don't want to carry on with the study?
Your participation in this study is entirely voluntary. You may refuse to participate or may withdraw from this study at any time without penalty or loss of any rights or benefits to which you are otherwise entitled. The study doctor may also stop your participation in the study at any time for safety reasons. If you decide to withdraw from the study you should contact a member of the study team immediately. You do not have to give a reason when stopping, however for safety reasons, it is suggested that you tell the study doctor if you decide to stop because of an unwanted side effect. If you withdraw from the study, we will only use data collected before this decision, unless you request this to also be withdrawn. If you withdraw from the study, researchers, authorized persons from the Sponsor and the regulatory authorities will still require access to your medical notes to verify the data collected up to the date of your withdrawal.

**Contact Details**
Nepal Study Site: Dr Reena Yadav, Sagarmatha Choudhary Eye Hospital, Lahan. T: +977 9807799958

Study Coordinator: Dr Jeremy Hoffman: email: Jeremy.hoffman@lshtm.ac.uk
Chief Investigator: Prof. Matthew Burton: email: matthew.burton@lshtm.ac.uk

**You will be given a copy of the information sheet and a signed consent form to keep.**
**Thank you for considering taking the time to read this sheet.**

For peer review only

**Randomised controlled trial of topical chlorhexidine 0.2% versus topical natamycin 5% for fungal keratitis**

**Consent Form No 2: Enrolment into the clinical trial**

**Participant Name**____________________________ **Study ID Number:** ____________________

| | **Please initial box** |
|---|---|
| 1. I confirm that I have read and understand the participant information sheet dated .......... (version .........) for the above study. I have had the opportunity to consider the information, ask questions and have had these answered fully. | |
| 2. I understand that my participation is voluntary and I am free to withdraw at any time, without giving any reason, without my medical care or legal rights being affected. | |
| 3. I understand that sections of my medical notes and data collected during the study may be looked at by responsible individuals from the London School of Hygiene & Tropical Medicine, from national regulatory authorities or from this hospital, where it is relevant to my taking part in this research. I give permission for these individuals to access my records. | |
| 4. I agree to take part in this clinical treatment trial. | |
| 5. I agree to the collection, laboratory tests and storage for future analysis of the samples from the surface my eye infection to understand the disease as described above. | |
| 6. I agree for the photographs of the front of my eye to be used in the publication or report released on the study, and for teaching purposes, including on the internet. | |
| 7. I understand that data about/from me/the participant may be shared via a public data repository or by sharing directly with other researchers, and that I will not be identifiable from this information. | |

| Name of Participant *(printed)* | Signature/Thumbprint | Date |
|---|---|---|
| Name of Person taking consent | Signature | Date |

The participant is unable to sign. As a witness, I confirm that all the information about the study was given and the participant consented to taking part.

| Name of Impartial Witness *(if required)* | Signature | Date |
|---|---|---|

*1 copy for participant; 1 copy for Principal Investigator; 1 copy to be kept with hospital notes*

For peer review only

## Appendix 2: Monitoring for Harm

## Definitions

**Adverse Event (AE):** any untoward medical occurrence in a patient or clinical trial subject administered a medicinal product and which does not necessarily have a causal relationship with this treatment. *An AE can therefore be any unfavourable and unintended sign (including an abnormal laboratory finding), symptom, or disease temporally associated with the use of an investigational medicinal product (IMP), whether or not considered related to the IMP.*

**Adverse Reaction (AR):** all untoward and unintended responses to an IMP related to any dose administered. *All AEs judged by either the reporting investigator or the sponsor as having reasonable causal relationship to a medicinal product qualify as adverse reactions. The expression reasonable causal relationship means to convey in general that there is evidence or argument to suggest a causal relationship.*

**Unexpected Adverse Reaction:** an AR, the nature or severity of which is not consistent with the applicable product information (e.g. investigator's brochure for an unapproved investigational product or summary of product characteristics (SmPC) for an authorised product). *When the outcome of the adverse reaction is not consistent with the applicable product information this adverse reaction should be considered as unexpected. Side effects documented in the SmPC which occur in a more severe form than anticipated are also considered to be unexpected.*

**Serious Adverse Event (SAE)** or **Serious Adverse Reaction:** any untoward medical occurrence or effect that at any dose

- **Results in death**
- **Is life-threatening** – *refers to an event in which the subject was at risk of death at the time of the event; it does not refer to an event which hypothetically might have caused death if it were more severe*
- **Requires hospitalisation, or prolongation of existing inpatients' hospitalisation**
- **Results in persistent or significant disability or incapacity**
- **Is a congenital anomaly or birth defect**

Medical judgement should be exercised in deciding whether an AE/AR is serious in other situations. Important AE/ARs that are not immediately life-threatening or do not result in death or hospitalisation but may jeopardise the subject or may require intervention to prevent one of the other outcomes listed in the definition above, should also be considered serious.

**Suspected Unexpected Serious Adverse Reaction (SUSAR):** any suspected adverse reaction related to an IMP that is both unexpected and serious.

## Causality

Most adverse events and adverse drug reactions that occur in this study, whether they are serious or not, will be expected treatment-related toxicities due to the drugs used in this study. The assignment of the causality should be made by the investigator responsible for the care of the participant using the definitions in the table below.

| Relationship | Description |
|---|---|
| **Unrelated** | There is no evidence of any causal relationship |
| **Unlikely** | There is little evidence to suggest there is a causal relationship (e.g. the event did not occur within a reasonable time after administration of the trial medication). There is another reasonable explanation for the event (e.g. the participant's clinical condition, other concomitant treatment). |
| **Possible** | There is some evidence to suggest a causal relationship (e.g. because the event occurs within a reasonable time after administration of the trial medication). However, the influence of other factors may have contributed to the event (e.g. the participant's clinical condition, other concomitant treatments). |
| **Probable** | There is evidence to suggest a causal relationship and the influence of other factors is unlikely. |

For peer review only

| | |
|---|---|
| **Definitely** | There is clear evidence to suggest a causal relationship and other possible contributing factors can be ruled out. |
| **Not assessable** | There is insufficient or incomplete evidence to make a clinical judgement of the causal relationship. |

If any doubt about the causality exists the local investigator should inform the study coordination centre who will notify the Chief Investigators. The pharmaceutical companies and/or other clinicians may be asked to advise in some cases.

In the case of discrepant views on causality between the investigator and others, all parties will discuss the case. In the event that no agreement is made, the MHRA will be informed of both points of view.

## Reporting Procedures

All adverse events should be reported. Depending on the nature of the event the reporting procedures below should be followed. Any questions concerning adverse event reporting should be directed to the study coordination centre in the first instance. A flowchart is given below (Figure 5) to aid in the reporting procedures and the reporting form is shown in (Data Collection Appendix).

***Figure 5: Adverse event reporting flow-chart***

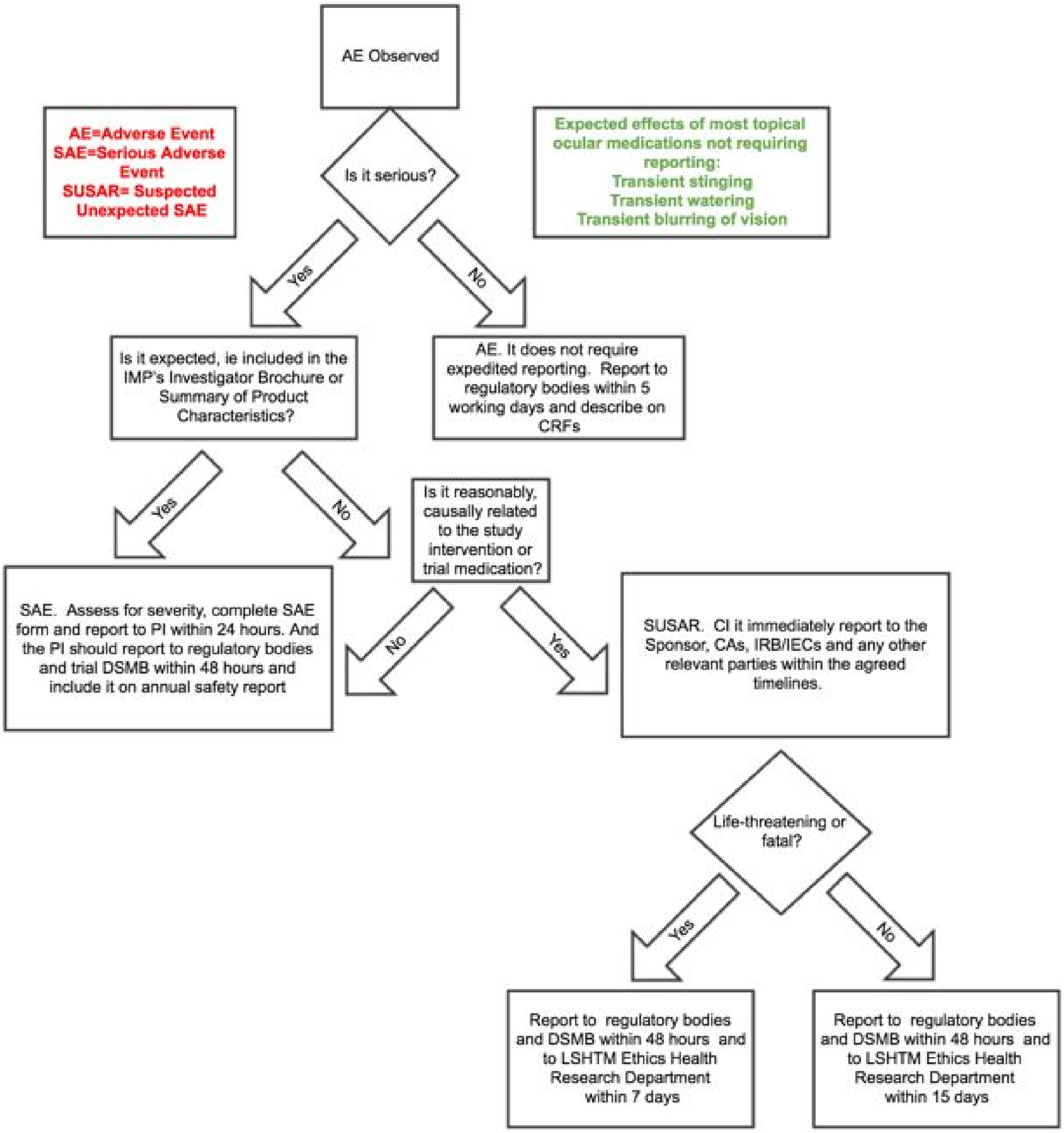

**Non-serious Adverse Reactions (ARs)/Adverse Events (AEs)**
All such events, whether expected or not, should be recorded in the toxicity section of the relevant case report form and sent to the study coordination centre, regulatory bodies and the trial DSMB within 5 working days from the day of occurrence.

**Serious Adverse Reactions (SARs)/Serious Adverse Events (SAEs)**
Fatal or life threatening Serious Adverse Events (SAEs), Serious Adverse Reactions (SARs) and Suspected Unexpected Serious Adverse Reactions (SUSARs) should be reported to the PI on the day that the local site is aware of the event. The SAE form asks for nature of event, date of onset, severity, corrective therapies given, outcome and causality (i.e. unrelated, unlikely, possible, probably, definitely). All SAEs or SARs whether expected, suspected or unexpected should be reported to regulatory bodies and the trial DSMB within 48 hours of occurrence. The responsible investigator should sign the causality of the event. Additional information should be sent within 5 days if the reaction has not resolved at the time of reporting. All investigators will be informed of all SARs occurring throughout the study.

**SAEs**
A SAE form should be completed and faxed to the study coordination centre for all SAEs within 24 hours. However, relapse and death due to a known pre-existing condition and hospitalisations for elective treatment of a pre-existing condition do not need reporting as SAEs.

**SUSARs**
In the case of serious, unexpected and related adverse events, the staff at the site should:

Complete the SAE case report form & send it immediately (within 24 hours, preferably by fax), signed and dated to the study coordination centre together with relevant treatment forms and anonymised copies of all relevant investigations.

**Or**

Contact the study coordination centre by phone and then send the completed SAE form to the study coordination centre within the following 24 hours as above.

The study coordination centre will notify all SUSARs occurring during the study within 48 hours to the Nepal Department of Drug Administration, and the trial DSMB. SUSARs will be reported to LSHTM Ethics Committee according to the following timelines; fatal and life-threatening within 7 days of notification and non-life threatening within 15 days. All investigators will be informed of all SUSARs occurring throughout the study.

Local investigators should report any SUSARs and /or SAEs as required by their Local Research Ethics Committee and/or Research & Development Office.

**Adverse Event Reporting for this Trial**
**Non-serious Ocular: narrative form not required**
- ☐ Local allergic reaction (conjunctiva, lid)
- ☐ Intraocular pressure ≥35 mmHg for 1 week despite therapy
- ☐ Progressive corneal thinning, ≤50% of enrolment thickness
- ☐ Other (specify)

**Serious Ocular: (1) report within 24hrs (2) Serious Adverse Event (SAE) Form required**
- ☐ Endophthalmitis
- ☐ Other serious ocular event thought to be related to the study drug, requiring non-elective hospitalization, loss of function, or systemic treatment (specify)

**Serious Non-ocular: (1) report within 24hrs (2) Serious Adverse Event (SAE) Form required**
- ☐ Death
- ☐ Non-elective surgery, hospitalization, or loss of function
- ☐ Myocardial infarction or stroke
- ☐ Systemic allergic reaction

For peer review only

## Appendix 3: Biological Specimens

The project will equip and staff an ocular microbiology laboratory at the hospital. The microbiology analysis will involve the following investigations:

1) **Microscopy** will be performed of the slides using Gram stain, KOH and calcofluor white.

2) **Culture** will be performed using a variety of media inoculated with corneal scrape samples in the clinic: blood agar, chocolate agar, brain-heart infusion broth (incubated for 2-7 days at 37°C) and Sabouraud agar (incubated for 7 days at 30°C). Culture isolates will be identified using standard microbiological techniques. Fungal and bacterial isolates will be stored frozen for long-term reference and subsequent additional analysis.

3) **Antibiotic susceptibility testing** of bacteria culture isolates will be performed using standard disc diffusion techniques in each of the laboratories.

4) **Antifungal susceptibility testing** of fungal isolates will be performed at KCMC, Tanzania . Isolates from Uganda and Nepal will be periodically transferred to KCMC, under a Material Transfer Agreement. They will be re-cultured and tested using a dilution series method for four drugs: Natamycin, Chlorhexidine, Voriconazole and Amphotericin-B. The technique has been developed at Radboud University, The Netherlands.

**PCR for microorganisms**

DNA from the dry swab will be extracted using the QIAamp mini kit (Qiagen) with bead-beating. Samples will be tested for the presence of bacterial (16rRNA), fungal (ITS1) and specific viral (HSV1&2) DNA by quantitative PCR. This PCR work will be performed at KCMC, Tanzania. Samples from Uganda and Nepal will be periodically transferred to KCMC for testing, under a Material Transfer Agreement. The performance of PCR as a diagnostic tool will be evaluated against a composite reference standard for positive cases. Cases will be considered positive if the pathogen is detected by either smear microscopy, IVCM or culture.

**Point of care tests for fungal infection**

Point-of-care tests for fungal keratitis would be very helpful. Molecular tests are generally more sensitive than antigen-based assays. Isothermal nucleic amplification techniques offer the sensitivity of PCR amplification without the need for specialized equipment. A result may be obtained within 30 minutes. Promising molecular approaches include isothermal amplification (Loop-mediated isothermal amplification (LAMP), Recombinase polymerase amplification (RPA)), associated with lateral flow assays for amplicon detection. Several assays have been developed that detect fungi, however, these have not been evaluated in fungal keratitis. The second ulcer swab will be used to test and develop isothermal molecular assays in comparison with the standard microbiological approaches described above. Results will be evaluated against a composite reference standard for fungal diagnosis as described above.

For peer review only

**Fungal DNA sequencing**

It is not always possible to determine the species of a fungus from classical microbiological techniques. It is now common to use fungal DNA sequencing for species-level diagnosis for some genera, including *Fusarium spp.*, which is the commonest fungal genus reported for MK in the study settings. We will use sequencing of the fungal ITS and TEF1α regions for this.[1] DNA will be extracted from the cultured fungal isolate when available. If there no fungus is cultured but the PCR is positive, then this PCR product will be used for sequencing. The DNA will be extracted from the isolates in KCMC, Tanzania. The extracted DNA will be sent under a Material Transfer Agreement to the Center of Expertise in Mycology Radboudumc/CWZ, Radboud University Nijmegen Medical Center and the CBS Fungal Biodiversity Centre, Utrecht, the Netherlands for the specialised sequencing and identification work.

**Corneal gene expression**

Three prime RNAseq (3'RNAseq) generates one read per transcript and enables strand-specific, highly multiplexed sequencing of poor concentration or quality samples (as low as 100pg total RNA input) for gene expression analysis. We have previously used this method for gene expression profiling of human conjunctival swabs from a trachoma-endemic area. RNA will be extracted from corneal swabs or scrapes using Norgen RNA purification kits. 3'RNAseq (Lexogen) will be performed on normalised RNA extracts from 500 Tanzanian and Ugandan participants with confirmed fungal keratitis. Pooled libraries will be sequenced on a HiSeq (Illumina). Transcriptomic profiles will be analysed for differences between controls and mild, moderate and severe FK cases using DEseq2, and between participants with good versus poor visual outcomes. A small number of healthy corneal tissue controls will be derived from the residual ring of tissue remaining after corneal graft surgery (ethics application for control tissue to be submitted separately).

**Human Genetics**

DNA will be extracted from buccal brush samples using QIAmp DNA mini kits (Qiagen). Samples from 500 confirmed fungal keratitis participants from Nepal and 500 matched controls will be run on immune-focussed Infinium ImmunoArray-24 BeadChips (Illumina) covering >240,000 genetic polymorphisms (including copy number variants, germline variants, insertions-deletions, single nucleotide polymorphisms (SNPs) and structural variants) at UCL genomics centre. In addition to SNP testing, two-field resolution Human Leukocyte Antigen (HLA) allele typing will be performed in all 1000 samples. Allele level HLA typing cannot currently be determined from SNP data but the HLA genes are primary immune response genes that have a role in many diseases and warrant independent study. Data will be analysed to determine sources of immunogenetic variation associated with fungal keratitis. These data will be linked to gene expression data from the same individuals to determine functional effects of genetic variation associated with fungal keratitis.

For peer review only

**HIV testing**

As outlined above, patients with microbial keratitis will be offered counselling and testing for HIV infection. This will be done through the established services in each of the hospitals. Appropriate onward referrals will be made if a test is found to be positive.

**Blood glucose testing**

Patients will have capillary blood glucose (CBG) testing done at presentation. Patients with an elevated CBG (>6.1mmol) will be referred to the hospital physicians for assessment and formal diagnosis of impaired glucose tolerance or diabetes mellitus. This level is considered a suitable cut-off to detect individuals with diabetes and has been validated in a south-Asian population.[2]

**References:**

1 Schoch CL, Seifert KA, Huhndorf S, *et al.* Nuclear ribosomal internal transcribed spacer (ITS) region as a universal DNA barcode marker for Fungi. *Proc Natl Acad Sci USA* 2012;**109**:6241–6. doi:10.1073/pnas.1117018109

2 Somannavar S, Ganesan A, Deepa M, *et al.* Random capillary blood glucose cut points for diabetes and pre-diabetes derived from community-based opportunistic screening in India. *Diabetes Care* 2009;**32**:641–3. doi:10.2337/dc08-0403

For peer review only

# Appendix 4: SPIRIT Checklist for Topical chlorhexidine 0.2% versus topical natamycin 5% for fungal keratitis in Nepal randomised clinical trial

| Section / Item | Item No. | Description | Location Reference |
|---|---|---|---|
| **Administrative information** | | | |
| Title | 1 | Descriptive title identifying the study design, population, interventions, and, if applicable, trial acronym | Page 1 of study protocol |
| Trial registration | 2a | Trial identifier and registry name. If not yet registered, name of intended registry | ISRCTN14332621; pre-results |
| | 2b | All items from the World Health Organization Trial Registration Data Set | ISRCTN14332621 Study Registry |
| Protocol Version | 3 | Date and version identifier | Every page of study protocol |
| Funding | 4 | Sources and types of financial, material, and other support 5a Names, affiliations, and roles of protocol contributors | Section 5.3 of study protocol, page 25<br>Page 17 of Manuscript |
| Roles and Responsibilities | 5a | Names, affiliations, and roles of protocol contributors | Page 1 of study protocol<br>Page 1 of Manuscript |
| | 5b | Name and contact information for the trial sponsor | Page 1 of study protocol<br>Page 1 of Manuscript |
| | 5c | Role of study sponsor and funders, if any, in study design; collection, management, analysis, and interpretation of data; writing of the report; and the decision to submit the report for publication, including whether they will have ultimate authority over any of these activities | Page 1 of study protocol and Section 5.2 of study protocol, page 25.<br><br>Page 17 of Manuscript |
| | 5d | Composition, roles, and responsibilities of the coordinating centre, steering committee, endpoint adjudication committee, data management team, and other individuals or groups overseeing the trial, if applicable (see Item 21a for data monitoring committee) | Section 5.1, page 24 of study protocol<br><br>Section 6, page 25 of study protocol<br><br>Page 11 of Manuscript |
| **Introduction** | | | |
| Background and rationale | 6a | Description of research question and justification for undertaking the trial, including summary of relevant studies (published and unpublished) examining benefits and harms for each intervention | Section 1, pages 6-9 of study protocol<br><br>Pages 4-5 of Manuscript |
| | 6b | Explanation for choice of comparators | Sections 1.3 – 1.4, pages 7-9 of study protocol<br>Pages 4-5 of Manuscript |

For peer review only

| | | | |
|---|---|---|---|
| Objectives | 7 | Specific objectives or hypotheses | Section 3, page 11 of study protocol<br><br>Page 5 of Manuscript |
| Trial design | 8 | Description of trial design including type of trial (eg, parallel group, crossover, factorial, single group), allocation ratio, and framework (eg, superiority, equivalence, noninferiority, exploratory) | Section 4, pages 12-24 of study protocol<br><br>Page 6 of Manuscript |
| **Methods: Participants, interventions, and outcomes** | | | |
| Study setting | 9 | Description of study settings (eg, community clinic, academic hospital) and list of countries where data will be collected. Reference to where list of study sites can be obtained | Section 4.3, page 13 of study protocol<br><br>Page 6 of Manuscript |
| Eligibility criteria | 10 | Inclusion and exclusion criteria for participants. If applicable, eligibility criteria for study centres and individuals who will perform the interventions (eg, surgeons, psychotherapists) | Section 4.4, page 13 of study protocol<br><br>Page 7 of Manuscript and Table 1 of Manuscript |
| Interventions | 11a | Interventions for each group with sufficient detail to allow replication, including how and when they will be administered | Section 4.7, page 17 of study protocol<br><br>Pages 8-9 of Manuscript |
| | 11b | Criteria for discontinuing or modifying allocated interventions for a given trial participant (eg, drug dose change in response to harms, participant request, or improving/worsening disease) | Page 11 of Manuscript |
| | 11c | Strategies to improve adherence to intervention protocols, and any procedures for monitoring adherence (eg, drug tablet return, laboratory tests) | Section 4.9, page 19 of study protocol<br><br>Pages 10-11 of Manuscript |
| | 11d | Relevant concomitant care and interventions that are permitted or prohibited during the trial | Section 4.7, pages 17-18 of study protocol<br>Pages 9-10 of Manuscript |
| Outcomes | 12 | Primary, secondary, and other outcomes, including the specific measurement variable (eg, systolic blood pressure), analysis metric (eg, change from baseline, final value, time to event), method of aggregation (eg, median, proportion), and time point for each outcome. Explanation of the clinical relevance of chosen efficacy and harm outcomes is strongly recommended | Section 4.11, page 22 of study protocol<br><br>Pages 10-11 of Manuscript |

For peer review only

| | | | |
|---|---|---|---|
| Participant timeline | 13 | Time schedule of enrolment, interventions (including any run-ins and washouts), assessments, and visits for participants. A schematic diagram is highly recommended (see Figure) | Table 3, page 19 of study protocol<br><br>Table 4 of Manuscript |
| Sample size | 14 | Estimated number of participants needed to achieve study objectives and how it was determined, including clinical and statistical assumptions supporting any sample size calculations | Section 4.12, page 22 of study protocol<br><br>Pages 12 -13 of Manuscript |
| Recruitment | 15 | Strategies for achieving adequate participant enrolment to reach target sample size | Section 4.5, page 14 of study protocol<br><br>Page 7 of Manuscript |
| **Methods: Assignment of interventions (for controlled trials)** | | | |
| Allocation: sequence generation | 16a | Method of generating the allocation sequence (eg, computer- generated random numbers), and list of any factors for stratification. To reduce predictability of a random sequence, details of any planned restriction (eg, blocking) should be provided in a separate document that is unavailable to those who enrol participants or assign interventions | Section 4.8, pages 18-19 of study protocol<br><br>Pages 7-8 of Manuscript |
| Allocation: concealment mechanism | 16b | Mechanism of implementing the allocation sequence (eg, central telephone; sequentially numbered, opaque, sealed envelopes), describing any steps to conceal the sequence until interventions are assigned | |
| Allocation: Implementation | 16c | Who will generate the allocation sequence, who will enrol participants, and who will assign participants to interventions | |
| Blinding (masking) | 17a | Who will be blinded after assignment to interventions (eg, trial participants, care providers, outcome assessors, data analysts), and how | Section 4.8, pages 18-19 of study protocol<br><br>Page 8 of Manuscript |
| | 17b | If blinded, circumstances under which unblinding is permissible, and procedure for revealing a participant's allocated intervention during the trial | Page 8 of Manuscript |
| **Methods: Data collection, management, and analysis** | | | |
| Data collection | 18a | Plans for assessment and collection of outcome, baseline, and other trial data, including any | Section 4.14, page 23 of study protocol |

For peer review only

| | | | |
|---|---|---|---|
| methods | | related processes to promote data quality (eg, duplicate measurements, training of assessors) and a description of study instruments (eg, questionnaires, laboratory tests) along with their reliability and validity, if known. Reference to where data collection forms can be found, if not in the protocol | Page 12 of Manuscript |
| | 18b | Plans to promote participant retention and complete follow-up, including list of any outcome data to be collected for participants who discontinue or deviate from intervention protocols | Page 12 of Manuscript |
| Data management | 19 | Plans for data entry, coding, security, and storage, including any related processes to promote data quality (eg, double data entry; range checks for data values). Reference to where details of data management procedures can be found, if not in the protocol | Section 4.14, page 23 of study protocol<br><br>Page 12 of Manuscript |
| Statistical methods | 20a | Statistical methods for analysing primary and secondary outcomes. Reference to where other details of the statistical analysis plan can be found, if not in the protocol | Section 4.13, pages 22-23 of study protocol<br><br>Pages 12-15 of Manuscript |
| | 20b | Methods for any additional analyses (eg, subgroup and adjusted analyses) | |
| | 20c | Definition of analysis population relating to protocol non-adherence (eg, as randomised analysis), and any statistical methods to handle missing data (eg, multiple imputation) | |
| **Methods: Monitoring** | | | |
| Data Monitoring | 21a | Composition of data monitoring committee (DMC); summary of its role and reporting structure; statement of whether it is independent from the sponsor and competing interests; and reference to where further details about its charter can be found, if not in the protocol. Alternatively, an explanation of why a DMC is not needed | Section 6, page 25 of study protocol<br><br>Page 12 of Manuscript |
| | 21b | Description of any interim analyses and stopping guidelines, including who will have access to | Section 4.13, page 23 of study protocol |

For peer review only

| | | | |
|---|---|---|---|
| | | these interim results and make the final decision to terminate the trial | Page 15 of Manuscript |
| Harms | 22 | Plans for collecting, assessing, reporting, and managing solicited and spontaneously reported adverse events and other unintended effects of trial interventions or trial conduct | Section 7, pages 26-28 of study protocol<br><br>Page 12 of Manuscript<br><br>Annex 2 |
| Auditing | 23 | Frequency and procedures for auditing trial conduct, if any, and whether the process will be independent from investigators and the sponsor | Section 6, page 25 of study protocol<br><br>Page 12 of Manuscript |
| **Ethics and Dissemination** | | | |
| Research ethics approval | 24 | Plans for seeking research ethics committee/institutional review board (REC/IRB) approval | Section 4.15, page 24 of study protocol<br>Page 15 of Manuscript |
| Protocol amendments | 25 | Plans for communicating important protocol modifications (eg, changes to eligibility criteria, outcomes, analyses) to relevant parties (eg, investigators, REC/IRBs, trial participants, trial registries, journals, regulators) | Section 4.15, page 24 of study protocol<br>Page 15 of Manuscript |
| Consent or assent | 26a | Who will obtain informed consent or assent from potential trial participants or authorised surrogates, and how (see Item 32) | Section 4.5, page 14 of study protocol<br><br>Page 7 of Manuscript<br><br>Appendix 1: Consent Forms |
| | 26b | Additional consent provisions for collection and use of participant data and biological specimens in ancillary studies, if applicable | |
| Confidentiality | 27 | How personal information about potential and enrolled participants will be collected, shared, and maintained in order to protect confidentiality before, during, and after the trial | Page 12 of Manuscript |
| Declaration of interests | 28 | Financial and other competing interests for principal investigators for the overall trial and each study site | Page 18 of submitted manuscript |
| Access to data | 29 | Statement of who will have access to the final trial dataset, and disclosure of contractual agreements that limit such access for investigators | Page 12 of Manuscript |
| Ancillary and post-trial care | 30 | Provisions, if any, for ancillary and post-trial care, and for compensation to those who suffer harm from trial participation | Page 9 of Manuscript |
| Dissemination policy | 31a | Plans for investigators and sponsor to communicate trial results to participants, healthcare | Section 10, page 33 of study protocol |

For peer review only

| | | | |
|---|---|---|---|
| | | professionals, the public, and other relevant groups (eg, via publication, reporting in results databases, or other data sharing arrangements), including any publication restrictions | Pages 15-16 of Manuscript |
| | 31b | Authorship eligibility guidelines and any intended use of professional writers | Page 24 of Manuscript |
| | 31c | Plans, if any, for granting public access to the full protocol, participant- level dataset, and statistical code | Page 24 of Manuscript |
| **Appendices** | | | |
| Informed consent materials | 32 | Model consent form and other related documentation given to participants and authorised surrogates | Appendix 1 of Manuscript |
| Biological specimens | 33 | Plans for collection, laboratory evaluation, and storage of biological specimens for genetic or molecular analysis in the current trial and for future use in ancillary studies, if applicable | Appendix 3 of Manuscript |

For peer review only

BMJ Open

# Topical chlorhexidine 0.2% versus topical natamycin 5% for fungal keratitis in Nepal: rationale and design of a randomised controlled non-inferiority trial

| | |
|---|---|
| Journal: | *BMJ Open* |
| Manuscript ID | bmjopen-2020-038066.R1 |
| Article Type: | Protocol |
| Date Submitted by the Author: | 22-May-2020 |
| Complete List of Authors: | Hoffman, Jeremy; London School of Hygiene and Tropical Medicine International Centre for Eye Health; Sagarmatha Choudhary Eye Hospital<br>Yadav, Reena; Sagarmatha Choudhary Eye Hospital<br>Das Sanyam, Sandip; Sagarmatha Choudhary Eye Hospital<br>Chaudhary, Pankaj; Sagarmatha Choudhary Eye Hospital<br>Roshan, Abhishek; Sagarmatha Choudhary Eye Hospital<br>Singh, Sanjay; Eastern Region Eye Care Programme<br>Arunga, Simon; London School of Hygiene and Tropical Medicine International Centre for Eye Health, International Centre for Eye Health; Mbarara University of Science and Technology Faculty of Medicine<br>Matayan, Einoti; Kilimanjaro Christian Medical Centre, Department of Ophthalmology<br>Macleod, David; London School of Hygiene & Tropical Medicine, MRC Tropical Epidemiology Group<br>Weiss, Helen; London School of Hygiene & Tropical Medicine, MRC Tropical Epidemiology Group<br>Leck, Astrid; London School of Hygiene and Tropical Medicine International Centre for Eye Health<br>Hu, Victor; London School of Hygiene and Tropical Medicine International Centre for Eye Health<br>Burton, Matthew J; London School of Hygiene and Tropical Medicine International Centre for Eye Health; Moorfields Eye Hospital NHS Foundation Trust |
| <b>Primary Subject Heading</b>: | Ophthalmology |
| Secondary Subject Heading: | Evidence based practice, Global health, Infectious diseases, Research methods |
| Keywords: | Corneal and external diseases < OPHTHALMOLOGY, OPHTHALMOLOGY, Clinical trials < THERAPEUTICS, MYCOLOGY |
| | |

SCHOLARONE™
Manuscripts

*I, the Submitting Author has the right to grant and does grant on behalf of all authors of the Work (as defined in the below author licence), an exclusive licence and/or a non-exclusive licence for contributions from authors who are: i) UK Crown employees; ii) where BMJ has agreed a CC-BY licence shall apply, and/or iii) in accordance with the terms applicable for US Federal Government officers or employees acting as part of their official duties; on a worldwide, perpetual, irrevocable, royalty-free basis to BMJ Publishing Group Ltd ("BMJ") its licensees and where the relevant Journal is co-owned by BMJ to the co-owners of the Journal, to publish the Work in this journal and any other BMJ products and to exploit all rights, as set out in our licence.*

*The Submitting Author accepts and understands that any supply made under these terms is made by BMJ to the Submitting Author unless you are acting as an employee on behalf of your employer or a postgraduate student of an affiliated institution which is paying any applicable article publishing charge ("APC") for Open Access articles. Where the Submitting Author wishes to make the Work available on an Open Access basis (and intends to pay the relevant APC), the terms of reuse of such Open Access shall be governed by a Creative Commons licence – details of these licences and which Creative Commons licence will apply to this Work are set out in our licence referred to above.*

*Other than as permitted in any relevant BMJ Author's Self Archiving Policies, I confirm this Work has not been accepted for publication elsewhere, is not being considered for publication elsewhere and does not duplicate material already published. I confirm all authors consent to publication of this Work and authorise the granting of this licence.*

For peer review only

# Topical chlorhexidine 0.2% versus topical natamycin 5% for fungal keratitis in Nepal: rationale and design of a randomised controlled non-inferiority trial

**Authors:**

Jeremy J Hoffman FRCOphth (1, 2, 5), Reena Yadav MD (2), Sandip Das Sanyam (2), Pankaj Chaudhary (2), Abhishek Roshan (2), Sanjay K Singh MD (3), Simon Arunga (1, 4), Einoti Matayan (5), David Macleod PhD (1, 6), Helen A Weiss PhD (6), Astrid Leck PhD (1), Victor H Hu PhD FRCOphth (1), Matthew J Burton PhD FRCOphth (1, 7).

**Affiliations:**

1. International Centre for Eye Health, London School of Hygiene and Tropical Medicine, London, UK
2. Sagarmatha Choudhary Eye Hospital, Lahan, Nepal
3. Eastern Region Eye Care Programme, Biratnagar, Nepal
4. Mbarara University of Science and Technology, Mbarara, Uganda
5. Kilimanjaro Christian Medical Centre, Moshi, Tanzania
6. MRC Tropical Epidemiology Group, London School of Hygiene & Tropical Medicine, London, UK
7. Moorfields Eye Hospital, London, UK

**Corresponding author:**

Jeremy J Hoffman FRCOphth

International Centre for Eye Health

London School of Hygiene and Tropical Medicine

Keppel Street

London

WC1E 7HT

United Kingdom

jeremy.hoffman@lshtm.ac.uk

**Key words:** Clinical trial; fungal keratitis; chlorhexidine; natamycin; corneal ulcer, Nepal.

**Word count:** 5132 (excluding abstract, titles, tables, figures and references)

For peer review only

## ABSTRACT

### Introduction

Fungal infections of the cornea, fungal keratitis (FK), are challenging to treat. Current topical antifungals are not always effective and are often unavailable, particularly in low and middle-income countries where most cases occur. Topical natamycin 5% is usually first-line treatment, however, even when treated intensively, infections may progress to perforation of the eye in around a quarter of cases. Alternative anti-fungal medications are needed to treat this blinding disease.

Chlorhexidine is an antiseptic agent with antibacterial and antifungal properties. Previous pilot studies suggest that topical chlorhexidine 0.2% compares favourably with topical natamycin. Full-scale randomised controlled trials (RCT) of topical chlorhexidine 0.2% are warranted to answer this question definitively.

### Methods and analysis

We will test the hypothesis that topical chlorhexidine 0.2% is non-inferior to topical natamycin 5% in a two-arm, single-masked RCT. Participants are adults with FK presenting to a tertiary ophthalmic hospital in Nepal. Baseline assessment includes history, examination, photography, *in vivo* confocal microscopy and cornea scrapes for microbiology. Participants will be randomised to alternative topical antifungal treatments (topical chlorhexidine 0.2% and topical natamycin 5%; 1:1 ratio, 2-6 random block size). Patients are reviewed at day 2, day 7 (with re-culture), day 14, day 21, month 2 and month 3. The primary outcome is best spectacle corrected visual acuity (BSCVA) at three months. Primary analysis (intention-to-treat) will be by linear regression, with treatment arm and baseline BSCVA pre-specified covariates. Secondary outcomes include epithelial healing time, scar/infiltrate size, ulcer depth, hypopyon size, perforation and/or therapeutic penetrating keratoplasty (TPK, corneal transplant), positive re-culture rate (day-7) and quality of life (EQ-5D, WHO/PBD-VF20, WHOQOL-BREF).

### Ethics and dissemination

The Nepal Health Research Council, the Nepal Department of Drug Administration and the London School of Hygiene and Tropical Medicine ethics committee have approved the trial. The results will be presented at local and international meetings and submitted to peer-reviewed journals for publication.

### Trial Registration

ISRCTN14332621; pre-results.

### Article summary

For peer review only

- First large-scale randomised controlled clinical trial comparing chlorhexidine 0.2% to natamycin 5% for the treatment of fungal keratitis.
- This study benefits from a pragmatic design: as a non-inferiority trial, if chlorhexidine is found to be within a pre-defined non-inferiority margin of 0.15 logMAR of natamycin at 3 months a recommendation to use chlorhexidine 0.2% can be made; this is a far cheaper, easy to formulate medication that could significantly increase access to anti-fungal treatment for the target population.
- Clinicians are masked to the treatment allocation, however, due to different physical appearance it is not possible to mask patients to their allocated treatment.
- This study will also assess the superiority of either medication as well as a number of key secondary outcome measures, analysed by arm.
- First randomised controlled trial investigating fungal keratitis to use *in vivo* confocal microscopy as a diagnostic tool for the detection of fungal elements.

For peer review only


For peer review only - http://bmjopen.bmj.com/site/about/guidelines.xhtml

## INTRODUCTION

Fungal keratitis (FK) is a severe and potentially blinding corneal infection (Figure 1). [1,2] The burden is greatest in tropical and subtropical countries, probably due to a combination of climate (higher temperatures and humidity) and frequent agriculture related eye injuries. [3] It is one of the causes of microbial keratitis (MK) and accounts for between 20 – 60% of corneal infections diagnosed in tropical regions. [4] It is often inadequately treated with significant barriers to receiving appropriate, timely intervention, compounded by indiscriminate and inappropriate use of conventional medicines such as topical corticosteroids or harmful traditional eye medicines. [1,2,5] Furthermore, when appropriate treatment is available, up to 30% of patients receiving current "gold-standard" therapy progress to corneal perforation and / or eye-loss (Figure 2). [1,6,7]

Treatment for fungal keratitis almost always involves topical antifungal agents. Surgical intervention, usually in the form of therapeutic penetrating keratoplasty (TPK), is generally reserved for cases of corneal perforation or progressive infection refractory to medical therapy. Corneal transplantation is also performed for visual rehabilitation after the acute infection has resolved. There are a limited number of antifungals available for treating FK, which fall into four main groups: imidazoles, triazoles, polyenes and fluorinated pyrimidines. These may be available topically, orally or by intravenous injection. Subconjunctival, corneal stromal or intracameral injections may also be given. The treatment of yeasts (*Candida spp.*) is often different to filamentous fungi, with the former being more common in temperate climates and the latter in hot and humid locations.[8,9]

There have been several clinical trials comparing treatment options for FK, which have been systematically reviewed.[10,11] Natamycin, which was approved in the 1960s by the FDA for FK, has been compared to a number of newer agents, including voriconazole. Natamycin and voriconazole have been compared in four trials, with the meta-analysis favouring natamycin. [6,10,12-14]

As a result, first line management of filamentous FK is usually with topical natamycin 5% when this is available. This was added to the WHO Essential Medicines List in 2017 for this indication. However, even when intensive topical natamycin is initiated, infections frequently progress relentlessly to perforation and loss of the eye in about a quarter of cases, Figure 2. [1,6,7] Moreover, in many countries antifungal eye drop treatments are simply not available. This includes most countries in SSA, some Asian countries and some countries in Europe.[1,2] Natamycin is relatively expensive even if it is available. Therefore, additional alternative and more affordable drugs are clearly needed if the outcome of these infections is to improve.


For peer review only - http://bmjopen.bmj.com/site/about/guidelines.xhtml
For peer review only

Chlorhexidine (CHX) is an antiseptic agent, with both antibacterial and antifungal properties. It is a widely-used broad-spectrum biocide, killing microorganisms through cell membrane disruption. [15-17] CHX has been used in ophthalmology for over 30 years as an eye-drop preservative and for sterilizing contact lenses, and has also been used to treat *Acanthamoeba* and fungal keratitis. [9,18-22] In a study of potential anti-fungal treatments, CHX was effective both *in vitro* against FK isolates from India and Ghana, as well as in an Indian case series.[23] Subsequently two pilot RCTs of CHX for fungal keratitis were conducted. In the first, three CHX concentrations (0.05%, 0.1%, 0.2%) were compared to each other and natamycin 5%; this concluded CHX 0.2% had reliable antifungal action. [19] The same concentration is used in mouthwash to prevent oral candidiasis. In the second trial, CHX 0.2% was compared to topical natamycin 2.5% (half standard concentration). There was evidence CHX produced a more favourable response by 5 days (RR=0.23, 95%CI 0.09-0.63). [18] A systematic review found a trend favouring CHX over natamycin in "curing" by 21-days (RR=0.70, 95%CI 0.45-1.09), suggesting CHX might prove superior in adequately powered trials.[10] CHX is safe and well-tolerated at these concentrations. [18,19,22,24] Based on this, CHX is used for treating FK in several countries. [1,9,11] However, the combined size of these two pilot trials comparing chlorhexidine and natamycin is not sufficient to reach firm conclusions. Currently, the meta-analysis indicates equipoise in terms of which treatment is best for treating fungal keratitis.

**Objectives**

The primary objective of this study is to determine if topical chlorhexidine 0.2% is non-inferior to topical natamycin 5% for treating fungal keratitis, in terms of vision at three months. The secondary objectives are: (1) to determine whether either treatment (chlorhexidine 0.2% or natamycin 5%) is superior to the other, in terms of vision at three months; and (2) to determine whether there is a difference between chlorhexidine 0.2% and natamycin 5% in terms of secondary clinical outcomes including infiltrate / scar size, time to re-epithelialisation, re-culture rates at one week and the effect of the alternative treatments on the Quality of Life of participants.

Chlorhexidine is cheap, stable and easily prepared by aqueous dilution. If chlorhexidine is found to be non-inferior (or even superior) to natamycin this offers the potential of an effective, affordable and accessible treatment for fungal keratitis, which could benefit millions of people each year who currently have no treatment options. This trial is a response to this expressed need from both clinicians and patients for a readily available and affordable medication for fungal infection.



For peer review only - http://bmjopen.bmj.com/site/about/guidelines.xhtml

## METHODS AND ANALYSIS

### Trial Design

We are conducting a single-masked, non-inferiority randomised controlled trial comparing chlorhexidine 0.2% to natamycin 5% for the treatment of fungal keratitis. The non-inferiority design of this trial offers a clinically pragmatic way to address this important question: if CHX is found to be within the pre-specified non-inferiority margin of 0.15 logMAR (about 1.5 Snellen lines) then CHX may prove to be a sustainable solution for this aspect of the complex problem of fungal keratitis.

### Trial Summary

This RCT follows a two-stage recruitment process (Figure 3). All patients presenting with acute microbial keratitis are reviewed and enrolled into Stage 1, following written, informed consent. This involves history, examination and investigations (corneal scrapes for microbiological assessment and *in vivo* confocal microscopy). If there is evidence of fungal hyphae on smear or confocal microscopy, patients then proceed to Stage 2. A trial eligibility checklist is completed and Stage 2 written informed consent is conducted. We will recruit 500 patients into Stage 2. Eligible FK patients are then randomised 1:1 to receive either natamycin 5% or chlorhexidine 0.2% topical treatments hourly for the first week, then two-hourly for the subsequent two weeks. Ongoing treatment duration will then be tailored to clinical response. Study personnel are masked to the treatment allocation. Patients are usually initially admitted and followed up on day-2, day-7 (with re-culture), day-14, day-21, month-2 and month-3. The primary outcome is best spectacle corrected visual acuity (BSCVA) at three months.

### Trial Setting

Low-land Nepal, a region with a high burden of fungal keratitis, provides a suitable location to conduct a clinical trial that requires a relatively large sample size. This trial will be conducted in Sagarmatha Choudhary Eye Hospital (SCEH), Lahan, Siraha District in south eastern Nepal. SCEH treats approximately 800 cases of keratitis per month, half of which is attributable to fungal infection. [1,25] SCEH is a tertiary level ophthalmic hospital, with several satellite Eye Care Clinics (ECCs) that refer patients to SCEH directly as required. SCEH and its ECCs serve an estimated population of about 5 million people. Due to its proximity to the border, approximately 50% of the outpatients are Indian nationals. It is anticipated that the study participants will present to the hospital from multiple districts within the region. Potentially eligible individuals will be recruited from outpatient clinics or referred directly by the ECCs to the study team. Based on the numbers of patients attending, it should be possible to complete recruitment of 500 participants within six to twelve months. A second separate trial in East Africa (Tanzania and Uganda) will also be conducted to compare chlorhexidine 0.2% to natamycin 5% following a very similar protocol to the one described here. This will be

For peer review only

registered as a separate trial. This will enable us to assess the generalisability of the findings in two geographically distinct regions with potentially different fungal aetiologies and susceptibility patterns.

**Eligibility Criteria**

Potential participants need to meet all the inclusion criteria and have none of the exclusion criteria listed in Table 1. In summary, they need to have an active fungal keratitis defined as acute microbial keratitis characterised by corneal epithelial ulceration (>1mm in diameter), corneal stromal infiltrate and signs of acute inflammation (e.g. conjunctival injection, anterior chamber inflammatory cells, hypopyon) in conjunction with evidence of a filamentous fungal infection on smear microscopy and/or *in vivo* confocal microscopy. As some patients will be enrolled on the basis of the results of *in vivo* confocal microscopy which is unable to detect most bacteria reliably, some patients with microscopically confirmed fungal infection will subsequently also be found to have had mixed infection at the time of being recruited into the study, as bacterial cultures may become positive a few days after enrolment. Based on previous experience at SCEH this is likely to account for about 10% of cases. These patients are included in the study but excluded from the primary analysis of the primary outcome (see below). Secondary analyses will include mixed infections.

**Table 1: Inclusion and exclusion criteria for enrolment in Stage 1 (microbial keratitis cases) and Stage 2 (the randomised controlled trial)**

| **Stage 1:** | |
|---|---|
| **Inclusion Criteria (all must be met):** | **Exclusion Criteria (any of the following):** |
| 1. Acute MK characterised by:<br>• Corneal epithelial ulceration >1mm diameter<br>• Corneal stromal infiltrate<br>• Acute inflammation: e.g. conjunctival injection, anterior chamber inflammatory cells, hypopyon.<br>2. Adults (18 years and older)<br>3. Able to provide informed consent | 1. Patients aged less than 18 years<br>2. Patients unable or unwilling to provide informed consent<br>3. Patients who do not have acute microbial keratitis or where there is a more likely alternative diagnosis |

| **Stage 2:** | |
|---|---|
| **Inclusion Criteria (all must be met):** | **Exclusion Criteria (any of the following):** |
| 1. Acute MK characterised by:<br>• Corneal epithelial ulceration >1mm diameter<br>• Corneal stromal infiltrate<br>• Acute inflammation: e.g. conjunctival injection, anterior chamber inflammatory cells, hypopyon.<br>2. Filamentous fungal hyphae visualised on smear microscopy and/or *in vivo* confocal microscopy.<br>3. Agree to be randomised to either treatment arm and are able to give informed consent<br>4. Agree to be followed up at 2 days, 1 week, 2 weeks, 3 weeks, 2 months and 3 months<br>5. Adults (18 years and older) | 1. Unwilling/unable to participate in trial and/or attend follow-up<br>2. Aged less than 18 years<br>3. Pregnancy: self-reported, or by urine hCG pregnancy test if uncertain.<br>4. Breast feeding: self-reported<br>5. Prior topical anti-fungal treatment<br>6. No light perception in the affected eye<br>7. Fellow eye visual acuity <6/60<br>8. Acanthamoebic infection visualised by smear microscopy or IVCM<br>9. Clinical evidence of herpetic keratitis<br>10. Known allergy to study medication (including preservatives)<br>11. Previous keratoplasty in the affected eye<br>12. Bilateral corneal ulcers<br>13. Very severe ulcers warranting immediate evisceration or conjunctival flap<br>14. Endophthalmitis |


For peer review only - http://bmjopen.bmj.com/site/about/guidelines.xhtml

**Consent Procedures**

There are two independent consent stages in this trial: Stage 1, where all adult patients with microbial keratitis are eligible; and Stage 2, only for fungal keratitis patients meeting the eligibility criteria. The two-stage process enables data collection on all potential patients at baseline before a diagnosis of fungal keratitis is confirmed. All patients who are eligible to participate will be given a participant information sheet in Nepali and its contents read to them. There will be an opportunity to discuss any questions that they might have. If the patient would like to participate, they will be asked to read and sign or place a thumb print on the study consent form. The consent will be witnessed by the eye health worker, confirmed by a signature on the form. For patients who are unable to read the documentation a second witness who is unrelated to the study is required. Consent Forms are given in Appendix 1.

**Baseline Assessment**

The detailed baseline assessment is described in Table 2. This includes clinical examination, corneal photography, *in vivo* confocal microscopy and the collection of microbiology samples. Quality of life questionnaires will also be completed (EQ-5D, WHO/PBD-VF20, WHOQOL-BREF).

**Table 2: Baseline assessment.** Assessment performed at baseline with details of how they are made.

| Assessment | Details |
| --- | --- |
| Visual Acuity | Presenting, Pin-Hole and Best Spectacle Corrected Visual Acuity (BSCVA) will be measured using an ETDRS Tumbling-E logMAR 3m chart (Good-Lite Inc., USA) mounted on an ESC 2000 ETDRS LED Cabinet, (Good-Lite Inc, USA) by a trial-certified optometrist, for each eye separately |
| Contrast Sensitivity | Measured using the Peek Contrast Sensitivity [26] smartphone application running on Android OS with a Sony Xperia Z3 Compact smartphone (Sony, Japan). |
| Clinical Photographs | Photographs will be taken separately of both corneas using a Nikon D7500 camera with an AF-S Micro Nikkor 105mm lens and lens mounted SB-200 flash units (Nikon, Japan). A standardised photography protocol is used to ensure images can be compared between time points. Standardised magnification will be used to allow epithelial defect and stromal infiltrate size measurements to be made. |
| Slit-lamp examination | Both eyes will be examined using a slit-lamp biomicroscope (standard ophthalmology examination) to assess the anterior segment of the eye. This examination will be performed by an ophthalmic clinician experienced in managing microbial keratitis. Particular attention will be paid to the following features:<br>1. Eyelids: trichiasis, lagophthalmos, facial weakness, Bell's reflex<br>2. Suppuration<br>3. Conjunctival inflammation<br>4. Corneal sensation<br>5. Cornea epithelial defect (measuring the longest dimension and the longest perpendicular) and ulcer depth<br>6. Corneal inflammatory infiltrate depth, size, profile, colour, edge pattern, texture, satellites.<br>7. Anterior chamber inflammatory cells, hypopyon, endothelial plaque<br>8. Relative afferent pupillary defect. |
| *In vivo* confocal microscopy (IVCM) | The Heidelberg Retinal Tomograph 3 (HRT3) *in vivo* confocal microscope enables the clinician to examine the cornea down to the cellular level. It is able to detect the presence of fungal hyphae.[27,28] A sterile, single-use disposable cap covers the objective lens and is changed between patients. Volume scans will be performed which provide a series of 400 x 400µm |



For peer review only - http://bmjopen.bmj.com/site/about/guidelines.xhtml

| | |
|---|---|
| | images over a depth range of 80µm. The resolution of the corneal scanning module is 7.6µm. IVCM images will be collected in a systematic way, starting at the centre of the ulcer, then at the superior, inferior, nasal and temporal borders of the ulcer. Volume scans will be performed in all of these locations, starting at the level of the corneal epithelium, and ending at the deepest affected aspect of the cornea assessed from IVCM images. Images will be assessed during the examination. |
| Ocular Sample Collection | The following samples will be collected from the corneal ulcer of each patient at the baseline assessment:<br>1. Corneal scrape specimens for microscopy and microbiological culture. A corneal scrape will be collected from the corneal ulcer after application of preservative free proxymetacaine local anaesthetic eye drops (Minims). Sterile needles are used to take corneal scrape specimens and then place on to glass slides for immediate Gram stain, KOH, and Calcofluor white. Samples will be directly inoculated onto blood, chocolate, Sabouraud agar and broths for culture.<br>2. Corneal specimen collection for PCR. Two sterile swabs will be gently swept over the surface of the corneal ulcer and placed into a 2ml tube. The swabs will be for pathogen detection by PCR, fungal sequencing and assessment of point of care tests for fungal infections. Swabs will be stored dry at -80°C. If swab yields are found to be too low for analysis an additional corneal scrape will be collected for PCR. The analysis of the PCR samples will not form part of the RCT work up and report. |
| HIV Testing | All individuals presenting with MK would be offered counselling and testing services. If this is found to be positive and the patient is unknown to the HIV care services an appropriate referral will be made. HIV testing is performed using HIV Tri-Dot rapid diagnostic test (J. Mitra & Co. Pvt. Ltd., India) |
| Random Blood Glucose | There is a suggestion that individuals with diabetes may be more susceptible to fungal keratitis. Participants will be offered a random blood glucose test, on a finger prick sample, analysed using HumaLyzer Primus (HUMAN Gesellschaft für Biochemica und Diagnostica mbH, Germany). If this is above 6.1 mmol/l they will be referred to the hospital physicians for assessment and formal diagnosis of impaired glucose tolerance or diabetes mellitus. This level is considered a suitable cut-off to detect individuals with diabetes and has been validated in a south-Asian population.[29] |
| Quality of Life Questionnaires | For those with confirmed fungal keratitis and who are enrolled in the trial, there will be several additional baseline assessments to evaluate the impact of FK on quality of life.<br><br>Vision Related Quality of Life (VRQoL): will be assessed by a vision disease specific tool the WHO/PBD-VF20. [30] This tool measures the impact of visual impairment in the person's life including mental wellbeing, dependency and social functioning. These have been used in a number of other vision related studies to show a difference in QoL.[31,32] This instrument consists of 20 questions divided into three sub-scales: visual symptom, general functioning and psychosocial. It begins by asking the patient *"Overall, how would you rate your eyesight using both eyes?"*; and uses a five point scale answer option such as *"very good", "good", "moderate", "bad", "very bad"*. The test is scored out of 100, with higher scores reflecting a better VRQoL.<br><br>General Health Related Quality of Life (HRQoL): We will use the EQ-5D questionnaire and EQ-Visual Analogue Scale. The EQ-5D is a standardised tool to measure health outcomes. [33] Patients will also be assessed using the WHOQOL-BREF. This has good applicability in low and middle-income countries (LMIC) as it was developed simultaneously from concept across 18 countries in Africa, Asia and Latin America. It measures 4 domains of health: Physical Health, Psychological Health, Social Relationships, and Environment. It asks respondents 26 questions how much (frequency) they have experienced and/or were able to do things (e.g. feel safe, able to concentrate, enjoy life) in the past 4 weeks and how satisfied they are with certain aspects of their lives (e.g. sleep, capacity for work).[33] |

For peer review only

**Randomisation and Masking**

***Sequence generation***: a computer-generated randomisation list will be prepared by an independent statistician at LSHTM, who will hold the sequence, will not be masked, and will not be involved in any other aspect of the trial. The sequence is in a 1:1 allocation ratio of CHX to NATA, with a random block size (2, 4 or 6).

***Allocation concealment and implementation:*** the randomisation sequences will be concealed in sequentially numbered, opaque envelopes. The envelopes will be prepared by a person independent of all other aspects of the trial. The randomisation administrator (nurse or pharmacist) will conduct the random allocation procedure. The investigational products will be stored in the trial coordination office in a locked and separate drug cabinet dedicated for this clinical trial only. The cabinet will only be used for drug storage and will only be accessed by the randomisation administrator. The randomisation administrator will work in a separate room in the clinic. After the patient has been recruited and assessed, they will be guided to this separate room where the randomisation allocation will be conducted.

***Masking:*** The topical treatments being compared in this trial have a different appearance: the chlorhexidine 0.2% is a clear, colourless solution and the natamycin 5% is an opaque, white suspension. Therefore, it is not possible to mask the participants to this difference in visual appearance. However, the patients will not be told which treatment they have been allocated. Prior to any follow-up clinical examinations, a nurse, otherwise uninvolved in the study, will wipe away any white natamycin residue from the patient's eyes to avoid unmasking the clinical assessor. This procedure was used successfully in other trials.[6] All clinicians involved in the clinical assessment of the patients will be masked to the allocation. The statistician who will perform the primary analysis will be masked to the allocation and only receive the actual allocation code sequence from the independent statistician after the analysis code has been prepared and pre-tested with a test sequence. The primary outcome is visual acuity at 3-months assessed by an optometrist who will not have been involved in any other aspects of the trial and masked to the allocation. By three months the treatment courses are likely to be completed. There will be masked grading of the photographs to independently confirm outcome measures and assess for any systematic bias on the part of the clinical examiners.

***Unmasking:*** Unmasking is a serious action and should only be performed if necessary to ensure the safety of a study participant. Anyone unmasked to randomised treatment for the purpose of creating analyses for independent data and safety monitoring committees will not be involved in any other aspects of the conduct or final analysis of the study. A list will be maintained of all unmasked members of staff and will be approved by the Chief Investigator. Such staff members must sign a document to indicate that they are aware of their


For peer review only - http://bmjopen.bmj.com/site/about/guidelines.xhtml

responsibilities with respect to confidentiality. The processes used to provide access to unmasked treatment codes and reports relating to these codes shall be documented.

**Intervention and Treatment**

The standard of care in this hospital is for cases of fungal keratitis to be admitted. If willing, patients will be admitted for close observation and supervised treatment, until there are signs of improvement and the supervising clinician considers it safe to provide ongoing management on an outpatient basis.

***Trial Treatment Arms***

A) *Chlorhexidine 0.2% w/v eye drops*

Chlorhexidine 0.2% w/v solution, as eye drops, will be applied to the surface of the infected eye (one drop per application). The chlorhexidine 0.2% w/v eye drops used in these studies is produced by Mandeville Medicines, UK.

B) *Natamycin 5% eye drops*

Natamycin 5% w/v suspension, as eye drops, will be applied to the surface of the infected eye (one drop per application) Topical natamycin 5% is produced by FDC Pharmaceuticals, India.

***Dosing Schedule:*** Both trial treatment arms will follow the same dosing schedule. Eye drops will be given hourly day and night for 48 hours, then hourly whilst awake for five days, and then two-hourly whilst awake for two further weeks. If the ulcer has healed (epithelial defect less than 1mm, infiltrate resolved, with or without corneal scarring), then treatment is stopped. If resolving stromal infiltration and/or epithelial defect >1mm but < 5mm, treatment is reduced to four times daily. If resolving but with epithelial defect > 5mm and/or stromal infiltration / hypopyon, treatment is reduced to six times daily. Treatment duration will be tailored to clinical response, with patients reviewed regularly whilst on treatment in addition to their scheduled study visits.

***Topical treatments:*** Several other topical medicines are used in the standard care of people with corneal infections:

1. **Fluorescein sodium ophthalmic strips** (Contacare Ophthalmics and Diagnostics, India) to highlight the area of cornea epithelial defect.
2. **Anaesthetic eye drops** to anaesthetise the cornea before for procedures such as microbiology samples: Proxymetacaine 0.5% eye drop Minims (Bausch and Lomb, UK).
3. **Antibiotic eye drops** may be used as per the judgement of the treating ophthalmologist if there is a suspicion of mixed bacterial and fungal keratitis: Moxifloxacin 0.5% eye drops (Centaur Pharmaceuticals, India).



For peer review only - http://bmjopen.bmj.com/site/about/guidelines.xhtml

4. **Mydriatic eye drops** for pupil dilation to reduce discomfort from the infection: Cyclopentolate 2% eye drops (Aurolab, India), three times a day.
5. **Ocular hypotensive eye drops** if the intra-ocular pressure is elevated >25 mmHg. This will be at the discretion of the supervising clinician; usual first line treatment is timolol 0.5% eye drops (Allergan, India).

***Ancillary treatment in patients refractory to trial medication:*** In patients with progressive fungal keratitis despite 7 days or more of trial medication, additional treatment options will be considered and offered. After repeating microbiological tests to rule out a mixed bacterial infection, deteriorating patients are started on topical voriconazole 1% hourly if the ulcer is superficial only. For ulcers that are deeper than 75% of the corneal thickness, oral ketoconazole 200mg twice-daily is added (with monitoring of liver function). The choice of ancillary treatment is due to local availability. If, following 7 days of additional treatment, despite these measures there is ongoing progression, surgical management such as a TPK can be considered.

***Non-pharmacological treatment:*** It is sometimes necessary to perform surgical procedures during the management of corneal infections. In the context of the trial this will be performed by the supervising consultant ophthalmologist. These can include:

1. Insertion of a bandage contact lens for very small perforations.
2. Tissue glue and patch for small perforations
3. Corneal transplant (TPK) for progressive fungal infections that are refractory to medical management or large perforations.
4. Conjunctival flaps to cover corneas in non-healing ulcers.

**Outcome Measures**

***Primary Outcome Measure:***

The primary outcome will be best spectacle corrected visual acuity (BSCVA) in logMAR units at three months. This will be measured by a trial-certified optometrist, independent of all other aspects of the study and masked to allocation. This has been the primary outcome of several other trials and will therefore facilitate comparison. Three months is the time at which clinical experience suggests most corneal ulcers have usually healed. BSCVA is chosen as it is easy to measure and is of functional significance. We will measure the BSCVA using an LED-backlit, Tumbling-E LogMAR chart (Good-Lite Co., IL, USA) under controlled conditions. We will also measure vision using Peek Acuity, a validated smartphone application, in situations where the patient is unable to attend the hospital for follow-up and outcome data is only available through a domiciliary visit. [34] For patients who have CF vision or less, pre-defined logMAR values will be assigned based on previous clinical trials. [35,36] Similarly, for patients who undergo a corneal transplant (therapeutic penetrating keratoplasty, TPK), a pre-defined visual acuity of 1.9 logMAR will be given, or last observation carried forward (LOCF, whichever is the better vision), as per previous studies. [6]


For peer review only - http://bmjopen.bmj.com/site/about/guidelines.xhtml

***Secondary Outcome Measures:***

We will be assessing a number of secondary outcome measures that relate to different measures of visual acuity such as Peek Acuity; clinical signs of healing such as reduction of epithelial defect; microbiological culture rates; and other clinical outcome measures such as scar size or perforation rate. These secondary outcomes together with their analyses are outlined in the Analysis Plan below.

**Outcome Assessments**

Participants will be re-assessed at 2-days, 1-week, 2-weeks, 3-weeks, 2-months and 3-months following enrolment. Additional examinations, outside the trial protocol schedule may be conducted by the supervising clinician as indicated. The specific assessments to be carried out at each visit are indicated in Table 3. These will be conducted in the same way described for the baseline assessment (Table 2). At each follow-up assessment the participant will be asked about adherence to treatment and symptoms, including side effects, if still receiving trial treatment. This will be recorded in the Case Record File. To monitor trial medication adherence, the study participants will be asked to bring their eye drop bottles to the 1, 2- and 3-week follow-ups. The amount of remaining medication will be measured by weighing the bottle and will be compared with the anticipated remaining amount provided the drops were being used as instructed, resulting in a ratio of actually remaining to what is anticipated. Participants will be resupplied with medication as needed. The presenting visual acuity will be measured on each visit. In addition, the BSCVA will also be measured at the three-month follow-up (primary outcome measure). At the final 3-month follow-up the three quality of life questionnaires will be repeated. On each occasion the eyes will be examined using a slit-lamp and the cornea photographed. The *in vivo* confocal microscopy will be repeated at 1, 2 and 3 weeks to determine whether there is evidence of fungal hyphae resolution with the fragmentation of linear elements. At the 1-week follow-up if the ulcer has not healed it will be re-scraped for repeat culture to determine whether the keratitis is now culture-positive or culture-negative. We will give the study participants appointment cards for the next follow-up and they will be reminded about the follow-up a week prior to the date. Public transport costs will be paid for participants who are outpatients.

For peer review only

**Table 3: Baseline and follow-up assessment components.**

| Assessment Item | Baseline | Day 2 | Day 7 | Day 14 | Day 21 | Day 60 | Day 90 |
|---|---|---|---|---|---|---|---|
| History / Baseline questionnaire | X | | | | | | |
| Check treatment adherence | | X | X | X | X | X | X |
| Check for side effects | | X | X | X | X | X | X |
| Visual Acuity – Presenting | X | X | X | X | X | X | X |
| Visual Acuity – BSCVA | X | | | | | | X |
| Contrast Sensitivity | X | | | | | | X |
| Slit-lamp Examination | X | X | X | X | X | X | X |
| Cornea Photography | X | X | X | X | X | X | X |
| *In vivo* confocal microscopy | X | | X | X | X | | |
| Cornea samples (Microbiology/PCR) | X | | X | | | | |
| Quality of Life Tools | X | | | | | | X |

**Treatment Review**

At each follow-up visit participants will be reviewed by an ophthalmic clinician experienced in the management of microbial keratitis. Clinical responses to anti-fungal therapy tend to be relatively slow (compared to bacterial infections). Prolonged topical treatment courses of 4-6 weeks are usually needed. Therefore, a change in therapy is usually not made for at least the first week. Other interventions may be indicated such as application of glue to corneal perforations, conjunctival flaps or therapeutic penetrating keratoplasty (corneal transplant).

**Stopping Rules**

If the study eye develops any serious adverse outcomes, the antifungal study medication may be discontinued if it is considered to be responsible for the adverse event. The patient will then be treated at the discretion of the supervising ophthalmologist, without breaking the randomisation code. If the medication is stopped, the patient will continue with the scheduled follow-up examinations.

**Loss to Follow-up**

Loss to follow-up rates are expected to be low, based on clinical experience. Participants who do not present for their follow-up visit will be contacted by telephone. Reasons for the loss to follow-up will be identified. Patients will be counselled about the importance of attending for ongoing treatment and monitoring. If they are unwell or unable to attend the hospital for some specific reason the study team will arrange to visit them in their home. Reasons for loss to follow-up will be recorded and reported.

**Data collection, management, confidentiality and access to data**

Data will be collected using paper Clinical Record Forms (CRFs). These will be stored securely at SCEH and scanned electronic copies taken at the end of the day, stored on an encrypted drive with encrypted backups made daily both on and off-site. These data will be double entered into two separate MS Access databases. After double data entry has been completed data will be cleaned using EpiData version 3.1 software. Data

For peer review only



For peer review only - http://bmjopen.bmj.com/site/about/guidelines.xhtml

entry is supervised by the local study co-ordinator on a daily basis, with data collection and data entry progress being reviewed by the study co-ordinator and chief investigator at LSHTM on a weekly basis. Data protection and confidentiality is maintained through restricted access to the database system and cupboard where the paper documents are kept. Only authorised users will have access to the locked filing cabinet. The database will be password protected, with each data entry staff member having their own password. Data exports for further analysis will be anonymized.

**Data and Safety Monitoring Board**

The Data and Safety Monitoring Board (DSMB) for this trial includes independent experts in bioethics, biostatistics, epidemiology and ophthalmology appointed by the Trial Steering Committee and approved by the national regulatory authorities, before the start of the study. The DSMB meets at least twice each year and organises teleconferences as needed for progress reporting. The study protocol and modifications are subject to review and approval by ethics committees in Nepal and LSHTM, and by the DSMB. The DSMB will monitor any severe or unexpected events and oversees the data collected. The DSMB will be responsible for reviewing the results of the interim analysis and determining whether or not the trial should continue, with or without modifications.

**Monitoring for Harm**

Patients will be monitored at each visit for adverse events or reactions. We will follow standardised definitions for adverse events, adverse reactions, unexpected adverse reactions, serious adverse events or reactions, and suspected unexpected serious adverse reaction. These, along with the reporting scheme, are given in Appendix 2.

**Biological specimens**

The processing and analysis of biological specimens are detailed in Appendix 3.

**Sample Size Considerations**

The study is powered to test the hypothesis that CHX is non-inferior to NATA in terms of the primary outcome (BSCVA at 3 months) at a pre-specified non-inferiority margin (Delta) of 0.15 logMAR units. It is possible that CHX is non-inferior to NATA, given pilot RCT data that showed no statistically significant difference between CHX and NATA, with a Cochrane review finding a non-significant trend favouring CHX over NATA.[10,18,19] Despite this trend favouring CHX, we have chosen to carry out a non-inferiority trial rather than a superiority trial as this is a more clinically pragmatic approach. It would be counter-productive to conduct a superiority trial and find no statistically significant difference between the two treatments leading clinicians to potentially disregard CHX as a treatment, when in fact it may be 'non-inferior" to NATA, and be the only available or most cost-effective treatment.



For peer review only - http://bmjopen.bmj.com/site/about/guidelines.xhtml

The choice of Delta: This clinically meaningful difference of 0.15 logMAR was chosen as a difference of 0.15 logMAR corresponds to approximately 1.5 lines on a Snellen chart; any difference greater than this is clinically significant, as a difference of less than 0.15 log MAR could potentially be accounted for by testing / re-testing error.[34] Furthermore, it was used in the MUTT1 trial, providing methodological consistency between studies.[6] In addition, previous studies have suggested treated ulcers improve at a mean of four Snellen lines from baseline.

Sample size was calculated for 90% power and adjusted final alpha of 0.0492, taking account of a single interim analysis using O'Brien-Fleming approach to maintain type-1 error rate of 5%. A sample size of 452 is required to detect a non-inferiority margin of 0.15-logMAR in BSCVA 3-months after enrolment between arms, assuming 0.5 SD for 3-month BSCVA and 15% dropout. However, given approximately 10% of infections are mixed and these will be excluded from the primary analysis, 500 patients will be recruited. This sample size provides 90% power to detect superiority in BSCVA at 3-months if there is ≥0.17 LogMAR units difference as a secondary analysis.

**Analysis Plan**

The analysis will be by intention to treat. All patient data will be analysed according to their randomisation allocation irrespective of whether or not the patient received or adhered to the allocated treatment. CONSORT guidelines for analysing/reporting non-inferiority RCTs will be followed. A flowchart showing cases assessed, recruited and followed-up by arm will be prepared.[37] Baseline characteristics will be summarised by arm. The SPIRIT checklist is given in Appendix 4.

***Primary Outcome Analysis – unadjusted analysis:*** The primary analysis will be conducted using all available data, missing data due to loss to follow up will be excluded. The primary analysis of the primary outcome (BSCVA at 3 month) will be by linear regression, with treatment arm and baseline BSCVA as pre-specified covariates. This controls for the baseline BSCVA. The treatment group is the primary predictor. Primary analysis will exclude mixed fungal and bacterial infection (isolated in the baseline sample).

We will use our alpha of 0.0492 to test the null hypotheses at 0.0492 significance. The null hypothesis for non-inferiority is that the mean BSCVA at three months for chlorhexidine is greater than or equal to 0.15 logMAR worse than the BSCVA when natamycin is used. Significance will be assessed using a two-tailed test at 0.0492 level for assessing non-inferiority. CHX will be non-inferior if the upper one-sided 95% confidence level for this regression coefficient (i.e. the effect of CHX controlling for baseline BSCVA) exceeds 0.15 logMAR.


For peer review only - http://bmjopen.bmj.com/site/about/guidelines.xhtml

***Primary Outcome Analysis – adjusted analysis:*** In the event that there is a baseline imbalance between the treatment groups in a baseline covariate due to chance, we will perform an adjusted (sensitivity) analysis (see below). This is particularly important if CHX has a better outcome than NATA, as the adjusted treatment effects may account for this observed imbalance whilst the unadjusted analyses may not. Sensitivity analyses will allow us to show that any observed positive treatment effect is not solely explained by imbalances at baseline in any of the covariates.

***Secondary Analyses of the Primary Outcome***

Per-protocol analysis

Repeat analysis of the primary outcome will be done as per the protocol, based on what the participants actually took. The per-protocol population will include all the individuals included in the primary intention-to-treat (ITT) analysis, excluding individuals who showed poor compliance with the medications (defined as taking less than 50% through self-reporting or bottle weighing, whichever is the lower); individuals where there have been major protocol deviations; and non-fungal or mixed corneal infections (should these patients happened to have been randomised). All analyses that are performed as ITT will be repeated as per-protocol and labelled as such.

Mixed infections

Secondary analysis of the primary outcome (by intention to treat) will include mixed infections and will be carried out in the same way as in primary analysis of the primary outcome above.

Sensitivity analyses

For the primary analysis those individuals with missing outcome data (i.e. lost to follow up) will be excluded. Sensitivity analyses will be performed by imputing a range of scenarios to demonstrate a range of potential results, where there is missing outcome data. In the case of substantial missing data in the trial, the primary analysis will be carried out as previously stated excluding missing observations. This however assumes data are missing completely at random. As a sensitivity analysis, we will then apply a multiple imputation approach to the missing data, if we consider the data is randomly missing conditional on other observed covariates.

In the case that there is a systematic (i.e. non-random) reason for a difference in the follow-up rates between the two groups, we will explore models in which these missing outcome data are assumed to be non-random i.e. dependent on the outcome being regressed, BSCVA or treatment group.



For peer review only - http://bmjopen.bmj.com/site/about/guidelines.xhtml

We will also perform sensitivity analyses on patients whose vision is CF or worse, those patients who have undergone a TPK or those with corneal perforation, to see if there is any change in the effect size or conclusions drawn.

Analysis of Other Potential Determinants for Success

Logistic regression random effects models will be used to analyse potential factors that may be associated with a poor primary outcome, BSCVA at 3 months, defined as >1.0 logMAR. Individual baseline characteristics will be used separately as an exposure variable with BSCVA at 3 months as the outcome, with the model adjusted for trial arm. A multivariate model will be built using parameters with a p-value of < 0.2 in the log likelihood ratio test. Variables will be removed one by one, by omitting the variable with the largest p-value each time, until all predictors in the model have a p<0.05.

***Secondary Outcome Analysis:*** The secondary outcomes outlined above and detailed in Table 4 will be analysed by arm. Additional adjustment for factors imbalanced between arms at baseline will be introduced as appropriate. Continuous outcomes will be analysed using linear regression. Binary and ordinal outcomes will analysed using logistic regression. Details of these are given in Table 4.

**Table 4: Secondary Outcome Measures that will be investigated as part of the trial, together with analysis details**

| Secondary outcome measure | Details |
|---|---|
| Three-week BSCVA | We will analyse the secondary outcome of three-week BSCVA in logMAR in the same manner as the primary analysis of the primary outcome described above. The three-week BSCVA will include values taken between 18 days and 5 weeks, with the value closest to 3 weeks used. |
| Presenting VA by Peek | We will analyse the presenting VA by Peek Acuity with and without pinhole at three months as a secondary outcome. This will be of interest if we are unable to get reliable BSCVA measurements at 3 months (i.e. if patients fail to attend and we need to attend their houses for visual acuity testing). This will be performed in the same way as the primary analysis of the primary outcome. We will also perform a sensitivity analysis including those lost to follow-up, by using the most recent observation of this variable. |
| Scar/infiltrate size at 1 week, 3 weeks and 3 months by slit lamp examination. | The geometric mean of the two principle axes in mm of the scar or infiltrate at 1 week, 3 weeks and 3 months will be used as a secondary outcome variable. The slit-lamp scar size will be compared at each of these time points between treatment arm in the same manner as described above. This will be by linear regression, with treatment arm and baseline infiltrate / scar size as pre-specified covariates. This controls for the baseline infiltrate / scar size. |
| Time to full epithelial healing (slit lamp examination by ophthalmic clinician) | Time of re-epithelialisation will be defined as the midpoint between the last review where an epithelial defect (ED) was present and the subsequent review where there was no ED. An area of fluorescein staining of less than 0.5mm will be considered as a resolved ED due to the difficulty in differentiating a smaller defect from a small amount of fluorescein pooling observed in a healed defect.<br><br>Analysis of time to healing will use Cox proportional hazards regression with treatment group as the primary predictor and with predictors of baseline ED size (using the geometric mean in mm as outlined above). Survival curves will be plotted |



For peer review only - http://bmjopen.bmj.com/site/about/guidelines.xhtml

| | |
|---|---|
| | using Kaplan-Meier analysis for both treatment arms up to the final visit at three months. The proportional hazards assumption will be checked by stratifying on quartiles of the baseline ED size and if the assumption does not hold, the stratified results will be the ones reported. Additionally, treatment failure (defined as persisting epithelial defect greater than 0.5mm at the three-month review) will be compared between treatment groups using Fisher's exact test. |
| Rate of healing | We will assess how quickly the area of ulceration reduces over time. The rate will be calculated between the 1 week, 3 week and 3-month review by taking the difference in ED size between the two time points, in mm, and diving by the number of days to give a rate of mm/day. Analysis will be performed using Cox regression. |
| Microbiological cure | Patients who have a persisting corneal ulcer (as defined by the presence of an ED) at day 7 will undergo a repeat corneal scrape and microbiological investigations. Microbiological cure at 7 days will be defined as the absence of any micro-organisms as no significant growth on culture. The number of patients with microbiological cure at day 7 will be compared between the two treatment arms using logistic regression with treatment group and organism (Aspergillus spp., Fusarium spp., or other) as covariates. |
| Ulcer depth at 1 week and 3 weeks (slit lamp examination by ophthalmic clinician). | The depth of ulcer in terms of percentage of healthy cornea will be compared at 1 week and 3 weeks between treatment arms, adjusting for baseline depth in the same manner with analysis performed by linear regression |
| Hypopyon height at 1 and 3 weeks, (slit lamp examination by ophthalmic clinician) | The hypopyon height in mm will be compared at 1 week and 3 weeks between treatment arms, adjusting for baseline hypopyon height in the same manner with analysis performed in the same way (linear regression) |
| Perforation and/or TPK and/or conjunctival advancement by three months (slit lamp examination by ophthalmic clinician) | The number of patients who undergo perforation and/or require TPK and/or have undergone conjunctival advancement by three months will be reported using confidence intervals and descriptive statistics. The study is not powered to detect a difference in perforation rate or TPK between treatment groups; not reporting a significant difference may be wrongly interpreted as there being no difference between groups. We will therefore perform an exploratory analysis to compare TPK or perforation rates between treatment groups. This will be by logistic regression to compute an odds ratio by arm. |
| Loss of Eye | The number of patients who have their eye surgically removed (evisceration or enucleation) during the 3 month follow-up period will be reported using confidence intervals and descriptive statistics in the same way as for TPK / perforation rate above, along with exploratory analysis using logistic regression to find risk factors for eye loss and odds ratio for this by arm. |
| Ocular adverse effects, slit lamp examination by ophthalmic clinician. | The proportion of patients with one or more adverse events will be compared using Fisher's Exact test. Additional analysis to compare the rate of adverse events during the three month follow-up will be by Poisson regression as this can take into account multiple instances within one participant. |
| Quality of life assessed using: EQ-5D, WHO/PBD-VF20, WHOQOL-BREF | QoL can be assessed quantitatively using different tools depending on what is of interest. For example, disease-related QoL can be assessed (e.g. vision related QoL, VRQoL) or more general health-related issues irrespective of the disease can be investigated (health-related QoL, HRQoL).[38]<br><br>We will use the WHO/PBD-VF20 (World Health Organisation/ Prevention of Blindness and Deafness—Visual Functioning 20 item questionnaire) VRQoL tool. This tool measures the impact of visual impairment in the person's life including mental wellbeing, dependency and social functioning. These have been used in a number of other visual related studies to show a difference in QoL. [31,32]<br><br>For HRQoL, we will use the EQ-5D questionnaire, EQ-Visual Analogue Scale and the WHOQOL-BREF. The EQ-5D is a standardised tool to measure health outcomes.[33] The WHOQOL-BREF has good applicability in (LMIC as it was developed simultaneously from concept across 18 countries in Africa, Asia and Latin America. It measures 4 domains of health: Physical Health, Psychological Health, Social Relationships, and Environment. |

For peer review only

| | |
|---|---|
| | Analysis will be by comparing the scores obtained for each QoL assessment for the two treatment arms to estimate the effect of CHX and NATA on patients' quality of life. This will be similar to that performed by Habtamu et al. [38] Comparisons between the two medication groups will be adjusted for the matching variables: age and sex. The VRQoL analysis was also adjusted for socio-economic status and the HRQoL analysis adjusted for both socio-economic status and presence of health problems during the previous four weeks, as these factors may confound the association between microbial keratitis and QoL. Logistic, linear and ordinal logistic regression methods will be used for binary, continuous and ordered categorical outcome variable analysis, respectively. Linear regression models and the t-test were employed to compare significant differences in QoL scores and to generate mean and mean differences between the two treatment arms in each QoL subscale and domain, respectively. |
| Cost effectiveness analysis, using EQ-5D data from 3 months and direct cost data | Direct cost data will be collected at the three-month follow-up. Economic costs to the patient can also be calculated from the EQ-5D questionnaire, which will be asked at baseline and at the three-month follow-up. Mean direct costs incurred by patients will be compared between interventional arms using the t-test for significance. The difference from the baseline EQ-5D and the three-month EQ-5D mean scores will also be compared in a similar fashion. |
| Drug adherence | The rate of drug adherence will be compared between the two treatment groups using descriptive statistics. |

***Interim Analysis:*** An interim analysis will be conducted for the DSMB by an independent statistician after 1/3 of patients recruited have completed follow-up.

**Patient and public involvement**

Mixed methods descriptive cross-sectional studies with semi-structured interviews and focus group discussions with patients and eye health providers were carried out in Sagarmatha zone, Eastern Nepal at various points in 2018.[39] These conversations highlighted the delayed presentation often seen with microbial keratitis combined with the often prohibitively high costs of treatment. Furthermore, treatment is not always felt to be effective. Eye health workers were keen to receive further training and highlighted the need for greater government support in the provision of eye care services in the community.

**ETHICS AND DISSEMINATION**

Ethics committee and regulatory review and approval has been obtained from the Nepal Health Research Council (NHRC) Ethics Committee, Kathmandu, Nepal; the Department of Drug Administration (DDA), Kathmandu, Nepal; and the London School of Hygiene and Tropical Medicine Ethics Committee, UK. The trial is registered with the ISRCTN clinical trials registry (ISRCTN14332621). Protocol modifications are submitted to the relevant parties for review and/or approval. Table 5 summarises the study protocol and trial registration information. At the end of the study period, patients who still require treatment or follow-up will continue to be treated at SCEH as per routine clinical practice. The trial Sponsor is the London School of Hygiene and Tropical Medicine. The results of this trial will be presented at local and international meetings and submitted to peer-reviewed journals for publication.

For peer review only

**Table 5: Registration Data and Protocol Summary**

| Data category | Information |
|---|---|
| Primary registry and trial identifying number | ISRCTN Registry; ISRCTN14332621 |
| Date of registration in primary registry | 15 May 2019 |
| Secondary identifying numbers | |
| Source(s) of monetary or material support | Wellcome Trust |
| Primary sponsor | London School of Hygiene and Tropical Medicine (LSHTM) |
| Secondary sponsor(s) | |
| Contact for queries | Jeremy Hoffman FRCOphth (Jeremy.hoffman@lshtm.ac.uk) |
| Title | Chlorhexidine 0.2% vs Natamycin 5% for the treatment of fungal corneal infections |
| Countries of recruitment | Nepal |
| Health condition(s) or problem(s) studied | Fungal keratitis |
| Intervention(s) | Participants will be randomised to either topical chlorhexidine 0.2% or topical natamycin 5% |
| Key eligibility criteria | 1. Acute MK characterised by:<br>• Corneal epithelial ulceration >1mm diameter<br>• Corneal stromal infiltrate<br>• Acute inflammation: e.g. conjunctival injection, anterior chamber inflammatory cells, hypopyon.<br>2. Filamentous fungal hyphae visualised on smear microscopy and/or *in vivo* confocal microscopy.<br>3. Agree to be randomised to either treatment arm and able to give informed consent<br>4. Agree to be followed up at 2 days, 1 week, 2 weeks, 3 weeks, 2 months and 3 months<br>5. Adults (18 years and older) |
| Study type | Randomised controlled trial |
| Date of first enrolment | 1 June 2019 |
| Target sample size | 500 |
| Recruitment status | Recruiting |
| Primary outcome(s) | Best Spectacle Corrected Visual Acuity (BSCVA) at 3 months by a trial certified optometrist |
| Key secondary outcomes | 1. Time to full epithelial healing (slit lamp examination by ophthalmic clinician).<br>2. Pin-hole visual acuity in logMAR at 3 months, trial-certified optometrist<br>3. Scar/infiltrate size at 1 week, 3 weeks and 3 months (slit lamp examination by ophthalmic clinician).<br>4. Ulcer depth at 1 week and 3 weeks (slit lamp examination by ophthalmic clinician).<br>5. Hypopyon height at 1 and 3 weeks, (slit lamp examination by ophthalmic clinician).<br>6. Perforation and/or TPK by three months (slit lamp examination by ophthalmic clinician).<br>7. Positive culture rate at 1 week<br>8. Ocular adverse effects at each follow up visit (Day 2, Day 7, Day 14, 3 weeks, 2 months, 3 months), slit lamp examination by ophthalmic clinician.<br>9. Quality of life (QoL) assessed using: EQ-5D, WHO/PBD-VF20, WHOQOL-BREF (comparison between baseline and QoL measures at 3 months)<br>10. Cost-effectiveness analysis, using EQ-5D data from 3 months and direct cost data.<br>11. Drug adherence at each follow up visit (Day 2, Day 7, Day 14, 3 weeks, 2 months, 3 months) whilst the patient is using study medications. |



For peer review only - http://bmjopen.bmj.com/site/about/guidelines.xhtml

**CONCLUSION**

This is the first large-scale randomised controlled clinical trial comparing topical chlorhexidine 0.2% to natamycin 5% for the treatment of fungal keratitis. It benefits from a pragmatic design: as a non-inferiority designed trial, if chlorhexidine is found to be within a pre-defined non-inferiority margin of 0.15 logMAR of natamycin at 3 months a recommendation to use chlorhexidine 0.2% can be made. This is a far cheaper, easy to formulate medication that could significantly increase access to anti-fungal treatment for the population most in need.

For peer review only



For peer review only - http://bmjopen.bmj.com/site/about/guidelines.xhtml

**ACKNOWLEDGEMENTS**

The authors would like to thank the Eastern Region Eye Care Programme (EREC-P), Nepal Netra Jyoti Sangh (NNJS) and the Nepal Health Research Council (NHRC) for helping with study co-ordination and implementation. The authors would like to thank the staff and management board at Sagarmatha Choudhary Eye Hospital (SCEH) for their continued support, co-ordination and implementation of the study. The authors are grateful to the guidance of the Data Safety Monitoring Board including Reeta Gurung (chair), Salma KC Rai, Sabina Shrestha and Meenu Chaudhary.

**COLLABORATORS**

SCEH:

Abhishek Roshan (Hospital Manager); Sanjay Kumar Singh (Medical Superintendent); Reena Yadav (Primary Investigator); Sandip Das Sanyam (Study Co-Ordinator); Pankaj Chaudhary (Microbiologist); Rabi Shankar Sah, Kamlesh Yadav (Investigators); Ram Narayan Bhandari, Aasha Chaudhary, Sharban Mandal (Eye Health Workers); Raja Ram Mahato (Randomisation Administrator and Logistics); Lalita Rajbanshi (Laboratory Assistant); Ramesh Sah, Arvind Ray, Sachindra Kamti (Optometrists); Avinash Chaudhary (Ophthalmic Assistant); Padma Narayan Chaudhary (Hospital Chairman); Suresh Singh, Ravi Pant, Rakesh Singh (Hospital Management); Ram Kumar Jha (Ophthalmic Assistant, Rajbiraj ECC)

NNJS:

Sailesh Kumar Mishra (Executive Director); Sabita KC (Board Secretary); Ranjan Shah (Programme Associate); Jaganath Dhital (Assistant)

EREC-P:

Sanjay Kumar Singh (Programme Director)

JEH:

Hemchandra Jha (Medical Superintendent); Mahesh Yadav (Investigator); Rudal Prasad Sah (Ophthalmic Assistant)

LSHTM:

Jeremy Hoffman (Primary Investigator); Matthew Burton (Chief Investigator); Astrid Leck (Microbiologist); David Macleod, Helen Weiss (Statisticians); Victor Hu (Investigator); Sarah O'Regan (Administrator)

For peer review only

**FUNDING STATEMENT**

This research was funded through a Senior Research Fellowship to MJB from the Wellcome Trust (207472/Z/17/Z). The funders had no role in study design, data collection and analysis, decision to publish, or preparation of the manuscript.

**COMPETING INTERESTS STATEMENT**

All authors declare there are no conflict of interests.

**DATA STATEMENT**

The datasets generated during and/or analysed during the current study will be available upon request from Prof Matthew Burton (matthew.burton@lshtm.ac.uk). The full data set will be available with all patient identifiable details removed. Data will be available after formal reporting of the study findings in a peer-reviewed scientific publication. Datasets will only be available to bona fide scientific investigators. Requests should be made to the Chief Investigator in writing detailing the scientific investigators background and intended use for the data. Consideration will be given to all proposed analyses, with likely envisaged uses including investigators planning on conducting meta-analyses for example. Patient Information Sheets and consent forms specifically referenced making anonymised data available and this has been approved by the relevant ethic committees.

**AUTHOR CONTRIBUTIONS**

Searched the literature: JJH, MJB

Drafted initial protocol: JJH

Contributed to protocol development and revision: JJH, RY, SD, PC, AR, SKS, SA, EM, DM, HAW, AL, VHH, MJB

Drafted this manuscript: JJH

Critically revised this manuscript: JJH, RY, SD, PC, AR, SKS, SA, EM, DM, HAW, AL, VHH, MJB

Conceptualization: MJB

Funding acquisition: MJB

For peer review only

**APPENDICES:**

Appendix 1 - Informed consent material

Appendix 2 – Monitoring for Harm

Appendix 3 - Biological specimens

Appendix 4 – SPIRIT Checklist

For peer review only

**LEGENDS FOR FIGURES**

**Figure 1: Fungal keratitis and corneal scarring.** (A) Active fungal keratitis with signs of acute inflammation and corneal ulceration. Photograph taken at presentation to SCEH. (B) Corneal Scar, the blinding sequela of a resolved episode of microbial keratitis. Photograph taken at two months following presentation (same patient as (A)).

**Figure 2: Progressive fungal keratitis.** (A) Early filamentous fungal keratitis; started immediately on intensive topical antifungal treatment (Natamycin 5%). (B) The same case one week later, unresponsive to intense Natamycin 5% treatment, with progression of the infection.

**Figure 3: Overview of the clinical trial.** Microbial keratitis is defined as presence of corneal epithelial ulceration (>1mm in diameter), corneal stromal infiltrate and signs of acute inflammation (e.g. conjunctival injection, anterior chamber inflammatory cells, hypopyon). Fungal elements to be detected by smear microscopy and/or confocal microscopy. Those eligible will be randomised 1:1 to CHX or NATA (n = 500). BSCVA: Best Spectacle Corrected Visual Acuity; TPK: Therapeutic Penetrating Keratoplasty.

For peer review only



For peer review only - http://bmjopen.bmj.com/site/about/guidelines.xhtml

## REFERENCES


1 Burton MJ, Pithuwa J, Okello E, *et al.* Microbial Keratitis in East Africa: Why are the Outcomes so Poor? *Ophthalmic Epidemiol* 2011;**18**:158–63. doi:10.3109/09286586.2011.595041

2 Thomas PA, Leck AK, Myatt M. Characteristic clinical features as an aid to the diagnosis of suppurative keratitis caused by filamentous fungi. *British Journal of Ophthalmology* 2005;**89**:1554–8. doi:10.1136/bjo.2005.076315

3 Leck AK, Thomas PA, Hagan M, *et al.* Aetiology of suppurative corneal ulcers in Ghana and south India, and epidemiology of fungal keratitis. *British Journal of Ophthalmology* 2002;**86**:1211–5. doi:10.1136/bjo.86.11.1211

4 Bongomin F, Gago S, Oladele RO, *et al.* Global and Multi-National Prevalence of Fungal Diseases-Estimate Precision. *J Fungi (Basel)* 2017;**3**:57. doi:10.3390/jof3040057

5 Ali Shah SI, Shah SA, Rai P, *et al.* Visual outcome in patients of keratomycosis, at a tertiary care centre in Larkana, Pakistan. *J Pak Med Assoc* 2017;**67**:1035–8.

6 Prajna NV, Krishnan T, Mascarenhas J, *et al.* The mycotic ulcer treatment trial: a randomized trial comparing natamycin vs voriconazole. *JAMA Ophthalmol* 2013;**131**:422–9.

7 Prajna NV, Krishnan T, Rajaraman R, *et al.* Effect of Oral Voriconazole on Fungal Keratitis in the Mycotic Ulcer Treatment Trial II (MUTT II): A Randomized Clinical Trial. *JAMA Ophthalmol* 2016;**134**:1365–72. doi:10.1001/jamaophthalmol.2016.4096

8 Galarreta DJ, Tuft SJ, Ramsay A, *et al.* Fungal keratitis in London: microbiological and clinical evaluation. *Cornea* 2007;**26**:1082–6. doi:10.1097/ICO.0b013e318142bff3

9 Ong HS, Fung SSM, Macleod D, *et al.* Altered Patterns of Fungal Keratitis at a London Ophthalmic Referral Hospital: An Eight-Year Retrospective Observational Study. *Am J Ophthalmol* 2016;**168**:227–36. doi:10.1016/j.ajo.2016.05.021

10 FlorCruz NV, Evans JR. Medical interventions for fungal keratitis. *Cochrane Database Syst Rev* 2015;**4**:CD004241. doi:10.1002/14651858.CD004241.pub4

11 Schein OD. Evidence-Based Treatment of Fungal Keratitis. *JAMA Ophthalmol* 2016;**134**:1372–3. doi:10.1001/jamaophthalmol.2016.4167

12 Arora R, Gupta D, Goyal J, *et al.* Voriconazole versus natamycin as primary treatment in fungal corneal ulcers. *Clin Experiment Ophthalmol* 2011;**39**:434–40. doi:10.1111/j.1442-9071.2010.02473.x

13 Prajna NV, Mascarenhas J, Krishnan T, *et al.* Comparison of natamycin and voriconazole for the treatment of fungal keratitis. *Arch Ophthalmol* 2010;**128**:672–8. doi:10.1001/archophthalmol.2010.102

14 Sharma S, Das S, Virdi A, *et al.* Re-appraisal of topical 1% voriconazole and 5% natamycin in the treatment of fungal keratitis in a randomised trial. *Br J Ophthalmol* 2015;**99**:1190–5. doi:10.1136/bjophthalmol-2014-306485

15 McDonnell G, Russell AD. Antiseptics and disinfectants: activity, action, and resistance. *Clinical Microbiology Reviews* 1999;**12**:147–79.


For peer review only

16 Shariff JA, Lee KC, Leyton A, *et al.* Neonatal mortality and topical application of chlorhexidine on umbilical cord stump: a meta-analysis of randomized control trials. *Public Health* 2016;**139**:27–35. doi:10.1016/j.puhe.2016.05.006

17 Zhou J, Hu B, Liu Y, *et al.* The efficacy of intra-alveolar 0.2% chlorhexidine gel on alveolar osteitis: a meta-analysis. *Oral Dis* 2017;**23**:598–608. doi:10.1111/odi.12553

18 Rahman MR, Johnson GJ, Husain R, *et al.* Randomised trial of 0.2% chlorhexidine gluconate and 2.5% natamycin for fungal keratitis in Bangladesh. *British Journal of Ophthalmology* 1998;**82**:919–25. doi:10.1136/bjo.82.8.919

19 Rahman MR, Minassian DC, Srinivasan M, *et al.* Trial of chlorhexidine gluconate for fungal corneal ulcers. *Ophthalmic Epidemiol* 1997;**4**:141–9. doi:10.3109/09286589709115721

20 Dart JKG, Saw VPJ, Kilvington S. Acanthamoeba keratitis: diagnosis and treatment update 2009. *Am J Ophthalmol* 2009;**148**:487–499.e2. doi:10.1016/j.ajo.2009.06.009

21 Kosrirukvongs P, Wanachiwanawin D, Visvesvara GS. Treatment of acanthamoeba keratitis with chlorhexidine. *Ophthalmology* 1999;**106**:798–802. doi:10.1016/S0161-6420(99)90169-0

22 Seal D, Hay J, Kirkness C, *et al.* Successful medical therapy of Acanthamoeba keratitis with topical chlorhexidine and propamidine. *Eye* 1996;**10 ( Pt 4)**:413–21. doi:10.1038/eye.1996.92

23 Martin MJ, Rahman MR, Johnson GJ, *et al.* Mycotic keratitis: susceptibility to antiseptic agents. *Int Ophthalmol* 1995;**19**:299–302.

24 Geffen N, Norman G, Kheradiya NS, *et al.* Chlorhexidine gluconate 0.02% as adjunct to primary treatment for corneal bacterial ulcers. *Isr Med Assoc J* 2009;**11**:664–8.

25 Upadhyay MP, Karmacharya PC, Koirala S, *et al.* The Bhaktapur eye study: ocular trauma and antibiotic prophylaxis for the prevention of corneal ulceration in Nepal. *British Journal of Ophthalmology* 2001;**85**:388–92.

26 Habtamu E, Bastawrous A, Bolster NM, *et al.* Development and Validation of a Smartphone-based Contrast Sensitivity Test. *Transl Vis Sci Technol* 2019;**8**:13. doi:10.1167/tvst.8.5.13

27 Chidambaram JD, Prajna NV, Larke N, *et al.* In vivo confocal microscopy appearance of Fusarium and Aspergillus species in fungal keratitis. *Br J Ophthalmol* 2017;**101**:1119–23. doi:10.1136/bjophthalmol-2016-309656

28 Chidambaram JD, Prajna NV, Larke NL, *et al.* Prospective Study of the Diagnostic Accuracy of the In Vivo Laser Scanning Confocal Microscope for Severe Microbial Keratitis. *Ophthalmology* 2016;**123**:2285–93. doi:10.1016/j.ophtha.2016.07.009

29 Somannavar S, Ganesan A, Deepa M, *et al.* Random capillary blood glucose cut points for diabetes and pre-diabetes derived from community-based opportunistic screening in India. *Diabetes Care* 2009;**32**:641–3. doi:10.2337/dc08-0403

30 World Health Organization. Prevention of Blindness & Deafness. Consultation on Development of Standards for Characterization of Vision Loss and Visual Functioning. In: *WHO / PBL*. Geneva: 2003.

31 Polack S, Kuper H, Mathenge W, *et al.* Cataract visual impairment and quality of life in a Kenyan population. *British Journal of Ophthalmology* 2007;**91**:927–32. doi:10.1136/bjo.2006.110973

For peer review only

32 Polack S, Kuper H, Wadud Z, *et al.* Quality of life and visual impairment from cataract in Satkhira district, Bangladesh. *Br J Ophthalmol* 2008;**92**:1026–30. doi:10.1136/bjo.2007.134791

33 EuroQol Research Foundation. EQ-5D-3L User Guide. 2018. https://euroqol.org/publications/user-guides

34 Bastawrous A, Rono HK, Livingstone IAT, *et al.* Development and Validation of a Smartphone-Based Visual Acuity Test (Peek Acuity) for Clinical Practice and Community-Based Fieldwork. *JAMA Ophthalmol* 2015;**133**:930–8. doi:10.1001/jamaophthalmol.2015.1468

35 Barron BA, Gee L, Hauck WW, *et al.* Herpetic Eye Disease Study. A controlled trial of oral acyclovir for herpes simplex stromal keratitis. *Ophthalmology* 1994;**101**:1871–82.

36 Wilhelmus KR, Beck RW, Moke PS, *et al.* Acyclovir for the prevention of recurrent herpes simplex virus eye disease. Herpetic Eye Disease Study Group. *N Engl J Med* 1998;**339**:300–6. doi:10.1056/NEJM199807303390503

37 Schulz KF, Altman DG, Moher D, *et al.* CONSORT 2010 statement: updated guidelines for reporting parallel group randomised trials. BMJ. 2010;**340**:c332–2. doi:10.1136/bmj.c332

38 Habtamu E, Wondie T, Aweke S, *et al.* The Impact of Trachomatous Trichiasis on Quality of Life: A Case Control Study. *PLoS Negl Trop Dis* 2015;**9**:e0004254. doi:10.1371/journal.pntd.0004254

39 Burn H, Puri L, Roshan A, *et al.* Primary Eye Care in Eastern Nepal. *Ophthalmic Epidemiol* 2019;**22**:1–12. doi:10.1080/09286586.2019.1702217

For peer review only

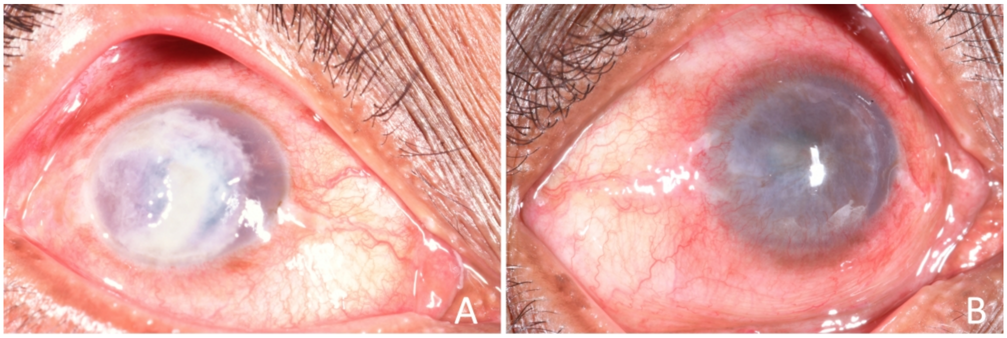


Figure 1: Fungal keratitis and corneal scarring. (A) Active fungal keratitis with signs of acute inflammation and corneal ulceration. Photograph taken at presentation to SCEH. (B) Corneal Scar, the blinding sequela of a resolved episode of microbial keratitis. Photograph taken at two months following presentation (same patient as (A)).

325x107mm (300 x 300 DPI)

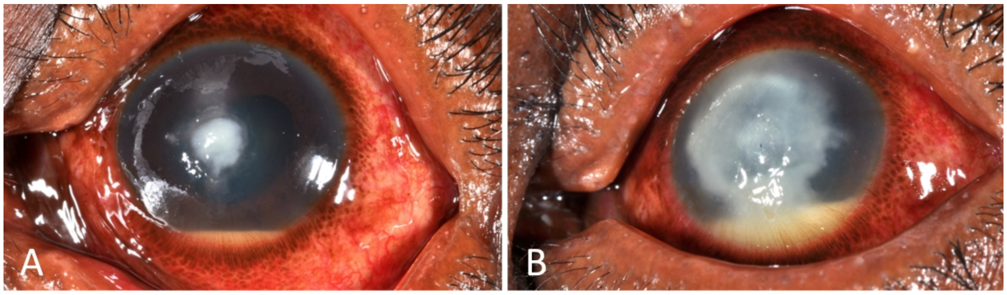


Figure 2: Progressive fungal keratitis. (A) Early filamentous fungal keratitis; started immediately on intensive topical antifungal treatment (Natamycin 5%). (B) The same case one week later, unresponsive to intense Natamycin 5% treatment, with progression of the infection.

325x93mm (300 x 300 DPI)

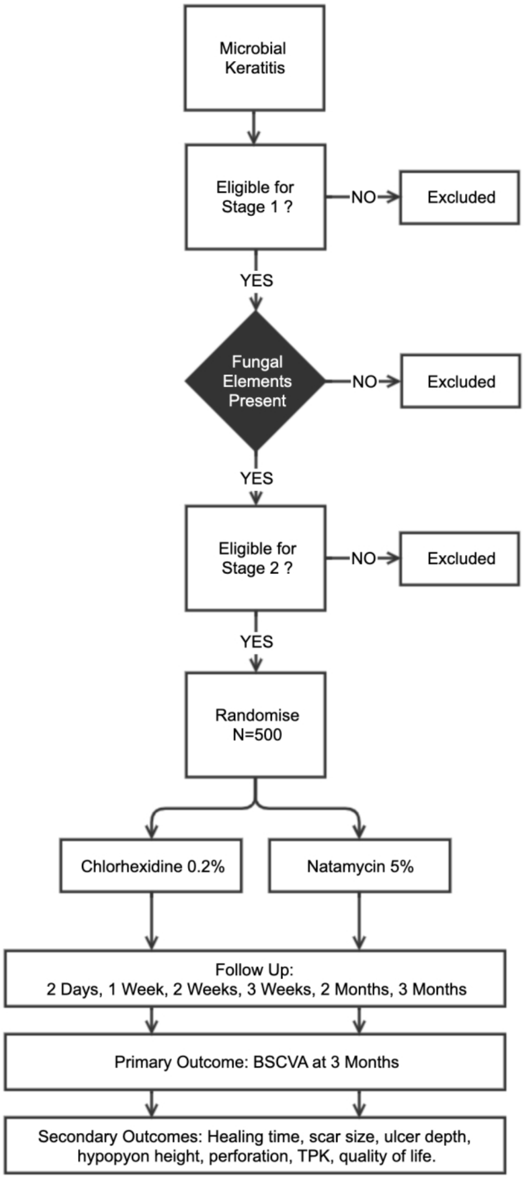


Figure 3: Overview of the clinical trial. Microbial keratitis is defined as presence of corneal epithelial ulceration (>1mm in diameter), corneal stromal infiltrate and signs ofacute inflammation (e.g. conjunctival injection, anterior chamberinflammatory cells, hypopyon). Fungal elements to be detected by smear microscopy and/or confocal microscopy. Those eligible will be randomised 1:1 to CHX or NATA (n = 500). BSCVA: Best Spectacle Corrected Visual Acuity; TPK: Therapeutic Penetrating Keratoplasty.

87x182mm (300 x 300 DPI)

# Appendix 1: Informed Consent Material

## Study Title: Randomised controlled trial of topical chlorhexidine 0.2% versus topical natamycin 5% for fungal keratitis in Nepal

*Participant Information Sheet and Consent Form - No 1:*
*For baseline assessment and investigations*

**Introduction**
You are being invited to take part in a medical research study. Before you decide it is important for you to understand why the research is being done and what it will involve. Please take time to read or listen to the following information carefully and to talk to others about the study, if you wish. Ask us if there is anything that is not clear or if you would like more information. Do not sign the consent form unless you are satisfied with the answers to your questions and decide that you want to be part of this study. Take time to decide whether or not you wish to take part.

**Why have I been invited?**
You have been invited to take part because you have a corneal infection. To guide the treatment of this it is helpful to do tests to find out the cause, to guide the choice of eye drop treatment. We are asking for your permission to carry out some tests that are listed below to find out what is causing the infection and to decide if you may be suitable for a trial comparing treatments for fungal eye infections.

**What is this study about?**
Infection of the clear part of the front of the eye (the cornea) is a corneal ulcer. It is an important cause of blindness in Nepal. A scratch in the cornea allows infection to enter and an ulcer to begin. These infections can be very serious with some people losing the sight in the affected eye.

Different types of infectious organisms can cause corneal ulcers. These include bacteria and fungi which need to be treated with different types of eye drop medicines. To find out what type of infection is present it is necessary to collect a small sample from the ulcer and perform various laboratory tests. The test results guide the treatment. In Nepal about half of the infections are caused by fungus.

Fungal infections can be difficult to diagnose. No single test can reliably detect it. It may need several different tests to find it. Currently, in many countries where fungal ulcers are common laboratory services are limited or not available. Tests are expensive and may not detect all of the infections. There is a need to understand which of the current test options give the most useful information.

The first purpose of this study is to carefully evaluate your eye infection using several different diagnostic tests to try to find out what type of infection you have, and to learn which tests are most useful in this process.

There is a need for alternative, affordable and more easily available eye drop treatments for fungal infections. We are also conducting a clinical trial to compare two eye drop treatments for fungal infection, to try to find out which is better.

Therefore, the second purpose of this initial Assessment and Investigation study is to identify people who have a fungal corneal infection who may be eligible and willing to enrol in the clinical trial. If we find out that you do have a fungal corneal infection, we will then ask if you would be interested in hearing about the clinical trial. If you are interested, then at that stage we will explain about the treatment trial in detail, provide you with a second information sheet and go throw a second consent process.

**Do I have to take part?**
No. Your involvement is entirely voluntary. If you agree to take part, we will then ask you to sign a consent form. If you decide to join and change your mind, you are free to withdraw at any time without giving a reason. This will not affect the standard of care you receive.
Why have I been invited?
You have been invited to take part because you have a corneal infection. To guide the treatment of this it is helpful to do tests to find out the cause, to guide the choice of eye drop treatment. We are asking for your permission to carry out some tests that are listed below to find out what is causing the infection and to decide if you may be suitable for a trial comparing treatments for fungal eye infections.

For peer review only

**What will happen to me if I take part?**
If you agree to be part of this study, the following will happen:

- We will ask you a series of questions. This will include basic demographic information, the history of your current eye problem, and treatment you have had before arriving at the hospital.
- We will then carefully examine both eyes using a special microscope.
- Your eyes will be photographed with a camera. This is additional to standard care, and will help us to monitor the infection and the response to treatment.
- We will use a special microscope to look at the cornea to see if we can find a fungal infection. This involves putting anaesthetic drops on the eye. A soft plastic device then gently touches the eye so to see if you have a fungal infection. This is additional to standard care, and will help us to find out the type of infection you have more rapidly so that we can offer you the most appropriate treatment.
- We will collect samples form the corneal ulcer to test in the laboratory to try to identify what is causing the infection. Anaesthetic eye drops will be used numb the eye so you will not feel anything while we collect the sample by brushing the surface.
-
- We will check your blood sugar level for diabetes. This is done using a finger-prick blood sample. If this is raised, we will refer you to a separate group of doctors to help you with this.
- You will be offered a test for HIV infection. This will be done through the hospitals counselling and testing services. If you choose to accept this, then you will be separately counselled about the test and the implications of the results. This would involve the collection of a blood test sample from your arm. If the HIV test is positive, then you will be referred to the appropriate team for ongoing care. The result of this test will be shared with the us (cornea infection study team), with your consent, as it is potentially relevant to the treatment of your cornea infection.
- We will collect a sample of the cells from the inside of your cheek by gently rubbing a swab for a few seconds. This is additional to the standard of care. The purpose is to try to understand how the body fights the infection and why some people develop this eye problem and others do not.
- You will be asked to complete three short questionnaires to assess your quality of life and vision function. This is additional to standard care, and will help us to better understand the impact corneal ulcers can have on people's lives.

If we find evidence of fungus infecting your eye, then we will discuss the details of the trial with you further. At that point we will give you a second information sheet to read (or read to you), and if you meet all the eligibility criteria for the trial, to then go through a second consent process with you.

If you do not have a fungal infection, for example if the infection is caused by bacteria, then your will receive standard ongoing treatment for this by the same team at the hospital.

You may be withdrawn from the study without your consent if the researchers believe it is in your best interest or if you fail to follow study procedures.

**What are the side effects or risks of taking part?**
- The anaesthetic eye drops usually sting for a few seconds and then your eye should feel more comfortable. There is a very low risk of allergic reaction from the anaesthetic eye drops.
- The procedures including the history taking, examination, photography, in vivo confocal microscopy and sample collection are part of the standard of care for assessing a corneal infection. The risk associated with these are very low and no different from routine care. To minimise discomfort, topical anaesthetic will be given before examinations and sample collection.
- You will experience a little pain when a blood sample is collected. The people collecting the blood sample will ensure that this is done with as little discomfort as possible.
- The questions, tests and examinations will add about 15 minutes to your hospital visit above what we would usually expect in assessing a patient with this problem.

**What are the benefits of taking part?**
- The study will involve tests for the type of infection. This helps the doctor looking after you to choose the best type of treatment for your eyes
- The costs for the clinical assessment and the tests will be paid for by the study.
- By participating in this study, you will be helping to further research into this field so more informed decisions can be made when treating people with corneal infection.

For peer review only

**What will happen to the clinical records, photographs and test results?**
Your records will remain strictly confidential at all times. The information will be held in a secure office at your treating hospital. Only the people organizing or supervising the trial and regulatory authority auditors will have access to it. These include officials delegated by the Sponsor (London School of Hygiene and Tropical Medicine), the local National Ethics Committee, The local National Drug Regulatory Authority and trial Data Safety Monitoring Body (DSMB).

Your name will not be passed to anyone else outside the research team, unless we have your direct instruction to do so, for example to make a medical referral.

The photographs of the eye and the result of the laboratory test for infection will be shared with computer engineers to help develop a programme that can automatically analyse the image to see if this can provide some indication of the cause of the infection. Images of corneal infection may also be used for educational and teaching purposes, including in publications. All personal identifying information will be removed before sharing images.

**What tests will we do on the sample?**
The samples collected from the surface of your eye will be tested in several different ways to determine what is causing the infection. This work will be done in the hospital microbiology laboratory, where you are being treated. A portion of the infection sample will be transferred for additional special tests at KCMC Hospital Biotechnology (Tanzania), the London School of Hygiene and Tropical Medicine (UK) and Radboud University Nijmegen Medical Center (The Netherlands), as some of the tests will require additional special equipment that is not available at all sites.

- We will look for the type of infection using a microscope and by growing the organisms in the laboratory. We will test the organisms that grow to see which medicines work best to kill the infection, which is helpful in guiding the choice of treatment to be used.

- The swab samples from the ulcer will be used to test for infection using molecular diagnostic tests and to evaluate new tests that may be used to find the cause rapidly in the clinic.

- We will use study genetic material of the organism causing the infection to find out the exact type of infection and its ability to resist treatments. Samples from the ulcer may also be used to investigate your immune response to the infection, so that we can better understand what causes corneal scarring

- We will store a sample of the infection causing organism indefinitely for additional testing.

- The genetic material from the cells from your cheek will be sent to the UK or to KCMC Hospital in Tanzania. We would like to store it until a later time when a sufficiently large number of samples has been collected to conduct an analysis of this genetic material. The purpose would be to try to better understand how the human immune system fights the infection and why some people develop this eye problem and others do not. By doing these tests we hope that it will help us to develop approaches that will help to prevent or improve outcomes from this condition.

- As part of this consent we are asking that you give us permission to store this material to be able to test it at a later date as mentioned above. We do not know exactly for how long we shall store the genetic material before we have assembled a sufficiently large collection during the course of several planned studies to be able to proceed to the sample analysis, however, we anticipate a period of at least five years.

**What will happen to the results of the research study?**
The results of the study will be available after it finishes and will be included in peer reviewed medical and scientific journals and may be presented at medical meetings. Results will also be published on a publicly accessible trials database. The data will be anonymous and none of the patients involved in the trial will be identified in any report or publication. Should you wish to see the results, or the publication, please ask your study doctor.

**Who is funding the research?**
The research is being funded as part of a grant from the Wellcome Trust, UK.

**Who is organising the research?**
It is being organised through a research partnership between the London School of Hygiene and Tropical Medicine and Sagarmatha Choudhary Eye Hospital in Nepal, Mbarara University of Science and Technology in Uganda and Kilimanjaro Christian Medical Centre in Tanzania.

**What if relevant new information becomes available?**
It is not anticipated that new information will become available during the course of this short study. The information from the microbiology tests will be available to the doctors to help select the most appropriate treatment for you.

For peer review only

**What if something goes wrong?**
If you have a concern about any aspect of this study, you should ask to speak to the researchers who will do their best to answer your questions. If you remain unhappy and wish to complain formally, you can do this through the head of the hospital eye department or the named person on the following page. The London School of Hygiene and Tropical Medicine holds insurance policies which apply to this study. If you experience harm or injury as a result of taking part in this study, you may be eligible to claim compensation.

**Who has reviewed the study?**
Prospective research such as this is looked at by an independent group of people, called a Research Ethics Committee, to protect your interests. This study has been reviewed and approved by (1) the London School of Hygiene and Tropical Medicine Research Ethics Committee; (2) Nepal Health Research Council.

**What will happen if I don't want to carry on with the study?**
Your participation in this study is entirely voluntary. You may refuse to participate or may withdraw from this study at any time without penalty or loss of any rights or benefits to which you are otherwise entitled. The study doctor may also stop your participation in the study at any time for safety reasons. If you decide to withdraw from the study you should contact a member of the study team immediately. You do not have to give a reason when stopping, however for safety reasons, it is suggested that you tell the study doctor if you decide to stop because of an unwanted side effect. If you withdraw from the study, we will only use data collected before this decision, unless you request this to also be withdrawn. If you withdraw from the study, researchers, authorized persons from the Sponsor and the regulatory authorities will still require access to your medical notes to verify the data collected up to the date of your withdrawal.

**Contact Details**
Nepal Study Site: Dr Reena Yadav, Sagarmatha Choudhary Eye Hospital, Lahan. T: +977 9807799958

Study Coordinator: Dr Jeremy Hoffman: email: Jeremy.hoffman@lshtm.ac.uk
Chief Investigator: Prof. Matthew Burton: email: matthew.burton@lshtm.ac.uk

**You will be given a copy of the information sheet and a signed consent form to keep.**
**Thank you for considering taking the time to read this sheet.**

For peer review only

**Randomised controlled trial of topical chlorhexidine 0.2% versus topical natamycin 5% for fungal keratitis**

## Consent Form No 1: Baseline assessment and investigations

**Participant Name**___________________________ **Study ID Number:** ____________________

| | **Please initial box** |
|---|---|
| 1. I confirm that I have read and understand the participant information sheet dated ……….. (version ………) for the above study. I have had the opportunity to consider the information, ask questions and have had these answered fully. | |
| 2. I understand that my participation is voluntary and I am free to withdraw at any time, without giving any reason, without my medical care or legal rights being affected. | |
| 3. I understand that sections of my medical notes and data collected during the study may be looked at by responsible individuals from the London School of Hygiene & Tropical Medicine, from national regulatory authorities or from this hospital, where it is relevant to my taking part in this research. I give permission for these individuals to access my records. | |
| 4. I agree to take part in the above study. | |
| 5. I agree to the collection, laboratory tests and storage for future analysis of the samples from the surface my eye infection to understand the disease as described above. | |
| 6. I agree to the collection and storage for future analysis of the samples from the inside of my mouth to investigate how human genes affect this disease. | |
| 7. I agree to having a blood sample collected to measure sugar levels | |
| 8. I agree to being referred for a HIV pre-test counselling, testing and for the results to be shared with the research team. | |
| 9. I agree for the photographs of the front of my eye to be used in the publication or report released on the study, and for teaching purposes, including on the internet. | |
| 10. I understand that data about/from me/the participant may be shared via a public data repository or by sharing directly with other researchers, and that I will not be identifiable from this information. | |

For peer review only

| Name of Participant *(printed)* | Signature/Thumbprint | Date |
|---|---|---|
| Name of Person taking consent | Signature | Date |

The participant is unable to sign. As a witness, I confirm that all the information about the study was given and the participant consented to taking part.

| Name of Impartial Witness *(if required)* | Signature | Date |
|---|---|---|

*1 copy for participant; 1 copy for Principal Investigator; 1 copy to be kept with hospital notes*

**Study Title: Randomised controlled trial of topical chlorhexidine 0.2% versus topical natamycin 5% for fungal keratitis in Nepal**

*Participant Information Sheet and Consent Form – No 2:*
*For enrolment into the clinical trial*

**Introduction**
Thank you for earlier participating in the first stage of the study. You are now being invited to take part in the clinical treatment trial part of this medical research study. Before you decide it is important for you to understand why the research is being done and what it will involve. Please take time to read or listen to the following information carefully and to talk to others about the study, if you wish. Ask us if there is anything that is not clear or if you would like more information. Do not sign the consent form unless you are satisfied with the answers to your questions and decide that you want to be part of this study. Take time to decide whether or not you wish to take part.

**Why have I been invited?**
You have been invited to take part because you have a corneal infection which we have found out from tests is caused by fungus.

**What is this study about?**
Infection of the clear part of the front of the eye (the cornea) is a corneal ulcer. It is an important cause of blindness in Nepal. A scratch in the cornea allows infection to enter and an ulcer to begin. These infections can be very serious with some people losing the sight in the affected eye.

Different types of infectious organisms can cause corneal ulcers. These include bacteria and fungi. In tropical regions about half of all corneal ulcers are caused by fungi. Bacteria and fungi need to be treated with different types of eye drop medicines.

Treatments for fungal eye infections are frequently not very effective, in addition access to these treatments in many countries is very limited and can be expensive. In some countries they are simply not available. Currently the most commonly used treatment for fungal corneal ulcers is an eye drop called Natamycin. There is a need for additional, alternative, affordable and more easily available eye drop treatments for fungal infections.

There is an antiseptic solution called Chlorhexidine. This is very effective at killing bacteria, fungi and other types of infectious organisms. It is used in medical care worldwide in several different ways. For example, it is used to clean skin before surgical operations, in antiseptic creams for skin cuts and as a mouth wash to prevent and treat mouth infections. It has been used in eye care for more than thirty years as an eye-drop preservative, for sterilizing contact lenses, for pre-operative topical antiseptic and for treating *Acanthamoeba* and fungal corneal infections.

About twenty years ago chlorhexidine eye drops were tested in two small clinical trials conducted in India and Bangladesh for the treatment of fungal corneal infections. The results of these studies suggested that chlorhexidine was as good as and possibly better than natamycin at controlling the infection. Neither eye drop had any serious side effect. However, the studies were not large enough to be certain.

Chlorhexidine is currently used in several countries for the treatment of fungal corneal infections when natamycin or alternative treatment is not working or is not available. We would like to conduct a large clinical to find out whether chlorhexidine 0.2% eye drops are as effective as or more effective than natamycin 5% eye drops for treating fungal corneal infections

**Do I have to take part?**
No. Your involvement is entirely voluntary. If you agree to take part, we will then ask you to sign a consent form. If you decide to join and change your mind, you are free to withdraw at any time without giving a reason. This will not affect the standard of care you receive. If you decide not to participate in the study, then you will be offered the standard treatment for fungal keratitis using natamycin.

**What will happen to me if I take part?**
If you agree to be part of this study, the following will happen:

***1) Baseline Assessment:***
As part of the initial assessment that has already taken place we have carefully examined yours eyes, taken photographs, performed a scan for infection and collected samples. The tests have found that you have a fungal infection in your cornea. The risks to an unborn or breast-fed baby from antifungal eye drops use are unknown. Therefore, pregnant and breastfeeding women are excluded from participating in this study. Pregnancy testing will be offered to potential female participants to confirm pregnancy status.

For peer review only

***2) Randomisation:***

We will randomly allocate you to one of the two treatment option: either chlorhexidine eye drops or natamycin eye drops. Sometimes we don't know which way of treating patients is best. To find out, we need to make comparisons between the different treatments. We put people into groups and give each group one of the two alternative treatments; the results are compared after some time to see if one is better. To try to make sure the groups are the same to start with, each patient is put into a group by chance (randomly). You have an equal chance of being put into the chlorhexidine or natamycin treatment group. Neither you nor the people examining your eyes will be told which treatment group you are in. It is important that neither you nor we know which of the two you are given. This information would be in our files, but we would not look at these files until after the research is finished. This is the best way we have for testing without being influenced by what we think might happen. We would then compare which of the two treatments has the best results.

***3) Treatment:***

Once you are allocated to one of the treatment groups, you will receive clear instruction on how to take the eye drops. For the first week we will ask you to take one drop every hour. For the second and the third weeks the frequency of the eye drops will be reduced to every other hour (2-hourly). After that the frequency and duration of treatment will depend on the severity of the infection and how it is responding. You will be given clear guidance on this by the eye doctor who is looking after you.

***4) Follow-up Assessment:***

Initially, most people with corneal infections stay in hospital for several days so that the clinical team can monitor the response of the infection to the treatment. For the purposes of the study, we would like to review the response to treatment and document the clinical findings at the following times after you start treatment: two days, 1 week, 2 weeks, 3 weeks, 2 months and 3 months.

On each occasion we will ask you a few questions about your eye and the treatment. We will measure your eye sight. We will examine the eye with a microscope and photograph it with a camera.

At 1 week, 2 weeks and 3 weeks we will repeat the *in vivo* confocal microscopy test that was done at your first assessment. This is done to see how the infection is responding to treatment. This involves putting anaesthetic drops on the eye so that you do not feel any discomfort. A soft plastic device then gently touches the surface of the eye so that we can take special photographs of the front of your eye ("scan")

At 1 week if you still have an open ulcer on the cornea we will repeat the sample collection to test for the ongoing presence of the infection. This involves first putting anaesthetic eye drops on the surface of the eye. Then gently scraping the surface of the corneal ulcer and testing for the presence of fungus and bacteria in the microbiology laboratory.

Sometimes fungal infections do not respond to the treatment. In such cases it may be necessary to alter the treatment or perform an operation. The eye doctors who will be looking after you will monitor your progress closely and advise you about further treatment might be needed.

At the three month review you will be asked to complete three short questionnaires to assess your quality of life and vision function. These are the same standardised questions used in the baseline assessment to try to understand the impact that different medical problems can have on people's lives.

You may be withdrawn from the study without your consent if the researchers believe it is in your best interest or if you fail to follow study procedures.

**What are the side effects or risks of taking part?**

1. **Random Allocation and Treatment Failure**: You will be randomly allocated to a treatment. The treatment you are allocated to may prove to be less effective or to have more side effects than the other study treatment or other available treatments.

   **It is important to recognise that corneal infection is a serious, sight threatening condition.** Many patients, whatever the treatment used, have reduced vision in the affected eye after it has resolved. In some people the affected eye will become blind. Sometimes the infection, despite lots of treatment, can progress to cause a hole to develop in the cornea (Perforation) and sometimes it is so severe it is necessary to perform an operation to remove the eye content.

2. **Local Irritation:** As with most eye drops, there is the risk of local irritation or stinging from either chlorhexidine or natamycin. This usually only lasts for a short time.

3. **Allergic Response**: Very rarely, either chlorhexidine or natamycin eye drops can provoke a local allergic reaction on the surface of the eye or the eyelids.

For peer review only

4. **Pregnancy and Breast Feeding:** The risks to an unborn or breast-fed baby from antifungal eye drops use are unknown. Therefore, pregnant and breastfeeding women are excluded from participating in this study.

5. **Natamycin 5% eye drops**: Natamycin is an approved antifungal medication that is currently being used for the treatment of fungal corneal ulcer. It is on the World Health Organisation Essential Medicines List for the treatment of fungal corneal infections. There are no known serious side effects with this medication. It may cause mild irritation and very rarely a local allergic response.

6. **Chlorhexidine 0.2% eye drops:** Chlorhexidine eye drops are used on the surface of the eye as an antiseptic before procedures and also in the treatment of fungal and other eye infections. It has not been associated we any serious side effects. It may cause mild irritation and very rarely a local allergic response. This concentration of chlorhexidine is approved to be used in much larger volumes as a mouth wash. It is considered to be safe and is not associated with any systemic side effects.

7. **Procedures:** including examinations, confocal microscopy, corneal sample collection and checking for the best glasses or contact lenses carry the same very small risk whether they are performed as part of this study or of usual care outside the study. To minimise discomfort, topical anaesthetic will be given before examinations and sample collection.

8. **Unknown Risks:** The treatments in this study may have rare side effects that are currently not known. If during the course of the study new information becomes available, the researchers will share this with.

**What are the possible benefits of taking part?**

- The study will involve tests for the type of infection. This helps the doctor looking after you to choose the best type of treatment for your eyes
- The costs for your clinical assessment, tests, treatment and transport will be paid for by the study.
- By participating in this study, you will be helping to answer the question about whether or not chlorhexidine is a suitable alternative treatment for fungal corneal infections.

**What will happen to the clinical records, photographs and test results?**

Your records will remain strictly confidential at all times. The information will be held in a secure office at your treating hospital. Only the people organizing or supervising the trial and regulatory authority auditors will have access to it. These include officials delegated by the Sponsor (London School of Hygiene and Tropical Medicine), the local National Ethics Committee, The local National Drug Regulatory Authority and trial Data Safety Monitoring Body (DSMB).

A study number rather than your name will be used on study records or the database wherever possible. Your name and other facts that might identify you will not appear when we present this study or publish its results.

Your name will not be passed to anyone else outside the research team, unless we have your direct instruction to do so, for example to make a medical referral.

Images of corneal infection may be used for educational and teaching purposes, including in publications. All personal identifying information will be removed before sharing images.

**What tests will we do on the sample?**

The samples collected from the surface of your eye will be tested in several different ways to determine what is causing the infection. This work will be done in the hospital microbiology laboratory, where you are being treated. A portion of the infection sample will be transferred for additional special tests at KCMC Hospital Biotechnology (Tanzania), the London School of Hygiene and Tropical Medicine (UK) and Radboud University Nijmegen Medical Center (The Netherlands).

We will look for the type of infection using a microscope and by growing the organisms in the laboratory. We will test the organisms that grow to see which medicines work best to kill the infection, which is helpful in guiding the choice of treatment to be used. The swab samples from the ulcer will be used to test for infection using molecular diagnostic tests and to evaluate new tests that may be used to find the cause rapidly in the clinic. We will use the genetic material of the organism causing the infection to sequence its genetic code, which helps us to find out the exact type of infection and its ability to resist treatments. We will store a sample of the infection causing organism indefinitely for additional testing.

**What will happen to the results of the research study?**

The results of the study will be available after it finishes and will be included in peer reviewed medical and scientific journals and may be presented at medical meetings. Results will also be published on a publicly accessible trials database. The data will be anonymous and none of the patients involved in the trial will be identified in any report or publication. Should you wish to see the results, or the publication, please ask your study doctor.

**Who is funding the research?**

For peer review only

The research is being funded as part of a grant from the Wellcome Trust, UK.

**Who is organising the research?**
It is being organised through a research partnership between the London School of Hygiene and Tropical Medicine and Sagarmatha Choudhary Eye Hospital in Nepal, Mbarara University of Science and Technology in Uganda and Kilimanjaro Christian Medical Centre in Tanzania.

**What if relevant new information becomes available?**
It is not anticipated that new information will become available during the course of this study. However, if it does, this will be shared with you by the researchers in case this affects whether you wish to continue in the study.

**What if something goes wrong?**
If you have a concern about any aspect of this study, you should ask to speak to the researchers who will do their best to answer your questions. If you remain unhappy and wish to complain formally, you can do this through the head of the hospital eye department or the named person on the following page. The London School of Hygiene and Tropical Medicine holds insurance policies which apply to this study. If you experience harm or injury as a result of taking part in this study, you may be eligible to claim compensation.

**Who has reviewed the study?**
Prospective research such as this is looked at by an independent group of people, called a Research Ethics Committee, to protect your interests. This study has been reviewed and approved by (1) the London School of Hygiene and Tropical Medicine Research Ethics Committee; (2) Nepal Health Research Council.

What will happen if I don't want to carry on with the study?
Your participation in this study is entirely voluntary. You may refuse to participate or may withdraw from this study at any time without penalty or loss of any rights or benefits to which you are otherwise entitled. The study doctor may also stop your participation in the study at any time for safety reasons. If you decide to withdraw from the study you should contact a member of the study team immediately. You do not have to give a reason when stopping, however for safety reasons, it is suggested that you tell the study doctor if you decide to stop because of an unwanted side effect. If you withdraw from the study, we will only use data collected before this decision, unless you request this to also be withdrawn. If you withdraw from the study, researchers, authorized persons from the Sponsor and the regulatory authorities will still require access to your medical notes to verify the data collected up to the date of your withdrawal.

**Contact Details**
Nepal Study Site: Dr Reena Yadav, Sagarmatha Choudhary Eye Hospital, Lahan. T: +977 9807799958

Study Coordinator: Dr Jeremy Hoffman: email: Jeremy.hoffman@lshtm.ac.uk
Chief Investigator: Prof. Matthew Burton: email: matthew.burton@lshtm.ac.uk

**You will be given a copy of the information sheet and a signed consent form to keep.**
**Thank you for considering taking the time to read this sheet.**

For peer review only

**Randomised controlled trial of topical chlorhexidine 0.2% versus topical natamycin 5% for fungal keratitis**

**Consent Form No 2: Enrolment into the clinical trial**

**Participant Name**___________________________ **Study ID Number:** ____________________

| | Please initial box |
|---|---|
| 1. I confirm that I have read and understand the participant information sheet dated .......... (version .........) for the above study. I have had the opportunity to consider the information, ask questions and have had these answered fully. | |
| 2. I understand that my participation is voluntary and I am free to withdraw at any time, without giving any reason, without my medical care or legal rights being affected. | |
| 3. I understand that sections of my medical notes and data collected during the study may be looked at by responsible individuals from the London School of Hygiene & Tropical Medicine, from national regulatory authorities or from this hospital, where it is relevant to my taking part in this research. I give permission for these individuals to access my records. | |
| 4. I agree to take part in this clinical treatment trial. | |
| 5. I agree to the collection, laboratory tests and storage for future analysis of the samples from the surface my eye infection to understand the disease as described above. | |
| 6. I agree for the photographs of the front of my eye to be used in the publication or report released on the study, and for teaching purposes, including on the internet. | |
| 7. I understand that data about/from me/the participant may be shared via a public data repository or by sharing directly with other researchers, and that I will not be identifiable from this information. | |

For peer review only

Name of Participant *(printed)* Signature/Thumbprint Date

Name of Person taking consent Signature Date

The participant is unable to sign. As a witness, I confirm that all the information about the study was given and the participant consented to taking part.

Name of Impartial Witness *(if required)* Signature Date

*1 copy for participant; 1 copy for Principal Investigator; 1 copy to be kept with hospital notes*

# Appendix 2: Monitoring for Harm

## Definitions

**Adverse Event (AE):** any untoward medical occurrence in a patient or clinical trial subject administered a medicinal product and which does not necessarily have a causal relationship with this treatment. *An AE can therefore be any unfavourable and unintended sign (including an abnormal laboratory finding), symptom, or disease temporally associated with the use of an investigational medicinal product (IMP), whether or not considered related to the IMP.*

**Adverse Reaction (AR):** all untoward and unintended responses to an IMP related to any dose administered. *All AEs judged by either the reporting investigator or the sponsor as having reasonable causal relationship to a medicinal product qualify as adverse reactions. The expression reasonable causal relationship means to convey in general that there is evidence or argument to suggest a causal relationship.*

**Unexpected Adverse Reaction:** an AR, the nature or severity of which is not consistent with the applicable product information (e.g. investigator's brochure for an unapproved investigational product or summary of product characteristics (SmPC) for an authorised product*). When the outcome of the adverse reaction is not consistent with the applicable product information this adverse reaction should be considered as unexpected. Side effects documented in the SmPC which occur in a more severe form than anticipated are also considered to be unexpected.*

**Serious Adverse Event (SAE)** or **Serious Adverse Reaction:** any untoward medical occurrence or effect that at any dose

- **Results in death**
- **Is life-threatening** – *refers to an event in which the subject was at risk of death at the time of the event; it does not refer to an event which hypothetically might have caused death if it were more severe*
- **Requires hospitalisation, or prolongation of existing inpatients' hospitalisation**
- **Results in persistent or significant disability or incapacity**
- **Is a congenital anomaly or birth defect**

Medical judgement should be exercised in deciding whether an AE/AR is serious in other situations. Important AE/ARs that are not immediately life-threatening or do not result in death or hospitalisation but may jeopardise the subject or may require intervention to prevent one of the other outcomes listed in the definition above, should also be considered serious.

**Suspected Unexpected Serious Adverse Reaction (SUSAR):** any suspected adverse reaction related to an IMP that is both unexpected and serious.

## Causality

Most adverse events and adverse drug reactions that occur in this study, whether they are serious or not, will be expected treatment-related toxicities due to the drugs used in this study. The assignment of the causality should be made by the investigator responsible for the care of the participant using the definitions in the table below.

| Relationship | Description |
|---|---|
| **Unrelated** | There is no evidence of any causal relationship |
| **Unlikely** | There is little evidence to suggest there is a causal relationship (e.g. the event did not occur within a reasonable time after administration of the trial medication). There is another reasonable explanation for the event (e.g. the participant's clinical condition, other concomitant treatment). |
| **Possible** | There is some evidence to suggest a causal relationship (e.g. because the event occurs within a reasonable time after administration of the trial medication). However, the influence of other factors may have contributed to the event (e.g. the participant's clinical condition, other concomitant treatments). |
| **Probable** | There is evidence to suggest a causal relationship and the influence of other factors is unlikely. |

For peer review only

| | |
|---|---|
| **Definitely** | There is clear evidence to suggest a causal relationship and other possible contributing factors can be ruled out. |
| **Not assessable** | There is insufficient or incomplete evidence to make a clinical judgement of the causal relationship. |

If any doubt about the causality exists the local investigator should inform the study coordination centre who will notify the Chief Investigators. The pharmaceutical companies and/or other clinicians may be asked to advise in some cases.

In the case of discrepant views on causality between the investigator and others, all parties will discuss the case. In the event that no agreement is made, the MHRA will be informed of both points of view.

## Reporting Procedures

All adverse events should be reported. Depending on the nature of the event the reporting procedures below should be followed. Any questions concerning adverse event reporting should be directed to the study coordination centre in the first instance. A flowchart is given below (Figure 5) to aid in the reporting procedures and the reporting form is shown in (Data Collection Appendix).

***Figure 5: Adverse event reporting flow-chart***

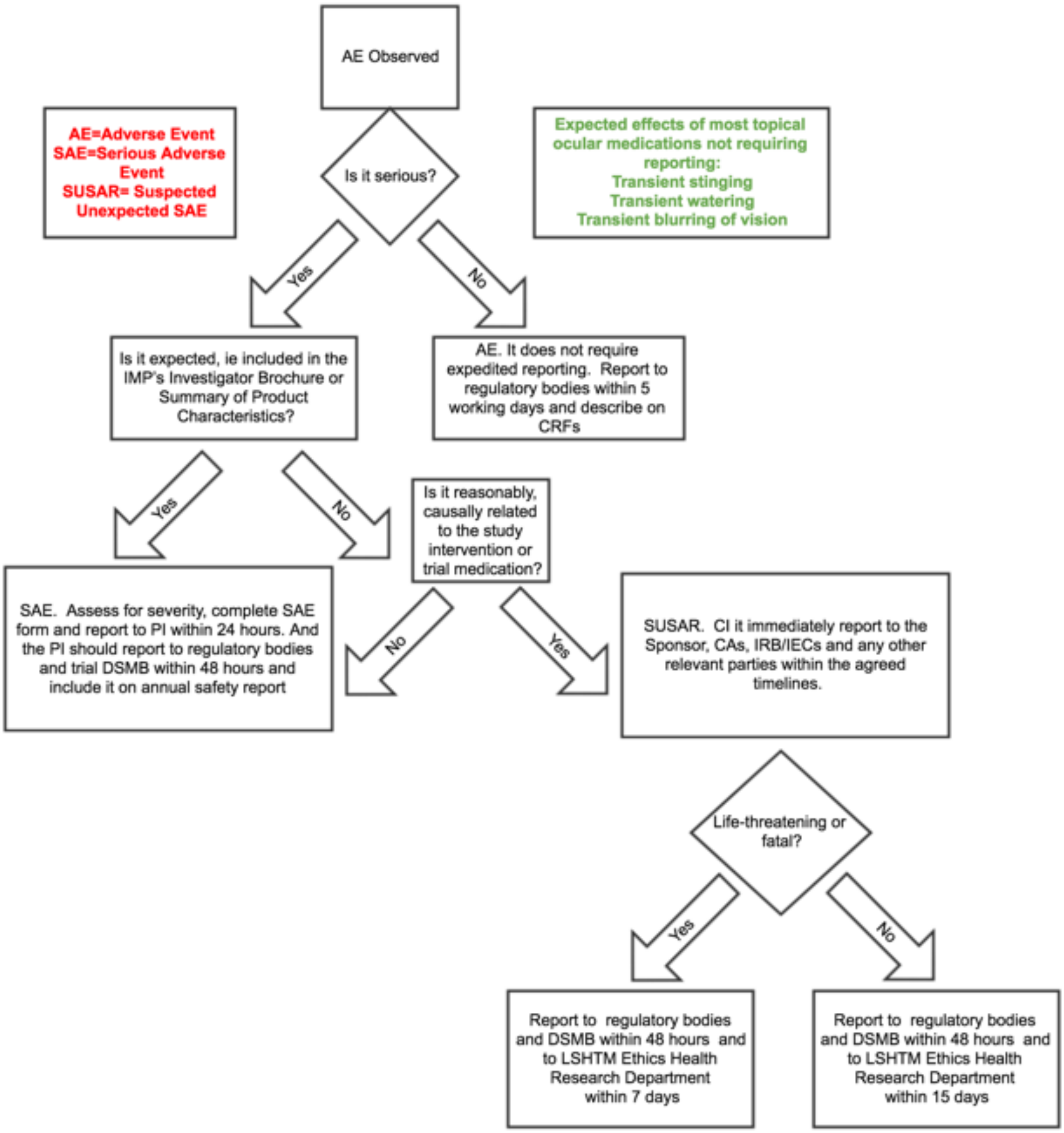

**Non-serious Adverse Reactions (ARs)/Adverse Events (AEs)**
All such events, whether expected or not, should be recorded in the toxicity section of the relevant case report form and sent to the study coordination centre, regulatory bodies and the trial DSMB within 5 working days from the day of occurrence.

**Serious Adverse Reactions (SARs)/Serious Adverse Events (SAEs)**
Fatal or life threatening Serious Adverse Events (SAEs), Serious Adverse Reactions (SARs) and Suspected Unexpected Serious Adverse Reactions (SUSARs) should be reported to the PI on the day that the local site is aware of the event. The SAE form asks for nature of event, date of onset, severity, corrective therapies given, outcome and causality (i.e. unrelated, unlikely, possible, probably, definitely). All SAEs or SARs whether expected, suspected or unexpected should be reported to regulatory bodies and the trial DSMB within 48 hours of occurrence. The responsible investigator should sign the causality of the event. Additional information should be sent within 5 days if the reaction has not resolved at the time of reporting. All investigators will be informed of all SARs occurring throughout the study.

**SAEs**
A SAE form should be completed and faxed to the study coordination centre for all SAEs within 24 hours. However, relapse and death due to a known pre-existing condition and hospitalisations for elective treatment of a pre-existing condition do not need reporting as SAEs.

**SUSARs**
In the case of serious, unexpected and related adverse events, the staff at the site should:

Complete the SAE case report form & send it immediately (within 24 hours, preferably by fax), signed and dated to the study coordination centre together with relevant treatment forms and anonymised copies of all relevant investigations.

**Or**

Contact the study coordination centre by phone and then send the completed SAE form to the study coordination centre within the following 24 hours as above.

The study coordination centre will notify all SUSARs occurring during the study within 48 hours to the Nepal Department of Drug Administration, and the trial DSMB. SUSARs will be reported to LSHTM Ethics Committee according to the following timelines; fatal and life-threatening within 7 days of notification and non-life threatening within 15 days. All investigators will be informed of all SUSARs occurring throughout the study.

Local investigators should report any SUSARs and /or SAEs as required by their Local Research Ethics Committee and/or Research & Development Office.

**Adverse Event Reporting for this Trial**
**Non-serious Ocular: narrative form not required**
- ☐ Local allergic reaction (conjunctiva, lid)
- ☐ Intraocular pressure ≥35 mmHg for 1 week despite therapy
- ☐ Progressive corneal thinning, ≤50% of enrolment thickness
- ☐ Other (specify)

**Serious Ocular: (1) report within 24hrs (2) Serious Adverse Event (SAE) Form required**
- ☐ Endophthalmitis
- ☐ Other serious ocular event thought to be related to the study drug, requiring non-elective hospitalization, loss of function, or systemic treatment (specify)

**Serious Non-ocular: (1) report within 24hrs (2) Serious Adverse Event (SAE) Form required**
- ☐ Death
- ☐ Non-elective surgery, hospitalization, or loss of function
- ☐ Myocardial infarction or stroke
- ☐ Systemic allergic reaction

For peer review only

## Appendix 3: Biological Specimens

The project will equip and staff an ocular microbiology laboratory at the hospital. The microbiology analysis will involve the following investigations:

1) **Microscopy** will be performed of the slides using Gram stain, KOH and calcofluor white.

2) **Culture** will be performed using a variety of media inoculated with corneal scrape samples in the clinic: blood agar, chocolate agar, brain-heart infusion broth (incubated for 2-7 days at 37°C) and Sabouraud agar (incubated for 7 days at 30°C). Culture isolates will be identified using standard microbiological techniques. Fungal and bacterial isolates will be stored frozen for long-term reference and subsequent additional analysis.

3) **Antibiotic susceptibility testing** of bacteria culture isolates will be performed using standard disc diffusion techniques in each of the laboratories.

4) **Antifungal susceptibility testing** of fungal isolates will be performed at KCMC, Tanzania . Isolates from Uganda and Nepal will be periodically transferred to KCMC, under a Material Transfer Agreement. They will be re-cultured and tested using a dilution series method for four drugs: Natamycin, Chlorhexidine, Voriconazole and Amphotericin-B. The technique has been developed at Radboud University, The Netherlands.

**PCR for microorganisms**

DNA from the dry swab will be extracted using the QIAamp mini kit (Qiagen) with bead-beating. Samples will be tested for the presence of bacterial (16rRNA), fungal (ITS1) and specific viral (HSV1&2) DNA by quantitative PCR. This PCR work will be performed at KCMC, Tanzania. Samples from Uganda and Nepal will be periodically transferred to KCMC for testing, under a Material Transfer Agreement. The performance of PCR as a diagnostic tool will be evaluated against a composite reference standard for positive cases. Cases will be considered positive if the pathogen is detected by either smear microscopy, IVCM or culture.

**Point of care tests for fungal infection**

Point-of-care tests for fungal keratitis would be very helpful. Molecular tests are generally more sensitive than antigen-based assays. Isothermal nucleic amplification techniques offer the sensitivity of PCR amplification without the need for specialized equipment. A result may be obtained within 30 minutes. Promising molecular approaches include isothermal amplification (Loop-mediated isothermal amplification (LAMP), Recombinase polymerase amplification (RPA)), associated with lateral flow assays for amplicon detection. Several assays have been developed that detect fungi, however, these have not been evaluated in fungal keratitis. The second ulcer swab will be used to test and develop isothermal molecular assays in comparison with the standard microbiological approaches described above. Results will be evaluated against a composite reference standard for fungal diagnosis as described above.

For peer review only

**Fungal DNA sequencing**

It is not always possible to determine the species of a fungus from classical microbiological techniques. It is now common to use fungal DNA sequencing for species-level diagnosis for some genera, including *Fusarium spp.*, which is the commonest fungal genus reported for MK in the study settings. We will use sequencing of the fungal ITS and TEF1$\alpha$ regions for this.[1] DNA will be extracted from the cultured fungal isolate when available. If there no fungus is cultured but the PCR is positive, then this PCR product will be used for sequencing. The DNA will be extracted from the isolates in KCMC, Tanzania. The extracted DNA will be sent under a Material Transfer Agreement to the Center of Expertise in Mycology Radboudumc/CWZ, Radboud University Nijmegen Medical Center and the CBS Fungal Biodiversity Centre, Utrecht, the Netherlands for the specialised sequencing and identification work.

**Corneal gene expression**

Three prime RNAseq (3'RNAseq) generates one read per transcript and enables strand-specific, highly multiplexed sequencing of poor concentration or quality samples (as low as 100pg total RNA input) for gene expression analysis. We have previously used this method for gene expression profiling of human conjunctival swabs from a trachoma-endemic area. RNA will be extracted from corneal swabs or scrapes using Norgen RNA purification kits. 3'RNAseq (Lexogen) will be performed on normalised RNA extracts from 500 Tanzanian and Ugandan participants with confirmed fungal keratitis. Pooled libraries will be sequenced on a HiSeq (Illumina). Transcriptomic profiles will be analysed for differences between controls and mild, moderate and severe FK cases using DEseq2, and between participants with good versus poor visual outcomes. A small number of healthy corneal tissue controls will be derived from the residual ring of tissue remaining after corneal graft surgery (ethics application for control tissue to be submitted separately).

**Human Genetics**

DNA will be extracted from buccal brush samples using QIAmp DNA mini kits (Qiagen). Samples from 500 confirmed fungal keratitis participants from Nepal and 500 matched controls will be run on immune-focussed Infinium ImmunoArray-24 BeadChips (Illumina) covering >240,000 genetic polymorphisms (including copy number variants, germline variants, insertions-deletions, single nucleotide polymorphisms (SNPs) and structural variants) at UCL genomics centre. In addition to SNP testing, two-field resolution Human Leukocyte Antigen (HLA) allele typing will be performed in all 1000 samples. Allele level HLA typing cannot currently be determined from SNP data but the HLA genes are primary immune response genes that have a role in many diseases and warrant independent study. Data will be analysed to determine sources of immunogenetic variation associated with fungal keratitis. These data will be linked to gene expression data from the same individuals to determine functional effects of genetic variation associated with fungal keratitis.

For peer review only

**HIV testing**

As outlined above, patients with microbial keratitis will be offered counselling and testing for HIV infection. This will be done through the established services in each of the hospitals. Appropriate onward referrals will be made if a test is found to be positive.

**Blood glucose testing**

Patients will have capillary blood glucose (CBG) testing done at presentation. Patients with an elevated CBG (>6.1mmol) will be referred to the hospital physicians for assessment and formal diagnosis of impaired glucose tolerance or diabetes mellitus. This level is considered a suitable cut-off to detect individuals with diabetes and has been validated in a south-Asian population.[2]

For peer review only

**References:**

1 Schoch CL, Seifert KA, Huhndorf S, *et al.* Nuclear ribosomal internal transcribed spacer (ITS) region as a universal DNA barcode marker for Fungi. *Proc Natl Acad Sci USA* 2012;**109**:6241–6. doi:10.1073/pnas.1117018109

2 Somannavar S, Ganesan A, Deepa M, *et al.* Random capillary blood glucose cut points for diabetes and pre-diabetes derived from community-based opportunistic screening in India. *Diabetes Care* 2009;**32**:641–3. doi:10.2337/dc08-0403

# Appendix 4: SPIRIT Checklist for Topical chlorhexidine 0.2% versus topical natamycin 5% for fungal keratitis in Nepal randomised clinical trial

| Section / Item | Item No. | Description | Location Reference |
|---|---|---|---|
| **Administrative information** | | | |
| Title | 1 | Descriptive title identifying the study design, population, interventions, and, if applicable, trial acronym | Page 1 of study protocol |
| Trial registration | 2a | Trial identifier and registry name. If not yet registered, name of intended registry | ISRCTN14332621; pre-results |
| | 2b | All items from the World Health Organization Trial Registration Data Set | ISRCTN14332621 Study Registry |
| Protocol Version | 3 | Date and version identifier | Every page of study protocol |
| Funding | 4 | Sources and types of financial, material, and other support 5a Names, affiliations, and roles of protocol contributors | Section 5.3 of study protocol, page 25<br>Page 17 of Manuscript |
| Roles and Responsibilities | 5a | Names, affiliations, and roles of protocol contributors | Page 1 of study protocol<br>Page 1 of Manuscript |
| | 5b | Name and contact information for the trial sponsor | Page 1 of study protocol<br>Page 1 of Manuscript |
| | 5c | Role of study sponsor and funders, if any, in study design; collection, management, analysis, and interpretation of data; writing of the report; and the decision to submit the report for publication, including whether they will have ultimate authority over any of these activities | Page 1 of study protocol and Section 5.2 of study protocol, page 25.<br><br>Page 17 of Manuscript |
| | 5d | Composition, roles, and responsibilities of the coordinating centre, steering committee, endpoint adjudication committee, data management team, and other individuals or groups overseeing the trial, if applicable (see Item 21a for data monitoring committee) | Section 5.1, page 24 of study protocol<br><br>Section 6, page 25 of study protocol<br><br>Page 11 of Manuscript |
| **Introduction** | | | |
| Background and rationale | 6a | Description of research question and justification for undertaking the trial, including summary of relevant studies (published and unpublished) examining benefits and harms for each intervention | Section 1, pages 6-9 of study protocol<br><br>Pages 4-5 of Manuscript |
| | 6b | Explanation for choice of comparators | Sections 1.3 – 1.4, pages 7-9 of study protocol<br>Pages 4-5 of Manuscript |

For peer review only

| | | | |
|---|---|---|---|
| Objectives | 7 | Specific objectives or hypotheses | Section 3, page 11 of study protocol<br><br>Page 5 of Manuscript |
| Trial design | 8 | Description of trial design including type of trial (eg, parallel group, crossover, factorial, single group), allocation ratio, and framework (eg, superiority, equivalence, noninferiority, exploratory) | Section 4, pages 12-24 of study protocol<br><br>Page 6 of Manuscript |
| **Methods: Participants, interventions, and outcomes** | | | |
| Study setting | 9 | Description of study settings (eg, community clinic, academic hospital) and list of countries where data will be collected. Reference to where list of study sites can be obtained | Section 4.3, page 13 of study protocol<br><br>Page 6 of Manuscript |
| Eligibility criteria | 10 | Inclusion and exclusion criteria for participants. If applicable, eligibility criteria for study centres and individuals who will perform the interventions (eg, surgeons, psychotherapists) | Section 4.4, page 13 of study protocol<br><br>Page 7 of Manuscript and Table 1 of Manuscript |
| Interventions | 11a | Interventions for each group with sufficient detail to allow replication, including how and when they will be administered | Section 4.7, page 17 of study protocol<br><br>Pages 8-9 of Manuscript |
| | 11b | Criteria for discontinuing or modifying allocated interventions for a given trial participant (eg, drug dose change in response to harms, participant request, or improving/worsening disease) | Page 11 of Manuscript |
| | 11c | Strategies to improve adherence to intervention protocols, and any procedures for monitoring adherence (eg, drug tablet return, laboratory tests) | Section 4.9, page 19 of study protocol<br><br>Pages 10-11 of Manuscript |
| | 11d | Relevant concomitant care and interventions that are permitted or prohibited during the trial | Section 4.7, pages 17-18 of study protocol<br>Pages 9-10 of Manuscript |
| Outcomes | 12 | Primary, secondary, and other outcomes, including the specific measurement variable (eg, systolic blood pressure), analysis metric (eg, change from baseline, final value, time to event), method of aggregation (eg, median, proportion), and time point for each outcome. Explanation of the clinical relevance of chosen efficacy and harm outcomes is strongly recommended | Section 4.11, page 22 of study protocol<br><br>Pages 10-11 of Manuscript |

For peer review only

| | | | |
|---|---|---|---|
| Participant timeline | 13 | Time schedule of enrolment, interventions (including any run-ins and washouts), assessments, and visits for participants. A schematic diagram is highly recommended (see Figure) | Table 3, page 19 of study protocol<br><br>Table 4 of Manuscript |
| Sample size | 14 | Estimated number of participants needed to achieve study objectives and how it was determined, including clinical and statistical assumptions supporting any sample size calculations | Section 4.12, page 22 of study protocol<br><br>Pages 12 -13 of Manuscript |
| Recruitment | 15 | Strategies for achieving adequate participant enrolment to reach target sample size | Section 4.5, page 14 of study protocol<br><br>Page 7 of Manuscript |
| **Methods: Assignment of interventions (for controlled trials)** | | | |
| Allocation: sequence generation | 16a | Method of generating the allocation sequence (eg, computer- generated random numbers), and list of any factors for stratification. To reduce predictability of a random sequence, details of any planned restriction (eg, blocking) should be provided in a separate document that is unavailable to those who enrol participants or assign interventions | Section 4.8, pages 18-19 of study protocol<br><br>Pages 7-8 of Manuscript |
| Allocation: concealment mechanism | 16b | Mechanism of implementing the allocation sequence (eg, central telephone; sequentially numbered, opaque, sealed envelopes), describing any steps to conceal the sequence until interventions are assigned | |
| Allocation: Implementation | 16c | Who will generate the allocation sequence, who will enrol participants, and who will assign participants to interventions | |
| Blinding (masking) | 17a | Who will be blinded after assignment to interventions (eg, trial participants, care providers, outcome assessors, data analysts), and how | Section 4.8, pages 18-19 of study protocol<br><br>Page 8 of Manuscript |
| | 17b | If blinded, circumstances under which unblinding is permissible, and procedure for revealing a participant's allocated intervention during the trial | Page 8 of Manuscript |
| **Methods: Data collection, management, and analysis** | | | |
| | 18a | Plans for assessment and collection of outcome, baseline, and other trial data, including any | Section 4.14, page 23 of study protocol |

For peer review only

| | | | |
|---|---|---|---|
| Data collection methods | | related processes to promote data quality (eg, duplicate measurements, training of assessors) and a description of study instruments (eg, questionnaires, laboratory tests) along with their reliability and validity, if known. Reference to where data collection forms can be found, if not in the protocol | Page 12 of Manuscript |
| | 18b | Plans to promote participant retention and complete follow-up, including list of any outcome data to be collected for participants who discontinue or deviate from intervention protocols | Page 12 of Manuscript |
| Data management | 19 | Plans for data entry, coding, security, and storage, including any related processes to promote data quality (eg, double data entry; range checks for data values). Reference to where details of data management procedures can be found, if not in the protocol | Section 4.14, page 23 of study protocol<br><br>Page 12 of Manuscript |
| Statistical methods | 20a | Statistical methods for analysing primary and secondary outcomes. Reference to where other details of the statistical analysis plan can be found, if not in the protocol | Section 4.13, pages 22-23 of study protocol<br><br>Pages 12-15 of Manuscript |
| | 20b | Methods for any additional analyses (eg, subgroup and adjusted analyses) | |
| | 20c | Definition of analysis population relating to protocol non-adherence (eg, as randomised analysis), and any statistical methods to handle missing data (eg, multiple imputation) | |
| **Methods: Monitoring** | | | |
| Data Monitoring | 21a | Composition of data monitoring committee (DMC); summary of its role and reporting structure; statement of whether it is independent from the sponsor and competing interests; and reference to where further details about its charter can be found, if not in the protocol. Alternatively, an explanation of why a DMC is not needed | Section 6, page 25 of study protocol<br><br>Page 12 of Manuscript |
| | 21b | Description of any interim analyses and stopping guidelines, including who will have access to | Section 4.13, page 23 of study protocol |

For peer review only

| | | | |
|---|---|---|---|
| | | these interim results and make the final decision to terminate the trial | Page 15 of Manuscript |
| Harms | 22 | Plans for collecting, assessing, reporting, and managing solicited and spontaneously reported adverse events and other unintended effects of trial interventions or trial conduct | Section 7, pages 26-28 of study protocol<br><br>Page 12 of Manuscript<br><br>Annex 2 |
| Auditing | 23 | Frequency and procedures for auditing trial conduct, if any, and whether the process will be independent from investigators and the sponsor | Section 6, page 25 of study protocol<br><br>Page 12 of Manuscript |
| **Ethics and Dissemination** | | | |
| Research ethics approval | 24 | Plans for seeking research ethics committee/institutional review board (REC/IRB) approval | Section 4.15, page 24 of study protocol<br>Page 15 of Manuscript |
| Protocol amendments | 25 | Plans for communicating important protocol modifications (eg, changes to eligibility criteria, outcomes, analyses) to relevant parties (eg, investigators, REC/IRBs, trial participants, trial registries, journals, regulators) | Section 4.15, page 24 of study protocol<br>Page 15 of Manuscript |
| Consent or assent | 26a | Who will obtain informed consent or assent from potential trial participants or authorised surrogates, and how (see Item 32) | Section 4.5, page 14 of study protocol<br><br>Page 7 of Manuscript |
| | 26b | Additional consent provisions for collection and use of participant data and biological specimens in ancillary studies, if applicable | Appendix 1: Consent Forms |
| Confidentiality | 27 | How personal information about potential and enrolled participants will be collected, shared, and maintained in order to protect confidentiality before, during, and after the trial | Page 12 of Manuscript |
| Declaration of interests | 28 | Financial and other competing interests for principal investigators for the overall trial and each study site | Page 18 of submitted manuscript |
| Access to data | 29 | Statement of who will have access to the final trial dataset, and disclosure of contractual agreements that limit such access for investigators | Page 12 of Manuscript |
| Ancillary and post-trial care | 30 | Provisions, if any, for ancillary and post-trial care, and for compensation to those who suffer harm from trial participation | Page 9 of Manuscript |
| Dissemination policy | 31a | Plans for investigators and sponsor to communicate trial results to participants, healthcare | Section 10, page 33 of study protocol |

For peer review only

| | | | |
|---|---|---|---|
| | | professionals, the public, and other relevant groups (eg, via publication, reporting in results databases, or other data sharing arrangements), including any publication restrictions | Pages 15-16 of Manuscript |
| | 31b | Authorship eligibility guidelines and any intended use of professional writers | Page 24 of Manuscript |
| | 31c | Plans, if any, for granting public access to the full protocol, participant- level dataset, and statistical code | Page 24 of Manuscript |
| **Appendices** | | | |
| Informed consent materials | 32 | Model consent form and other related documentation given to participants and authorised surrogates | Appendix 1 of Manuscript |
| Biological specimens | 33 | Plans for collection, laboratory evaluation, and storage of biological specimens for genetic or molecular analysis in the current trial and for future use in ancillary studies, if applicable | Appendix 3 of Manuscript |

For peer review only

BMJ Open

# Topical chlorhexidine 0.2% versus topical natamycin 5% for fungal keratitis in Nepal: rationale and design of a randomised controlled non-inferiority trial

| | |
|---|---|
| Journal: | *BMJ Open* |
| Manuscript ID | bmjopen-2020-038066.R2 |
| Article Type: | Protocol |
| Date Submitted by the Author: | 23-Jul-2020 |
| Complete List of Authors: | Hoffman, Jeremy; London School of Hygiene and Tropical Medicine International Centre for Eye Health; Sagarmatha Choudhary Eye Hospital<br>Yadav, Reena; Sagarmatha Choudhary Eye Hospital<br>Das Sanyam, Sandip; Sagarmatha Choudhary Eye Hospital<br>Chaudhary, Pankaj; Sagarmatha Choudhary Eye Hospital<br>Roshan, Abhishek; Sagarmatha Choudhary Eye Hospital<br>Singh, Sanjay; Eastern Region Eye Care Programme<br>Arunga, Simon; London School of Hygiene and Tropical Medicine International Centre for Eye Health, International Centre for Eye Health; Mbarara University of Science and Technology Faculty of Medicine<br>Matayan, Einoti; Kilimanjaro Christian Medical Centre, Department of Ophthalmology<br>Macleod, David; London School of Hygiene & Tropical Medicine, MRC Tropical Epidemiology Group<br>Weiss, Helen; London School of Hygiene & Tropical Medicine, MRC Tropical Epidemiology Group<br>Leck, Astrid; London School of Hygiene and Tropical Medicine International Centre for Eye Health<br>Hu, Victor; London School of Hygiene and Tropical Medicine International Centre for Eye Health<br>Burton, Matthew J; London School of Hygiene and Tropical Medicine International Centre for Eye Health; Moorfields Eye Hospital NHS Foundation Trust |
| <b>Primary Subject Heading</b>: | Ophthalmology |
| Secondary Subject Heading: | Evidence based practice, Global health, Infectious diseases, Research methods |
| Keywords: | Corneal and external diseases < OPHTHALMOLOGY, OPHTHALMOLOGY, Clinical trials < THERAPEUTICS, MYCOLOGY |
| | |

SCHOLARONE™
Manuscripts

*I, the Submitting Author has the right to grant and does grant on behalf of all authors of the Work (as defined in the below author licence), an exclusive licence and/or a non-exclusive licence for contributions from authors who are: i) UK Crown employees; ii) where BMJ has agreed a CC-BY licence shall apply, and/or iii) in accordance with the terms applicable for US Federal Government officers or employees acting as part of their official duties; on a worldwide, perpetual, irrevocable, royalty-free basis to BMJ Publishing Group Ltd ("BMJ") its licensees and where the relevant Journal is co-owned by BMJ to the co-owners of the Journal, to publish the Work in this journal and any other BMJ products and to exploit all rights, as set out in our licence.*

*The Submitting Author accepts and understands that any supply made under these terms is made by BMJ to the Submitting Author unless you are acting as an employee on behalf of your employer or a postgraduate student of an affiliated institution which is paying any applicable article publishing charge ("APC") for Open Access articles. Where the Submitting Author wishes to make the Work available on an Open Access basis (and intends to pay the relevant APC), the terms of reuse of such Open Access shall be governed by a Creative Commons licence – details of these licences and which Creative Commons licence will apply to this Work are set out in our licence referred to above.*

*Other than as permitted in any relevant BMJ Author's Self Archiving Policies, I confirm this Work has not been accepted for publication elsewhere, is not being considered for publication elsewhere and does not duplicate material already published. I confirm all authors consent to publication of this Work and authorise the granting of this licence.*

For peer review only

# Topical chlorhexidine 0.2% versus topical natamycin 5% for fungal keratitis in Nepal: rationale and design of a randomised controlled non-inferiority trial

**Authors:**

Jeremy J Hoffman FRCOphth (1, 2, 5), Reena Yadav MD (2), Sandip Das Sanyam (2), Pankaj Chaudhary (2), Abhishek Roshan (2), Sanjay K Singh MD (3), Simon Arunga (1, 4), Einoti Matayan (5), David Macleod PhD (1, 6), Helen A Weiss PhD (6), Astrid Leck PhD (1), Victor H Hu PhD FRCOphth (1), Matthew J Burton PhD FRCOphth (1, 7).

**Affiliations:**

1. International Centre for Eye Health, London School of Hygiene and Tropical Medicine, London, UK
2. Sagarmatha Choudhary Eye Hospital, Lahan, Nepal
3. Eastern Region Eye Care Programme, Biratnagar, Nepal
4. Mbarara University of Science and Technology, Mbarara, Uganda
5. Kilimanjaro Christian Medical Centre, Moshi, Tanzania
6. MRC Tropical Epidemiology Group, London School of Hygiene & Tropical Medicine, London, UK
7. Moorfields Eye Hospital, London, UK

**Corresponding author:**

Jeremy J Hoffman FRCOphth

International Centre for Eye Health

London School of Hygiene and Tropical Medicine

Keppel Street

London

WC1E 7HT

United Kingdom

jeremy.hoffman@lshtm.ac.uk

**Key words:** Clinical trial; fungal keratitis; chlorhexidine; natamycin; corneal ulcer, Nepal.

**Word count:** 5132 (excluding abstract, titles, tables, figures and references)

For peer review only

## ABSTRACT

### Introduction

Fungal infections of the cornea, fungal keratitis (FK), are challenging to treat. Current topical antifungals are not always effective and are often unavailable, particularly in low and middle-income countries where most cases occur. Topical natamycin 5% is usually first-line treatment, however, even when treated intensively, infections may progress to perforation of the eye in around a quarter of cases. Alternative anti-fungal medications are needed to treat this blinding disease.

Chlorhexidine is an antiseptic agent with antibacterial and antifungal properties. Previous pilot studies suggest that topical chlorhexidine 0.2% compares favourably with topical natamycin. Full-scale randomised controlled trials (RCT) of topical chlorhexidine 0.2% are warranted to answer this question definitively.

### Methods and analysis

We will test the hypothesis that topical chlorhexidine 0.2% is non-inferior to topical natamycin 5% in a two-arm, single-masked RCT. Participants are adults with FK presenting to a tertiary ophthalmic hospital in Nepal. Baseline assessment includes history, examination, photography, *in vivo* confocal microscopy and cornea scrapes for microbiology. Participants will be randomised to alternative topical antifungal treatments (topical chlorhexidine 0.2% and topical natamycin 5%; 1:1 ratio, 2-6 random block size). Patients are reviewed at day 2, day 7 (with re-culture), day 14, day 21, month 2 and month 3. The primary outcome is best spectacle corrected visual acuity (BSCVA) at three months. Primary analysis (intention-to-treat) will be by linear regression, with treatment arm and baseline BSCVA pre-specified covariates. Secondary outcomes include epithelial healing time, scar/infiltrate size, ulcer depth, hypopyon size, perforation and/or therapeutic penetrating keratoplasty (TPK, corneal transplant), positive re-culture rate (day-7) and quality of life (EQ-5D, WHO/PBD-VF20, WHOQOL-BREF).

### Ethics and dissemination

The Nepal Health Research Council, the Nepal Department of Drug Administration and the London School of Hygiene and Tropical Medicine ethics committee have approved the trial. The results will be presented at local and international meetings and submitted to peer-reviewed journals for publication.

### Trial Registration

ISRCTN14332621; pre-results.


### Article summary

For peer review only

- First large-scale randomised controlled clinical trial comparing chlorhexidine 0.2% to natamycin 5% for the treatment of fungal keratitis.
- This study benefits from a pragmatic design: as a non-inferiority trial, if chlorhexidine is found to be within a pre-defined non-inferiority margin of 0.15 logMAR of natamycin at 3 months a recommendation to use chlorhexidine 0.2% can be made; this is a far cheaper, easy to formulate medication that could significantly increase access to anti-fungal treatment for the target population.
- Clinicians are masked to the treatment allocation, however, due to different physical appearance it is not possible to mask patients to their allocated treatment.
- This study will also assess the superiority of either medication as well as a number of key secondary outcome measures, analysed by arm.
- First randomised controlled trial investigating fungal keratitis to use *in vivo* confocal microscopy as a diagnostic tool for the detection of fungal elements.

For peer review only

## INTRODUCTION

Fungal keratitis (FK) is a severe and potentially blinding corneal infection (Figure 1). [1,2] The burden is greatest in tropical and subtropical countries, probably due to a combination of climate (higher temperatures and humidity) and frequent agriculture related eye injuries. [3] It is one of the causes of microbial keratitis (MK) and accounts for between 20 – 60% of corneal infections diagnosed in tropical regions. [4] It is often inadequately treated with significant barriers to receiving appropriate, timely intervention, compounded by indiscriminate and inappropriate use of conventional medicines such as topical corticosteroids or harmful traditional eye medicines. [1,2,5] Furthermore, when appropriate treatment is available, up to 30% of patients receiving current "gold-standard" therapy progress to corneal perforation and / or eye-loss (Figure 2). [1,6,7]

Treatment for fungal keratitis almost always involves topical antifungal agents. Surgical intervention, usually in the form of therapeutic penetrating keratoplasty (TPK), is generally reserved for cases of corneal perforation or progressive infection refractory to medical therapy. Corneal transplantation is also performed for visual rehabilitation after the acute infection has resolved. There are a limited number of antifungals available for treating FK, which fall into four main groups: imidazoles, triazoles, polyenes and fluorinated pyrimidines. These may be available topically, orally or by intravenous injection. Subconjunctival, corneal stromal or intracameral injections may also be given. The treatment of yeasts (*Candida spp.*) is often different to filamentous fungi, with the former being more common in temperate climates and the latter in hot and humid locations.[8,9]

There have been several clinical trials comparing treatment options for FK, which have been systematically reviewed.[10,11] Natamycin, which was approved in the 1960s by the FDA for FK, has been compared to a number of newer agents, including voriconazole. Natamycin and voriconazole have been compared in four trials, with the meta-analysis favouring natamycin. [6,10,12-14]

As a result, first line management of filamentous FK is usually with topical natamycin 5% when this is available. This was added to the WHO Essential Medicines List in 2017 for this indication. However, even when intensive topical natamycin is initiated, infections frequently progress relentlessly to perforation and loss of the eye in about a quarter of cases, Figure 2. [1,6,7] Moreover, in many countries antifungal eye drop treatments are simply not available. This includes most countries in SSA, some Asian countries and some countries in Europe.[1,2] Natamycin is relatively expensive even if it is available. Therefore, additional alternative and more affordable drugs are clearly needed if the outcome of these infections is to improve.



For peer review only - http://bmjopen.bmj.com/site/about/guidelines.xhtml

Chlorhexidine (CHX) is an antiseptic agent, with both antibacterial and antifungal properties. It is a widely-used broad-spectrum biocide, killing microorganisms through cell membrane disruption. [15-17] CHX has been used in ophthalmology for over 30 years as an eye-drop preservative and for sterilizing contact lenses, and has also been used to treat *Acanthamoeba* and fungal keratitis. [9,18-22] In a study of potential anti-fungal treatments, CHX was effective both *in vitro* against FK isolates from India and Ghana, as well as in an Indian case series.[23] Subsequently two pilot RCTs of CHX for fungal keratitis were conducted. In the first, three CHX concentrations (0.05%, 0.1%, 0.2%) were compared to each other and natamycin 5%; this concluded CHX 0.2% had reliable antifungal action. [19] The same concentration is used in mouthwash to prevent oral candidiasis. In the second trial, CHX 0.2% was compared to topical natamycin 2.5% (half standard concentration). There was evidence CHX produced a more favourable response by 5 days (RR=0.23, 95%CI 0.09-0.63). [18] A systematic review found a trend favouring CHX over natamycin in "curing" by 21-days (RR=0.70, 95%CI 0.45-1.09), suggesting CHX might prove superior in adequately powered trials.[10] CHX is safe and well-tolerated at these concentrations. [18,19,22,24] Based on this, CHX is used for treating FK in several countries. [1,9,11] However, the combined size of these two pilot trials comparing chlorhexidine and natamycin is not sufficient to reach firm conclusions. Currently, the meta-analysis indicates equipoise in terms of which treatment is best for treating fungal keratitis.

**Objectives**

The primary objective of this study is to determine if topical chlorhexidine 0.2% is non-inferior to topical natamycin 5% for treating filamentous fungal keratitis, in terms of vision at three months. The secondary objectives are: (1) to determine whether either treatment (chlorhexidine 0.2% or natamycin 5%) is superior to the other, in terms of vision at three months; and (2) to determine whether there is a difference between chlorhexidine 0.2% and natamycin 5% in terms of secondary clinical outcomes including infiltrate / scar size, time to re-epithelialisation, re-culture rates at one week and the effect of the alternative treatments on the Quality of Life of participants.

Chlorhexidine is cheap, stable and easily prepared by aqueous dilution. If chlorhexidine is found to be non-inferior (or even superior) to natamycin this offers the potential of an effective, affordable and accessible treatment for fungal keratitis, which could benefit millions of people each year who currently have no treatment options. This trial is a response to this expressed need from both clinicians and patients for a readily available and affordable medication for fungal infection.



For peer review only - http://bmjopen.bmj.com/site/about/guidelines.xhtml

## METHODS AND ANALYSIS

### Trial Design

We are conducting a single-masked, non-inferiority randomised controlled trial comparing chlorhexidine 0.2% to natamycin 5% for the treatment of fungal keratitis. The non-inferiority design of this trial offers a clinically pragmatic way to address this important question: if CHX is found to be within the pre-specified non-inferiority margin of 0.15 logMAR (about 1.5 Snellen lines) then CHX may prove to be a sustainable solution for this aspect of the complex problem of fungal keratitis.

### Trial Summary

This RCT follows a two-stage recruitment process (Figure 3). All patients presenting with acute microbial keratitis are reviewed and enrolled into Stage 1, following written, informed consent. This involves history, examination and investigations (corneal scrapes for microbiological assessment and *in vivo* confocal microscopy). If there is evidence of fungal hyphae on smear or confocal microscopy, patients then proceed to Stage 2. A trial eligibility checklist is completed and Stage 2 written informed consent is conducted. We will recruit 500 patients into Stage 2. Eligible FK patients are then randomised 1:1 to receive either natamycin 5% or chlorhexidine 0.2% topical treatments hourly for the first week, then two-hourly for the subsequent two weeks. Ongoing treatment duration will then be tailored to clinical response. Study personnel are masked to the treatment allocation. Patients are usually initially admitted and followed up on day-2, day-7 (with re-culture), day-14, day-21, month-2 and month-3. The primary outcome is best spectacle corrected visual acuity (BSCVA) at three months.

### Trial Setting

Low-land Nepal, a region with a high burden of fungal keratitis, provides a suitable location to conduct a clinical trial that requires a relatively large sample size. This trial will be conducted in Sagarmatha Choudhary Eye Hospital (SCEH), Lahan, Siraha District in south eastern Nepal. SCEH treats approximately 800 cases of keratitis per month, half of which is attributable to fungal infection. [1,25] SCEH is a tertiary level ophthalmic hospital, with several satellite Eye Care Clinics (ECCs) that refer patients to SCEH directly as required. SCEH and its ECCs serve an estimated population of about 5 million people. Due to its proximity to the border, approximately 50% of the outpatients are Indian nationals. It is anticipated that the study participants will present to the hospital from multiple districts within the region. Potentially eligible individuals will be recruited from outpatient clinics or referred directly by the ECCs to the study team. Based on the numbers of patients attending, it should be possible to complete recruitment of 500 participants within six to twelve months. A second separate trial in East Africa (Tanzania and Uganda) will also be conducted to compare chlorhexidine 0.2% to natamycin 5% following a very similar protocol to the one described here. This will be


For peer review only - http://bmjopen.bmj.com/site/about/guidelines.xhtml

registered as a separate trial. This will enable us to assess the generalisability of the findings in two geographically distinct regions with potentially different fungal aetiologies and susceptibility patterns.

**Eligibility Criteria**

Potential participants need to meet all the inclusion criteria and have none of the exclusion criteria listed in Table 1. In summary, they need to have an active fungal keratitis defined as acute microbial keratitis characterised by corneal epithelial ulceration (>1mm in diameter), corneal stromal infiltrate and signs of acute inflammation (e.g. conjunctival injection, anterior chamber inflammatory cells, hypopyon) in conjunction with evidence of a filamentous fungal infection on smear microscopy and/or *in vivo* confocal microscopy. There is strong evidence supporting the use of IVCM for diagnosing filamentary fungal keratitis. Studies have reported sensitivities of 85.7 – 89.2% and specificities of between 81.4 and 92.7%, respectively. [26-28] As some patients will be enrolled on the basis of the results of *in vivo* confocal microscopy which is unable to detect most bacteria reliably, some patients with microscopically confirmed fungal infection will subsequently also be found to have had mixed infection at the time of being recruited into the study, as bacterial cultures may become positive a few days after enrolment. Based on previous experience at SCEH this is likely to account for about 10% of cases. These patients are included in the study but excluded from the primary analysis of the primary outcome (see below). Secondary analyses will include mixed infections.

**Table 1: Inclusion and exclusion criteria for enrolment in Stage 1 (microbial keratitis cases) and Stage 2 (the randomised controlled trial)**

| **Stage 1:** | |
|---|---|
| **Inclusion Criteria (all must be met):** | **Exclusion Criteria (any of the following):** |
| 1. Acute MK characterised by:<br>• Corneal epithelial ulceration >1mm diameter<br>• Corneal stromal infiltrate<br>• Acute inflammation: e.g. conjunctival injection, anterior chamber inflammatory cells, hypopyon.<br>2. Adults (18 years and older)<br>3. Able to provide informed consent | 1. Patients aged less than 18 years<br>2. Patients unable or unwilling to provide informed consent<br>3. Patients who do not have acute microbial keratitis or where there is a more likely alternative diagnosis |
| **Stage 2:** | |
| **Inclusion Criteria (all must be met):** | **Exclusion Criteria (any of the following):** |
| 1. Acute MK characterised by:<br>• Corneal epithelial ulceration >1mm diameter<br>• Corneal stromal infiltrate<br>• Acute inflammation: e.g. conjunctival injection, anterior chamber inflammatory cells, hypopyon.<br>2. Filamentous fungal hyphae visualised on smear microscopy and/or *in vivo* confocal microscopy.<br>3. Agree to be randomised to either treatment arm and are able to give informed consent<br>4. Agree to be followed up at 2 days, 1 week, 2 weeks, 3 weeks, 2 months and 3 months<br>5. Adults (18 years and older) | 1. Unwilling/unable to participate in trial and/or attend follow-up<br>2. Aged less than 18 years<br>3. Pregnancy: self-reported, or by urine hCG pregnancy test if uncertain.<br>4. Breast feeding: self-reported<br>5. Prior topical anti-fungal treatment<br>6. No light perception in the affected eye<br>7. Fellow eye visual acuity <6/60<br>8. Acanthamoebic infection visualised by smear microscopy or IVCM<br>9. Clinical evidence of herpetic keratitis<br>10. Known allergy to study medication (including preservatives)<br>11. Previous keratoplasty in the affected eye<br>12. Bilateral corneal ulcers<br>13. Very severe ulcers warranting immediate evisceration or conjunctival flap<br>14. Endophthalmitis |



For peer review only - http://bmjopen.bmj.com/site/about/guidelines.xhtml

For peer review only

**Consent Procedures**

There are two independent consent stages in this trial: Stage 1, where all adult patients with microbial keratitis are eligible; and Stage 2, only for fungal keratitis patients meeting the eligibility criteria. The two-stage process enables data collection on all potential patients at baseline before a diagnosis of fungal keratitis is confirmed. All patients who are eligible to participate will be given a participant information sheet in Nepali and its contents read to them. There will be an opportunity to discuss any questions that they might have. If the patient would like to participate, they will be asked to read and sign or place a thumb print on the study consent form. The consent will be witnessed by the eye health worker, confirmed by a signature on the form. For patients who are unable to read the documentation a second witness who is unrelated to the study is required. Consent Forms are given in Appendix 1.

**Baseline Assessment**

The detailed baseline assessment is described in Table 2. This includes clinical examination, corneal photography, *in vivo* confocal microscopy and the collection of microbiology samples. Quality of life questionnaires will also be completed (EQ-5D, WHO/PBD-VF20, WHOQOL-BREF).

For peer review only

**Table 2: Baseline assessment.** Assessment performed at baseline with details of how they are made.

| Assessment | Details |
|---|---|
| Visual Acuity | Presenting, Pin-Hole and Best Spectacle Corrected Visual Acuity (BSCVA) will be measured using an ETDRS Tumbling-E logMAR 3m chart (Good-Lite Inc., USA) mounted on an ESC 2000 ETDRS LED Cabinet, (Good-Lite Inc, USA) by a trial-certified optometrist, for each eye separately |
| Contrast Sensitivity | Measured using the Peek Contrast Sensitivity [29] smartphone application running on Android OS with a Sony Xperia Z3 Compact smartphone (Sony, Japan). |
| Clinical Photographs | Photographs will be taken separately of both corneas using a Nikon D7500 camera with an AF-S Micro Nikkor 105mm lens and lens mounted SB-200 flash units (Nikon, Japan). A standardised photography protocol is used to ensure images can be compared between time points. Standardised magnification will be used to allow epithelial defect and stromal infiltrate size measurements to be made. |
| Slit-lamp examination | Both eyes will be examined using a slit-lamp biomicroscope (standard ophthalmology examination) to assess the anterior segment of the eye. This examination will be performed by an ophthalmic clinician experienced in managing microbial keratitis. Particular attention will be paid to the following features:<br>1. Eyelids: trichiasis, lagophthalmos, facial weakness, Bell's reflex<br>2. Suppuration<br>3. Conjunctival inflammation<br>4. Corneal sensation<br>5. Cornea epithelial defect (measuring the longest dimension and the longest perpendicular) and ulcer depth<br>6. Corneal inflammatory infiltrate depth, size, profile, colour, edge pattern, texture, satellites.<br>7. Anterior chamber inflammatory cells, hypopyon, endothelial plaque<br>8. Relative afferent pupillary defect. |
| *In vivo* confocal microscopy (IVCM) | The Heidelberg Retinal Tomograph 3 (HRT3) *in vivo* confocal microscope enables the clinician to examine the cornea down to the cellular level. It is able to detect the presence of fungal hyphae.[26,27] A sterile, single-use disposable cap covers the objective lens and is changed between patients. Volume scans will be performed which provide a series of 400 x 400µm images over a depth range of 80µm. The resolution of the corneal scanning module is 7.6µm. IVCM images will be collected in a systematic way, starting at the centre of the ulcer, then at the superior, inferior, nasal and temporal borders of the ulcer. Volume scans will be performed in all of these locations, starting at the level of the corneal epithelium, and ending at the deepest affected aspect of the cornea assessed from IVCM images. Images will be assessed during the examination. |
| Ocular Sample Collection | The following samples will be collected from the corneal ulcer of each patient at the baseline assessment:<br>1. Corneal scrape specimens for microscopy and microbiological culture. A corneal scrape will be collected from the corneal ulcer after application of preservative free proxymetacaine local anaesthetic eye drops (Minims). Sterile needles are used to take corneal scrape specimens and then place on to glass slides for immediate Gram stain, KOH, and Calcofluor white. Samples will be directly inoculated onto blood, chocolate, Sabouraud agar and broths for culture.<br>2. Corneal specimen collection for PCR. Two sterile swabs will be gently swept over the surface of the corneal ulcer and placed into a 2ml tube. The swabs will be for pathogen detection by PCR, fungal sequencing and assessment of point of care tests for fungal infections. Swabs will be stored dry at -80°C. If swab yields are found to be too low for analysis an additional corneal scrape will be collected for PCR. The analysis of the PCR samples will not form part of the RCT work up and report. |
| HIV Testing | All individuals presenting with MK would be offered counselling and testing services. If this is found to be positive and the patient is unknown to the HIV care services an appropriate referral will be made. HIV testing is performed using HIV Tri-Dot rapid diagnostic test (J. Mitra & Co. Pvt. Ltd., India) |

For peer review only

| | |
|---|---|
| Random Blood Glucose | There is a suggestion that individuals with diabetes may be more susceptible to fungal keratitis. Participants will be offered a random blood glucose test, on a finger prick sample, analysed using HumaLyzer Primus (HUMAN Gesellschaft für Biochemica und Diagnostica mbH, Germany). If this is above 6.1 mmol/l they will be referred to the hospital physicians for assessment and formal diagnosis of impaired glucose tolerance or diabetes mellitus. This level is considered a suitable cut-off to detect individuals with diabetes and has been validated in a south-Asian population.[30] |
| Quality of Life Questionnaires | For those with confirmed fungal keratitis and who are enrolled in the trial, there will be several additional baseline assessments to evaluate the impact of FK on quality of life.<br><br>Vision Related Quality of Life (VRQoL): will be assessed by a vision disease specific tool the WHO/PBD-VF20. [31] This tool measures the impact of visual impairment in the person's life including mental wellbeing, dependency and social functioning. These have been used in a number of other vision related studies to show a difference in QoL.[32,33] This instrument consists of 20 questions divided into three sub-scales: visual symptom, general functioning and psychosocial. It begins by asking the patient *"Overall, how would you rate your eyesight using both eyes?"*; and uses a five point scale answer option such as *"very good", "good", "moderate", "bad", "very bad"*. The test is scored out of 100, with higher scores reflecting a better VRQoL.<br><br>General Health Related Quality of Life (HRQoL): We will use the EQ-5D questionnaire and EQ-Visual Analogue Scale. The EQ-5D is a standardised tool to measure health outcomes. [34] Patients will also be assessed using the WHOQOL-BREF. This has good applicability in low and middle-income countries (LMIC) as it was developed simultaneously from concept across 18 countries in Africa, Asia and Latin America. It measures 4 domains of health: Physical Health, Psychological Health, Social Relationships, and Environment. It asks respondents 26 questions how much (frequency) they have experienced and/or were able to do things (e.g. feel safe, able to concentrate, enjoy life) in the past 4 weeks and how satisfied they are with certain aspects of their lives (e.g. sleep, capacity for work).[34] |

For peer review only

**Randomisation and Masking**

***Sequence generation***: a computer-generated randomisation list will be prepared by an independent statistician at LSHTM, who will hold the sequence, will not be masked, and will not be involved in any other aspect of the trial. The sequence is in a 1:1 allocation ratio of CHX to NATA, with a random block size (2, 4 or 6).

***Allocation concealment and implementation:*** the randomisation sequences will be concealed in sequentially numbered, opaque envelopes. The envelopes will be prepared by a person independent of all other aspects of the trial. The randomisation administrator (nurse or pharmacist) will conduct the random allocation procedure. The investigational products will be stored in the trial coordination office in a locked and separate drug cabinet dedicated for this clinical trial only. The cabinet will only be used for drug storage and will only be accessed by the randomisation administrator. The randomisation administrator will work in a separate room in the clinic. After the patient has been recruited and assessed, they will be guided to this separate room where the randomisation allocation will be conducted.

***Masking:*** The topical treatments being compared in this trial have a different appearance: the chlorhexidine 0.2% is a clear, colourless solution and the natamycin 5% is an opaque, white suspension. Therefore, it is not possible to mask the participants to this difference in visual appearance. However, the patients will not be told which treatment they have been allocated. Prior to any follow-up clinical examinations, a nurse, otherwise uninvolved in the study, will wipe away any white natamycin residue from the patient's eyes to avoid unmasking the clinical assessor. This procedure was used successfully in other trials.[6] All clinicians involved in the clinical assessment of the patients will be masked to the allocation. The statistician who will perform the primary analysis will be masked to the allocation and only receive the actual allocation code sequence from the independent statistician after the analysis code has been prepared and pre-tested with a test sequence. The primary outcome is visual acuity at 3-months assessed by an optometrist who will not have been involved in any other aspects of the trial and masked to the allocation. By three months the treatment courses are likely to be completed. There will be masked grading of the photographs to independently confirm outcome measures and assess for any systematic bias on the part of the clinical examiners.

***Unmasking:*** Unmasking is a serious action and should only be performed if necessary to ensure the safety of a study participant. Anyone unmasked to randomised treatment for the purpose of creating analyses for independent data and safety monitoring committees will not be involved in any other aspects of the conduct or final analysis of the study. A list will be maintained of all unmasked members of staff and will be approved by the Chief Investigator. Such staff members must sign a document to indicate that they are aware of their



For peer review only - http://bmjopen.bmj.com/site/about/guidelines.xhtml

responsibilities with respect to confidentiality. The processes used to provide access to unmasked treatment codes and reports relating to these codes shall be documented.

**Intervention and Treatment**

The standard of care in this hospital is for cases of fungal keratitis to be admitted. If willing, patients will be admitted for close observation and supervised treatment, until there are signs of improvement and the supervising clinician considers it safe to provide ongoing management on an outpatient basis.

***Trial Treatment Arms***

A) *Chlorhexidine 0.2% w/v eye drops*

Chlorhexidine 0.2% w/v solution, as eye drops, will be applied to the surface of the infected eye (one drop per application). The chlorhexidine 0.2% w/v eye drops used in these studies is produced by Mandeville Medicines, UK.

B) *Natamycin 5% eye drops*

Natamycin 5% w/v suspension, as eye drops, will be applied to the surface of the infected eye (one drop per application) Topical natamycin 5% is produced by FDC Pharmaceuticals, India.

***Dosing Schedule:*** Both trial treatment arms will follow the same dosing schedule. Eye drops will be given hourly day and night for 48 hours, then hourly whilst awake for five days, and then two-hourly whilst awake for two further weeks. If the ulcer has healed (epithelial defect less than 1mm, infiltrate resolved, with or without corneal scarring), then treatment is stopped. If resolving stromal infiltration and/or epithelial defect >1mm but < 5mm, treatment is reduced to four times daily. If resolving but with epithelial defect > 5mm and/or stromal infiltration / hypopyon, treatment is reduced to six times daily. Treatment duration will be tailored to clinical response, with patients reviewed regularly whilst on treatment in addition to their scheduled study visits.

***Topical treatments:*** Several other topical medicines are used in the standard care of people with corneal infections:

1. **Fluorescein sodium ophthalmic strips** (Contacare Ophthalmics and Diagnostics, India) to highlight the area of cornea epithelial defect.
2. **Anaesthetic eye drops** to anaesthetise the cornea before procedures such as microbiology samples: Proxymetacaine 0.5% eye drop Minims (Bausch and Lomb, UK).
3. **Antibiotic eye drops** may be used as per the judgement of the treating ophthalmologist if there is a suspicion of mixed bacterial and fungal keratitis: Moxifloxacin 0.5% eye drops (Centaur Pharmaceuticals, India).



For peer review only - http://bmjopen.bmj.com/site/about/guidelines.xhtml

4. **Mydriatic eye drops** for pupil dilation to reduce discomfort from the infection: Cyclopentolate 2% eye drops (Aurolab, India), three times a day.
5. **Ocular hypotensive eye drops** if the intra-ocular pressure is elevated >25 mmHg. This will be at the discretion of the supervising clinician; usual first line treatment is timolol 0.5% eye drops (Allergan, India).

***Ancillary treatment in patients refractory to trial medication:*** In patients with progressive fungal keratitis despite 7 days or more of trial medication, additional treatment options will be considered and offered. After repeating microbiological tests to rule out a mixed bacterial infection, deteriorating patients are started on topical voriconazole 1% hourly if the ulcer is superficial only. For ulcers that are deeper than 75% of the corneal thickness, oral ketoconazole 200mg twice-daily is added (with monitoring of liver function). The choice of ancillary treatment is due to local availability. If, following 7 days of additional treatment, despite these measures there is ongoing progression, surgical management such as a TPK can be considered.

***Non-pharmacological treatment:*** It is sometimes necessary to perform surgical procedures during the management of corneal infections. In the context of the trial these will be performed by the supervising consultant ophthalmologist. These can include:

1. Insertion of a bandage contact lens for very small perforations.
2. Tissue glue and patch for small perforations
3. Corneal transplant (TPK) for progressive fungal infections that are refractory to medical management or large perforations.
4. Conjunctival flaps to cover corneas in non-healing ulcers.

**Outcome Measures**

***Primary Outcome Measure:***

The primary outcome will be best spectacle corrected visual acuity (BSCVA) in logMAR units at three months. This will be measured by a trial-certified optometrist, independent of all other aspects of the study and masked to allocation. This has been the primary outcome of several other trials and will therefore facilitate comparison. Three months is the time at which clinical experience suggests most corneal ulcers have usually healed. BSCVA is chosen as it is easy to measure and is of functional significance. We will measure the BSCVA using an LED-backlit, Tumbling-E LogMAR chart (Good-Lite Co., IL, USA) under controlled conditions. We will also measure vision using Peek Acuity, a validated smartphone application, in situations where the patient is unable to attend the hospital for follow-up and outcome data is only available through a domiciliary visit. [35] For patients who have CF vision or less, pre-defined logMAR values will be assigned based on previous clinical trials. [36,37] Similarly, for patients who undergo a corneal transplant (therapeutic penetrating keratoplasty, TPK), a pre-defined visual acuity of 1.9 logMAR will be given, or last observation carried forward (LOCF, whichever is the better vision), as per previous studies. [6]



For peer review only - http://bmjopen.bmj.com/site/about/guidelines.xhtml

***Secondary Outcome Measures:***

We will be assessing a number of secondary outcome measures that relate to different measures of visual acuity such as Peek Acuity; clinical signs of healing such as reduction of epithelial defect; microbiological culture rates; and other clinical outcome measures such as scar size or perforation rate. These secondary outcomes together with their analyses are outlined in the Analysis Plan below.

**Outcome Assessments**

Participants will be re-assessed at 2-days, 1-week, 2-weeks, 3-weeks, 2-months and 3-months following enrolment. Additional examinations, outside the trial protocol schedule may be conducted by the supervising clinician as indicated. The specific assessments to be carried out at each visit are indicated in Table 3. These will be conducted in the same way described for the baseline assessment as described (Table 2). At each follow-up assessment the participant will be asked about adherence to treatment and symptoms, including side effects, if still receiving trial treatment. This will be recorded in the Case Record File. To monitor trial medication adherence, the study participants will be asked to bring their eye drop bottles to the 1, 2- and 3-week follow-ups. The amount of remaining medication will be measured by weighing the bottle and will be compared with the anticipated remaining amount provided the drops were being used as instructed, resulting in a ratio of actually remaining to what is anticipated. Participants will be resupplied with medication as needed. The presenting visual acuity will be measured on each visit. In addition, the BSCVA will also be measured at the three-month follow-up (primary outcome measure). At the final 3-month follow-up the three quality of life questionnaires will be repeated. On each occasion the eyes will be examined using a slit-lamp and the cornea photographed. The *in vivo* confocal microscopy will be repeated at 1, 2 and 3 weeks to determine whether there is evidence of fungal hyphae resolution with the fragmentation of linear elements. At the 1-week follow-up if the ulcer has not healed it will be re-scraped for repeat culture to determine whether the keratitis is now culture-positive or culture-negative. We will give the study participants appointment cards for the next follow-up and they will be reminded about the follow-up a week prior to the date. Public transport costs will be paid for participants who are outpatients.

For peer review only

**Table 3: Baseline and follow-up assessment components.**

| Assessment Item | Baseline | Day 2 | Day 7 | Day 14 | Day 21 | Day 60 | Day 90 |
|---|---|---|---|---|---|---|---|
| History / Baseline questionnaire | X | | | | | | |
| Check treatment adherence | | X | X | X | X | X | X |
| Check for side effects | | X | X | X | X | X | X |
| Visual Acuity – Presenting | X | X | X | X | X | X | X |
| Visual Acuity – BSCVA | X | | | | | | X |
| Contrast Sensitivity | X | | | | | | X |
| Slit-lamp Examination | X | X | X | X | X | X | X |
| Cornea Photography | X | X | X | X | X | X | X |
| *In vivo* confocal microscopy | X | | X | X | X | | |
| Cornea samples (Microbiology/PCR) | X | | X | | | | |
| Quality of Life Tools | X | | | | | | X |

**Treatment Review**

At each follow-up visit participants will be reviewed by an ophthalmic clinician experienced in the management of fungal keratitis. Clinical responses to anti-fungal therapy tend to be relatively slow (compared to bacterial infections). Prolonged topical treatment courses of 4-6 weeks are usually needed. Therefore, a change in therapy is usually not made for at least the first week. Other interventions may be indicated such as application of glue to corneal perforations, conjunctival flaps or therapeutic penetrating keratoplasty (corneal transplant).

**Stopping Rules**

If the study eye develops any serious adverse outcomes, the antifungal study medication may be discontinued if it is considered to be responsible for the adverse event. The patient will then be treated at the discretion of the supervising ophthalmologist, without breaking the randomisation code. If the medication is stopped, the patient will continue with the scheduled follow-up examinations.

**Loss to Follow-up**

Loss to follow-up rates are expected to be low, based on clinical experience. Participants who do not present for their follow-up visit will be contacted by telephone. Reasons for the loss to follow-up will be identified. Patients will be counselled about the importance of attending for ongoing treatment and monitoring. If they are unwell or unable to attend the hospital for some specific reason the study team will arrange to visit them in their home. Reasons for loss to follow-up will be recorded and reported.

**Data collection, management, confidentiality and access to data**

Data will be collected using paper Clinical Record Forms (CRFs). These will be stored securely at SCEH and scanned electronic copies taken at the end of the day, stored on an encrypted drive with encrypted backups made daily both on and off-site. These data will be double entered into two separate MS Access databases. After double data entry has been completed data will be cleaned using EpiData version 3.1 software. Data

For peer review only

entry is supervised by the local study co-ordinator on a daily basis, with data collection and data entry progress being reviewed by the study co-ordinator and chief investigator at LSHTM on a weekly basis. Data protection and confidentiality is maintained through restricted access to the database system and cupboard where the paper documents are kept. Only authorised users will have access to the locked filing cabinet. The database will be password protected, with each data entry staff member having their own password. Data exports for further analysis will be anonymized.

**Data and Safety Monitoring Board**

The Data and Safety Monitoring Board (DSMB) for this trial includes independent experts in bioethics, biostatistics, epidemiology and ophthalmology appointed by the Trial Steering Committee and approved by the national regulatory authorities, before the start of the study. The DSMB meets at least twice each year and organises teleconferences as needed for progress reporting. The study protocol and modifications are subject to review and approval by ethics committees in Nepal and LSHTM, and by the DSMB. The DSMB will monitor any severe or unexpected events and oversees the data collected. The DSMB will be responsible for reviewing the results of the interim analysis and determining whether or not the trial should continue, with or without modifications.

**Monitoring for Harm**

Patients will be monitored at each visit for adverse events or reactions. We will follow standardised definitions for adverse events, adverse reactions, unexpected adverse reactions, serious adverse events or reactions, and suspected unexpected serious adverse reaction. These, along with the reporting scheme, are given in Appendix 2.

**Biological specimens**

The processing and analysis of biological specimens are detailed in Appendix 3.

**Sample Size Considerations**

The study is powered to test the hypothesis that CHX is non-inferior to NATA in terms of the primary outcome (BSCVA at 3 months) at a pre-specified non-inferiority margin (Delta) of 0.15 logMAR units. It is possible that CHX is non-inferior to NATA, given pilot RCT data that showed no statistically significant difference between CHX and NATA, with a Cochrane review finding a non-significant trend favouring CHX over NATA.[10,18,19] Despite this trend favouring CHX, we have chosen to carry out a non-inferiority trial rather than a superiority trial as this is a more clinically pragmatic approach. It would be counter-productive to conduct a superiority trial and find no statistically significant difference between the two treatments leading clinicians to potentially disregard CHX as a treatment, when in fact it may be 'non-inferior" to NATA, and be the only available or most cost-effective treatment.



For peer review only - http://bmjopen.bmj.com/site/about/guidelines.xhtml

For peer review only

The choice of Delta: This clinically meaningful difference of 0.15 logMAR was chosen as a difference of 0.15 logMAR corresponds to approximately 1.5 lines on a Snellen chart; any difference greater than this is clinically significant, as a difference of less than 0.15 log MAR could potentially be accounted for by testing / re-testing error.[35] Furthermore, it was used in the MUTT1 trial, providing methodological consistency between studies.[6] In addition, previous studies have suggested treated ulcers improve at a mean of four Snellen lines from baseline.

Sample size was calculated for 90% power and adjusted final alpha of 0.0492, taking account of a single interim analysis using O'Brien-Fleming approach to maintain type-1 error rate of 5%. A sample size of 452 is required to detect a non-inferiority margin of 0.15-logMAR in BSCVA 3-months after enrolment between arms, assuming 0.5 SD for 3-month BSCVA and 15% dropout. However, given approximately 10% of infections are mixed and these will be excluded from the primary analysis, 500 patients will be recruited. This sample size provides 90% power to detect superiority in BSCVA at 3-months if there is ≥0.17 LogMAR units difference as a secondary analysis.

**Analysis Plan**

The analysis will be by intention to treat. All patient data will be analysed according to their randomisation allocation irrespective of whether or not the patient received or adhered to the allocated treatment. CONSORT guidelines for analysing/reporting non-inferiority RCTs will be followed. A flowchart showing cases assessed, recruited and followed-up by arm will be prepared.[38] Baseline characteristics will be summarised by arm. The SPIRIT checklist is given in Appendix 4.

***Primary Outcome Analysis – unadjusted analysis:*** The primary analysis will be conducted using all available data, missing data due to loss to follow up will be excluded. The primary analysis of the primary outcome (BSCVA at 3 month) will be by linear regression, with treatment arm and baseline BSCVA as pre-specified covariates. This controls for the baseline BSCVA. The treatment group is the primary predictor. Primary analysis will exclude mixed fungal and bacterial infection (isolated in the baseline sample).

We will use our alpha of 0.0492 to test the null hypotheses at 0.0492 significance. The null hypothesis for non-inferiority is that the mean BSCVA at three months for chlorhexidine is greater than or equal to 0.15 logMAR worse than the BSCVA when natamycin is used. Significance will be assessed using a two-tailed test at 0.0492 level for assessing non-inferiority. CHX will be non-inferior if the upper one-sided 95% confidence level for this regression coefficient (i.e. the effect of CHX controlling for baseline BSCVA) exceeds 0.15 logMAR.

For peer review only

***Primary Outcome Analysis – adjusted analysis:*** In the event that there is a baseline imbalance between the treatment groups in a baseline covariate due to chance, we will perform an adjusted (sensitivity) analysis (see below). This is particularly important if CHX has a better outcome than NATA, as the adjusted treatment effects may account for this observed imbalance whilst the unadjusted analyses may not. Sensitivity analyses will allow us to show that any observed positive treatment effect is not solely explained by imbalances at baseline in any of the covariates.

***Secondary Analyses of the Primary Outcome***

Per-protocol analysis

Repeat analysis of the primary outcome will be done as per the protocol, based on what the participants actually took. The per-protocol population will include all the individuals included in the primary intention-to-treat (ITT) analysis, excluding individuals who showed poor compliance with the medications (defined as taking less than 50% through self-reporting or bottle weighing, whichever is the lower); individuals where there have been major protocol deviations; and non-fungal or mixed corneal infections (should these patients happened to have been randomised). All analyses that are performed as ITT will be repeated as per-protocol and labelled as such.

Mixed infections

Secondary analysis of the primary outcome (by intention to treat) will include mixed infections and will be carried out in the same way as in primary analysis of the primary outcome above.

Sensitivity analyses

For the primary analysis those individuals with missing outcome data (i.e. lost to follow up) will be excluded. Sensitivity analyses will be performed by imputing a range of scenarios to demonstrate a range of potential results, where there is missing outcome data. In the case of substantial missing data in the trial, the primary analysis will be carried out as previously stated excluding missing observations. This however assumes data are missing completely at random. As a sensitivity analysis, we will then apply a multiple imputation approach to the missing data, if we consider the data is randomly missing conditional on other observed covariates.

In the case that there is a systematic (i.e. non-random) reason for a difference in the follow-up rates between the two groups, we will explore models in which these missing outcome data are assumed to be non-random i.e. dependent on the outcome being regressed, BSCVA or treatment group.



For peer review only - http://bmjopen.bmj.com/site/about/guidelines.xhtml

We will also perform sensitivity analyses on patients whose vision is CF or worse, those patients who have undergone a TPK or those with corneal perforation, to see if there is any change in the effect size or conclusions drawn.

Analysis of Other Potential Determinants for Success

Logistic regression random effects models will be used to analyse potential factors that may be associated with a poor primary outcome, BSCVA at 3 months, defined as >1.0 logMAR. Individual baseline characteristics will be used separately as an exposure variable with BSCVA at 3 months as the outcome, with the model adjusted for trial arm. A multivariate model will be built using parameters with a p-value of < 0.2 in the log likelihood ratio test. Variables will be removed one by one, by omitting the variable with the largest p-value each time, until all predictors in the model have a p<0.05.

***Secondary Outcome Analysis:*** The secondary outcomes outlined above and detailed in Table 4 will be analysed by arm. Additional adjustment for factors imbalanced between arms at baseline will be introduced as appropriate. Continuous outcomes will be analysed using linear regression. Binary and ordinal outcomes will analysed using logistic regression. Details of these are given in Table 4.

For peer review only

**Table 4: Secondary Outcome Measures that will be investigated as part of the trial, together with analysis details**

| Secondary outcome measure | Details |
|---|---|
| Three-week BSCVA | We will analyse the secondary outcome of three-week BSCVA in logMAR in the same manner as the primary analysis of the primary outcome described above. The three-week BSCVA will include values taken between 18 days and 5 weeks, with the value closest to 3 weeks used. |
| Presenting VA by Peek | We will analyse the presenting VA by Peek Acuity with and without pinhole at three months as a secondary outcome. This will be of interest if we are unable to get reliable BSCVA measurements at 3 months (i.e. if patients fail to attend and we need to attend their houses for visual acuity testing). This will be performed in the same way as the primary analysis of the primary outcome. We will also perform a sensitivity analysis including those lost to follow-up, by using the most recent observation of this variable. |
| Scar/infiltrate size at 1 week, 3 weeks and 3 months by slit lamp examination. | The geometric mean of the two principle axes in mm of the scar or infiltrate at 1 week, 3 weeks and 3 months will be used as a secondary outcome variable. The slit-lamp scar size will be compared at each of these time points between treatment arm in the same manner as described above. This will be by linear regression, with treatment arm and baseline infiltrate / scar size as pre-specified covariates. This controls for the baseline infiltrate / scar size. |
| Time to full epithelial healing (slit lamp examination by ophthalmic clinician) | Time of re-epithelialisation will be defined as the midpoint between the last review where an epithelial defect (ED) was present and the subsequent review where there was no ED. An area of fluorescein staining of less than 0.5mm will be considered as a resolved ED due to the difficulty in differentiating a smaller defect from a small amount of fluorescein pooling observed in a healed defect.<br><br>Analysis of time to healing will use Cox proportional hazards regression with treatment group as the primary predictor and with predictors of baseline ED size (using the geometric mean in mm as outlined above). Survival curves will be plotted using Kaplan-Meier analysis for both treatment arms up to the final visit at three months. The proportional hazards assumption will be checked by stratifying on quartiles of the baseline ED size and if the assumption does not hold, the stratified results will be the ones reported. Additionally, treatment failure (defined as persisting epithelial defect greater than 0.5mm at the three-month review) will be compared between treatment groups using Fisher's exact test. |
| Rate of healing | We will assess how quickly the area of ulceration reduces over time. The rate will be calculated between the 1 week, 3 week and 3-month review by taking the difference in ED size between the two time points, in mm, and diving by the number of days to give a rate of mm/day. Analysis will be performed using Cox regression. |
| Microbiological cure | Patients who have a persisting corneal ulcer (as defined by the presence of an ED) at day 7 will undergo a repeat corneal scrape and microbiological investigations. Microbiological cure at 7 days will be defined as the absence of any micro-organisms as no significant growth on culture. The number of patients with microbiological cure at day 7 will be compared between the two treatment arms using logistic regression with treatment group and organism (Aspergillus spp., Fusarium spp., or other) as covariates. |
| Ulcer depth at 1 week and 3 weeks (slit lamp examination by ophthalmic clinician). | The depth of ulcer in terms of percentage of healthy cornea will be compared at 1 week and 3 weeks between treatment arms, adjusting for baseline depth in the same manner with analysis performed by linear regression |
| Hypopyon height at 1 and 3 weeks, (slit lamp examination by ophthalmic clinician) | The hypopyon height in mm will be compared at 1 week and 3 weeks between treatment arms, adjusting for baseline hypopyon height in the same manner with analysis performed in the same way (linear regression) |
| Perforation and/or TPK and/or conjunctival advancement by three months (slit lamp examination by ophthalmic clinician) | The number of patients who undergo perforation and/or require TPK and/or have undergone conjunctival advancement by three months will be reported using confidence intervals and descriptive statistics. The study is not powered to detect a difference in perforation rate or TPK between treatment groups; not reporting a significant difference may be wrongly interpreted as there being no difference between groups. We will therefore perform an exploratory analysis to compare TPK |



For peer review only - http://bmjopen.bmj.com/site/about/guidelines.xhtml

| | |
|---|---|
| | or perforation rates between treatment groups. This will be by logistic regression to compute an odds ratio by arm. |
| Loss of Eye | The number of patients who have their eye surgically removed (evisceration or enucleation) during the 3 month follow-up period will be reported using confidence intervals and descriptive statistics in the same way as for TPK / perforation rate above, along with exploratory analysis using logistic regression to find risk factors for eye loss and odds ratio for this by arm. |
| Ocular adverse effects, slit lamp examination by ophthalmic clinician. | The proportion of patients with one or more adverse events will be compared using Fisher's Exact test. Additional analysis to compare the rate of adverse events during the three month follow-up will be by Poisson regression as this can take into account multiple instances within one participant. |
| Quality of life assessed using: EQ-5D, WHO/PBD-VF20, WHOQOL-BREF | QoL can be assessed quantitatively using different tools depending on what is of interest. For example, disease-related QoL can be assessed (e.g. vision related QoL, VRQoL) or more general health-related issues irrespective of the disease can be investigated (health-related QoL, HRQoL).[39]<br><br>We will use the WHO/PBD-VF20 (World Health Organisation/ Prevention of Blindness and Deafness—Visual Functioning 20 item questionnaire) VRQoL tool. This tool measures the impact of visual impairment in the person's life including mental wellbeing, dependency and social functioning. These have been used in a number of other visual related studies to show a difference in QoL. [32,33]<br><br>For HRQoL, we will use the EQ-5D questionnaire, EQ-Visual Analogue Scale and the WHOQOL-BREF. The EQ-5D is a standardised tool to measure health outcomes.[34] The WHOQOL-BREF has good applicability in (LMIC as it was developed simultaneously from concept across 18 countries in Africa, Asia and Latin America. It measures 4 domains of health: Physical Health, Psychological Health, Social Relationships, and Environment.<br><br>QoL scoring tools are presented in the Online Supplementary Material, Appendix 5.<br><br>Analysis will be by comparing the scores obtained for each QoL assessment for the two treatment arms to estimate the effect of CHX and NATA on patients' quality of life. This will be similar to that performed by Habtamu et al. [39] Comparisons between the two medication groups will be adjusted for the matching variables: age and sex. The VRQoL analysis was also adjusted for socio-economic status and the HRQoL analysis adjusted for both socio-economic status and presence of health problems during the previous four weeks, as these factors may confound the association between fungal keratitis and QoL. Logistic, linear and ordinal logistic regression methods will be used for binary, continuous and ordered categorical outcome variable analysis, respectively. Linear regression models and the t-test were employed to compare significant differences in QoL scores and to generate mean and mean differences between the two treatment arms in each QoL subscale and domain, respectively. |
| Cost effectiveness analysis, using EQ-5D data from 3 months and direct cost data | Direct cost data will be collected at the three-month follow-up. Economic costs to the patient can also be calculated from the EQ-5D questionnaire, which will be asked at baseline and at the three-month follow-up. Mean direct costs incurred by patients will be compared between interventional arms using the t-test for significance. The difference from the baseline EQ-5D and the three-month EQ-5D mean scores will also be compared in a similar fashion. |
| Drug adherence | The rate of drug adherence will be compared between the two treatment groups using descriptive statistics. |

For peer review only

***Interim Analysis:*** An interim analysis will be conducted for the DSMB by an independent statistician after 1/3 of patients recruited have completed follow-up.

**Patient and public involvement**

Mixed methods descriptive cross-sectional studies with semi-structured interviews and focus group discussions with patients and eye health providers were carried out in Sagarmatha zone, Eastern Nepal at various points in 2018.[40] These conversations highlighted the delayed presentation often seen with fungal keratitis combined with the often prohibitively high costs of treatment. Furthermore, treatment is not always felt to be effective. Eye health workers were keen to receive further training and highlighted the need for greater government support in the provision of eye care services in the community.

**ETHICS AND DISSEMINATION**

Ethics committee and regulatory review and approval has been obtained from the Nepal Health Research Council (NHRC) Ethics Committee, Kathmandu, Nepal; the Department of Drug Administration (DDA), Kathmandu, Nepal; and the London School of Hygiene and Tropical Medicine Ethics Committee, UK. The trial is registered with the ISRCTN clinical trials registry (ISRCTN14332621). Protocol modifications are submitted to the relevant parties for review and/or approval. Table 5 summarises the study protocol and trial registration information. At the end of the study period, patients who still require treatment or follow-up will continue to be treated at SCEH as per routine clinical practice. The trial Sponsor is the London School of Hygiene and Tropical Medicine. The results of this trial will be presented at local and international meetings and submitted to peer-reviewed journals for publication.

For peer review only



For peer review only - http://bmjopen.bmj.com/site/about/guidelines.xhtml

**Table 5: Registration Data and Protocol Summary**

| **Data category** | **Information** |
|---|---|
| Primary registry and trial identifying number | ISRCTN Registry; ISRCTN14332621 |
| Date of registration in primary registry | 15 May 2019 |
| Secondary identifying numbers | |
| Source(s) of monetary or material support | Wellcome Trust |
| Primary sponsor | London School of Hygiene and Tropical Medicine (LSHTM) |
| Secondary sponsor(s) | |
| Contact for queries | Jeremy Hoffman FRCOphth (Jeremy.hoffman@lshtm.ac.uk) |
| Title | Chlorhexidine 0.2% vs Natamycin 5% for the treatment of fungal corneal infections |
| Countries of recruitment | Nepal |
| Health condition(s) or problem(s) studied | Fungal keratitis |
| Intervention(s) | Participants will be randomised to either topical chlorhexidine 0.2% or topical natamycin 5% |
| Key eligibility criteria | 1. Acute MK characterised by:<br>• Corneal epithelial ulceration >1mm diameter<br>• Corneal stromal infiltrate<br>• Acute inflammation: e.g. conjunctival injection, anterior chamber inflammatory cells, hypopyon.<br>2. Filamentous fungal hyphae visualised on smear microscopy and/or *in vivo* confocal microscopy.<br>3. Agree to be randomised to either treatment arm and able to give informed consent<br>4. Agree to be followed up at 2 days, 1 week, 2 weeks, 3 weeks, 2 months and 3 months<br>5. Adults (18 years and older) |
| Study type | Randomised controlled trial |
| Date of first enrolment | 1 June 2019 |
| Target sample size | 500 |
| Recruitment status | Recruiting |
| Primary outcome(s) | Best Spectacle Corrected Visual Acuity (BSCVA) at 3 months by a trial certified optometrist |
| Key secondary outcomes | 1. Time to full epithelial healing (slit lamp examination by ophthalmic clinician).<br>2. Pin-hole visual acuity in logMAR at 3 months, trial-certified optometrist<br>3. Scar/infiltrate size at 1 week, 3 weeks and 3 months (slit lamp examination by ophthalmic clinician).<br>4. Ulcer depth at 1 week and 3 weeks (slit lamp examination by ophthalmic clinician).<br>5. Hypopyon height at 1 and 3 weeks, (slit lamp examination by ophthalmic clinician).<br>6. Perforation and/or TPK by three months (slit lamp examination by ophthalmic clinician).<br>7. Positive culture rate at 1 week<br>8. Ocular adverse effects at each follow up visit (Day 2, Day 7, Day 14, 3 weeks, 2 months, 3 months), slit lamp examination by ophthalmic clinician.<br>9. Quality of life (QoL) assessed using: EQ-5D, WHO/PBD-VF20, WHOQOL-BREF (comparison between baseline and QoL measures at 3 months)<br>10. Cost-effectiveness analysis, using EQ-5D data from 3 months and direct cost data.<br>11. Drug adherence at each follow up visit (Day 2, Day 7, Day 14, 3 weeks, 2 months, 3 months) whilst the patient is using study medications. |

For peer review only

**ACKNOWLEDGEMENTS**

The authors would like to thank the Eastern Region Eye Care Programme (EREC-P), Nepal Netra Jyoti Sangh (NNJS) and the Nepal Health Research Council (NHRC) for helping with study co-ordination and implementation. The authors would like to thank the staff and management board at Sagarmatha Choudhary Eye Hospital (SCEH) for their continued support, co-ordination and implementation of the study. The authors are grateful to the guidance of the Data Safety Monitoring Board including Reeta Gurung (chair), Salma KC Rai, Sabina Shrestha and Meenu Chaudhary.

**COLLABORATORS**

SCEH:

Abhishek Roshan (Hospital Manager); Sanjay Kumar Singh (Medical Superintendent); Reena Yadav (Primary Investigator); Sandip Das Sanyam (Study Co-Ordinator); Pankaj Chaudhary (Microbiologist); Rabi Shankar Sah, Kamlesh Yadav (Investigators); Ram Narayan Bhandari, Aasha Chaudhary, Sharban Mandal (Eye Health Workers); Raja Ram Mahato (Randomisation Administrator and Logistics); Lalita Rajbanshi (Laboratory Assistant); Ramesh Sah, Arvind Ray, Sachindra Kamti (Optometrists); Avinash Chaudhary (Ophthalmic Assistant); Padma Narayan Chaudhary (Hospital Chairman); Suresh Singh, Ravi Pant, Rakesh Singh (Hospital Management); Ram Kumar Jha (Ophthalmic Assistant, Rajbiraj ECC)

NNJS:

Sailesh Kumar Mishra (Executive Director); Sabita KC (Board Secretary); Ranjan Shah (Programme Associate); Jaganath Dhital (Assistant)

EREC-P:

Sanjay Kumar Singh (Programme Director)

JEH:

Hemchandra Jha (Medical Superintendent); Mahesh Yadav (Investigator); Rudal Prasad Sah (Ophthalmic Assistant)

LSHTM:

Jeremy Hoffman (Primary Investigator); Matthew Burton (Chief Investigator); Astrid Leck (Microbiologist); David Macleod, Helen Weiss (Statisticians); Victor Hu (Investigator); Sarah O'Regan (Administrator)

For peer review only



For peer review only - http://bmjopen.bmj.com/site/about/guidelines.xhtml

**FUNDING STATEMENT**

This research was funded through a Senior Research Fellowship to MJB from the Wellcome Trust (207472/Z/17/Z). The funders had no role in study design, data collection and analysis, decision to publish, or preparation of the manuscript.

**COMPETING INTERESTS STATEMENT**

All authors declare there are no conflict of interests.

**DATA STATEMENT**

The datasets generated during and/or analysed during the current study will be available upon request from Prof Matthew Burton (matthew.burton@lshtm.ac.uk). The full data set will be available with all patient identifiable details removed. Data will be available after formal reporting of the study findings in a peer-reviewed scientific publication. Datasets will only be available to bona fide scientific investigators. Requests should be made to the Chief Investigator in writing detailing the scientific investigators background and intended use for the data. Consideration will be given to all proposed analyses, with likely envisaged uses including investigators planning on conducting meta-analyses for example. Patient Information Sheets and consent forms specifically referenced making anonymised data available and this has been approved by the relevant ethic committees.

**AUTHOR CONTRIBUTIONS**

Searched the literature: JJH, MJB

Drafted initial protocol: JJH

Contributed to protocol development and revision: JJH, RY, SD, PC, AR, SKS, SA, EM, DM, HAW, AL, VHH, MJB

Drafted this manuscript: JJH

Critically revised this manuscript: JJH, RY, SD, PC, AR, SKS, SA, EM, DM, HAW, AL, VHH, MJB

Conceptualization: MJB

Funding acquisition: MJB

For peer review only

**APPENDICES:**

Appendix 1 - Informed consent material

Appendix 2 – Monitoring for Harm

Appendix 3 - Biological specimens

Appendix 4 – SPIRIT Checklist

Appendix 5 – Quality of Life Scoring Tools

For peer review only

**LEGENDS FOR FIGURES**

**Figure 1: Fungal keratitis and corneal scarring.** (A) Active fungal keratitis with signs of acute inflammation and corneal ulceration. Photograph taken at presentation to SCEH. (B) Corneal Scar, the blinding sequela of a resolved episode of fungal keratitis. Photograph taken at two months following presentation (same patient as (A)).

**Figure 2: Progressive fungal keratitis.** (A) Early filamentous fungal keratitis; started immediately on intensive topical antifungal treatment (Natamycin 5%). (B) The same case one week later, unresponsive to intense Natamycin 5% treatment, with progression of the infection.

**Figure 3: Overview of the clinical trial.** Microbial keratitis is defined as presence of corneal epithelial ulceration (>1mm in diameter), corneal stromal infiltrate and signs of acute inflammation (e.g. conjunctival injection, anterior chamber inflammatory cells, hypopyon). Fungal elements to be detected by smear microscopy and/or confocal microscopy. Those eligible will be randomised 1:1 to CHX or NATA (n = 500). BSCVA: Best Spectacle Corrected Visual Acuity; TPK: Therapeutic Penetrating Keratoplasty.

For peer review only

**REFERENCES**

1 Burton MJ, Pithuwa J, Okello E, *et al.* Microbial Keratitis in East Africa: Why are the Outcomes so Poor? *Ophthalmic Epidemiol* 2011;**18**:158–63. doi:10.3109/09286586.2011.595041

2 Thomas PA, Leck AK, Myatt M. Characteristic clinical features as an aid to the diagnosis of suppurative keratitis caused by filamentous fungi. *British Journal of Ophthalmology* 2005;**89**:1554–8. doi:10.1136/bjo.2005.076315

3 Leck AK, Thomas PA, Hagan M, *et al.* Aetiology of suppurative corneal ulcers in Ghana and south India, and epidemiology of fungal keratitis. *British Journal of Ophthalmology* 2002;**86**:1211–5. doi:10.1136/bjo.86.11.1211

4 Bongomin F, Gago S, Oladele RO, *et al.* Global and Multi-National Prevalence of Fungal Diseases-Estimate Precision. *J Fungi (Basel)* 2017;**3**:57. doi:10.3390/jof3040057

5 Ali Shah SI, Shah SA, Rai P, *et al.* Visual outcome in patients of keratomycosis, at a tertiary care centre in Larkana, Pakistan. *J Pak Med Assoc* 2017;**67**:1035–8.

6 Prajna NV, Krishnan T, Mascarenhas J, *et al.* The mycotic ulcer treatment trial: a randomized trial comparing natamycin vs voriconazole. *JAMA Ophthalmol* 2013;**131**:422–9.

7 Prajna NV, Krishnan T, Rajaraman R, *et al.* Effect of Oral Voriconazole on Fungal Keratitis in the Mycotic Ulcer Treatment Trial II (MUTT II): A Randomized Clinical Trial. *JAMA Ophthalmol* 2016;**134**:1365–72. doi:10.1001/jamaophthalmol.2016.4096

8 Galarreta DJ, Tuft SJ, Ramsay A, *et al.* Fungal keratitis in London: microbiological and clinical evaluation. *Cornea* 2007;**26**:1082–6. doi:10.1097/ICO.0b013e318142bff3

9 Ong HS, Fung SSM, Macleod D, *et al.* Altered Patterns of Fungal Keratitis at a London Ophthalmic Referral Hospital: An Eight-Year Retrospective Observational Study. *Am J Ophthalmol* 2016;**168**:227–36. doi:10.1016/j.ajo.2016.05.021

10 FlorCruz NV, Evans JR. Medical interventions for fungal keratitis. *Cochrane Database Syst Rev* 2015;**4**:CD004241. doi:10.1002/14651858.CD004241.pub4

11 Schein OD. Evidence-Based Treatment of Fungal Keratitis. *JAMA Ophthalmol* 2016;**134**:1372–3. doi:10.1001/jamaophthalmol.2016.4167

12 Arora R, Gupta D, Goyal J, *et al.* Voriconazole versus natamycin as primary treatment in fungal corneal ulcers. *Clin Experiment Ophthalmol* 2011;**39**:434–40. doi:10.1111/j.1442-9071.2010.02473.x

13 Prajna NV, Mascarenhas J, Krishnan T, *et al.* Comparison of natamycin and voriconazole for the treatment of fungal keratitis. *Arch Ophthalmol* 2010;**128**:672–8. doi:10.1001/archophthalmol.2010.102

14 Sharma S, Das S, Virdi A, *et al.* Re-appraisal of topical 1% voriconazole and 5% natamycin in the treatment of fungal keratitis in a randomised trial. *Br J Ophthalmol* 2015;**99**:1190–5. doi:10.1136/bjophthalmol-2014-306485

15 McDonnell G, Russell AD. Antiseptics and disinfectants: activity, action, and resistance. *Clinical Microbiology Reviews* 1999;**12**:147–79.



For peer review only - http://bmjopen.bmj.com/site/about/guidelines.xhtml

16 Shariff JA, Lee KC, Leyton A, *et al.* Neonatal mortality and topical application of chlorhexidine on umbilical cord stump: a meta-analysis of randomized control trials. *Public Health* 2016;**139**:27–35. doi:10.1016/j.puhe.2016.05.006

17 Zhou J, Hu B, Liu Y, *et al.* The efficacy of intra-alveolar 0.2% chlorhexidine gel on alveolar osteitis: a meta-analysis. *Oral Dis* 2017;**23**:598–608. doi:10.1111/odi.12553

18 Rahman MR, Johnson GJ, Husain R, *et al.* Randomised trial of 0.2% chlorhexidine gluconate and 2.5% natamycin for fungal keratitis in Bangladesh. *British Journal of Ophthalmology* 1998;**82**:919–25. doi:10.1136/bjo.82.8.919

19 Rahman MR, Minassian DC, Srinivasan M, *et al.* Trial of chlorhexidine gluconate for fungal corneal ulcers. *Ophthalmic Epidemiol* 1997;**4**:141–9. doi:10.3109/09286589709115721

20 Dart JKG, Saw VPJ, Kilvington S. Acanthamoeba keratitis: diagnosis and treatment update 2009. *Am J Ophthalmol* 2009;**148**:487–499.e2. doi:10.1016/j.ajo.2009.06.009

21 Kosrirukvongs P, Wanachiwanawin D, Visvesvara GS. Treatment of acanthamoeba keratitis with chlorhexidine. *Ophthalmology* 1999;**106**:798–802. doi:10.1016/S0161-6420(99)90169-0

22 Seal D, Hay J, Kirkness C, *et al.* Successful medical therapy of Acanthamoeba keratitis with topical chlorhexidine and propamidine. *Eye* 1996;**10 ( Pt 4)**:413–21. doi:10.1038/eye.1996.92

23 Martin MJ, Rahman MR, Johnson GJ, *et al.* Mycotic keratitis: susceptibility to antiseptic agents. *Int Ophthalmol* 1995;**19**:299–302.

24 Geffen N, Norman G, Kheradiya NS, *et al.* Chlorhexidine gluconate 0.02% as adjunct to primary treatment for corneal bacterial ulcers. *Isr Med Assoc J* 2009;**11**:664–8.

25 Upadhyay MP, Karmacharya PC, Koirala S, *et al.* The Bhaktapur eye study: ocular trauma and antibiotic prophylaxis for the prevention of corneal ulceration in Nepal. *British Journal of Ophthalmology* 2001;**85**:388–92.

26 Chidambaram JD, Prajna NV, Larke N, *et al.* In vivo confocal microscopy appearance of Fusarium and Aspergillus species in fungal keratitis. *Br J Ophthalmol* 2017;**101**:1119–23. doi:10.1136/bjophthalmol-2016-309656

27 Chidambaram JD, Prajna NV, Larke NL, *et al.* Prospective Study of the Diagnostic Accuracy of the In Vivo Laser Scanning Confocal Microscope for Severe Microbial Keratitis. *Ophthalmology* 2016;**123**:2285–93. doi:10.1016/j.ophtha.2016.07.009

28 Vaddavalli PK, Garg P, Sharma S, *et al.* Role of confocal microscopy in the diagnosis of fungal and acanthamoeba keratitis. *Ophthalmology* 2011;**118**:29–35. doi:10.1016/j.ophtha.2010.05.018

29 Habtamu E, Bastawrous A, Bolster NM, *et al.* Development and Validation of a Smartphone-based Contrast Sensitivity Test. *Transl Vis Sci Technol* 2019;**8**:13. doi:10.1167/tvst.8.5.13

30 Somannavar S, Ganesan A, Deepa M, *et al.* Random capillary blood glucose cut points for diabetes and pre-diabetes derived from community-based opportunistic screening in India. *Diabetes Care* 2009;**32**:641–3. doi:10.2337/dc08-0403

31 World Health Organization. Prevention of Blindness & Deafness. Consultation on Development of Standards for Characterization of Vision Loss and Visual Functioning. In: *WHO / PBL*. Geneva: 2003.


For peer review only

32 Polack S, Kuper H, Mathenge W, *et al.* Cataract visual impairment and quality of life in a Kenyan population. *British Journal of Ophthalmology* 2007;**91**:927–32. doi:10.1136/bjo.2006.110973

33 Polack S, Kuper H, Wadud Z, *et al.* Quality of life and visual impairment from cataract in Satkhira district, Bangladesh. *Br J Ophthalmol* 2008;**92**:1026–30. doi:10.1136/bjo.2007.134791

34 EuroQol Research Foundation. EQ-5D-3L User Guide. 2018. https://euroqol.org/publications/user-guides

35 Bastawrous A, Rono HK, Livingstone IAT, *et al.* Development and Validation of a Smartphone-Based Visual Acuity Test (Peek Acuity) for Clinical Practice and Community-Based Fieldwork. *JAMA Ophthalmol* 2015;**133**:930–8. doi:10.1001/jamaophthalmol.2015.1468

36 Barron BA, Gee L, Hauck WW, *et al.* Herpetic Eye Disease Study. A controlled trial of oral acyclovir for herpes simplex stromal keratitis. *Ophthalmology* 1994;**101**:1871–82.

37 Wilhelmus KR, Beck RW, Moke PS, *et al.* Acyclovir for the prevention of recurrent herpes simplex virus eye disease. Herpetic Eye Disease Study Group. *N Engl J Med* 1998;**339**:300–6. doi:10.1056/NEJM199807303390503

38 Schulz KF, Altman DG, Moher D, *et al.* CONSORT 2010 statement: updated guidelines for reporting parallel group randomised trials. BMJ. 2010;**340**:c332–2. doi:10.1136/bmj.c332

39 Habtamu E, Wondie T, Aweke S, *et al.* The Impact of Trachomatous Trichiasis on Quality of Life: A Case Control Study. *PLoS Negl Trop Dis* 2015;**9**:e0004254. doi:10.1371/journal.pntd.0004254

40 Burn H, Puri L, Roshan A, *et al.* Primary Eye Care in Eastern Nepal. *Ophthalmic Epidemiol* 2019;**22**:1–12. doi:10.1080/09286586.2019.1702217

For peer review only

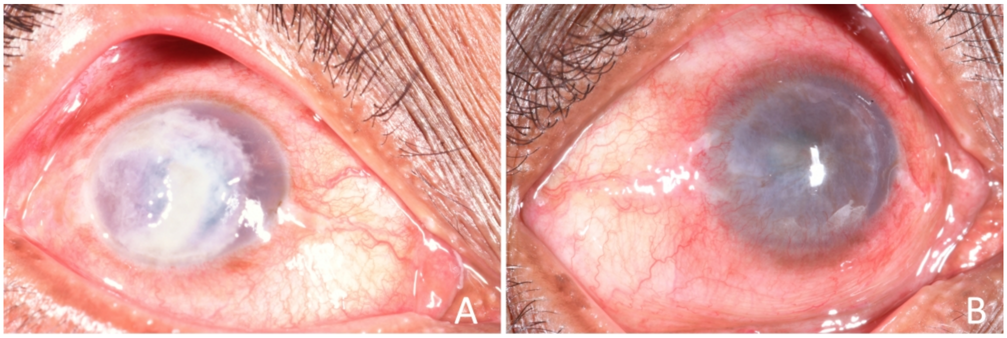


Figure 1: Fungal keratitis and corneal scarring. (A) Active fungal keratitis with signs of acute inflammation and corneal ulceration. Photograph taken at presentation to SCEH. (B) Corneal Scar, the blinding sequela of a resolved episode of microbial keratitis. Photograph taken at two months following presentation (same patient as (A)).

325x107mm (300 x 300 DPI)

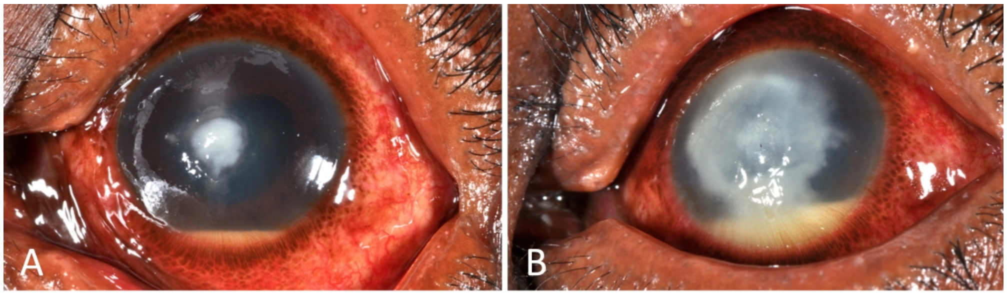


Figure 2: Progressive fungal keratitis. (A) Early filamentous fungal keratitis; started immediately on intensive topical antifungal treatment (Natamycin 5%). (B) The same case one week later, unresponsive to intense Natamycin 5% treatment, with progression of the infection.

325x93mm (300 x 300 DPI)

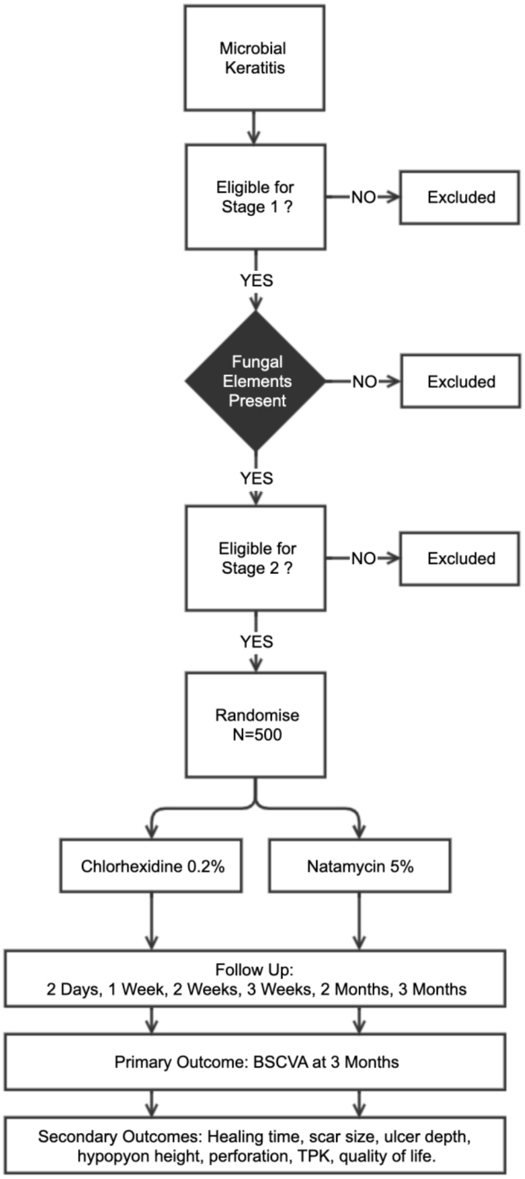


Figure 3: Overview of the clinical trial. Microbial keratitis is defined as presence of corneal epithelial ulceration (>1mm in diameter), corneal stromal infiltrate and signs ofacute inflammation (e.g. conjunctival injection, anterior chamberinflammatory cells, hypopyon). Fungal elements to be detected by smear microscopy and/or confocal microscopy. Those eligible will be randomised 1:1 to CHX or NATA (n = 500). BSCVA: Best Spectacle Corrected Visual Acuity; TPK: Therapeutic Penetrating Keratoplasty.

87x182mm (300 x 300 DPI)

# Appendix 1: Informed Consent Material

## Study Title: Randomised controlled trial of topical chlorhexidine 0.2% versus topical natamycin 5% for fungal keratitis in Nepal

*Participant Information Sheet and Consent Form - No 1:*
*For baseline assessment and investigations*

**Introduction**
You are being invited to take part in a medical research study. Before you decide it is important for you to understand why the research is being done and what it will involve. Please take time to read or listen to the following information carefully and to talk to others about the study, if you wish. Ask us if there is anything that is not clear or if you would like more information. Do not sign the consent form unless you are satisfied with the answers to your questions and decide that you want to be part of this study. Take time to decide whether or not you wish to take part.

**Why have I been invited?**
You have been invited to take part because you have a corneal infection. To guide the treatment of this it is helpful to do tests to find out the cause, to guide the choice of eye drop treatment. We are asking for your permission to carry out some tests that are listed below to find out what is causing the infection and to decide if you may be suitable for a trial comparing treatments for fungal eye infections.

**What is this study about?**
Infection of the clear part of the front of the eye (the cornea) is a corneal ulcer. It is an important cause of blindness in Nepal. A scratch in the cornea allows infection to enter and an ulcer to begin. These infections can be very serious with some people losing the sight in the affected eye.

Different types of infectious organisms can cause corneal ulcers. These include bacteria and fungi which need to be treated with different types of eye drop medicines. To find out what type of infection is present it is necessary to collect a small sample from the ulcer and perform various laboratory tests. The test results guide the treatment. In Nepal about half of the infections are caused by fungus.

Fungal infections can be difficult to diagnose. No single test can reliably detect it. It may need several different tests to find it. Currently, in many countries where fungal ulcers are common laboratory services are limited or not available. Tests are expensive and may not detect all of the infections. There is a need to understand which of the current test options give the most useful information.

The first purpose of this study is to carefully evaluate your eye infection using several different diagnostic tests to try to find out what type of infection you have, and to learn which tests are most useful in this process.

There is a need for alternative, affordable and more easily available eye drop treatments for fungal infections. We are also conducting a clinical trial to compare two eye drop treatments for fungal infection, to try to find out which is better.

Therefore, the second purpose of this initial Assessment and Investigation study is to identify people who have a fungal corneal infection who may be eligible and willing to enrol in the clinical trial. If we find out that you do have a fungal corneal infection, we will then ask if you would be interested in hearing about the clinical trial. If you are interested, then at that stage we will explain about the treatment trial in detail, provide you with a second information sheet and go throw a second consent process.

**Do I have to take part?**
No. Your involvement is entirely voluntary. If you agree to take part, we will then ask you to sign a consent form. If you decide to join and change your mind, you are free to withdraw at any time without giving a reason. This will not affect the standard of care you receive.
Why have I been invited?
You have been invited to take part because you have a corneal infection. To guide the treatment of this it is helpful to do tests to find out the cause, to guide the choice of eye drop treatment. We are asking for your permission to carry out some tests that are listed below to find out what is causing the infection and to decide if you may be suitable for a trial comparing treatments for fungal eye infections.

For peer review only

**What will happen to me if I take part?**
If you agree to be part of this study, the following will happen:

- We will ask you a series of questions. This will include basic demographic information, the history of your current eye problem, and treatment you have had before arriving at the hospital.

- We will then carefully examine both eyes using a special microscope.

- Your eyes will be photographed with a camera. This is additional to standard care, and will help us to monitor the infection and the response to treatment.

- We will use a special microscope to look at the cornea to see if we can find a fungal infection. This involves putting anaesthetic drops on the eye. A soft plastic device then gently touches the eye so to see if you have a fungal infection. This is additional to standard care, and will help us to find out the type of infection you have more rapidly so that we can offer you the most appropriate treatment.

- We will collect samples form the corneal ulcer to test in the laboratory to try to identify what is causing the infection. Anaesthetic eye drops will be used numb the eye so you will not feel anything while we collect the sample by brushing the surface.
-
- We will check your blood sugar level for diabetes. This is done using a finger-prick blood sample. If this is raised, we will refer you to a separate group of doctors to help you with this.

- You will be offered a test for HIV infection. This will be done through the hospitals counselling and testing services. If you choose to accept this, then you will be separately counselled about the test and the implications of the results. This would involve the collection of a blood test sample from your arm. If the HIV test is positive, then you will be referred to the appropriate team for ongoing care. The result of this test will be shared with the us (cornea infection study team), with your consent, as it is potentially relevant to the treatment of your cornea infection.

- We will collect a sample of the cells from the inside of your cheek by gently rubbing a swab for a few seconds. This is additional to the standard of care. The purpose is to try to understand how the body fights the infection and why some people develop this eye problem and others do not.

- You will be asked to complete three short questionnaires to assess your quality of life and vision function. This is additional to standard care, and will help us to better understand the impact corneal ulcers can have on people's lives.

If we find evidence of fungus infecting your eye, then we will discuss the details of the trial with you further. At that point we will give you a second information sheet to read (or read to you), and if you meet all the eligibility criteria for the trial, to then go through a second consent process with you.

If you do not have a fungal infection, for example if the infection is caused by bacteria, then your will receive standard ongoing treatment for this by the same team at the hospital.

You may be withdrawn from the study without your consent if the researchers believe it is in your best interest or if you fail to follow study procedures.

**What are the side effects or risks of taking part?**
- The anaesthetic eye drops usually sting for a few seconds and then your eye should feel more comfortable. There is a very low risk of allergic reaction from the anaesthetic eye drops.
- The procedures including the history taking, examination, photography, in vivo confocal microscopy and sample collection are part of the standard of care for assessing a corneal infection. The risk associated with these are very low and no different from routine care. To minimise discomfort, topical anaesthetic will be given before examinations and sample collection.
- You will experience a little pain when a blood sample is collected. The people collecting the blood sample will ensure that this is done with as little discomfort as possible.
- The questions, tests and examinations will add about 15 minutes to your hospital visit above what we would usually expect in assessing a patient with this problem.

**What are the benefits of taking part?**
- The study will involve tests for the type of infection. This helps the doctor looking after you to choose the best type of treatment for your eyes
- The costs for the clinical assessment and the tests will be paid for by the study.
- By participating in this study, you will be helping to further research into this field so more informed decisions can be made when treating people with corneal infection.

For peer review only

**What will happen to the clinical records, photographs and test results?**
Your records will remain strictly confidential at all times. The information will be held in a secure office at your treating hospital. Only the people organizing or supervising the trial and regulatory authority auditors will have access to it. These include officials delegated by the Sponsor (London School of Hygiene and Tropical Medicine), the local National Ethics Committee, The local National Drug Regulatory Authority and trial Data Safety Monitoring Body (DSMB).

Your name will not be passed to anyone else outside the research team, unless we have your direct instruction to do so, for example to make a medical referral.

The photographs of the eye and the result of the laboratory test for infection will be shared with computer engineers to help develop a programme that can automatically analyse the image to see if this can provide some indication of the cause of the infection. Images of corneal infection may also be used for educational and teaching purposes, including in publications. All personal identifying information will be removed before sharing images.

**What tests will we do on the sample?**
The samples collected from the surface of your eye will be tested in several different ways to determine what is causing the infection. This work will be done in the hospital microbiology laboratory, where you are being treated. A portion of the infection sample will be transferred for additional special tests at KCMC Hospital Biotechnology (Tanzania), the London School of Hygiene and Tropical Medicine (UK) and Radboud University Nijmegen Medical Center (The Netherlands), as some of the tests will require additional special equipment that is not available at all sites.

- We will look for the type of infection using a microscope and by growing the organisms in the laboratory. We will test the organisms that grow to see which medicines work best to kill the infection, which is helpful in guiding the choice of treatment to be used.

- The swab samples from the ulcer will be used to test for infection using molecular diagnostic tests and to evaluate new tests that may be used to find the cause rapidly in the clinic.

- We will use study genetic material of the organism causing the infection to find out the exact type of infection and its ability to resist treatments. Samples from the ulcer may also be used to investigate your immune response to the infection, so that we can better understand what causes corneal scarring

- We will store a sample of the infection causing organism indefinitely for additional testing.

- The genetic material from the cells from your cheek will be sent to the UK or to KCMC Hospital in Tanzania. We would like to store it until a later time when a sufficiently large number of samples has been collected to conduct an analysis of this genetic material. The purpose would be to try to better understand how the human immune system fights the infection and why some people develop this eye problem and others do not. By doing these tests we hope that it will help us to develop approaches that will help to prevent or improve outcomes from this condition.

- As part of this consent we are asking that you give us permission to store this material to be able to test it at a later date as mentioned above. We do not know exactly for how long we shall store the genetic material before we have assembled a sufficiently large collection during the course of several planned studies to be able to proceed to the sample analysis, however, we anticipate a period of at least five years.

**What will happen to the results of the research study?**
The results of the study will be available after it finishes and will be included in peer reviewed medical and scientific journals and may be presented at medical meetings. Results will also be published on a publicly accessible trials database. The data will be anonymous and none of the patients involved in the trial will be identified in any report or publication. Should you wish to see the results, or the publication, please ask your study doctor.

**Who is funding the research?**
The research is being funded as part of a grant from the Wellcome Trust, UK.

**Who is organising the research?**
It is being organised through a research partnership between the London School of Hygiene and Tropical Medicine and Sagarmatha Choudhary Eye Hospital in Nepal, Mbarara University of Science and Technology in Uganda and Kilimanjaro Christian Medical Centre in Tanzania.

**What if relevant new information becomes available?**
It is not anticipated that new information will become available during the course of this short study. The information from the microbiology tests will be available to the doctors to help select the most appropriate treatment for you.

For peer review only

**What if something goes wrong?**
If you have a concern about any aspect of this study, you should ask to speak to the researchers who will do their best to answer your questions. If you remain unhappy and wish to complain formally, you can do this through the head of the hospital eye department or the named person on the following page. The London School of Hygiene and Tropical Medicine holds insurance policies which apply to this study. If you experience harm or injury as a result of taking part in this study, you may be eligible to claim compensation.

**Who has reviewed the study?**
Prospective research such as this is looked at by an independent group of people, called a Research Ethics Committee, to protect your interests. This study has been reviewed and approved by (1) the London School of Hygiene and Tropical Medicine Research Ethics Committee; (2) Nepal Health Research Council.

**What will happen if I don't want to carry on with the study?**
Your participation in this study is entirely voluntary. You may refuse to participate or may withdraw from this study at any time without penalty or loss of any rights or benefits to which you are otherwise entitled. The study doctor may also stop your participation in the study at any time for safety reasons. If you decide to withdraw from the study you should contact a member of the study team immediately. You do not have to give a reason when stopping, however for safety reasons, it is suggested that you tell the study doctor if you decide to stop because of an unwanted side effect. If you withdraw from the study, we will only use data collected before this decision, unless you request this to also be withdrawn. If you withdraw from the study, researchers, authorized persons from the Sponsor and the regulatory authorities will still require access to your medical notes to verify the data collected up to the date of your withdrawal.

**Contact Details**
Nepal Study Site: Dr Reena Yadav, Sagarmatha Choudhary Eye Hospital, Lahan. T: +977 9807799958

Study Coordinator: Dr Jeremy Hoffman: email: Jeremy.hoffman@lshtm.ac.uk
Chief Investigator: Prof. Matthew Burton: email: matthew.burton@lshtm.ac.uk

**You will be given a copy of the information sheet and a signed consent form to keep.**
**Thank you for considering taking the time to read this sheet.**

For peer review only

**Randomised controlled trial of topical chlorhexidine 0.2% versus topical natamycin 5% for fungal keratitis**

## Consent Form No 1: Baseline assessment and investigations

**Participant Name**__________________________ **Study ID Number:** ___________________

| | **Please initial box** |
|---|---|
| 1. I confirm that I have read and understand the participant information sheet dated ……….. (version ………) for the above study. I have had the opportunity to consider the information, ask questions and have had these answered fully. | |
| 2. I understand that my participation is voluntary and I am free to withdraw at any time, without giving any reason, without my medical care or legal rights being affected. | |
| 3. I understand that sections of my medical notes and data collected during the study may be looked at by responsible individuals from the London School of Hygiene & Tropical Medicine, from national regulatory authorities or from this hospital, where it is relevant to my taking part in this research. I give permission for these individuals to access my records. | |
| 4. I agree to take part in the above study. | |
| 5. I agree to the collection, laboratory tests and storage for future analysis of the samples from the surface my eye infection to understand the disease as described above. | |
| 6. I agree to the collection and storage for future analysis of the samples from the inside of my mouth to investigate how human genes affect this disease. | |
| 7. I agree to having a blood sample collected to measure sugar levels | |
| 8. I agree to being referred for a HIV pre-test counselling, testing and for the results to be shared with the research team. | |
| 9. I agree for the photographs of the front of my eye to be used in the publication or report released on the study, and for teaching purposes, including on the internet. | |
| 10. I understand that data about/from me/the participant may be shared via a public data repository or by sharing directly with other researchers, and that I will not be identifiable from this information. | |

For peer review only

| Name of Participant *(printed)* | Signature/Thumbprint | Date |
|---|---|---|
| Name of Person taking consent | Signature | Date |

The participant is unable to sign. As a witness, I confirm that all the information about the study was given and the participant consented to taking part.

| Name of Impartial Witness *(if required)* | Signature | Date |
|---|---|---|

*1 copy for participant; 1 copy for Principal Investigator; 1 copy to be kept with hospital notes*

**Study Title: Randomised controlled trial of topical chlorhexidine 0.2% versus topical natamycin 5% for fungal keratitis in Nepal**

*Participant Information Sheet and Consent Form – No 2:*
*For enrolment into the clinical trial*

**Introduction**
Thank you for earlier participating in the first stage of the study. You are now being invited to take part in the clinical treatment trial part of this medical research study. Before you decide it is important for you to understand why the research is being done and what it will involve. Please take time to read or listen to the following information carefully and to talk to others about the study, if you wish. Ask us if there is anything that is not clear or if you would like more information. Do not sign the consent form unless you are satisfied with the answers to your questions and decide that you want to be part of this study. Take time to decide whether or not you wish to take part.

**Why have I been invited?**
You have been invited to take part because you have a corneal infection which we have found out from tests is caused by fungus.

**What is this study about?**
Infection of the clear part of the front of the eye (the cornea) is a corneal ulcer. It is an important cause of blindness in Nepal. A scratch in the cornea allows infection to enter and an ulcer to begin. These infections can be very serious with some people losing the sight in the affected eye.

Different types of infectious organisms can cause corneal ulcers. These include bacteria and fungi. In tropical regions about half of all corneal ulcers are caused by fungi. Bacteria and fungi need to be treated with different types of eye drop medicines.

Treatments for fungal eye infections are frequently not very effective, in addition access to these treatments in many countries is very limited and can be expensive. In some countries they are simply not available. Currently the most commonly used treatment for fungal corneal ulcers is an eye drop called Natamycin. There is a need for additional, alternative, affordable and more easily available eye drop treatments for fungal infections.

There is an antiseptic solution called Chlorhexidine. This is very effective at killing bacteria, fungi and other types of infectious organisms. It is used in medical care worldwide in several different ways. For example, it is used to clean skin before surgical operations, in antiseptic creams for skin cuts and as a mouth wash to prevent and treat mouth infections. It has been used in eye care for more than thirty years as an eye-drop preservative, for sterilizing contact lenses, for pre-operative topical antiseptic and for treating *Acanthamoeba* and fungal corneal infections.

About twenty years ago chlorhexidine eye drops were tested in two small clinical trials conducted in India and Bangladesh for the treatment of fungal corneal infections. The results of these studies suggested that chlorhexidine was as good as and possibly better than natamycin at controlling the infection. Neither eye drop had any serious side effect. However, the studies were not large enough to be certain.

Chlorhexidine is currently used in several countries for the treatment of fungal corneal infections when natamycin or alternative treatment is not working or is not available. We would like to conduct a large clinical to find out whether chlorhexidine 0.2% eye drops are as effective as or more effective than natamycin 5% eye drops for treating fungal corneal infections

**Do I have to take part?**
No. Your involvement is entirely voluntary. If you agree to take part, we will then ask you to sign a consent form. If you decide to join and change your mind, you are free to withdraw at any time without giving a reason. This will not affect the standard of care you receive. If you decide not to participate in the study, then you will be offered the standard treatment for fungal keratitis using natamycin.

**What will happen to me if I take part?**
If you agree to be part of this study, the following will happen:

***1) Baseline Assessment:***
As part of the initial assessment that has already taken place we have carefully examined yours eyes, taken photographs, performed a scan for infection and collected samples. The tests have found that you have a fungal infection in your cornea. The risks to an unborn or breast-fed baby from antifungal eye drops use are unknown. Therefore, pregnant and breastfeeding women are excluded from participating in this study. Pregnancy testing will be offered to potential female participants to confirm pregnancy status.

For peer review only

***2) Randomisation:***
We will randomly allocate you to one of the two treatment option: either chlorhexidine eye drops or natamycin eye drops. Sometimes we don't know which way of treating patients is best. To find out, we need to make comparisons between the different treatments. We put people into groups and give each group one of the two alternative treatments; the results are compared after some time to see if one is better. To try to make sure the groups are the same to start with, each patient is put into a group by chance (randomly). You have an equal chance of being put into the chlorhexidine or natamycin treatment group. Neither you nor the people examining your eyes will be told which treatment group you are in. It is important that neither you nor we know which of the two you are given. This information would be in our files, but we would not look at these files until after the research is finished. This is the best way we have for testing without being influenced by what we think might happen. We would then compare which of the two treatments has the best results.

***3) Treatment:***
Once you are allocated to one of the treatment groups, you will receive clear instruction on how to take the eye drops. For the first week we will ask you to take one drop every hour. For the second and the third weeks the frequency of the eye drops will be reduced to every other hour (2-hourly). After that the frequency and duration of treatment will depend on the severity of the infection and how it is responding. You will be given clear guidance on this by the eye doctor who is looking after you.

***4) Follow-up Assessment:***
Initially, most people with corneal infections stay in hospital for several days so that the clinical team can monitor the response of the infection to the treatment. For the purposes of the study, we would like to review the response to treatment and document the clinical findings at the following times after you start treatment: two days, 1 week, 2 weeks, 3 weeks, 2 months and 3 months.

On each occasion we will ask you a few questions about your eye and the treatment. We will measure your eye sight. We will examine the eye with a microscope and photograph it with a camera.

At 1 week, 2 weeks and 3 weeks we will repeat the *in vivo* confocal microscopy test that was done at your first assessment. This is done to see how the infection is responding to treatment. This involves putting anaesthetic drops on the eye so that you do not feel any discomfort. A soft plastic device then gently touches the surface of the eye so that we can take special photographs of the front of your eye ("scan")

At 1 week if you still have an open ulcer on the cornea we will repeat the sample collection to test for the ongoing presence of the infection. This involves first putting anaesthetic eye drops on the surface of the eye. Then gently scraping the surface of the corneal ulcer and testing for the presence of fungus and bacteria in the microbiology laboratory.

Sometimes fungal infections do not respond to the treatment. In such cases it may be necessary to alter the treatment or perform an operation. The eye doctors who will be looking after you will monitor your progress closely and advise you about further treatment might be needed.

At the three month review you will be asked to complete three short questionnaires to assess your quality of life and vision function. These are the same standardised questions used in the baseline assessment to try to understand the impact that different medical problems can have on people's lives.

You may be withdrawn from the study without your consent if the researchers believe it is in your best interest or if you fail to follow study procedures.

**What are the side effects or risks of taking part?**

1. **Random Allocation and Treatment Failure**: You will be randomly allocated to a treatment. The treatment you are allocated to may prove to be less effective or to have more side effects than the other study treatment or other available treatments.

   **<u>It is important to recognise that corneal infection is a serious, sight threatening condition.</u>** Many patients, whatever the treatment used, have reduced vision in the affected eye after it has resolved. In some people the affected eye will become blind. Sometimes the infection, despite lots of treatment, can progress to cause a hole to develop in the cornea (Perforation) and sometimes it is so severe it is necessary to perform an operation to remove the eye content.

2. **Local Irritation:** As with most eye drops, there is the risk of local irritation or stinging from either chlorhexidine or natamycin. This usually only lasts for a short time.

3. **Allergic Response**: Very rarely, either chlorhexidine or natamycin eye drops can provoke a local allergic reaction on the surface of the eye or the eyelids.

For peer review only

4. **Pregnancy and Breast Feeding:** The risks to an unborn or breast-fed baby from antifungal eye drops use are unknown. Therefore, pregnant and breastfeeding women are excluded from participating in this study.

5. **Natamycin 5% eye drops**: Natamycin is an approved antifungal medication that is currently being used for the treatment of fungal corneal ulcer. It is on the World Health Organisation Essential Medicines List for the treatment of fungal corneal infections. There are no known serious side effects with this medication. It may cause mild irritation and very rarely a local allergic response.

6. **Chlorhexidine 0.2% eye drops:** Chlorhexidine eye drops are used on the surface of the eye as an antiseptic before procedures and also in the treatment of fungal and other eye infections. It has not been associated we any serious side effects. It may cause mild irritation and very rarely a local allergic response. This concentration of chlorhexidine is approved to be used in much larger volumes as a mouth wash. It is considered to be safe and is not associated with any systemic side effects.

7. **Procedures:** including examinations, confocal microscopy, corneal sample collection and checking for the best glasses or contact lenses carry the same very small risk whether they are performed as part of this study or of usual care outside the study. To minimise discomfort, topical anaesthetic will be given before examinations and sample collection.

8. **Unknown Risks:** The treatments in this study may have rare side effects that are currently not known. If during the course of the study new information becomes available, the researchers will share this with.

**What are the possible benefits of taking part?**

- The study will involve tests for the type of infection. This helps the doctor looking after you to choose the best type of treatment for your eyes
- The costs for your clinical assessment, tests, treatment and transport will be paid for by the study.
- By participating in this study, you will be helping to answer the question about whether or not chlorhexidine is a suitable alternative treatment for fungal corneal infections.

**What will happen to the clinical records, photographs and test results?**

Your records will remain strictly confidential at all times. The information will be held in a secure office at your treating hospital. Only the people organizing or supervising the trial and regulatory authority auditors will have access to it. These include officials delegated by the Sponsor (London School of Hygiene and Tropical Medicine), the local National Ethics Committee, The local National Drug Regulatory Authority and trial Data Safety Monitoring Body (DSMB).

A study number rather than your name will be used on study records or the database wherever possible. Your name and other facts that might identify you will not appear when we present this study or publish its results.

Your name will not be passed to anyone else outside the research team, unless we have your direct instruction to do so, for example to make a medical referral.

Images of corneal infection may be used for educational and teaching purposes, including in publications. All personal identifying information will be removed before sharing images.

**What tests will we do on the sample?**

The samples collected from the surface of your eye will be tested in several different ways to determine what is causing the infection. This work will be done in the hospital microbiology laboratory, where you are being treated. A portion of the infection sample will be transferred for additional special tests at KCMC Hospital Biotechnology (Tanzania), the London School of Hygiene and Tropical Medicine (UK) and Radboud University Nijmegen Medical Center (The Netherlands).

We will look for the type of infection using a microscope and by growing the organisms in the laboratory. We will test the organisms that grow to see which medicines work best to kill the infection, which is helpful in guiding the choice of treatment to be used. The swab samples from the ulcer will be used to test for infection using molecular diagnostic tests and to evaluate new tests that may be used to find the cause rapidly in the clinic. We will use the genetic material of the organism causing the infection to sequence its genetic code, which helps us to find out the exact type of infection and its ability to resist treatments. We will store a sample of the infection causing organism indefinitely for additional testing.

**What will happen to the results of the research study?**

The results of the study will be available after it finishes and will be included in peer reviewed medical and scientific journals and may be presented at medical meetings. Results will also be published on a publicly accessible trials database. The data will be anonymous and none of the patients involved in the trial will be identified in any report or publication. Should you wish to see the results, or the publication, please ask your study doctor.

**Who is funding the research?**

For peer review only

The research is being funded as part of a grant from the Wellcome Trust, UK.

**Who is organising the research?**
It is being organised through a research partnership between the London School of Hygiene and Tropical Medicine and Sagarmatha Choudhary Eye Hospital in Nepal, Mbarara University of Science and Technology in Uganda and Kilimanjaro Christian Medical Centre in Tanzania.

**What if relevant new information becomes available?**
It is not anticipated that new information will become available during the course of this study. However, if it does, this will be shared with you by the researchers in case this affects whether you wish to continue in the study.

**What if something goes wrong?**
If you have a concern about any aspect of this study, you should ask to speak to the researchers who will do their best to answer your questions. If you remain unhappy and wish to complain formally, you can do this through the head of the hospital eye department or the named person on the following page. The London School of Hygiene and Tropical Medicine holds insurance policies which apply to this study. If you experience harm or injury as a result of taking part in this study, you may be eligible to claim compensation.

**Who has reviewed the study?**
Prospective research such as this is looked at by an independent group of people, called a Research Ethics Committee, to protect your interests. This study has been reviewed and approved by (1) the London School of Hygiene and Tropical Medicine Research Ethics Committee; (2) Nepal Health Research Council.

What will happen if I don't want to carry on with the study?
Your participation in this study is entirely voluntary. You may refuse to participate or may withdraw from this study at any time without penalty or loss of any rights or benefits to which you are otherwise entitled. The study doctor may also stop your participation in the study at any time for safety reasons. If you decide to withdraw from the study you should contact a member of the study team immediately. You do not have to give a reason when stopping, however for safety reasons, it is suggested that you tell the study doctor if you decide to stop because of an unwanted side effect. If you withdraw from the study, we will only use data collected before this decision, unless you request this to also be withdrawn. If you withdraw from the study, researchers, authorized persons from the Sponsor and the regulatory authorities will still require access to your medical notes to verify the data collected up to the date of your withdrawal.

**Contact Details**
Nepal Study Site: Dr Reena Yadav, Sagarmatha Choudhary Eye Hospital, Lahan. T: +977 9807799958

Study Coordinator: Dr Jeremy Hoffman: email: Jeremy.hoffman@lshtm.ac.uk
Chief Investigator: Prof. Matthew Burton: email: matthew.burton@lshtm.ac.uk

**You will be given a copy of the information sheet and a signed consent form to keep.**
**Thank you for considering taking the time to read this sheet.**

For peer review only

**Randomised controlled trial of topical chlorhexidine 0.2% versus topical natamycin 5% for fungal keratitis**

**Consent Form No 2: Enrolment into the clinical trial**

**Participant Name**___________________________ **Study ID Number:** ____________________

| | **Please initial box** |
|---|---|
| 1. I confirm that I have read and understand the participant information sheet dated .......... (version .........) for the above study. I have had the opportunity to consider the information, ask questions and have had these answered fully. | |
| 2. I understand that my participation is voluntary and I am free to withdraw at any time, without giving any reason, without my medical care or legal rights being affected. | |
| 3. I understand that sections of my medical notes and data collected during the study may be looked at by responsible individuals from the London School of Hygiene & Tropical Medicine, from national regulatory authorities or from this hospital, where it is relevant to my taking part in this research. I give permission for these individuals to access my records. | |
| 4. I agree to take part in this clinical treatment trial. | |
| 5. I agree to the collection, laboratory tests and storage for future analysis of the samples from the surface my eye infection to understand the disease as described above. | |
| 6. I agree for the photographs of the front of my eye to be used in the publication or report released on the study, and for teaching purposes, including on the internet. | |
| 7. I understand that data about/from me/the participant may be shared via a public data repository or by sharing directly with other researchers, and that I will not be identifiable from this information. | |

Name of Participant *(printed)* Signature/Thumbprint Date

Name of Person taking consent Signature Date

The participant is unable to sign. As a witness, I confirm that all the information about the study was given and the participant consented to taking part.

Name of Impartial Witness *(if required)* Signature Date

*1 copy for participant; 1 copy for Principal Investigator; 1 copy to be kept with hospital notes*

For peer review only

# Appendix 2: Monitoring for Harm

## Definitions

**Adverse Event (AE):** any untoward medical occurrence in a patient or clinical trial subject administered a medicinal product and which does not necessarily have a causal relationship with this treatment. *An AE can therefore be any unfavourable and unintended sign (including an abnormal laboratory finding), symptom, or disease temporally associated with the use of an investigational medicinal product (IMP), whether or not considered related to the IMP.*

**Adverse Reaction (AR):** all untoward and unintended responses to an IMP related to any dose administered. *All AEs judged by either the reporting investigator or the sponsor as having reasonable causal relationship to a medicinal product qualify as adverse reactions. The expression reasonable causal relationship means to convey in general that there is evidence or argument to suggest a causal relationship.*

**Unexpected Adverse Reaction:** an AR, the nature or severity of which is not consistent with the applicable product information (e.g. investigator's brochure for an unapproved investigational product or summary of product characteristics (SmPC) for an authorised product*). When the outcome of the adverse reaction is not consistent with the applicable product information this adverse reaction should be considered as unexpected. Side effects documented in the SmPC which occur in a more severe form than anticipated are also considered to be unexpected.*

**Serious Adverse Event (SAE)** or **Serious Adverse Reaction:** any untoward medical occurrence or effect that at any dose

- **Results in death**
- **Is life-threatening** – *refers to an event in which the subject was at risk of death at the time of the event; it does not refer to an event which hypothetically might have caused death if it were more severe*
- **Requires hospitalisation, or prolongation of existing inpatients' hospitalisation**
- **Results in persistent or significant disability or incapacity**
- **Is a congenital anomaly or birth defect**

Medical judgement should be exercised in deciding whether an AE/AR is serious in other situations. Important AE/ARs that are not immediately life-threatening or do not result in death or hospitalisation but may jeopardise the subject or may require intervention to prevent one of the other outcomes listed in the definition above, should also be considered serious.

**Suspected Unexpected Serious Adverse Reaction (SUSAR):** any suspected adverse reaction related to an IMP that is both unexpected and serious.

## Causality

Most adverse events and adverse drug reactions that occur in this study, whether they are serious or not, will be expected treatment-related toxicities due to the drugs used in this study. The assignment of the causality should be made by the investigator responsible for the care of the participant using the definitions in the table below.

| Relationship | Description |
|---|---|
| **Unrelated** | There is no evidence of any causal relationship |
| **Unlikely** | There is little evidence to suggest there is a causal relationship (e.g. the event did not occur within a reasonable time after administration of the trial medication). There is another reasonable explanation for the event (e.g. the participant's clinical condition, other concomitant treatment). |
| **Possible** | There is some evidence to suggest a causal relationship (e.g. because the event occurs within a reasonable time after administration of the trial medication). However, the influence of other factors may have contributed to the event (e.g. the participant's clinical condition, other concomitant treatments). |
| **Probable** | There is evidence to suggest a causal relationship and the influence of other factors is unlikely. |

For peer review only

| | |
|---|---|
| **Definitely** | There is clear evidence to suggest a causal relationship and other possible contributing factors can be ruled out. |
| **Not assessable** | There is insufficient or incomplete evidence to make a clinical judgement of the causal relationship. |

If any doubt about the causality exists the local investigator should inform the study coordination centre who will notify the Chief Investigators. The pharmaceutical companies and/or other clinicians may be asked to advise in some cases.

In the case of discrepant views on causality between the investigator and others, all parties will discuss the case. In the event that no agreement is made, the MHRA will be informed of both points of view.

## Reporting Procedures

All adverse events should be reported. Depending on the nature of the event the reporting procedures below should be followed. Any questions concerning adverse event reporting should be directed to the study coordination centre in the first instance. A flowchart is given below (Figure 5) to aid in the reporting procedures and the reporting form is shown in (Data Collection Appendix).

***Figure 5: Adverse event reporting flow-chart***

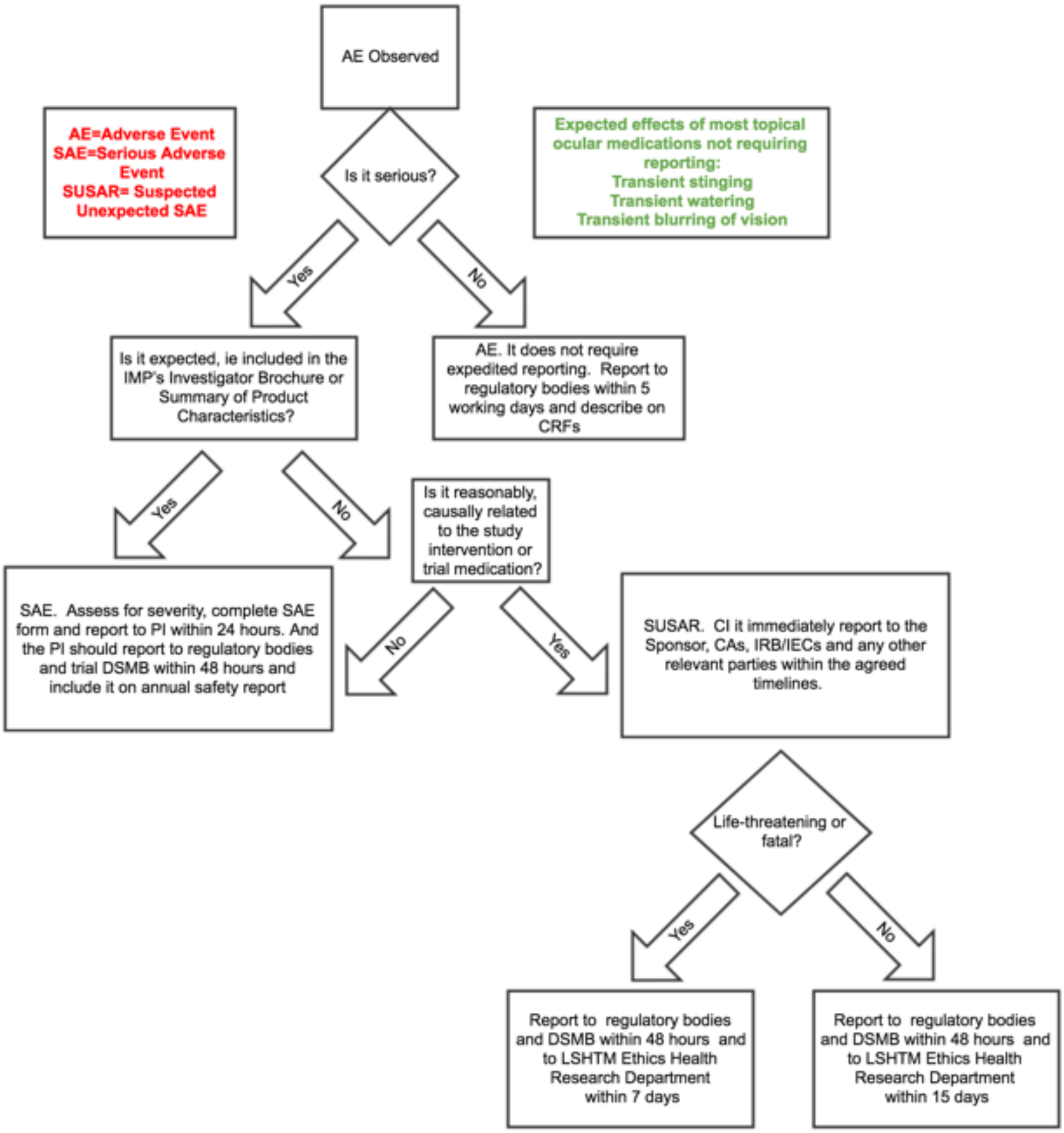

**Non-serious Adverse Reactions (ARs)/Adverse Events (AEs)**
All such events, whether expected or not, should be recorded in the toxicity section of the relevant case report form and sent to the study coordination centre, regulatory bodies and the trial DSMB within 5 working days from the day of occurrence.

**Serious Adverse Reactions (SARs)/Serious Adverse Events (SAEs)**
Fatal or life threatening Serious Adverse Events (SAEs), Serious Adverse Reactions (SARs) and Suspected Unexpected Serious Adverse Reactions (SUSARs) should be reported to the PI on the day that the local site is aware of the event. The SAE form asks for nature of event, date of onset, severity, corrective therapies given, outcome and causality (i.e. unrelated, unlikely, possible, probably, definitely). All SAEs or SARs whether expected, suspected or unexpected should be reported to regulatory bodies and the trial DSMB within 48 hours of occurrence. The responsible investigator should sign the causality of the event. Additional information should be sent within 5 days if the reaction has not resolved at the time of reporting. All investigators will be informed of all SARs occurring throughout the study.

**SAEs**
A SAE form should be completed and faxed to the study coordination centre for all SAEs within 24 hours. However, relapse and death due to a known pre-existing condition and hospitalisations for elective treatment of a pre-existing condition do not need reporting as SAEs.

**SUSARs**
In the case of serious, unexpected and related adverse events, the staff at the site should:

Complete the SAE case report form & send it immediately (within 24 hours, preferably by fax), signed and dated to the study coordination centre together with relevant treatment forms and anonymised copies of all relevant investigations.

**Or**

Contact the study coordination centre by phone and then send the completed SAE form to the study coordination centre within the following 24 hours as above.

The study coordination centre will notify all SUSARs occurring during the study within 48 hours to the Nepal Department of Drug Administration, and the trial DSMB. SUSARs will be reported to LSHTM Ethics Committee according to the following timelines; fatal and life-threatening within 7 days of notification and non-life threatening within 15 days. All investigators will be informed of all SUSARs occurring throughout the study.

Local investigators should report any SUSARs and /or SAEs as required by their Local Research Ethics Committee and/or Research & Development Office.

**Adverse Event Reporting for this Trial**
**Non-serious Ocular: narrative form not required**

- ☐ Local allergic reaction (conjunctiva, lid)
- ☐ Intraocular pressure ≥35 mmHg for 1 week despite therapy
- ☐ Progressive corneal thinning, ≤50% of enrolment thickness
- ☐ Other (specify)

**Serious Ocular: (1) report within 24hrs (2) Serious Adverse Event (SAE) Form required**

- ☐ Endophthalmitis
- ☐ Other serious ocular event thought to be related to the study drug, requiring non-elective hospitalization, loss of function, or systemic treatment (specify)

**Serious Non-ocular: (1) report within 24hrs (2) Serious Adverse Event (SAE) Form required**

- ☐ Death
- ☐ Non-elective surgery, hospitalization, or loss of function
- ☐ Myocardial infarction or stroke
- ☐ Systemic allergic reaction

For peer review only

## Appendix 3: Biological Specimens

The project will equip and staff an ocular microbiology laboratory at the hospital. The microbiology analysis will involve the following investigations:

1) **Microscopy** will be performed of the slides using Gram stain, KOH and calcofluor white.

2) **Culture** will be performed using a variety of media inoculated with corneal scrape samples in the clinic: blood agar, chocolate agar, brain-heart infusion broth (incubated for 2-7 days at 37°C) and Sabouraud agar (incubated for 7 days at 30°C). Culture isolates will be identified using standard microbiological techniques. Fungal and bacterial isolates will be stored frozen for long-term reference and subsequent additional analysis.

3) **Antibiotic susceptibility testing** of bacteria culture isolates will be performed using standard disc diffusion techniques in each of the laboratories.

4) **Antifungal susceptibility testing** of fungal isolates will be performed at KCMC, Tanzania . Isolates from Uganda and Nepal will be periodically transferred to KCMC, under a Material Transfer Agreement. They will be re-cultured and tested using a dilution series method for four drugs: Natamycin, Chlorhexidine, Voriconazole and Amphotericin-B. The technique has been developed at Radboud University, The Netherlands.

**PCR for microorganisms**

DNA from the dry swab will be extracted using the QIAamp mini kit (Qiagen) with bead-beating. Samples will be tested for the presence of bacterial (16rRNA), fungal (ITS1) and specific viral (HSV1&2) DNA by quantitative PCR. This PCR work will be performed at KCMC, Tanzania. Samples from Uganda and Nepal will be periodically transferred to KCMC for testing, under a Material Transfer Agreement. The performance of PCR as a diagnostic tool will be evaluated against a composite reference standard for positive cases. Cases will be considered positive if the pathogen is detected by either smear microscopy, IVCM or culture.

**Point of care tests for fungal infection**

Point-of-care tests for fungal keratitis would be very helpful. Molecular tests are generally more sensitive than antigen-based assays. Isothermal nucleic amplification techniques offer the sensitivity of PCR amplification without the need for specialized equipment. A result may be obtained within 30 minutes. Promising molecular approaches include isothermal amplification (Loop-mediated isothermal amplification (LAMP), Recombinase polymerase amplification (RPA)), associated with lateral flow assays for amplicon detection. Several assays have been developed that detect fungi, however, these have not been evaluated in fungal keratitis. The second ulcer swab will be used to test and develop isothermal molecular assays in comparison with the standard microbiological approaches described above. Results will be evaluated against a composite reference standard for fungal diagnosis as described above.

For peer review only

**Fungal DNA sequencing**

It is not always possible to determine the species of a fungus from classical microbiological techniques. It is now common to use fungal DNA sequencing for species-level diagnosis for some genera, including *Fusarium spp.*, which is the commonest fungal genus reported for MK in the study settings. We will use sequencing of the fungal ITS and TEF1α regions for this.[1] DNA will be extracted from the cultured fungal isolate when available. If there no fungus is cultured but the PCR is positive, then this PCR product will be used for sequencing. The DNA will be extracted from the isolates in KCMC, Tanzania. The extracted DNA will be sent under a Material Transfer Agreement to the Center of Expertise in Mycology Radboudumc/CWZ, Radboud University Nijmegen Medical Center and the CBS Fungal Biodiversity Centre, Utrecht, the Netherlands for the specialised sequencing and identification work.

**Corneal gene expression**

Three prime RNAseq (3'RNAseq) generates one read per transcript and enables strand-specific, highly multiplexed sequencing of poor concentration or quality samples (as low as 100pg total RNA input) for gene expression analysis. We have previously used this method for gene expression profiling of human conjunctival swabs from a trachoma-endemic area. RNA will be extracted from corneal swabs or scrapes using Norgen RNA purification kits. 3'RNAseq (Lexogen) will be performed on normalised RNA extracts from 500 Tanzanian and Ugandan participants with confirmed fungal keratitis. Pooled libraries will be sequenced on a HiSeq (Illumina). Transcriptomic profiles will be analysed for differences between controls and mild, moderate and severe FK cases using DEseq2, and between participants with good versus poor visual outcomes. A small number of healthy corneal tissue controls will be derived from the residual ring of tissue remaining after corneal graft surgery (ethics application for control tissue to be submitted separately).

**Human Genetics**

DNA will be extracted from buccal brush samples using QIAmp DNA mini kits (Qiagen). Samples from 500 confirmed fungal keratitis participants from Nepal and 500 matched controls will be run on immune-focussed Infinium ImmunoArray-24 BeadChips (Illumina) covering >240,000 genetic polymorphisms (including copy number variants, germline variants, insertions-deletions, single nucleotide polymorphisms (SNPs) and structural variants) at UCL genomics centre. In addition to SNP testing, two-field resolution Human Leukocyte Antigen (HLA) allele typing will be performed in all 1000 samples. Allele level HLA typing cannot currently be determined from SNP data but the HLA genes are primary immune response genes that have a role in many diseases and warrant independent study. Data will be analysed to determine sources of immunogenetic variation associated with fungal keratitis. These data will be linked to gene expression data from the same individuals to determine functional effects of genetic variation associated with fungal keratitis.

For peer review only

**HIV testing**

As outlined above, patients with microbial keratitis will be offered counselling and testing for HIV infection. This will be done through the established services in each of the hospitals. Appropriate onward referrals will be made if a test is found to be positive.

**Blood glucose testing**

Patients will have capillary blood glucose (CBG) testing done at presentation. Patients with an elevated CBG (>6.1mmol) will be referred to the hospital physicians for assessment and formal diagnosis of impaired glucose tolerance or diabetes mellitus. This level is considered a suitable cut-off to detect individuals with diabetes and has been validated in a south-Asian population.[2]

**References:**


1 Schoch CL, Seifert KA, Huhndorf S, *et al.* Nuclear ribosomal internal transcribed spacer (ITS) region as a universal DNA barcode marker for Fungi. *Proc Natl Acad Sci USA* 2012;**109**:6241–6. doi:10.1073/pnas.1117018109

2 Somannavar S, Ganesan A, Deepa M, *et al.* Random capillary blood glucose cut points for diabetes and pre-diabetes derived from community-based opportunistic screening in India. *Diabetes Care* 2009;**32**:641–3. doi:10.2337/dc08-0403


For peer review only

# Appendix 4: SPIRIT Checklist for Topical chlorhexidine 0.2% versus topical natamycin 5% for fungal keratitis in Nepal randomised clinical trial

| Section / Item | Item No. | Description | Location Reference |
|---|---|---|---|
| **Administrative information** | | | |
| Title | 1 | Descriptive title identifying the study design, population, interventions, and, if applicable, trial acronym | Page 1 of study protocol |
| Trial registration | 2a | Trial identifier and registry name. If not yet registered, name of intended registry | ISRCTN14332621; pre-results |
| | 2b | All items from the World Health Organization Trial Registration Data Set | ISRCTN14332621 Study Registry |
| Protocol Version | 3 | Date and version identifier | Every page of study protocol |
| Funding | 4 | Sources and types of financial, material, and other support 5a Names, affiliations, and roles of protocol contributors | Section 5.3 of study protocol, page 25<br>Page 17 of Manuscript |
| Roles and Responsibilities | 5a | Names, affiliations, and roles of protocol contributors | Page 1 of study protocol<br>Page 1 of Manuscript |
| | 5b | Name and contact information for the trial sponsor | Page 1 of study protocol<br>Page 1 of Manuscript |
| | 5c | Role of study sponsor and funders, if any, in study design; collection, management, analysis, and interpretation of data; writing of the report; and the decision to submit the report for publication, including whether they will have ultimate authority over any of these activities | Page 1 of study protocol and Section 5.2 of study protocol, page 25.<br><br>Page 17 of Manuscript |
| | 5d | Composition, roles, and responsibilities of the coordinating centre, steering committee, endpoint adjudication committee, data management team, and other individuals or groups overseeing the trial, if applicable (see Item 21a for data monitoring committee) | Section 5.1, page 24 of study protocol<br><br>Section 6, page 25 of study protocol<br><br>Page 11 of Manuscript |
| **Introduction** | | | |
| Background and rationale | 6a | Description of research question and justification for undertaking the trial, including summary of relevant studies (published and unpublished) examining benefits and harms for each intervention | Section 1, pages 6-9 of study protocol<br><br>Pages 4-5 of Manuscript |
| | 6b | Explanation for choice of comparators | Sections 1.3 – 1.4, pages 7-9 of study protocol<br>Pages 4-5 of Manuscript |

For peer review only

| | | | |
|---|---|---|---|
| Objectives | 7 | Specific objectives or hypotheses | Section 3, page 11 of study protocol<br><br>Page 5 of Manuscript |
| Trial design | 8 | Description of trial design including type of trial (eg, parallel group, crossover, factorial, single group), allocation ratio, and framework (eg, superiority, equivalence, noninferiority, exploratory) | Section 4, pages 12-24 of study protocol<br><br>Page 6 of Manuscript |
| **Methods: Participants, interventions, and outcomes** | | | |
| Study setting | 9 | Description of study settings (eg, community clinic, academic hospital) and list of countries where data will be collected. Reference to where list of study sites can be obtained | Section 4.3, page 13 of study protocol<br><br>Page 6 of Manuscript |
| Eligibility criteria | 10 | Inclusion and exclusion criteria for participants. If applicable, eligibility criteria for study centres and individuals who will perform the interventions (eg, surgeons, psychotherapists) | Section 4.4, page 13 of study protocol<br><br>Page 7 of Manuscript and Table 1 of Manuscript |
| Interventions | 11a | Interventions for each group with sufficient detail to allow replication, including how and when they will be administered | Section 4.7, page 17 of study protocol<br><br>Pages 8-9 of Manuscript |
| | 11b | Criteria for discontinuing or modifying allocated interventions for a given trial participant (eg, drug dose change in response to harms, participant request, or improving/worsening disease) | Page 11 of Manuscript |
| | 11c | Strategies to improve adherence to intervention protocols, and any procedures for monitoring adherence (eg, drug tablet return, laboratory tests) | Section 4.9, page 19 of study protocol<br><br>Pages 10-11 of Manuscript |
| | 11d | Relevant concomitant care and interventions that are permitted or prohibited during the trial | Section 4.7, pages 17-18 of study protocol<br>Pages 9-10 of Manuscript |
| Outcomes | 12 | Primary, secondary, and other outcomes, including the specific measurement variable (eg, systolic blood pressure), analysis metric (eg, change from baseline, final value, time to event), method of aggregation (eg, median, proportion), and time point for each outcome. Explanation of the clinical relevance of chosen efficacy and harm outcomes is strongly recommended | Section 4.11, page 22 of study protocol<br><br>Pages 10-11 of Manuscript |

For peer review only

| | | | |
|---|---|---|---|
| Participant timeline | 13 | Time schedule of enrolment, interventions (including any run-ins and washouts), assessments, and visits for participants. A schematic diagram is highly recommended (see Figure) | Table 3, page 19 of study protocol<br><br>Table 4 of Manuscript |
| Sample size | 14 | Estimated number of participants needed to achieve study objectives and how it was determined, including clinical and statistical assumptions supporting any sample size calculations | Section 4.12, page 22 of study protocol<br><br>Pages 12 -13 of Manuscript |
| Recruitment | 15 | Strategies for achieving adequate participant enrolment to reach target sample size | Section 4.5, page 14 of study protocol<br><br>Page 7 of Manuscript |
| **Methods: Assignment of interventions (for controlled trials)** | | | |
| Allocation: sequence generation | 16a | Method of generating the allocation sequence (eg, computer- generated random numbers), and list of any factors for stratification. To reduce predictability of a random sequence, details of any planned restriction (eg, blocking) should be provided in a separate document that is unavailable to those who enrol participants or assign interventions | Section 4.8, pages 18-19 of study protocol<br><br>Pages 7-8 of Manuscript |
| Allocation: concealment mechanism | 16b | Mechanism of implementing the allocation sequence (eg, central telephone; sequentially numbered, opaque, sealed envelopes), describing any steps to conceal the sequence until interventions are assigned | |
| Allocation: Implementation | 16c | Who will generate the allocation sequence, who will enrol participants, and who will assign participants to interventions | |
| Blinding (masking) | 17a | Who will be blinded after assignment to interventions (eg, trial participants, care providers, outcome assessors, data analysts), and how | Section 4.8, pages 18-19 of study protocol<br><br>Page 8 of Manuscript |
| | 17b | If blinded, circumstances under which unblinding is permissible, and procedure for revealing a participant's allocated intervention during the trial | Page 8 of Manuscript |
| **Methods: Data collection, management, and analysis** | | | |
| | 18a | Plans for assessment and collection of outcome, baseline, and other trial data, including any | Section 4.14, page 23 of study protocol |

For peer review only

| | | | |
|---|---|---|---|
| Data collection methods | | related processes to promote data quality (eg, duplicate measurements, training of assessors) and a description of study instruments (eg, questionnaires, laboratory tests) along with their reliability and validity, if known. Reference to where data collection forms can be found, if not in the protocol | Page 12 of Manuscript |
| | 18b | Plans to promote participant retention and complete follow-up, including list of any outcome data to be collected for participants who discontinue or deviate from intervention protocols | Page 12 of Manuscript |
| Data management | 19 | Plans for data entry, coding, security, and storage, including any related processes to promote data quality (eg, double data entry; range checks for data values). Reference to where details of data management procedures can be found, if not in the protocol | Section 4.14, page 23 of study protocol<br><br>Page 12 of Manuscript |
| Statistical methods | 20a | Statistical methods for analysing primary and secondary outcomes. Reference to where other details of the statistical analysis plan can be found, if not in the protocol | Section 4.13, pages 22-23 of study protocol<br><br>Pages 12-15 of Manuscript |
| | 20b | Methods for any additional analyses (eg, subgroup and adjusted analyses) | |
| | 20c | Definition of analysis population relating to protocol non-adherence (eg, as randomised analysis), and any statistical methods to handle missing data (eg, multiple imputation) | |
| **Methods: Monitoring** | | | |
| Data Monitoring | 21a | Composition of data monitoring committee (DMC); summary of its role and reporting structure; statement of whether it is independent from the sponsor and competing interests; and reference to where further details about its charter can be found, if not in the protocol. Alternatively, an explanation of why a DMC is not needed | Section 6, page 25 of study protocol<br><br>Page 12 of Manuscript |
| | 21b | Description of any interim analyses and stopping guidelines, including who will have access to | Section 4.13, page 23 of study protocol |

For peer review only

| | | | |
|---|---|---|---|
| | | these interim results and make the final decision to terminate the trial | Page 15 of Manuscript |
| Harms | 22 | Plans for collecting, assessing, reporting, and managing solicited and spontaneously reported adverse events and other unintended effects of trial interventions or trial conduct | Section 7, pages 26-28 of study protocol<br><br>Page 12 of Manuscript<br><br>Annex 2 |
| Auditing | 23 | Frequency and procedures for auditing trial conduct, if any, and whether the process will be independent from investigators and the sponsor | Section 6, page 25 of study protocol<br><br>Page 12 of Manuscript |
| **Ethics and Dissemination** | | | |
| Research ethics approval | 24 | Plans for seeking research ethics committee/institutional review board (REC/IRB) approval | Section 4.15, page 24 of study protocol<br>Page 15 of Manuscript |
| Protocol amendments | 25 | Plans for communicating important protocol modifications (eg, changes to eligibility criteria, outcomes, analyses) to relevant parties (eg, investigators, REC/IRBs, trial participants, trial registries, journals, regulators) | Section 4.15, page 24 of study protocol<br>Page 15 of Manuscript |
| Consent or assent | 26a | Who will obtain informed consent or assent from potential trial participants or authorised surrogates, and how (see Item 32) | Section 4.5, page 14 of study protocol<br><br>Page 7 of Manuscript |
| | 26b | Additional consent provisions for collection and use of participant data and biological specimens in ancillary studies, if applicable | Appendix 1: Consent Forms |
| Confidentiality | 27 | How personal information about potential and enrolled participants will be collected, shared, and maintained in order to protect confidentiality before, during, and after the trial | Page 12 of Manuscript |
| Declaration of interests | 28 | Financial and other competing interests for principal investigators for the overall trial and each study site | Page 18 of submitted manuscript |
| Access to data | 29 | Statement of who will have access to the final trial dataset, and disclosure of contractual agreements that limit such access for investigators | Page 12 of Manuscript |
| Ancillary and post-trial care | 30 | Provisions, if any, for ancillary and post-trial care, and for compensation to those who suffer harm from trial participation | Page 9 of Manuscript |
| Dissemination policy | 31a | Plans for investigators and sponsor to communicate trial results to participants, healthcare | Section 10, page 33 of study protocol |

For peer review only

| | | | |
|---|---|---|---|
| | | professionals, the public, and other relevant groups (eg, via publication, reporting in results databases, or other data sharing arrangements), including any publication restrictions | Pages 15-16 of Manuscript |
| | 31b | Authorship eligibility guidelines and any intended use of professional writers | Page 24 of Manuscript |
| | 31c | Plans, if any, for granting public access to the full protocol, participant- level dataset, and statistical code | Page 24 of Manuscript |
| **Appendices** | | | |
| Informed consent materials | 32 | Model consent form and other related documentation given to participants and authorised surrogates | Appendix 1 of Manuscript |
| Biological specimens | 33 | Plans for collection, laboratory evaluation, and storage of biological specimens for genetic or molecular analysis in the current trial and for future use in ancillary studies, if applicable | Appendix 3 of Manuscript |

For peer review only

BMJ Open

# Topical chlorhexidine 0.2% versus topical natamycin 5% for fungal keratitis in Nepal: rationale and design of a randomised controlled non-inferiority trial



| | |
|---|---|
| Journal: | *BMJ Open* |
| Manuscript ID | bmjopen-2020-038066.R3 |
| Article Type: | Protocol |
| Date Submitted by the Author: | 24-Jul-2020 |





| | |
|---|---|
| Complete List of Authors: | Hoffman, Jeremy; London School of Hygiene and Tropical Medicine International Centre for Eye Health; Sagarmatha Choudhary Eye Hospital<br>Yadav, Reena; Sagarmatha Choudhary Eye Hospital<br>Das Sanyam, Sandip; Sagarmatha Choudhary Eye Hospital<br>Chaudhary, Pankaj; Sagarmatha Choudhary Eye Hospital<br>Roshan, Abhishek; Sagarmatha Choudhary Eye Hospital<br>Singh, Sanjay; Eastern Region Eye Care Programme<br>Arunga, Simon; London School of Hygiene and Tropical Medicine International Centre for Eye Health, International Centre for Eye Health; Mbarara University of Science and Technology Faculty of Medicine<br>Matayan, Einoti; Kilimanjaro Christian Medical Centre, Department of Ophthalmology<br>Macleod, David; London School of Hygiene & Tropical Medicine, MRC Tropical Epidemiology Group<br>Weiss, Helen; London School of Hygiene & Tropical Medicine, MRC Tropical Epidemiology Group<br>Leck, Astrid; London School of Hygiene and Tropical Medicine International Centre for Eye Health<br>Hu, Victor; London School of Hygiene and Tropical Medicine International Centre for Eye Health<br>Burton, Matthew J; London School of Hygiene and Tropical Medicine International Centre for Eye Health; Moorfields Eye Hospital NHS Foundation Trust |





| | |
|---|---|
| <b>Primary Subject Heading</b>: | Ophthalmology |
| Secondary Subject Heading: | Evidence based practice, Global health, Infectious diseases, Research methods |
| Keywords: | Corneal and external diseases < OPHTHALMOLOGY, OPHTHALMOLOGY, Clinical trials < THERAPEUTICS, MYCOLOGY |
| | |



SCHOLARONE™
Manuscripts

*I, the Submitting Author has the right to grant and does grant on behalf of all authors of the Work (as defined in the below author licence), an exclusive licence and/or a non-exclusive licence for contributions from authors who are: i) UK Crown employees; ii) where BMJ has agreed a CC-BY licence shall apply, and/or iii) in accordance with the terms applicable for US Federal Government officers or employees acting as part of their official duties; on a worldwide, perpetual, irrevocable, royalty-free basis to BMJ Publishing Group Ltd ("BMJ") its licensees and where the relevant Journal is co-owned by BMJ to the co-owners of the Journal, to publish the Work in this journal and any other BMJ products and to exploit all rights, as set out in our licence.*

*The Submitting Author accepts and understands that any supply made under these terms is made by BMJ to the Submitting Author unless you are acting as an employee on behalf of your employer or a postgraduate student of an affiliated institution which is paying any applicable article publishing charge ("APC") for Open Access articles. Where the Submitting Author wishes to make the Work available on an Open Access basis (and intends to pay the relevant APC), the terms of reuse of such Open Access shall be governed by a Creative Commons licence – details of these licences and which Creative Commons licence will apply to this Work are set out in our licence referred to above.*

*Other than as permitted in any relevant BMJ Author's Self Archiving Policies, I confirm this Work has not been accepted for publication elsewhere, is not being considered for publication elsewhere and does not duplicate material already published. I confirm all authors consent to publication of this Work and authorise the granting of this licence.*

For peer review only

# Topical chlorhexidine 0.2% versus topical natamycin 5% for fungal keratitis in Nepal: rationale and design of a randomised controlled non-inferiority trial


**Authors:**

Jeremy J Hoffman FRCOphth (1, 2, 5), Reena Yadav MD (2), Sandip Das Sanyam (2), Pankaj Chaudhary (2), Abhishek Roshan (2), Sanjay K Singh MD (3), Simon Arunga (1, 4), Einoti Matayan (5), David Macleod PhD (1, 6), Helen A Weiss PhD (6), Astrid Leck PhD (1), Victor H Hu PhD FRCOphth (1), Matthew J Burton PhD FRCOphth (1, 7).

**Affiliations:**

1. International Centre for Eye Health, London School of Hygiene and Tropical Medicine, London, UK
2. Sagarmatha Choudhary Eye Hospital, Lahan, Nepal
3. Eastern Region Eye Care Programme, Biratnagar, Nepal
4. Mbarara University of Science and Technology, Mbarara, Uganda
5. Kilimanjaro Christian Medical Centre, Moshi, Tanzania
6. MRC Tropical Epidemiology Group, London School of Hygiene & Tropical Medicine, London, UK
7. Moorfields Eye Hospital, London, UK

**Corresponding author:**

Jeremy J Hoffman FRCOphth

International Centre for Eye Health

London School of Hygiene and Tropical Medicine

Keppel Street

London

WC1E 7HT

United Kingdom

jeremy.hoffman@lshtm.ac.uk


**Key words:** Clinical trial; fungal keratitis; chlorhexidine; natamycin; corneal ulcer, Nepal.

**Word count:** 5132 (excluding abstract, titles, tables, figures and references)

For peer review only

## ABSTRACT

### Introduction

Fungal infections of the cornea, fungal keratitis (FK), are challenging to treat. Current topical antifungals are not always effective and are often unavailable, particularly in low and middle-income countries where most cases occur. Topical natamycin 5% is usually first-line treatment, however, even when treated intensively, infections may progress to perforation of the eye in around a quarter of cases. Alternative anti-fungal medications are needed to treat this blinding disease.

Chlorhexidine is an antiseptic agent with antibacterial and antifungal properties. Previous pilot studies suggest that topical chlorhexidine 0.2% compares favourably with topical natamycin. Full-scale randomised controlled trials (RCT) of topical chlorhexidine 0.2% are warranted to answer this question definitively.

### Methods and analysis

We will test the hypothesis that topical chlorhexidine 0.2% is non-inferior to topical natamycin 5% in a two-arm, single-masked RCT. Participants are adults with FK presenting to a tertiary ophthalmic hospital in Nepal. Baseline assessment includes history, examination, photography, *in vivo* confocal microscopy and cornea scrapes for microbiology. Participants will be randomised to alternative topical antifungal treatments (topical chlorhexidine 0.2% and topical natamycin 5%; 1:1 ratio, 2-6 random block size). Patients are reviewed at day 2, day 7 (with re-culture), day 14, day 21, month 2 and month 3. The primary outcome is best spectacle corrected visual acuity (BSCVA) at three months. Primary analysis (intention-to-treat) will be by linear regression, with treatment arm and baseline BSCVA pre-specified covariates. Secondary outcomes include epithelial healing time, scar/infiltrate size, ulcer depth, hypopyon size, perforation and/or therapeutic penetrating keratoplasty (TPK, corneal transplant), positive re-culture rate (day-7) and quality of life (EQ-5D, WHO/PBD-VF20, WHOQOL-BREF).

### Ethics and dissemination

The Nepal Health Research Council, the Nepal Department of Drug Administration and the London School of Hygiene and Tropical Medicine ethics committee have approved the trial. The results will be presented at local and international meetings and submitted to peer-reviewed journals for publication.

### Trial Registration

ISRCTN14332621; pre-results.

### Article summary


For peer review only - http://bmjopen.bmj.com/site/about/guidelines.xhtml

- First large-scale randomised controlled clinical trial comparing chlorhexidine 0.2% to natamycin 5% for the treatment of fungal keratitis.
- This study benefits from a pragmatic design: as a non-inferiority trial, if chlorhexidine is found to be within a pre-defined non-inferiority margin of 0.15 logMAR of natamycin at 3 months a recommendation to use chlorhexidine 0.2% can be made; this is a far cheaper, easy to formulate medication that could significantly increase access to anti-fungal treatment for the target population.
- Clinicians are masked to the treatment allocation, however, due to different physical appearance it is not possible to mask patients to their allocated treatment.
- This study will also assess the superiority of either medication as well as a number of key secondary outcome measures, analysed by arm.
- First randomised controlled trial investigating fungal keratitis to use *in vivo* confocal microscopy as a diagnostic tool for the detection of fungal elements.

For peer review only


For peer review only - http://bmjopen.bmj.com/site/about/guidelines.xhtml

## INTRODUCTION

Fungal keratitis (FK) is a severe and potentially blinding corneal infection (Figure 1). [1,2] The burden is greatest in tropical and subtropical countries, probably due to a combination of climate (higher temperatures and humidity) and frequent agriculture related eye injuries. [3] It is one of the causes of microbial keratitis (MK) and accounts for between 20 – 60% of corneal infections diagnosed in tropical regions. [4] It is often inadequately treated with significant barriers to receiving appropriate, timely intervention, compounded by indiscriminate and inappropriate use of conventional medicines such as topical corticosteroids or harmful traditional eye medicines. [1,2,5] Furthermore, when appropriate treatment is available, up to 30% of patients receiving current "gold-standard" therapy progress to corneal perforation and / or eye-loss (Figure 2). [1,6,7]

Treatment for fungal keratitis almost always involves topical antifungal agents. Surgical intervention, usually in the form of therapeutic penetrating keratoplasty (TPK), is generally reserved for cases of corneal perforation or progressive infection refractory to medical therapy. Corneal transplantation is also performed for visual rehabilitation after the acute infection has resolved. There are a limited number of antifungals available for treating FK, which fall into four main groups: imidazoles, triazoles, polyenes and fluorinated pyrimidines. These may be available topically, orally or by intravenous injection. Subconjunctival, corneal stromal or intracameral injections may also be given. The treatment of yeasts (*Candida spp.*) is often different to filamentous fungi, with the former being more common in temperate climates and the latter in hot and humid locations.[8,9]

There have been several clinical trials comparing treatment options for FK, which have been systematically reviewed.[10,11] Natamycin, which was approved in the 1960s by the FDA for FK, has been compared to a number of newer agents, including voriconazole. Natamycin and voriconazole have been compared in four trials, with the meta-analysis favouring natamycin. [6,10,12-14]

As a result, first line management of filamentous FK is usually with topical natamycin 5% when this is available. This was added to the WHO Essential Medicines List in 2017 for this indication. However, even when intensive topical natamycin is initiated, infections frequently progress relentlessly to perforation and loss of the eye in about a quarter of cases, Figure 2. [1,6,7] Moreover, in many countries antifungal eye drop treatments are simply not available. This includes most countries in SSA, some Asian countries and some countries in Europe.[1,2] Natamycin is relatively expensive even if it is available. Therefore, additional alternative and more affordable drugs are clearly needed if the outcome of these infections is to improve.

For peer review only

Chlorhexidine (CHX) is an antiseptic agent, with both antibacterial and antifungal properties. It is a widely-used broad-spectrum biocide, killing microorganisms through cell membrane disruption. [15-17] CHX has been used in ophthalmology for over 30 years as an eye-drop preservative and for sterilizing contact lenses, and has also been used to treat *Acanthamoeba* and fungal keratitis. [9,18-22] In a study of potential anti-fungal treatments, CHX was effective both *in vitro* against FK isolates from India and Ghana, as well as in an Indian case series.[23] Subsequently two pilot RCTs of CHX for fungal keratitis were conducted. In the first, three CHX concentrations (0.05%, 0.1%, 0.2%) were compared to each other and natamycin 5%; this concluded CHX 0.2% had reliable antifungal action. [19] The same concentration is used in mouthwash to prevent oral candidiasis. In the second trial, CHX 0.2% was compared to topical natamycin 2.5% (half standard concentration). There was evidence CHX produced a more favourable response by 5 days (RR=0.23, 95%CI 0.09-0.63). [18] A systematic review found a trend favouring CHX over natamycin in "curing" by 21-days (RR=0.70, 95%CI 0.45-1.09), suggesting CHX might prove superior in adequately powered trials.[10] CHX is safe and well-tolerated at these concentrations. [18,19,22,24] Based on this, CHX is used for treating FK in several countries. [1,9,11] However, the combined size of these two pilot trials comparing chlorhexidine and natamycin is not sufficient to reach firm conclusions. Currently, the meta-analysis indicates equipoise in terms of which treatment is best for treating fungal keratitis.

**Objectives**

The primary objective of this study is to determine if topical chlorhexidine 0.2% is non-inferior to topical natamycin 5% for treating filamentous fungal keratitis, in terms of vision at three months. The secondary objectives are: (1) to determine whether either treatment (chlorhexidine 0.2% or natamycin 5%) is superior to the other, in terms of vision at three months; and (2) to determine whether there is a difference between chlorhexidine 0.2% and natamycin 5% in terms of secondary clinical outcomes including infiltrate / scar size, time to re-epithelialisation, re-culture rates at one week and the effect of the alternative treatments on the Quality of Life of participants.

Chlorhexidine is cheap, stable and easily prepared by aqueous dilution. If chlorhexidine is found to be non-inferior (or even superior) to natamycin this offers the potential of an effective, affordable and accessible treatment for fungal keratitis, which could benefit millions of people each year who currently have no treatment options. This trial is a response to this expressed need from both clinicians and patients for a readily available and affordable medication for fungal infection.

For peer review only



For peer review only - http://bmjopen.bmj.com/site/about/guidelines.xhtml

## METHODS AND ANALYSIS

### Trial Design

We are conducting a single-masked, non-inferiority randomised controlled trial comparing chlorhexidine 0.2% to natamycin 5% for the treatment of fungal keratitis. The non-inferiority design of this trial offers a clinically pragmatic way to address this important question: if CHX is found to be within the pre-specified non-inferiority margin of 0.15 logMAR (about 1.5 Snellen lines) then CHX may prove to be a sustainable solution for this aspect of the complex problem of fungal keratitis.

### Trial Summary

This RCT follows a two-stage recruitment process (Figure 3). All patients presenting with acute microbial keratitis are reviewed and enrolled into Stage 1, following written, informed consent. This involves history, examination and investigations (corneal scrapes for microbiological assessment and *in vivo* confocal microscopy). If there is evidence of fungal hyphae on smear or confocal microscopy, patients then proceed to Stage 2. A trial eligibility checklist is completed and Stage 2 written informed consent is conducted. We will recruit 500 patients into Stage 2. Eligible FK patients are then randomised 1:1 to receive either natamycin 5% or chlorhexidine 0.2% topical treatments hourly for the first week, then two-hourly for the subsequent two weeks. Ongoing treatment duration will then be tailored to clinical response. Study personnel are masked to the treatment allocation. Patients are usually initially admitted and followed up on day-2, day-7 (with re-culture), day-14, day-21, month-2 and month-3. The primary outcome is best spectacle corrected visual acuity (BSCVA) at three months.

### Trial Setting

Low-land Nepal, a region with a high burden of fungal keratitis, provides a suitable location to conduct a clinical trial that requires a relatively large sample size. This trial will be conducted in Sagarmatha Choudhary Eye Hospital (SCEH), Lahan, Siraha District in south eastern Nepal. SCEH treats approximately 800 cases of keratitis per month, half of which is attributable to fungal infection. [1,25] SCEH is a tertiary level ophthalmic hospital, with several satellite Eye Care Clinics (ECCs) that refer patients to SCEH directly as required. SCEH and its ECCs serve an estimated population of about 5 million people. Due to its proximity to the border, approximately 50% of the outpatients are Indian nationals. It is anticipated that the study participants will present to the hospital from multiple districts within the region. Potentially eligible individuals will be recruited from outpatient clinics or referred directly by the ECCs to the study team. Based on the numbers of patients attending, it should be possible to complete recruitment of 500 participants within six to twelve months. A second separate trial in East Africa (Tanzania and Uganda) will also be conducted to compare chlorhexidine 0.2% to natamycin 5% following a very similar protocol to the one described here. This will be

For peer review only

registered as a separate trial. This will enable us to assess the generalisability of the findings in two geographically distinct regions with potentially different fungal aetiologies and susceptibility patterns.

**Eligibility Criteria**

Potential participants need to meet all the inclusion criteria and have none of the exclusion criteria listed in Table 1. In summary, they need to have an active fungal keratitis defined as acute microbial keratitis characterised by corneal epithelial ulceration (>1mm in diameter), corneal stromal infiltrate and signs of acute inflammation (e.g. conjunctival injection, anterior chamber inflammatory cells, hypopyon) in conjunction with evidence of a filamentous fungal infection on smear microscopy and/or *in vivo* confocal microscopy. There is strong evidence supporting the use of IVCM for diagnosing filamentary fungal keratitis. Studies have reported sensitivities of 85.7 – 89.2% and specificities of between 81.4 and 92.7%, respectively. [26-28] As some patients will be enrolled on the basis of the results of *in vivo* confocal microscopy which is unable to detect most bacteria reliably, some patients with microscopically confirmed fungal infection will subsequently also be found to have had mixed infection at the time of being recruited into the study, as bacterial cultures may become positive a few days after enrolment. Based on previous experience at SCEH this is likely to account for about 10% of cases. These patients are included in the study but excluded from the primary analysis of the primary outcome (see below). Secondary analyses will include mixed infections.

For peer review only

**Table 1: Inclusion and exclusion criteria for enrolment in Stage 1 (microbial keratitis cases) and Stage 2 (the randomised controlled trial)**

| **Stage 1:** | |
|---|---|
| **Inclusion Criteria (all must be met):** | **Exclusion Criteria (any of the following):** |
| 1. Acute MK characterised by:<br>• Corneal epithelial ulceration >1mm diameter<br>• Corneal stromal infiltrate<br>• Acute inflammation: e.g. conjunctival injection, anterior chamber inflammatory cells, hypopyon.<br>2. Adults (18 years and older)<br>3. Able to provide informed consent | 1. Patients aged less than 18 years<br>2. Patients unable or unwilling to provide informed consent<br>3. Patients who do not have acute microbial keratitis or where there is a more likely alternative diagnosis |
| **Stage 2:** | |
| **Inclusion Criteria (all must be met):** | **Exclusion Criteria (any of the following):** |
| 1. Acute MK characterised by:<br>• Corneal epithelial ulceration >1mm diameter<br>• Corneal stromal infiltrate<br>• Acute inflammation: e.g. conjunctival injection, anterior chamber inflammatory cells, hypopyon.<br>2. Filamentous fungal hyphae visualised on smear microscopy and/or *in vivo* confocal microscopy.<br>3. Agree to be randomised to either treatment arm and are able to give informed consent<br>4. Agree to be followed up at 2 days, 1 week, 2 weeks, 3 weeks, 2 months and 3 months<br>5. Adults (18 years and older) | 1. Unwilling/unable to participate in trial and/or attend follow-up<br>2. Aged less than 18 years<br>3. Pregnancy: self-reported, or by urine hCG pregnancy test if uncertain.<br>4. Breast feeding: self-reported<br>5. Prior topical anti-fungal treatment<br>6. No light perception in the affected eye<br>7. Fellow eye visual acuity <6/60<br>8. Acanthamoebic infection visualised by smear microscopy or IVCM<br>9. Clinical evidence of herpetic keratitis<br>10. Known allergy to study medication (including preservatives)<br>11. Previous keratoplasty in the affected eye<br>12. Bilateral corneal ulcers<br>13. Very severe ulcers warranting immediate evisceration or conjunctival flap<br>14. Endophthalmitis |



For peer review only - http://bmjopen.bmj.com/site/about/guidelines.xhtml

**Consent Procedures**

There are two independent consent stages in this trial: Stage 1, where all adult patients with microbial keratitis are eligible; and Stage 2, only for fungal keratitis patients meeting the eligibility criteria. The two-stage process enables data collection on all potential patients at baseline before a diagnosis of fungal keratitis is confirmed. All patients who are eligible to participate will be given a participant information sheet in Nepali and its contents read to them. There will be an opportunity to discuss any questions that they might have. If the patient would like to participate, they will be asked to read and sign or place a thumb print on the study consent form. The consent will be witnessed by the eye health worker, confirmed by a signature on the form. For patients who are unable to read the documentation a second witness who is unrelated to the study is required. Consent Forms are given in Appendix 1.

**Baseline Assessment**

The detailed baseline assessment is described in Table 2. This includes clinical examination, corneal photography, *in vivo* confocal microscopy and the collection of microbiology samples. Quality of life questionnaires will also be completed (EQ-5D, WHO/PBD-VF20, WHOQOL-BREF), details of the scoring for these are given in Table 2.

For peer review only

**Table 2: Baseline assessment.** Assessment performed at baseline with details of how they are made.

| Assessment | Details |
|---|---|
| Visual Acuity | Presenting, Pin-Hole and Best Spectacle Corrected Visual Acuity (BSCVA) will be measured using an ETDRS Tumbling-E logMAR 3m chart (Good-Lite Inc., USA) mounted on an ESC 2000 ETDRS LED Cabinet, (Good-Lite Inc, USA) by a trial-certified optometrist, for each eye separately |
| Contrast Sensitivity | Measured using the Peek Contrast Sensitivity smartphone application running on Android OS with a Sony Xperia Z3 Compact smartphone (Sony, Japan).[26] |
| Clinical Photographs | Photographs will be taken separately of both corneas using a Nikon D7500 camera with an AF-S Micro Nikkor 105mm lens and lens mounted SB-200 flash units (Nikon, Japan). A standardised photography protocol is used to ensure images can be compared between time points. Standardised magnification will be used to allow epithelial defect and stromal infiltrate size measurements to be made. |
| Slit-lamp examination | Both eyes will be examined using a slit-lamp biomicroscope (standard ophthalmology examination) to assess the anterior segment of the eye. This examination will be performed by an ophthalmic clinician experienced in managing microbial keratitis. Particular attention will be paid to the following features:<br>1. Eyelids: trichiasis, lagophthalmos, facial weakness, Bell's reflex<br>2. Suppuration<br>3. Conjunctival inflammation<br>4. Corneal sensation<br>5. Cornea epithelial defect (measuring the longest dimension and the longest perpendicular) and ulcer depth<br>6. Corneal inflammatory infiltrate depth, size, profile, colour, edge pattern, texture, satellites.<br>7. Anterior chamber inflammatory cells, hypopyon, endothelial plaque<br>8. Relative afferent pupillary defect. |
| *In vivo* confocal microscopy (IVCM) | The Heidelberg Retinal Tomograph 3 (HRT3) *in vivo* confocal microscope enables the clinician to examine the cornea down to the cellular level. It is able to detect the presence of fungal hyphae.[27,28] A sterile, single-use disposable cap covers the objective lens and is changed between patients. Volume scans will be performed which provide a series of 400 x 400µm images over a depth range of 80µm. The resolution of the corneal scanning module is 7.6µm. IVCM images will be collected in a systematic way, starting at the centre of the ulcer, then at the superior, inferior, nasal and temporal borders of the ulcer. Volume scans will be performed in all of these locations, starting at the level of the corneal epithelium, and ending at the deepest affected aspect of the cornea assessed from IVCM images. Images will be assessed during the examination. |
| Ocular Sample Collection | The following samples will be collected from the corneal ulcer of each patient at the baseline assessment:<br>1. Corneal scrape specimens for microscopy and microbiological culture. A corneal scrape will be collected from the corneal ulcer after application of preservative free proxymetacaine local anaesthetic eye drops (Minims). Sterile needles are used to take corneal scrape specimens and then place on to glass slides for immediate Gram stain, KOH, and Calcofluor white. Samples will be directly inoculated onto blood, chocolate, Sabouraud agar and broths for culture.<br>2. Corneal specimen collection for PCR. Two sterile swabs will be gently swept over the surface of the corneal ulcer and placed into a 2ml tube. The swabs will be for pathogen detection by PCR, fungal sequencing and assessment of point of care tests for fungal infections. Swabs will be stored dry at -80°C. If swab yields are found to be too low for analysis an additional corneal scrape will be collected for PCR. The analysis of the PCR samples will not form part of the RCT work up and report. |
| HIV Testing | All individuals presenting with MK would be offered counselling and testing services. If this is found to be positive and the patient is unknown to the HIV care services an appropriate referral will be made. HIV testing is performed using HIV Tri-Dot rapid diagnostic test (J. Mitra & Co. Pvt. Ltd., India) |



For peer review only - http://bmjopen.bmj.com/site/about/guidelines.xhtml

| | |
|---|---|
| Random Blood Glucose | There is a suggestion that individuals with diabetes may be more susceptible to fungal keratitis. Participants will be offered a random blood glucose test, on a finger prick sample, analysed using HumaLyzer Primus (HUMAN Gesellschaft für Biochemica und Diagnostica mbH, Germany). If this is above 6.1 mmol/l they will be referred to the hospital physicians for assessment and formal diagnosis of impaired glucose tolerance or diabetes mellitus. This level is considered a suitable cut-off to detect individuals with diabetes and has been validated in a south-Asian population.[29] |
| Quality of Life Questionnaires | For those with confirmed fungal keratitis and who are enrolled in the trial, there will be several additional baseline assessments to evaluate the impact of FK on quality of life.<br><br>Vision Related Quality of Life (VRQoL): will be assessed by a vision disease specific tool the WHO/PBD-VF20.[30] This tool measures the impact of visual impairment in the person's life including mental wellbeing, dependency and social functioning. These have been used in a number of other vision related studies to show a difference in QoL.[31,32] This instrument consists of 20 questions divided into three sub-scales: visual symptom, general functioning and psychosocial. It begins by asking the patient *"Overall, how would you rate your eyesight using both eyes?"*; and uses a five point scale answer option such as *"very good", "good", "moderate", "bad", "very bad"*. The test is scored out of 100, with higher scores reflecting a better VRQoL.<br><br>General Health Related Quality of Life (HRQoL): We will use the EQ-5D questionnaire and EQ-Visual Analogue Scale. The EQ-5D is a standardised tool to measure health outcomes.[33] Patients will also be assessed using the WHOQOL-BREF.[34] This has good applicability in low and middle-income countries (LMIC) as it was developed simultaneously from concept across 18 countries in Africa, Asia and Latin America. It measures 4 domains of health: Physical Health, Psychological Health, Social Relationships, and Environment. It asks respondents 26 questions how much (frequency) they have experienced and/or were able to do things (e.g. feel safe, able to concentrate, enjoy life) in the past 4 weeks and how satisfied they are with certain aspects of their lives (e.g. sleep, capacity for work). Each question is scored between one and five in a positive direction, with one being attributed for a very low or dissatisfied quality of life, and five being very good or very satisfied with their quality of life (i.e. higher scores denote a higher quality of life). Each domain has its own score calculated by calculating the mean of the item scores within each domain. In addition, there are two items that are examined separately: question 1 asks about an individual's overall perception of quality of life and question 2 asks about an individual's overall perception of their health. These are scored on the same positive scale from one to five. The mean domain scores can then be multiplied by four in order to make domain scores comparable with the scores used in the WHOQOL-100.[34] |


For peer review only - http://bmjopen.bmj.com/site/about/guidelines.xhtml

**Randomisation and Masking**

***Sequence generation***: a computer-generated randomisation list will be prepared by an independent statistician at LSHTM, who will hold the sequence, will not be masked, and will not be involved in any other aspect of the trial. The sequence is in a 1:1 allocation ratio of CHX to NATA, with a random block size (2, 4 or 6).

***Allocation concealment and implementation:*** the randomisation sequences will be concealed in sequentially numbered, opaque envelopes. The envelopes will be prepared by a person independent of all other aspects of the trial. The randomisation administrator (nurse or pharmacist) will conduct the random allocation procedure. The investigational products will be stored in the trial coordination office in a locked and separate drug cabinet dedicated for this clinical trial only. The cabinet will only be used for drug storage and will only be accessed by the randomisation administrator. The randomisation administrator will work in a separate room in the clinic. After the patient has been recruited and assessed, they will be guided to this separate room where the randomisation allocation will be conducted.

***Masking:*** The topical treatments being compared in this trial have a different appearance: the chlorhexidine 0.2% is a clear, colourless solution and the natamycin 5% is an opaque, white suspension. Therefore, it is not possible to mask the participants to this difference in visual appearance. However, the patients will not be told which treatment they have been allocated. Prior to any follow-up clinical examinations, a nurse, otherwise uninvolved in the study, will wipe away any white natamycin residue from the patient's eyes to avoid unmasking the clinical assessor. This procedure was used successfully in other trials.[6] All clinicians involved in the clinical assessment of the patients will be masked to the allocation. The statistician who will perform the primary analysis will be masked to the allocation and only receive the actual allocation code sequence from the independent statistician after the analysis code has been prepared and pre-tested with a test sequence. The primary outcome is visual acuity at 3-months assessed by an optometrist who will not have been involved in any other aspects of the trial and masked to the allocation. By three months the treatment courses are likely to be completed. There will be masked grading of the photographs to independently confirm outcome measures and assess for any systematic bias on the part of the clinical examiners.

***Unmasking:*** Unmasking is a serious action and should only be performed if necessary to ensure the safety of a study participant. Anyone unmasked to randomised treatment for the purpose of creating analyses for independent data and safety monitoring committees will not be involved in any other aspects of the conduct or final analysis of the study. A list will be maintained of all unmasked members of staff and will be approved by the Chief Investigator. Such staff members must sign a document to indicate that they are aware of their



For peer review only - http://bmjopen.bmj.com/site/about/guidelines.xhtml

responsibilities with respect to confidentiality. The processes used to provide access to unmasked treatment codes and reports relating to these codes shall be documented.

**Intervention and Treatment**

The standard of care in this hospital is for cases of fungal keratitis to be admitted. If willing, patients will be admitted for close observation and supervised treatment, until there are signs of improvement and the supervising clinician considers it safe to provide ongoing management on an outpatient basis.

***Trial Treatment Arms***

A) *Chlorhexidine 0.2% w/v eye drops*

Chlorhexidine 0.2% w/v solution, as eye drops, will be applied to the surface of the infected eye (one drop per application). The chlorhexidine 0.2% w/v eye drops used in these studies is produced by Mandeville Medicines, UK.

B) *Natamycin 5% eye drops*

Natamycin 5% w/v suspension, as eye drops, will be applied to the surface of the infected eye (one drop per application) Topical natamycin 5% is produced by FDC Pharmaceuticals, India.

***Dosing Schedule:*** Both trial treatment arms will follow the same dosing schedule. Eye drops will be given hourly day and night for 48 hours, then hourly whilst awake for five days, and then two-hourly whilst awake for two further weeks. If the ulcer has healed (epithelial defect less than 1mm, infiltrate resolved, with or without corneal scarring), then treatment is stopped. If resolving stromal infiltration and/or epithelial defect >1mm but < 5mm, treatment is reduced to four times daily. If resolving but with epithelial defect > 5mm and/or stromal infiltration / hypopyon, treatment is reduced to six times daily. Treatment duration will be tailored to clinical response, with patients reviewed regularly whilst on treatment in addition to their scheduled study visits.

***Topical treatments:*** Several other topical medicines are used in the standard care of people with corneal infections:

1. **Fluorescein sodium ophthalmic strips** (Contacare Ophthalmics and Diagnostics, India) to highlight the area of cornea epithelial defect.
2. **Anaesthetic eye drops** to anaesthetise the cornea before procedures such as microbiology samples: Proxymetacaine 0.5% eye drop Minims (Bausch and Lomb, UK).
3. **Antibiotic eye drops** may be used as per the judgement of the treating ophthalmologist if there is a suspicion of mixed bacterial and fungal keratitis: Moxifloxacin 0.5% eye drops (Centaur Pharmaceuticals, India).



For peer review only - http://bmjopen.bmj.com/site/about/guidelines.xhtml

4. **Mydriatic eye drops** for pupil dilation to reduce discomfort from the infection: Cyclopentolate 2% eye drops (Aurolab, India), three times a day.
5. **Ocular hypotensive eye drops** if the intra-ocular pressure is elevated >25 mmHg. This will be at the discretion of the supervising clinician; usual first line treatment is timolol 0.5% eye drops (Allergan, India).

***Ancillary treatment in patients refractory to trial medication:*** In patients with progressive fungal keratitis despite 7 days or more of trial medication, additional treatment options will be considered and offered. After repeating microbiological tests to rule out a mixed bacterial infection, deteriorating patients are started on topical voriconazole 1% hourly if the ulcer is superficial only. For ulcers that are deeper than 75% of the corneal thickness, oral ketoconazole 200mg twice-daily is added (with monitoring of liver function). The choice of ancillary treatment is due to local availability. If, following 7 days of additional treatment, despite these measures there is ongoing progression, surgical management such as a TPK can be considered.

***Non-pharmacological treatment:*** It is sometimes necessary to perform surgical procedures during the management of corneal infections. In the context of the trial these will be performed by the supervising consultant ophthalmologist. These can include:

1. Insertion of a bandage contact lens for very small perforations.
2. Tissue glue and patch for small perforations
3. Corneal transplant (TPK) for progressive fungal infections that are refractory to medical management or large perforations.
4. Conjunctival flaps to cover corneas in non-healing ulcers.

**Outcome Measures**

***Primary Outcome Measure:***

The primary outcome will be best spectacle corrected visual acuity (BSCVA) in logMAR units at three months. This will be measured by a trial-certified optometrist, independent of all other aspects of the study and masked to allocation. This has been the primary outcome of several other trials and will therefore facilitate comparison. Three months is the time at which clinical experience suggests most corneal ulcers have usually healed. BSCVA is chosen as it is easy to measure and is of functional significance. We will measure the BSCVA using an LED-backlit, Tumbling-E LogMAR chart (Good-Lite Co., IL, USA) under controlled conditions. We will also measure vision using Peek Acuity, a validated smartphone application, in situations where the patient is unable to attend the hospital for follow-up and outcome data is only available through a domiciliary visit. [35] For patients who have CF vision or less, pre-defined logMAR values will be assigned based on previous clinical trials. [36,37] Similarly, for patients who undergo a corneal transplant (therapeutic penetrating keratoplasty, TPK), a pre-defined visual acuity of 1.9 logMAR will be given, or last observation carried forward (LOCF, whichever is the better vision), as per previous studies. [6]


For peer review only - http://bmjopen.bmj.com/site/about/guidelines.xhtml

***Secondary Outcome Measures:***

We will be assessing a number of secondary outcome measures that relate to different measures of visual acuity such as Peek Acuity; clinical signs of healing such as reduction of epithelial defect; microbiological culture rates; and other clinical outcome measures such as scar size or perforation rate. These secondary outcomes together with their analyses are outlined in the Analysis Plan below.

**Outcome Assessments**

Participants will be re-assessed at 2-days, 1-week, 2-weeks, 3-weeks, 2-months and 3-months following enrolment. Additional examinations, outside the trial protocol schedule may be conducted by the supervising clinician as indicated. The specific assessments to be carried out at each visit are indicated in Table 3. These will be conducted in the same way described for the baseline assessment as described (Table 2). At each follow-up assessment the participant will be asked about adherence to treatment and symptoms, including side effects, if still receiving trial treatment. This will be recorded in the Case Record File. To monitor trial medication adherence, the study participants will be asked to bring their eye drop bottles to the 1, 2- and 3-week follow-ups. The amount of remaining medication will be measured by weighing the bottle and will be compared with the anticipated remaining amount provided the drops were being used as instructed, resulting in a ratio of actually remaining to what is anticipated. Participants will be resupplied with medication as needed. The presenting visual acuity will be measured on each visit. In addition, the BSCVA will also be measured at the three-month follow-up (primary outcome measure). At the final 3-month follow-up the three quality of life questionnaires will be repeated. On each occasion the eyes will be examined using a slit-lamp and the cornea photographed. The *in vivo* confocal microscopy will be repeated at 1, 2 and 3 weeks to determine whether there is evidence of fungal hyphae resolution with the fragmentation of linear elements. At the 1-week follow-up if the ulcer has not healed it will be re-scraped for repeat culture to determine whether the keratitis is now culture-positive or culture-negative. We will give the study participants appointment cards for the next follow-up and they will be reminded about the follow-up a week prior to the date. Public transport costs will be paid for participants who are outpatients.

For peer review only

**Table 3: Baseline and follow-up assessment components.**

| Assessment Item | Baseline | Day 2 | Day 7 | Day 14 | Day 21 | Day 60 | Day 90 |
|---|---|---|---|---|---|---|---|
| History / Baseline questionnaire | X | | | | | | |
| Check treatment adherence | | X | X | X | X | X | X |
| Check for side effects | | X | X | X | X | X | X |
| Visual Acuity – Presenting | X | X | X | X | X | X | X |
| Visual Acuity – BSCVA | X | | | | | | X |
| Contrast Sensitivity | X | | | | | | X |
| Slit-lamp Examination | X | X | X | X | X | X | X |
| Cornea Photography | X | X | X | X | X | X | X |
| *In vivo* confocal microscopy | X | | X | X | X | | |
| Cornea samples (Microbiology/PCR) | X | | X | | | | |
| Quality of Life Tools | X | | | | | | X |

**Treatment Review**

At each follow-up visit participants will be reviewed by an ophthalmic clinician experienced in the management of fungal keratitis. Clinical responses to anti-fungal therapy tend to be relatively slow (compared to bacterial infections). Prolonged topical treatment courses of 4-6 weeks are usually needed. Therefore, a change in therapy is usually not made for at least the first week. Other interventions may be indicated such as application of glue to corneal perforations, conjunctival flaps or therapeutic penetrating keratoplasty (corneal transplant).

**Stopping Rules**

If the study eye develops any serious adverse outcomes, the antifungal study medication may be discontinued if it is considered to be responsible for the adverse event. The patient will then be treated at the discretion of the supervising ophthalmologist, without breaking the randomisation code. If the medication is stopped, the patient will continue with the scheduled follow-up examinations.

**Loss to Follow-up**

Loss to follow-up rates are expected to be low, based on clinical experience. Participants who do not present for their follow-up visit will be contacted by telephone. Reasons for the loss to follow-up will be identified. Patients will be counselled about the importance of attending for ongoing treatment and monitoring. If they are unwell or unable to attend the hospital for some specific reason the study team will arrange to visit them in their home. Reasons for loss to follow-up will be recorded and reported.

**Data collection, management, confidentiality and access to data**

Data will be collected using paper Clinical Record Forms (CRFs). These will be stored securely at SCEH and scanned electronic copies taken at the end of the day, stored on an encrypted drive with encrypted backups made daily both on and off-site. These data will be double entered into two separate MS Access databases. After double data entry has been completed data will be cleaned using EpiData version 3.1 software. Data

For peer review only

entry is supervised by the local study co-ordinator on a daily basis, with data collection and data entry progress being reviewed by the study co-ordinator and chief investigator at LSHTM on a weekly basis. Data protection and confidentiality is maintained through restricted access to the database system and cupboard where the paper documents are kept. Only authorised users will have access to the locked filing cabinet. The database will be password protected, with each data entry staff member having their own password. Data exports for further analysis will be anonymized.

**Data and Safety Monitoring Board**

The Data and Safety Monitoring Board (DSMB) for this trial includes independent experts in bioethics, biostatistics, epidemiology and ophthalmology appointed by the Trial Steering Committee and approved by the national regulatory authorities, before the start of the study. The DSMB meets at least twice each year and organises teleconferences as needed for progress reporting. The study protocol and modifications are subject to review and approval by ethics committees in Nepal and LSHTM, and by the DSMB. The DSMB will monitor any severe or unexpected events and oversees the data collected. The DSMB will be responsible for reviewing the results of the interim analysis and determining whether or not the trial should continue, with or without modifications.

**Monitoring for Harm**

Patients will be monitored at each visit for adverse events or reactions. We will follow standardised definitions for adverse events, adverse reactions, unexpected adverse reactions, serious adverse events or reactions, and suspected unexpected serious adverse reaction. These, along with the reporting scheme, are given in Appendix 2.

**Biological specimens**

The processing and analysis of biological specimens are detailed in Appendix 3.

**Sample Size Considerations**

The study is powered to test the hypothesis that CHX is non-inferior to NATA in terms of the primary outcome (BSCVA at 3 months) at a pre-specified non-inferiority margin (Delta) of 0.15 logMAR units. It is possible that CHX is non-inferior to NATA, given pilot RCT data that showed no statistically significant difference between CHX and NATA, with a Cochrane review finding a non-significant trend favouring CHX over NATA.[10,18,19] Despite this trend favouring CHX, we have chosen to carry out a non-inferiority trial rather than a superiority trial as this is a more clinically pragmatic approach. It would be counter-productive to conduct a superiority trial and find no statistically significant difference between the two treatments leading clinicians to potentially disregard CHX as a treatment, when in fact it may be ‘non-inferior” to NATA, and be the only available or most cost-effective treatment.



For peer review only - http://bmjopen.bmj.com/site/about/guidelines.xhtml

The choice of Delta: This clinically meaningful difference of 0.15 logMAR was chosen as a difference of 0.15 logMAR corresponds to approximately 1.5 lines on a Snellen chart; any difference greater than this is clinically significant, as a difference of less than 0.15 log MAR could potentially be accounted for by testing / re-testing error.[35] Furthermore, it was used in the MUTT1 trial, providing methodological consistency between studies.[6] In addition, previous studies have suggested treated ulcers improve at a mean of four Snellen lines from baseline.

Sample size was calculated for 90% power and adjusted final alpha of 0.0492, taking account of a single interim analysis using O'Brien-Fleming approach to maintain type-1 error rate of 5%. A sample size of 452 is required to detect a non-inferiority margin of 0.15-logMAR in BSCVA 3-months after enrolment between arms, assuming 0.5 SD for 3-month BSCVA and 15% dropout. However, given approximately 10% of infections are mixed and these will be excluded from the primary analysis, 500 patients will be recruited. This sample size provides 90% power to detect superiority in BSCVA at 3-months if there is ≥0.17 LogMAR units difference as a secondary analysis.

**Analysis Plan**

The analysis will be by intention to treat. All patient data will be analysed according to their randomisation allocation irrespective of whether or not the patient received or adhered to the allocated treatment. CONSORT guidelines for analysing/reporting non-inferiority RCTs will be followed. A flowchart showing cases assessed, recruited and followed-up by arm will be prepared.[38] Baseline characteristics will be summarised by arm. The SPIRIT checklist is given in Appendix 4.

***Primary Outcome Analysis – unadjusted analysis:*** The primary analysis will be conducted using all available data, missing data due to loss to follow up will be excluded. The primary analysis of the primary outcome (BSCVA at 3 month) will be by linear regression, with treatment arm and baseline BSCVA as pre-specified covariates. This controls for the baseline BSCVA. The treatment group is the primary predictor. Primary analysis will exclude mixed fungal and bacterial infection (isolated in the baseline sample).

We will use our alpha of 0.0492 to test the null hypotheses at 0.0492 significance. The null hypothesis for non-inferiority is that the mean BSCVA at three months for chlorhexidine is greater than or equal to 0.15 logMAR worse than the BSCVA when natamycin is used. Significance will be assessed using a two-tailed test at 0.0492 level for assessing non-inferiority. CHX will be non-inferior if the upper one-sided 95% confidence level for this regression coefficient (i.e. the effect of CHX controlling for baseline BSCVA) exceeds 0.15 logMAR.


For peer review only - http://bmjopen.bmj.com/site/about/guidelines.xhtml

***Primary Outcome Analysis – adjusted analysis:*** In the event that there is a baseline imbalance between the treatment groups in a baseline covariate due to chance, we will perform an adjusted (sensitivity) analysis (see below). This is particularly important if CHX has a better outcome than NATA, as the adjusted treatment effects may account for this observed imbalance whilst the unadjusted analyses may not. Sensitivity analyses will allow us to show that any observed positive treatment effect is not solely explained by imbalances at baseline in any of the covariates.

***Secondary Analyses of the Primary Outcome***

Per-protocol analysis

Repeat analysis of the primary outcome will be done as per the protocol, based on what the participants actually took. The per-protocol population will include all the individuals included in the primary intention-to-treat (ITT) analysis, excluding individuals who showed poor compliance with the medications (defined as taking less than 50% through self-reporting or bottle weighing, whichever is the lower); individuals where there have been major protocol deviations; and non-fungal or mixed corneal infections (should these patients happened to have been randomised). All analyses that are performed as ITT will be repeated as per-protocol and labelled as such.

Mixed infections

Secondary analysis of the primary outcome (by intention to treat) will include mixed infections and will be carried out in the same way as in primary analysis of the primary outcome above.

Sensitivity analyses

For the primary analysis those individuals with missing outcome data (i.e. lost to follow up) will be excluded. Sensitivity analyses will be performed by imputing a range of scenarios to demonstrate a range of potential results, where there is missing outcome data. In the case of substantial missing data in the trial, the primary analysis will be carried out as previously stated excluding missing observations. This however assumes data are missing completely at random. As a sensitivity analysis, we will then apply a multiple imputation approach to the missing data, if we consider the data is randomly missing conditional on other observed covariates.

In the case that there is a systematic (i.e. non-random) reason for a difference in the follow-up rates between the two groups, we will explore models in which these missing outcome data are assumed to be non-random i.e. dependent on the outcome being regressed, BSCVA or treatment group.



For peer review only - http://bmjopen.bmj.com/site/about/guidelines.xhtml

We will also perform sensitivity analyses on patients whose vision is CF or worse, those patients who have undergone a TPK or those with corneal perforation, to see if there is any change in the effect size or conclusions drawn.

Analysis of Other Potential Determinants for Success

Logistic regression random effects models will be used to analyse potential factors that may be associated with a poor primary outcome, BSCVA at 3 months, defined as >1.0 logMAR. Individual baseline characteristics will be used separately as an exposure variable with BSCVA at 3 months as the outcome, with the model adjusted for trial arm. A multivariate model will be built using parameters with a p-value of < 0.2 in the log likelihood ratio test. Variables will be removed one by one, by omitting the variable with the largest p-value each time, until all predictors in the model have a p<0.05.

***Secondary Outcome Analysis:*** The secondary outcomes outlined above and detailed in Table 4 will be analysed by arm. Additional adjustment for factors imbalanced between arms at baseline will be introduced as appropriate. Continuous outcomes will be analysed using linear regression. Binary and ordinal outcomes will analysed using logistic regression. Details of these are given in Table 4.

For peer review only



For peer review only - http://bmjopen.bmj.com/site/about/guidelines.xhtml

**Table 4: Secondary Outcome Measures that will be investigated as part of the trial, together with analysis details**

| Secondary outcome measure | Details |
|---|---|
| Three-week BSCVA | We will analyse the secondary outcome of three-week BSCVA in logMAR in the same manner as the primary analysis of the primary outcome described above. The three-week BSCVA will include values taken between 18 days and 5 weeks, with the value closest to 3 weeks used. |
| Presenting VA by Peek | We will analyse the presenting VA by Peek Acuity with and without pinhole at three months as a secondary outcome. This will be of interest if we are unable to get reliable BSCVA measurements at 3 months (i.e. if patients fail to attend and we need to attend their houses for visual acuity testing). This will be performed in the same way as the primary analysis of the primary outcome. We will also perform a sensitivity analysis including those lost to follow-up, by using the most recent observation of this variable. |
| Scar/infiltrate size at 1 week, 3 weeks and 3 months by slit lamp examination. | The geometric mean of the two principle axes in mm of the scar or infiltrate at 1 week, 3 weeks and 3 months will be used as a secondary outcome variable. The slit-lamp scar size will be compared at each of these time points between treatment arm in the same manner as described above. This will be by linear regression, with treatment arm and baseline infiltrate / scar size as pre-specified covariates. This controls for the baseline infiltrate / scar size. |
| Time to full epithelial healing (slit lamp examination by ophthalmic clinician) | Time of re-epithelialisation will be defined as the midpoint between the last review where an epithelial defect (ED) was present and the subsequent review where there was no ED. An area of fluorescein staining of less than 0.5mm will be considered as a resolved ED due to the difficulty in differentiating a smaller defect from a small amount of fluorescein pooling observed in a healed defect.<br><br>Analysis of time to healing will use Cox proportional hazards regression with treatment group as the primary predictor and with predictors of baseline ED size (using the geometric mean in mm as outlined above). Survival curves will be plotted using Kaplan-Meier analysis for both treatment arms up to the final visit at three months. The proportional hazards assumption will be checked by stratifying on quartiles of the baseline ED size and if the assumption does not hold, the stratified results will be the ones reported. Additionally, treatment failure (defined as persisting epithelial defect greater than 0.5mm at the three-month review) will be compared between treatment groups using Fisher's exact test. |
| Rate of healing | We will assess how quickly the area of ulceration reduces over time. The rate will be calculated between the 1 week, 3 week and 3-month review by taking the difference in ED size between the two time points, in mm, and diving by the number of days to give a rate of mm/day. Analysis will be performed using Cox regression. |
| Microbiological cure | Patients who have a persisting corneal ulcer (as defined by the presence of an ED) at day 7 will undergo a repeat corneal scrape and microbiological investigations. Microbiological cure at 7 days will be defined as the absence of any micro-organisms as no significant growth on culture. The number of patients with microbiological cure at day 7 will be compared between the two treatment arms using logistic regression with treatment group and organism (Aspergillus spp., Fusarium spp., or other) as covariates. |
| Ulcer depth at 1 week and 3 weeks (slit lamp examination by ophthalmic clinician). | The depth of ulcer in terms of percentage of healthy cornea will be compared at 1 week and 3 weeks between treatment arms, adjusting for baseline depth in the same manner with analysis performed by linear regression |
| Hypopyon height at 1 and 3 weeks, (slit lamp examination by ophthalmic clinician) | The hypopyon height in mm will be compared at 1 week and 3 weeks between treatment arms, adjusting for baseline hypopyon height in the same manner with analysis performed in the same way (linear regression) |
| Perforation and/or TPK and/or conjunctival advancement by three months (slit lamp examination by ophthalmic clinician) | The number of patients who undergo perforation and/or require TPK and/or have undergone conjunctival advancement by three months will be reported using confidence intervals and descriptive statistics. The study is not powered to detect a difference in perforation rate or TPK between treatment groups; not reporting a significant difference may be wrongly interpreted as there being no difference between groups. We will therefore perform an exploratory analysis to compare TPK |



For peer review only - http://bmjopen.bmj.com/site/about/guidelines.xhtml

| | |
|---|---|
| | or perforation rates between treatment groups. This will be by logistic regression to compute an odds ratio by arm. |
| Loss of Eye | The number of patients who have their eye surgically removed (evisceration or enucleation) during the 3 month follow-up period will be reported using confidence intervals and descriptive statistics in the same way as for TPK / perforation rate above, along with exploratory analysis using logistic regression to find risk factors for eye loss and odds ratio for this by arm. |
| Ocular adverse effects, slit lamp examination by ophthalmic clinician. | The proportion of patients with one or more adverse events will be compared using Fisher's Exact test. Additional analysis to compare the rate of adverse events during the three month follow-up will be by Poisson regression as this can take into account multiple instances within one participant. |
| Quality of life assessed using: EQ-5D, WHO/PBD-VF20, WHOQOL-BREF | QoL can be assessed quantitatively using different tools depending on what is of interest. For example, disease-related QoL can be assessed (e.g. vision related QoL, VRQoL) or more general health-related issues irrespective of the disease can be investigated (health-related QoL, HRQoL).[39]<br><br>We will use the WHO/PBD-VF20 (World Health Organisation/ Prevention of Blindness and Deafness—Visual Functioning 20 item questionnaire) VRQoL tool. This tool measures the impact of visual impairment in the person's life including mental wellbeing, dependency and social functioning. These have been used in a number of other visual related studies to show a difference in QoL. [31,32]<br><br>For HRQoL, we will use the EQ-5D questionnaire, EQ-Visual Analogue Scale and the WHOQOL-BREF. The EQ-5D is a standardised tool to measure health outcomes.[33] The WHOQOL-BREF has good applicability in (LMIC as it was developed simultaneously from concept across 18 countries in Africa, Asia and Latin America. It measures 4 domains of health: Physical Health, Psychological Health, Social Relationships, and Environment. Details of this scoring are given in Table 2.[34]<br><br>Analysis will be by comparing the scores obtained for each QoL assessment for the two treatment arms to estimate the effect of CHX and NATA on patients' quality of life. This will be similar to that performed by Habtamu et al. [39] Comparisons between the two medication groups will be adjusted for the matching variables: age and sex. The VRQoL analysis was also adjusted for socio-economic status and the HRQoL analysis adjusted for both socio-economic status and presence of health problems during the previous four weeks, as these factors may confound the association between fungal keratitis and QoL. Logistic, linear and ordinal logistic regression methods will be used for binary, continuous and ordered categorical outcome variable analysis, respectively. Linear regression models and the t-test were employed to compare significant differences in QoL scores and to generate mean and mean differences between the two treatment arms in each QoL subscale and domain, respectively. |
| Cost effectiveness analysis, using EQ-5D data from 3 months and direct cost data | Direct cost data will be collected at the three-month follow-up. Economic costs to the patient can also be calculated from the EQ-5D questionnaire, which will be asked at baseline and at the three-month follow-up. Mean direct costs incurred by patients will be compared between interventional arms using the t-test for significance. The difference from the baseline EQ-5D and the three-month EQ-5D mean scores will also be compared in a similar fashion. |
| Drug adherence | The rate of drug adherence will be compared between the two treatment groups using descriptive statistics. |

For peer review only

***Interim Analysis:*** An interim analysis will be conducted for the DSMB by an independent statistician after 1/3 of patients recruited have completed follow-up.

**Patient and public involvement**

Mixed methods descriptive cross-sectional studies with semi-structured interviews and focus group discussions with patients and eye health providers were carried out in Sagarmatha zone, Eastern Nepal at various points in 2018.[40] These conversations highlighted the delayed presentation often seen with fungal keratitis combined with the often prohibitively high costs of treatment. Furthermore, treatment is not always felt to be effective. Eye health workers were keen to receive further training and highlighted the need for greater government support in the provision of eye care services in the community.

**ETHICS AND DISSEMINATION**

Ethics committee and regulatory review and approval has been obtained from the Nepal Health Research Council (NHRC) Ethics Committee, Kathmandu, Nepal; the Department of Drug Administration (DDA), Kathmandu, Nepal; and the London School of Hygiene and Tropical Medicine Ethics Committee, UK. The trial is registered with the ISRCTN clinical trials registry (ISRCTN14332621). Protocol modifications are submitted to the relevant parties for review and/or approval. Table 5 summarises the study protocol and trial registration information. At the end of the study period, patients who still require treatment or follow-up will continue to be treated at SCEH as per routine clinical practice. The trial Sponsor is the London School of Hygiene and Tropical Medicine. The results of this trial will be presented at local and international meetings and submitted to peer-reviewed journals for publication.

For peer review only

**Table 5: Registration Data and Protocol Summary**

| Data category | Information |
|---|---|
| Primary registry and trial identifying number | ISRCTN Registry; ISRCTN14332621 |
| Date of registration in primary registry | 15 May 2019 |
| Secondary identifying numbers | |
| Source(s) of monetary or material support | Wellcome Trust |
| Primary sponsor | London School of Hygiene and Tropical Medicine (LSHTM) |
| Secondary sponsor(s) | |
| Contact for queries | Jeremy Hoffman FRCOphth (Jeremy.hoffman@lshtm.ac.uk) |
| Title | Chlorhexidine 0.2% vs Natamycin 5% for the treatment of fungal corneal infections |
| Countries of recruitment | Nepal |
| Health condition(s) or problem(s) studied | Fungal keratitis |
| Intervention(s) | Participants will be randomised to either topical chlorhexidine 0.2% or topical natamycin 5% |
| Key eligibility criteria | 1. Acute MK characterised by:<br>• Corneal epithelial ulceration >1mm diameter<br>• Corneal stromal infiltrate<br>• Acute inflammation: e.g. conjunctival injection, anterior chamber inflammatory cells, hypopyon.<br>2. Filamentous fungal hyphae visualised on smear microscopy and/or *in vivo* confocal microscopy.<br>3. Agree to be randomised to either treatment arm and able to give informed consent<br>4. Agree to be followed up at 2 days, 1 week, 2 weeks, 3 weeks, 2 months and 3 months<br>5. Adults (18 years and older) |
| Study type | Randomised controlled trial |
| Date of first enrolment | 1 June 2019 |
| Target sample size | 500 |
| Recruitment status | Recruiting |
| Primary outcome(s) | Best Spectacle Corrected Visual Acuity (BSCVA) at 3 months by a trial certified optometrist |
| Key secondary outcomes | 1. Time to full epithelial healing (slit lamp examination by ophthalmic clinician).<br>2. Pin-hole visual acuity in logMAR at 3 months, trial-certified optometrist<br>3. Scar/infiltrate size at 1 week, 3 weeks and 3 months (slit lamp examination by ophthalmic clinician).<br>4. Ulcer depth at 1 week and 3 weeks (slit lamp examination by ophthalmic clinician).<br>5. Hypopyon height at 1 and 3 weeks, (slit lamp examination by ophthalmic clinician).<br>6. Perforation and/or TPK by three months (slit lamp examination by ophthalmic clinician).<br>7. Positive culture rate at 1 week<br>8. Ocular adverse effects at each follow up visit (Day 2, Day 7, Day 14, 3 weeks, 2 months, 3 months), slit lamp examination by ophthalmic clinician.<br>9. Quality of life (QoL) assessed using: EQ-5D, WHO/PBD-VF20, WHOQOL-BREF (comparison between baseline and QoL measures at 3 months)<br>10. Cost-effectiveness analysis, using EQ-5D data from 3 months and direct cost data.<br>11. Drug adherence at each follow up visit (Day 2, Day 7, Day 14, 3 weeks, 2 months, 3 months) whilst the patient is using study medications. |

For peer review only



For peer review only - http://bmjopen.bmj.com/site/about/guidelines.xhtml

**ACKNOWLEDGEMENTS**

The authors would like to thank the Eastern Region Eye Care Programme (EREC-P), Nepal Netra Jyoti Sangh (NNJS) and the Nepal Health Research Council (NHRC) for helping with study co-ordination and implementation. The authors would like to thank the staff and management board at Sagarmatha Choudhary Eye Hospital (SCEH) for their continued support, co-ordination and implementation of the study. The authors are grateful to the guidance of the Data Safety Monitoring Board including Reeta Gurung (chair), Salma KC Rai, Sabina Shrestha and Meenu Chaudhary.

**COLLABORATORS**

SCEH:

Abhishek Roshan (Hospital Manager); Sanjay Kumar Singh (Medical Superintendent); Reena Yadav (Primary Investigator); Sandip Das Sanyam (Study Co-Ordinator); Pankaj Chaudhary (Microbiologist); Rabi Shankar Sah, Kamlesh Yadav (Investigators); Ram Narayan Bhandari, Aasha Chaudhary, Sharban Mandal (Eye Health Workers); Raja Ram Mahato (Randomisation Administrator and Logistics); Lalita Rajbanshi (Laboratory Assistant); Ramesh Sah, Arvind Ray, Sachindra Kamti (Optometrists); Avinash Chaudhary (Ophthalmic Assistant); Padma Narayan Chaudhary (Hospital Chairman); Suresh Singh, Ravi Pant, Rakesh Singh (Hospital Management); Ram Kumar Jha (Ophthalmic Assistant, Rajbiraj ECC)

NNJS:

Sailesh Kumar Mishra (Executive Director); Sabita KC (Board Secretary); Ranjan Shah (Programme Associate); Jaganath Dhital (Assistant)

EREC-P:

Sanjay Kumar Singh (Programme Director)

JEH:

Hemchandra Jha (Medical Superintendent); Mahesh Yadav (Investigator); Rudal Prasad Sah (Ophthalmic Assistant)

LSHTM:

Jeremy Hoffman (Primary Investigator); Matthew Burton (Chief Investigator); Astrid Leck (Microbiologist); David Macleod, Helen Weiss (Statisticians); Victor Hu (Investigator); Sarah O'Regan (Administrator)

For peer review only

**FUNDING STATEMENT**

This research was funded through a Senior Research Fellowship to MJB from the Wellcome Trust (207472/Z/17/Z). The funders had no role in study design, data collection and analysis, decision to publish, or preparation of the manuscript.

**COMPETING INTERESTS STATEMENT**

All authors declare there are no conflict of interests.

**DATA STATEMENT**

The datasets generated during and/or analysed during the current study will be available upon request from Prof Matthew Burton (matthew.burton@lshtm.ac.uk). The full data set will be available with all patient identifiable details removed. Data will be available after formal reporting of the study findings in a peer-reviewed scientific publication. Datasets will only be available to bona fide scientific investigators. Requests should be made to the Chief Investigator in writing detailing the scientific investigators background and intended use for the data. Consideration will be given to all proposed analyses, with likely envisaged uses including investigators planning on conducting meta-analyses for example. Patient Information Sheets and consent forms specifically referenced making anonymised data available and this has been approved by the relevant ethic committees.

**AUTHOR CONTRIBUTIONS**

Searched the literature: JJH, MJB

Drafted initial protocol: JJH

Contributed to protocol development and revision: JJH, RY, SD, PC, AR, SKS, SA, EM, DM, HAW, AL, VHH, MJB

Drafted this manuscript: JJH

Critically revised this manuscript: JJH, RY, SD, PC, AR, SKS, SA, EM, DM, HAW, AL, VHH, MJB

Conceptualization: MJB

Funding acquisition: MJB

For peer review only

**APPENDICES:**

Appendix 1 - Informed consent material

Appendix 2 – Monitoring for Harm

Appendix 3 - Biological specimens

Appendix 4 – SPIRIT Checklist

For peer review only



For peer review only - http://bmjopen.bmj.com/site/about/guidelines.xhtml

**LEGENDS FOR FIGURES**

**Figure 1: Fungal keratitis and corneal scarring.** (A) Active fungal keratitis with signs of acute inflammation and corneal ulceration. Photograph taken at presentation to SCEH. (B) Corneal Scar, the blinding sequela of a resolved episode of fungal keratitis. Photograph taken at two months following presentation (same patient as (A)).

**Figure 2: Progressive fungal keratitis.** (A) Early filamentous fungal keratitis; started immediately on intensive topical antifungal treatment (Natamycin 5%). (B) The same case one week later, unresponsive to intense Natamycin 5% treatment, with progression of the infection.

**Figure 3: Overview of the clinical trial.** Microbial keratitis is defined as presence of corneal epithelial ulceration (>1mm in diameter), corneal stromal infiltrate and signs of acute inflammation (e.g. conjunctival injection, anterior chamber inflammatory cells, hypopyon). Fungal elements to be detected by smear microscopy and/or confocal microscopy. Those eligible will be randomised 1:1 to CHX or NATA (n = 500). BSCVA: Best Spectacle Corrected Visual Acuity; TPK: Therapeutic Penetrating Keratoplasty.

For peer review only


For peer review only - http://bmjopen.bmj.com/site/about/guidelines.xhtml

## REFERENCES


1 Burton MJ, Pithuwa J, Okello E, *et al.* Microbial Keratitis in East Africa: Why are the Outcomes so Poor? *Ophthalmic Epidemiol* 2011;**18**:158–63. doi:10.3109/09286586.2011.595041

2 Thomas PA, Leck AK, Myatt M. Characteristic clinical features as an aid to the diagnosis of suppurative keratitis caused by filamentous fungi. *British Journal of Ophthalmology* 2005;**89**:1554–8. doi:10.1136/bjo.2005.076315

3 Leck AK, Thomas PA, Hagan M, *et al.* Aetiology of suppurative corneal ulcers in Ghana and south India, and epidemiology of fungal keratitis. *British Journal of Ophthalmology* 2002;**86**:1211–5. doi:10.1136/bjo.86.11.1211

4 Bongomin F, Gago S, Oladele RO, *et al.* Global and Multi-National Prevalence of Fungal Diseases-Estimate Precision. *J Fungi (Basel)* 2017;**3**:57. doi:10.3390/jof3040057

5 Ali Shah SI, Shah SA, Rai P, *et al.* Visual outcome in patients of keratomycosis, at a tertiary care centre in Larkana, Pakistan. *J Pak Med Assoc* 2017;**67**:1035–8.

6 Prajna NV, Krishnan T, Mascarenhas J, *et al.* The mycotic ulcer treatment trial: a randomized trial comparing natamycin vs voriconazole. *JAMA Ophthalmol* 2013;**131**:422–9.

7 Prajna NV, Krishnan T, Rajaraman R, *et al.* Effect of Oral Voriconazole on Fungal Keratitis in the Mycotic Ulcer Treatment Trial II (MUTT II): A Randomized Clinical Trial. *JAMA Ophthalmol* 2016;**134**:1365–72. doi:10.1001/jamaophthalmol.2016.4096

8 Galarreta DJ, Tuft SJ, Ramsay A, *et al.* Fungal keratitis in London: microbiological and clinical evaluation. *Cornea* 2007;**26**:1082–6. doi:10.1097/ICO.0b013e318142bff3

9 Ong HS, Fung SSM, Macleod D, *et al.* Altered Patterns of Fungal Keratitis at a London Ophthalmic Referral Hospital: An Eight-Year Retrospective Observational Study. *Am J Ophthalmol* 2016;**168**:227–36. doi:10.1016/j.ajo.2016.05.021

10 FlorCruz NV, Evans JR. Medical interventions for fungal keratitis. *Cochrane Database Syst Rev* 2015;**4**:CD004241. doi:10.1002/14651858.CD004241.pub4

11 Schein OD. Evidence-Based Treatment of Fungal Keratitis. *JAMA Ophthalmol* 2016;**134**:1372–3. doi:10.1001/jamaophthalmol.2016.4167

12 Arora R, Gupta D, Goyal J, *et al.* Voriconazole versus natamycin as primary treatment in fungal corneal ulcers. *Clin Experiment Ophthalmol* 2011;**39**:434–40. doi:10.1111/j.1442-9071.2010.02473.x

13 Prajna NV, Mascarenhas J, Krishnan T, *et al.* Comparison of natamycin and voriconazole for the treatment of fungal keratitis. *Arch Ophthalmol* 2010;**128**:672–8. doi:10.1001/archophthalmol.2010.102

14 Sharma S, Das S, Virdi A, *et al.* Re-appraisal of topical 1% voriconazole and 5% natamycin in the treatment of fungal keratitis in a randomised trial. *Br J Ophthalmol* 2015;**99**:1190–5. doi:10.1136/bjophthalmol-2014-306485

15 McDonnell G, Russell AD. Antiseptics and disinfectants: activity, action, and resistance. *Clinical Microbiology Reviews* 1999;**12**:147–79.


For peer review only

16 Shariff JA, Lee KC, Leyton A, *et al.* Neonatal mortality and topical application of chlorhexidine on umbilical cord stump: a meta-analysis of randomized control trials. *Public Health* 2016;**139**:27–35. doi:10.1016/j.puhe.2016.05.006

17 Zhou J, Hu B, Liu Y, *et al.* The efficacy of intra-alveolar 0.2% chlorhexidine gel on alveolar osteitis: a meta-analysis. *Oral Dis* 2017;**23**:598–608. doi:10.1111/odi.12553

18 Rahman MR, Johnson GJ, Husain R, *et al.* Randomised trial of 0.2% chlorhexidine gluconate and 2.5% natamycin for fungal keratitis in Bangladesh. *British Journal of Ophthalmology* 1998;**82**:919–25. doi:10.1136/bjo.82.8.919

19 Rahman MR, Minassian DC, Srinivasan M, *et al.* Trial of chlorhexidine gluconate for fungal corneal ulcers. *Ophthalmic Epidemiol* 1997;**4**:141–9. doi:10.3109/09286589709115721

20 Dart JKG, Saw VPJ, Kilvington S. Acanthamoeba keratitis: diagnosis and treatment update 2009. *Am J Ophthalmol* 2009;**148**:487–499.e2. doi:10.1016/j.ajo.2009.06.009

21 Kosrirukvongs P, Wanachiwanawin D, Visvesvara GS. Treatment of acanthamoeba keratitis with chlorhexidine. *Ophthalmology* 1999;**106**:798–802. doi:10.1016/S0161-6420(99)90169-0

22 Seal D, Hay J, Kirkness C, *et al.* Successful medical therapy of Acanthamoeba keratitis with topical chlorhexidine and propamidine. *Eye* 1996;**10 ( Pt 4)**:413–21. doi:10.1038/eye.1996.92

23 Martin MJ, Rahman MR, Johnson GJ, *et al.* Mycotic keratitis: susceptibility to antiseptic agents. *Int Ophthalmol* 1995;**19**:299–302.

24 Geffen N, Norman G, Kheradiya NS, *et al.* Chlorhexidine gluconate 0.02% as adjunct to primary treatment for corneal bacterial ulcers. *Isr Med Assoc J* 2009;**11**:664–8.

25 Upadhyay MP, Karmacharya PC, Koirala S, *et al.* The Bhaktapur eye study: ocular trauma and antibiotic prophylaxis for the prevention of corneal ulceration in Nepal. *British Journal of Ophthalmology* 2001;**85**:388–92.

26 Habtamu E, Bastawrous A, Bolster NM, *et al.* Development and Validation of a Smartphone-based Contrast Sensitivity Test. *Transl Vis Sci Technol* 2019;**8**:13. doi:10.1167/tvst.8.5.13

27 Chidambaram JD, Prajna NV, Larke N, *et al.* In vivo confocal microscopy appearance of Fusarium and Aspergillus species in fungal keratitis. *Br J Ophthalmol* 2017;**101**:1119–23. doi:10.1136/bjophthalmol-2016-309656

28 Chidambaram JD, Prajna NV, Larke NL, *et al.* Prospective Study of the Diagnostic Accuracy of the In Vivo Laser Scanning Confocal Microscope for Severe Microbial Keratitis. *Ophthalmology* 2016;**123**:2285–93. doi:10.1016/j.ophtha.2016.07.009

29 Somannavar S, Ganesan A, Deepa M, *et al.* Random capillary blood glucose cut points for diabetes and pre-diabetes derived from community-based opportunistic screening in India. *Diabetes Care* 2009;**32**:641–3. doi:10.2337/dc08-0403

30 World Health Organization. Prevention of Blindness & Deafness. Consultation on Development of Standards for Characterization of Vision Loss and Visual Functioning. In: *WHO / PBL*. Geneva: 2003.

31 Polack S, Kuper H, Mathenge W, *et al.* Cataract visual impairment and quality of life in a Kenyan population. *British Journal of Ophthalmology* 2007;**91**:927–32. doi:10.1136/bjo.2006.110973


For peer review only

32 Polack S, Kuper H, Wadud Z, *et al.* Quality of life and visual impairment from cataract in Satkhira district, Bangladesh. *Br J Ophthalmol* 2008;**92**:1026–30. doi:10.1136/bjo.2007.134791

33 EuroQol Research Foundation. EQ-5D-3L User Guide. 2018. https://euroqol.org/publications/user-guides

34 World Health Organization. *WHOQOL-BREF: introduction, administration, scoring and generic version of the assessment: field trial version, December 1996*. Geneva: : World Health Organization 1996.

35 Bastawrous A, Rono HK, Livingstone IAT, *et al.* Development and Validation of a Smartphone-Based Visual Acuity Test (Peek Acuity) for Clinical Practice and Community-Based Fieldwork. *JAMA Ophthalmol* 2015;**133**:930–8. doi:10.1001/jamaophthalmol.2015.1468

36 Barron BA, Gee L, Hauck WW, *et al.* Herpetic Eye Disease Study. A controlled trial of oral acyclovir for herpes simplex stromal keratitis. *Ophthalmology* 1994;**101**:1871–82.

37 Wilhelmus KR, Beck RW, Moke PS, *et al.* Acyclovir for the prevention of recurrent herpes simplex virus eye disease. Herpetic Eye Disease Study Group. *N Engl J Med* 1998;**339**:300–6. doi:10.1056/NEJM199807303390503

38 Schulz KF, Altman DG, Moher D, *et al.* CONSORT 2010 statement: updated guidelines for reporting parallel group randomised trials. BMJ. 2010;**340**:c332–2. doi:10.1136/bmj.c332

39 Habtamu E, Wondie T, Aweke S, *et al.* The Impact of Trachomatous Trichiasis on Quality of Life: A Case Control Study. *PLoS Negl Trop Dis* 2015;**9**:e0004254. doi:10.1371/journal.pntd.0004254

40 Burn H, Puri L, Roshan A, *et al.* Primary Eye Care in Eastern Nepal. *Ophthalmic Epidemiol* 2019;**22**:1–12. doi:10.1080/09286586.2019.1702217

For peer review only

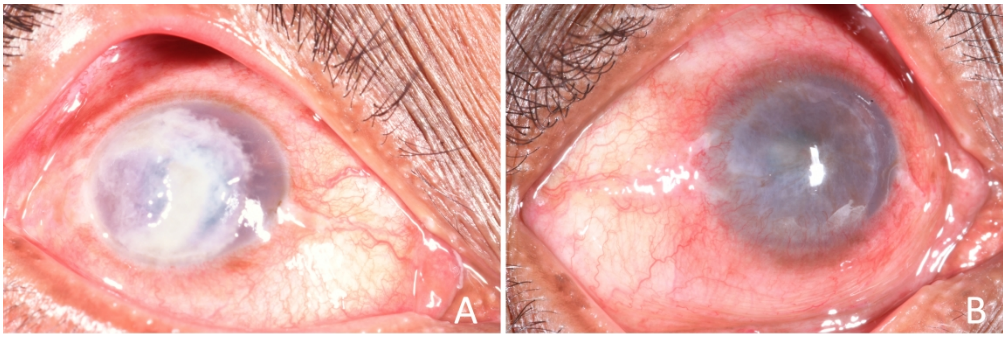


Figure 1: Fungal keratitis and corneal scarring. (A) Active fungal keratitis with signs of acute inflammation and corneal ulceration. Photograph taken at presentation to SCEH. (B) Corneal Scar, the blinding sequela of a resolved episode of microbial keratitis. Photograph taken at two months following presentation (same patient as (A)).

325x107mm (300 x 300 DPI)

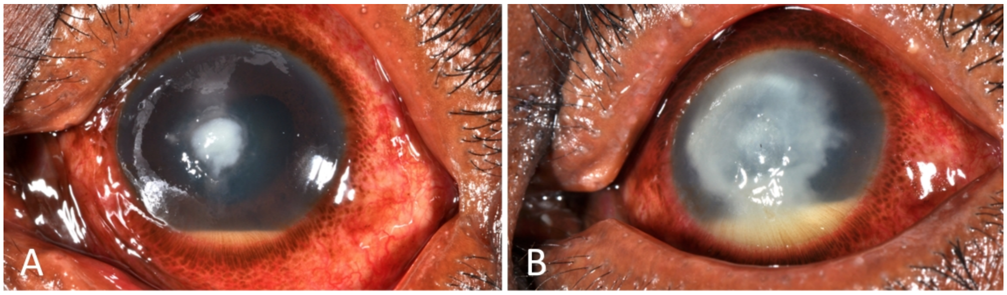


Figure 2: Progressive fungal keratitis. (A) Early filamentous fungal keratitis; started immediately on intensive topical antifungal treatment (Natamycin 5%). (B) The same case one week later, unresponsive to intense Natamycin 5% treatment, with progression of the infection.

325x93mm (300 x 300 DPI)

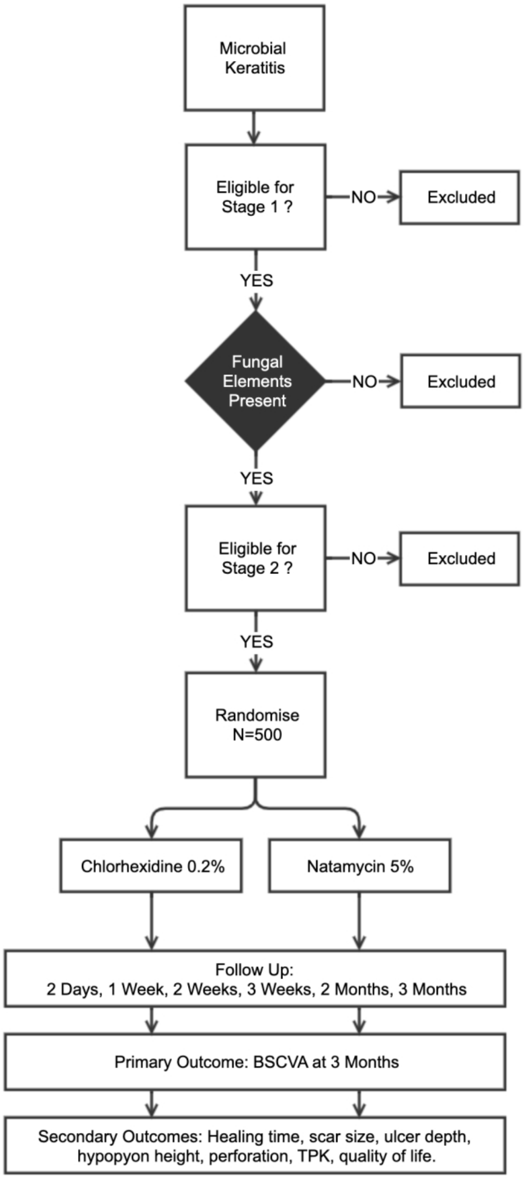


Figure 3: Overview of the clinical trial. Microbial keratitis is defined as presence of corneal epithelial ulceration (>1mm in diameter), corneal stromal infiltrate and signs ofacute inflammation (e.g. conjunctival injection, anterior chamberinflammatory cells, hypopyon). Fungal elements to be detected by smear microscopy and/or confocal microscopy. Those eligible will be randomised 1:1 to CHX or NATA (n = 500). BSCVA: Best Spectacle Corrected Visual Acuity; TPK: Therapeutic Penetrating Keratoplasty.

87x182mm (300 x 300 DPI)

# Appendix 1: Informed Consent Material

## Study Title: Randomised controlled trial of topical chlorhexidine 0.2% versus topical natamycin 5% for fungal keratitis in Nepal

*Participant Information Sheet and Consent Form - No 1:*
*For baseline assessment and investigations*

**Introduction**
You are being invited to take part in a medical research study. Before you decide it is important for you to understand why the research is being done and what it will involve. Please take time to read or listen to the following information carefully and to talk to others about the study, if you wish. Ask us if there is anything that is not clear or if you would like more information. Do not sign the consent form unless you are satisfied with the answers to your questions and decide that you want to be part of this study. Take time to decide whether or not you wish to take part.

**Why have I been invited?**
You have been invited to take part because you have a corneal infection. To guide the treatment of this it is helpful to do tests to find out the cause, to guide the choice of eye drop treatment. We are asking for your permission to carry out some tests that are listed below to find out what is causing the infection and to decide if you may be suitable for a trial comparing treatments for fungal eye infections.

**What is this study about?**
Infection of the clear part of the front of the eye (the cornea) is a corneal ulcer. It is an important cause of blindness in Nepal. A scratch in the cornea allows infection to enter and an ulcer to begin. These infections can be very serious with some people losing the sight in the affected eye.

Different types of infectious organisms can cause corneal ulcers. These include bacteria and fungi which need to be treated with different types of eye drop medicines. To find out what type of infection is present it is necessary to collect a small sample from the ulcer and perform various laboratory tests. The test results guide the treatment. In Nepal about half of the infections are caused by fungus.

Fungal infections can be difficult to diagnose. No single test can reliably detect it. It may need several different tests to find it. Currently, in many countries where fungal ulcers are common laboratory services are limited or not available. Tests are expensive and may not detect all of the infections. There is a need to understand which of the current test options give the most useful information.

The first purpose of this study is to carefully evaluate your eye infection using several different diagnostic tests to try to find out what type of infection you have, and to learn which tests are most useful in this process.

There is a need for alternative, affordable and more easily available eye drop treatments for fungal infections. We are also conducting a clinical trial to compare two eye drop treatments for fungal infection, to try to find out which is better.

Therefore, the second purpose of this initial Assessment and Investigation study is to identify people who have a fungal corneal infection who may be eligible and willing to enrol in the clinical trial. If we find out that you do have a fungal corneal infection, we will then ask if you would be interested in hearing about the clinical trial. If you are interested, then at that stage we will explain about the treatment trial in detail, provide you with a second information sheet and go throw a second consent process.

**Do I have to take part?**
No. Your involvement is entirely voluntary. If you agree to take part, we will then ask you to sign a consent form. If you decide to join and change your mind, you are free to withdraw at any time without giving a reason. This will not affect the standard of care you receive.
Why have I been invited?
You have been invited to take part because you have a corneal infection. To guide the treatment of this it is helpful to do tests to find out the cause, to guide the choice of eye drop treatment. We are asking for your permission to carry out some tests that are listed below to find out what is causing the infection and to decide if you may be suitable for a trial comparing treatments for fungal eye infections.

For peer review only

**What will happen to me if I take part?**
If you agree to be part of this study, the following will happen:

- We will ask you a series of questions. This will include basic demographic information, the history of your current eye problem, and treatment you have had before arriving at the hospital.

- We will then carefully examine both eyes using a special microscope.

- Your eyes will be photographed with a camera. This is additional to standard care, and will help us to monitor the infection and the response to treatment.

- We will use a special microscope to look at the cornea to see if we can find a fungal infection. This involves putting anaesthetic drops on the eye. A soft plastic device then gently touches the eye so to see if you have a fungal infection. This is additional to standard care, and will help us to find out the type of infection you have more rapidly so that we can offer you the most appropriate treatment.

- We will collect samples form the corneal ulcer to test in the laboratory to try to identify what is causing the infection. Anaesthetic eye drops will be used numb the eye so you will not feel anything while we collect the sample by brushing the surface.

-

- We will check your blood sugar level for diabetes. This is done using a finger-prick blood sample. If this is raised, we will refer you to a separate group of doctors to help you with this.

- You will be offered a test for HIV infection. This will be done through the hospitals counselling and testing services. If you choose to accept this, then you will be separately counselled about the test and the implications of the results. This would involve the collection of a blood test sample from your arm. If the HIV test is positive, then you will be referred to the appropriate team for ongoing care. The result of this test will be shared with the us (cornea infection study team), with your consent, as it is potentially relevant to the treatment of your cornea infection.

- We will collect a sample of the cells from the inside of your cheek by gently rubbing a swab for a few seconds. This is additional to the standard of care. The purpose is to try to understand how the body fights the infection and why some people develop this eye problem and others do not.

- You will be asked to complete three short questionnaires to assess your quality of life and vision function. This is additional to standard care, and will help us to better understand the impact corneal ulcers can have on people's lives.

If we find evidence of fungus infecting your eye, then we will discuss the details of the trial with you further. At that point we will give you a second information sheet to read (or read to you), and if you meet all the eligibility criteria for the trial, to then go through a second consent process with you.

If you do not have a fungal infection, for example if the infection is caused by bacteria, then your will receive standard ongoing treatment for this by the same team at the hospital.

You may be withdrawn from the study without your consent if the researchers believe it is in your best interest or if you fail to follow study procedures.

**What are the side effects or risks of taking part?**

- The anaesthetic eye drops usually sting for a few seconds and then your eye should feel more comfortable. There is a very low risk of allergic reaction from the anaesthetic eye drops.
- The procedures including the history taking, examination, photography, in vivo confocal microscopy and sample collection are part of the standard of care for assessing a corneal infection. The risk associated with these are very low and no different from routine care. To minimise discomfort, topical anaesthetic will be given before examinations and sample collection.
- You will experience a little pain when a blood sample is collected. The people collecting the blood sample will ensure that this is done with as little discomfort as possible.
- The questions, tests and examinations will add about 15 minutes to your hospital visit above what we would usually expect in assessing a patient with this problem.

**What are the benefits of taking part?**

- The study will involve tests for the type of infection. This helps the doctor looking after you to choose the best type of treatment for your eyes
- The costs for the clinical assessment and the tests will be paid for by the study.
- By participating in this study, you will be helping to further research into this field so more informed decisions can be made when treating people with corneal infection.

For peer review only

**What will happen to the clinical records, photographs and test results?**
Your records will remain strictly confidential at all times. The information will be held in a secure office at your treating hospital. Only the people organizing or supervising the trial and regulatory authority auditors will have access to it. These include officials delegated by the Sponsor (London School of Hygiene and Tropical Medicine), the local National Ethics Committee, The local National Drug Regulatory Authority and trial Data Safety Monitoring Body (DSMB).

Your name will not be passed to anyone else outside the research team, unless we have your direct instruction to do so, for example to make a medical referral.

The photographs of the eye and the result of the laboratory test for infection will be shared with computer engineers to help develop a programme that can automatically analyse the image to see if this can provide some indication of the cause of the infection. Images of corneal infection may also be used for educational and teaching purposes, including in publications. All personal identifying information will be removed before sharing images.

**What tests will we do on the sample?**
The samples collected from the surface of your eye will be tested in several different ways to determine what is causing the infection. This work will be done in the hospital microbiology laboratory, where you are being treated. A portion of the infection sample will be transferred for additional special tests at KCMC Hospital Biotechnology (Tanzania), the London School of Hygiene and Tropical Medicine (UK) and Radboud University Nijmegen Medical Center (The Netherlands), as some of the tests will require additional special equipment that is not available at all sites.

- We will look for the type of infection using a microscope and by growing the organisms in the laboratory. We will test the organisms that grow to see which medicines work best to kill the infection, which is helpful in guiding the choice of treatment to be used.

- The swab samples from the ulcer will be used to test for infection using molecular diagnostic tests and to evaluate new tests that may be used to find the cause rapidly in the clinic.

- We will use study genetic material of the organism causing the infection to find out the exact type of infection and its ability to resist treatments. Samples from the ulcer may also be used to investigate your immune response to the infection, so that we can better understand what causes corneal scarring

- We will store a sample of the infection causing organism indefinitely for additional testing.

- The genetic material from the cells from your cheek will be sent to the UK or to KCMC Hospital in Tanzania. We would like to store it until a later time when a sufficiently large number of samples has been collected to conduct an analysis of this genetic material. The purpose would be to try to better understand how the human immune system fights the infection and why some people develop this eye problem and others do not. By doing these tests we hope that it will help us to develop approaches that will help to prevent or improve outcomes from this condition.

- As part of this consent we are asking that you give us permission to store this material to be able to test it at a later date as mentioned above. We do not know exactly for how long we shall store the genetic material before we have assembled a sufficiently large collection during the course of several planned studies to be able to proceed to the sample analysis, however, we anticipate a period of at least five years.

**What will happen to the results of the research study?**
The results of the study will be available after it finishes and will be included in peer reviewed medical and scientific journals and may be presented at medical meetings. Results will also be published on a publicly accessible trials database. The data will be anonymous and none of the patients involved in the trial will be identified in any report or publication. Should you wish to see the results, or the publication, please ask your study doctor.

**Who is funding the research?**
The research is being funded as part of a grant from the Wellcome Trust, UK.

**Who is organising the research?**
It is being organised through a research partnership between the London School of Hygiene and Tropical Medicine and Sagarmatha Choudhary Eye Hospital in Nepal, Mbarara University of Science and Technology in Uganda and Kilimanjaro Christian Medical Centre in Tanzania.

**What if relevant new information becomes available?**
It is not anticipated that new information will become available during the course of this short study. The information from the microbiology tests will be available to the doctors to help select the most appropriate treatment for you.

For peer review only

**What if something goes wrong?**
If you have a concern about any aspect of this study, you should ask to speak to the researchers who will do their best to answer your questions. If you remain unhappy and wish to complain formally, you can do this through the head of the hospital eye department or the named person on the following page. The London School of Hygiene and Tropical Medicine holds insurance policies which apply to this study. If you experience harm or injury as a result of taking part in this study, you may be eligible to claim compensation.

**Who has reviewed the study?**
Prospective research such as this is looked at by an independent group of people, called a Research Ethics Committee, to protect your interests. This study has been reviewed and approved by (1) the London School of Hygiene and Tropical Medicine Research Ethics Committee; (2) Nepal Health Research Council.

**What will happen if I don't want to carry on with the study?**
Your participation in this study is entirely voluntary. You may refuse to participate or may withdraw from this study at any time without penalty or loss of any rights or benefits to which you are otherwise entitled. The study doctor may also stop your participation in the study at any time for safety reasons. If you decide to withdraw from the study you should contact a member of the study team immediately. You do not have to give a reason when stopping, however for safety reasons, it is suggested that you tell the study doctor if you decide to stop because of an unwanted side effect. If you withdraw from the study, we will only use data collected before this decision, unless you request this to also be withdrawn. If you withdraw from the study, researchers, authorized persons from the Sponsor and the regulatory authorities will still require access to your medical notes to verify the data collected up to the date of your withdrawal.

**Contact Details**
Nepal Study Site: Dr Reena Yadav, Sagarmatha Choudhary Eye Hospital, Lahan. T: +977 9807799958

Study Coordinator: Dr Jeremy Hoffman: email: Jeremy.hoffman@lshtm.ac.uk
Chief Investigator: Prof. Matthew Burton: email: matthew.burton@lshtm.ac.uk

**You will be given a copy of the information sheet and a signed consent form to keep.**
**Thank you for considering taking the time to read this sheet.**

For peer review only

**Randomised controlled trial of topical chlorhexidine 0.2% versus topical natamycin 5% for fungal keratitis**

## Consent Form No 1: Baseline assessment and investigations

**Participant Name**___________________________ **Study ID Number:** ____________________

| | **Please initial box** |
|---|---|
| 1. I confirm that I have read and understand the participant information sheet dated ……….. (version ………) for the above study. I have had the opportunity to consider the information, ask questions and have had these answered fully. | |
| 2. I understand that my participation is voluntary and I am free to withdraw at any time, without giving any reason, without my medical care or legal rights being affected. | |
| 3. I understand that sections of my medical notes and data collected during the study may be looked at by responsible individuals from the London School of Hygiene & Tropical Medicine, from national regulatory authorities or from this hospital, where it is relevant to my taking part in this research. I give permission for these individuals to access my records. | |
| 4. I agree to take part in the above study. | |
| 5. I agree to the collection, laboratory tests and storage for future analysis of the samples from the surface my eye infection to understand the disease as described above. | |
| 6. I agree to the collection and storage for future analysis of the samples from the inside of my mouth to investigate how human genes affect this disease. | |
| 7. I agree to having a blood sample collected to measure sugar levels | |
| 8. I agree to being referred for a HIV pre-test counselling, testing and for the results to be shared with the research team. | |
| 9. I agree for the photographs of the front of my eye to be used in the publication or report released on the study, and for teaching purposes, including on the internet. | |
| 10. I understand that data about/from me/the participant may be shared via a public data repository or by sharing directly with other researchers, and that I will not be identifiable from this information. | |

Name of Participant *(printed)* Signature/Thumbprint Date

Name of Person taking consent Signature Date

The participant is unable to sign. As a witness, I confirm that all the information about the study was given and the participant consented to taking part.

Name of Impartial Witness *(if required)* Signature Date

*1 copy for participant; 1 copy for Principal Investigator; 1 copy to be kept with hospital notes*

For peer review only

# Study Title: Randomised controlled trial of topical chlorhexidine 0.2% versus topical natamycin 5% for fungal keratitis in Nepal

*Participant Information Sheet and Consent Form – No 2:*
*For enrolment into the clinical trial*

**Introduction**
Thank you for earlier participating in the first stage of the study. You are now being invited to take part in the clinical treatment trial part of this medical research study. Before you decide it is important for you to understand why the research is being done and what it will involve. Please take time to read or listen to the following information carefully and to talk to others about the study, if you wish. Ask us if there is anything that is not clear or if you would like more information. Do not sign the consent form unless you are satisfied with the answers to your questions and decide that you want to be part of this study. Take time to decide whether or not you wish to take part.

**Why have I been invited?**
You have been invited to take part because you have a corneal infection which we have found out from tests is caused by fungus.

**What is this study about?**
Infection of the clear part of the front of the eye (the cornea) is a corneal ulcer. It is an important cause of blindness in Nepal. A scratch in the cornea allows infection to enter and an ulcer to begin. These infections can be very serious with some people losing the sight in the affected eye.

Different types of infectious organisms can cause corneal ulcers. These include bacteria and fungi. In tropical regions about half of all corneal ulcers are caused by fungi. Bacteria and fungi need to be treated with different types of eye drop medicines.

Treatments for fungal eye infections are frequently not very effective, in addition access to these treatments in many countries is very limited and can be expensive. In some countries they are simply not available. Currently the most commonly used treatment for fungal corneal ulcers is an eye drop called Natamycin. There is a need for additional, alternative, affordable and more easily available eye drop treatments for fungal infections.

There is an antiseptic solution called Chlorhexidine. This is very effective at killing bacteria, fungi and other types of infectious organisms. It is used in medical care worldwide in several different ways. For example, it is used to clean skin before surgical operations, in antiseptic creams for skin cuts and as a mouth wash to prevent and treat mouth infections. It has been used in eye care for more than thirty years as an eye-drop preservative, for sterilizing contact lenses, for pre-operative topical antiseptic and for treating *Acanthamoeba* and fungal corneal infections.

About twenty years ago chlorhexidine eye drops were tested in two small clinical trials conducted in India and Bangladesh for the treatment of fungal corneal infections. The results of these studies suggested that chlorhexidine was as good as and possibly better than natamycin at controlling the infection. Neither eye drop had any serious side effect. However, the studies were not large enough to be certain.

Chlorhexidine is currently used in several countries for the treatment of fungal corneal infections when natamycin or alternative treatment is not working or is not available. We would like to conduct a large clinical to find out whether chlorhexidine 0.2% eye drops are as effective as or more effective than natamycin 5% eye drops for treating fungal corneal infections

**Do I have to take part?**
No. Your involvement is entirely voluntary. If you agree to take part, we will then ask you to sign a consent form. If you decide to join and change your mind, you are free to withdraw at any time without giving a reason. This will not affect the standard of care you receive. If you decide not to participate in the study, then you will be offered the standard treatment for fungal keratitis using natamycin.

**What will happen to me if I take part?**
If you agree to be part of this study, the following will happen:

***1) Baseline Assessment:***
As part of the initial assessment that has already taken place we have carefully examined yours eyes, taken photographs, performed a scan for infection and collected samples. The tests have found that you have a fungal infection in your cornea. The risks to an unborn or breast-fed baby from antifungal eye drops use are unknown. Therefore, pregnant and breastfeeding women are excluded from participating in this study. Pregnancy testing will be offered to potential female participants to confirm pregnancy status.

For peer review only

***2) Randomisation:***
We will randomly allocate you to one of the two treatment option: either chlorhexidine eye drops or natamycin eye drops. Sometimes we don't know which way of treating patients is best. To find out, we need to make comparisons between the different treatments. We put people into groups and give each group one of the two alternative treatments; the results are compared after some time to see if one is better. To try to make sure the groups are the same to start with, each patient is put into a group by chance (randomly). You have an equal chance of being put into the chlorhexidine or natamycin treatment group. Neither you nor the people examining your eyes will be told which treatment group you are in. It is important that neither you nor we know which of the two you are given. This information would be in our files, but we would not look at these files until after the research is finished. This is the best way we have for testing without being influenced by what we think might happen. We would then compare which of the two treatments has the best results.

***3) Treatment:***
Once you are allocated to one of the treatment groups, you will receive clear instruction on how to take the eye drops. For the first week we will ask you to take one drop every hour. For the second and the third weeks the frequency of the eye drops will be reduced to every other hour (2-hourly). After that the frequency and duration of treatment will depend on the severity of the infection and how it is responding. You will be given clear guidance on this by the eye doctor who is looking after you.

***4) Follow-up Assessment:***
Initially, most people with corneal infections stay in hospital for several days so that the clinical team can monitor the response of the infection to the treatment. For the purposes of the study, we would like to review the response to treatment and document the clinical findings at the following times after you start treatment: two days, 1 week, 2 weeks, 3 weeks, 2 months and 3 months.

On each occasion we will ask you a few questions about your eye and the treatment. We will measure your eye sight. We will examine the eye with a microscope and photograph it with a camera.

At 1 week, 2 weeks and 3 weeks we will repeat the *in vivo* confocal microscopy test that was done at your first assessment. This is done to see how the infection is responding to treatment. This involves putting anaesthetic drops on the eye so that you do not feel any discomfort. A soft plastic device then gently touches the surface of the eye so that we can take special photographs of the front of your eye ("scan")

At 1 week if you still have an open ulcer on the cornea we will repeat the sample collection to test for the ongoing presence of the infection. This involves first putting anaesthetic eye drops on the surface of the eye. Then gently scraping the surface of the corneal ulcer and testing for the presence of fungus and bacteria in the microbiology laboratory.

Sometimes fungal infections do not respond to the treatment. In such cases it may be necessary to alter the treatment or perform an operation. The eye doctors who will be looking after you will monitor your progress closely and advise you about further treatment might be needed.

At the three month review you will be asked to complete three short questionnaires to assess your quality of life and vision function. These are the same standardised questions used in the baseline assessment to try to understand the impact that different medical problems can have on people's lives.

You may be withdrawn from the study without your consent if the researchers believe it is in your best interest or if you fail to follow study procedures.

**What are the side effects or risks of taking part?**

1. **Random Allocation and Treatment Failure**: You will be randomly allocated to a treatment. The treatment you are allocated to may prove to be less effective or to have more side effects than the other study treatment or other available treatments.

   **It is important to recognise that corneal infection is a serious, sight threatening condition.** Many patients, whatever the treatment used, have reduced vision in the affected eye after it has resolved. In some people the affected eye will become blind. Sometimes the infection, despite lots of treatment, can progress to cause a hole to develop in the cornea (Perforation) and sometimes it is so severe it is necessary to perform an operation to remove the eye content.

2. **Local Irritation:** As with most eye drops, there is the risk of local irritation or stinging from either chlorhexidine or natamycin. This usually only lasts for a short time.

3. **Allergic Response**: Very rarely, either chlorhexidine or natamycin eye drops can provoke a local allergic reaction on the surface of the eye or the eyelids.

For peer review only

4. **Pregnancy and Breast Feeding:** The risks to an unborn or breast-fed baby from antifungal eye drops use are unknown. Therefore, pregnant and breastfeeding women are excluded from participating in this study.

5. **Natamycin 5% eye drops**: Natamycin is an approved antifungal medication that is currently being used for the treatment of fungal corneal ulcer. It is on the World Health Organisation Essential Medicines List for the treatment of fungal corneal infections. There are no known serious side effects with this medication. It may cause mild irritation and very rarely a local allergic response.

6. **Chlorhexidine 0.2% eye drops:** Chlorhexidine eye drops are used on the surface of the eye as an antiseptic before procedures and also in the treatment of fungal and other eye infections. It has not been associated we any serious side effects. It may cause mild irritation and very rarely a local allergic response. This concentration of chlorhexidine is approved to be used in much larger volumes as a mouth wash. It is considered to be safe and is not associated with any systemic side effects.

7. **Procedures:** including examinations, confocal microscopy, corneal sample collection and checking for the best glasses or contact lenses carry the same very small risk whether they are performed as part of this study or of usual care outside the study. To minimise discomfort, topical anaesthetic will be given before examinations and sample collection.

8. **Unknown Risks:** The treatments in this study may have rare side effects that are currently not known. If during the course of the study new information becomes available, the researchers will share this with.

**What are the possible benefits of taking part?**

- The study will involve tests for the type of infection. This helps the doctor looking after you to choose the best type of treatment for your eyes
- The costs for your clinical assessment, tests, treatment and transport will be paid for by the study.
- By participating in this study, you will be helping to answer the question about whether or not chlorhexidine is a suitable alternative treatment for fungal corneal infections.

**What will happen to the clinical records, photographs and test results?**

Your records will remain strictly confidential at all times. The information will be held in a secure office at your treating hospital. Only the people organizing or supervising the trial and regulatory authority auditors will have access to it. These include officials delegated by the Sponsor (London School of Hygiene and Tropical Medicine), the local National Ethics Committee, The local National Drug Regulatory Authority and trial Data Safety Monitoring Body (DSMB).

A study number rather than your name will be used on study records or the database wherever possible. Your name and other facts that might identify you will not appear when we present this study or publish its results.

Your name will not be passed to anyone else outside the research team, unless we have your direct instruction to do so, for example to make a medical referral.

Images of corneal infection may be used for educational and teaching purposes, including in publications. All personal identifying information will be removed before sharing images.

**What tests will we do on the sample?**

The samples collected from the surface of your eye will be tested in several different ways to determine what is causing the infection. This work will be done in the hospital microbiology laboratory, where you are being treated. A portion of the infection sample will be transferred for additional special tests at KCMC Hospital Biotechnology (Tanzania), the London School of Hygiene and Tropical Medicine (UK) and Radboud University Nijmegen Medical Center (The Netherlands).

We will look for the type of infection using a microscope and by growing the organisms in the laboratory. We will test the organisms that grow to see which medicines work best to kill the infection, which is helpful in guiding the choice of treatment to be used. The swab samples from the ulcer will be used to test for infection using molecular diagnostic tests and to evaluate new tests that may be used to find the cause rapidly in the clinic. We will use the genetic material of the organism causing the infection to sequence its genetic code, which helps us to find out the exact type of infection and its ability to resist treatments. We will store a sample of the infection causing organism indefinitely for additional testing.

**What will happen to the results of the research study?**

The results of the study will be available after it finishes and will be included in peer reviewed medical and scientific journals and may be presented at medical meetings. Results will also be published on a publicly accessible trials database. The data will be anonymous and none of the patients involved in the trial will be identified in any report or publication. Should you wish to see the results, or the publication, please ask your study doctor.

**Who is funding the research?**

For peer review only

The research is being funded as part of a grant from the Wellcome Trust, UK.

**Who is organising the research?**
It is being organised through a research partnership between the London School of Hygiene and Tropical Medicine and Sagarmatha Choudhary Eye Hospital in Nepal, Mbarara University of Science and Technology in Uganda and Kilimanjaro Christian Medical Centre in Tanzania.

**What if relevant new information becomes available?**
It is not anticipated that new information will become available during the course of this study. However, if it does, this will be shared with you by the researchers in case this affects whether you wish to continue in the study.

**What if something goes wrong?**
If you have a concern about any aspect of this study, you should ask to speak to the researchers who will do their best to answer your questions. If you remain unhappy and wish to complain formally, you can do this through the head of the hospital eye department or the named person on the following page. The London School of Hygiene and Tropical Medicine holds insurance policies which apply to this study. If you experience harm or injury as a result of taking part in this study, you may be eligible to claim compensation.

**Who has reviewed the study?**
Prospective research such as this is looked at by an independent group of people, called a Research Ethics Committee, to protect your interests. This study has been reviewed and approved by (1) the London School of Hygiene and Tropical Medicine Research Ethics Committee; (2) Nepal Health Research Council.

What will happen if I don't want to carry on with the study?
Your participation in this study is entirely voluntary. You may refuse to participate or may withdraw from this study at any time without penalty or loss of any rights or benefits to which you are otherwise entitled. The study doctor may also stop your participation in the study at any time for safety reasons. If you decide to withdraw from the study you should contact a member of the study team immediately. You do not have to give a reason when stopping, however for safety reasons, it is suggested that you tell the study doctor if you decide to stop because of an unwanted side effect. If you withdraw from the study, we will only use data collected before this decision, unless you request this to also be withdrawn. If you withdraw from the study, researchers, authorized persons from the Sponsor and the regulatory authorities will still require access to your medical notes to verify the data collected up to the date of your withdrawal.

**Contact Details**
Nepal Study Site: Dr Reena Yadav, Sagarmatha Choudhary Eye Hospital, Lahan. T: +977 9807799958

Study Coordinator: Dr Jeremy Hoffman: email: Jeremy.hoffman@lshtm.ac.uk
Chief Investigator: Prof. Matthew Burton: email: matthew.burton@lshtm.ac.uk

**You will be given a copy of the information sheet and a signed consent form to keep.**
**Thank you for considering taking the time to read this sheet.**

For peer review only

**Randomised controlled trial of topical chlorhexidine 0.2% versus topical natamycin 5% for fungal keratitis**

**Consent Form No 2: Enrolment into the clinical trial**

**Participant Name**____________________________ **Study ID Number:** ____________________

| | **Please initial box** |
|---|---|
| 1. I confirm that I have read and understand the participant information sheet dated .......... (version .........) for the above study. I have had the opportunity to consider the information, ask questions and have had these answered fully. | |
| 2. I understand that my participation is voluntary and I am free to withdraw at any time, without giving any reason, without my medical care or legal rights being affected. | |
| 3. I understand that sections of my medical notes and data collected during the study may be looked at by responsible individuals from the London School of Hygiene & Tropical Medicine, from national regulatory authorities or from this hospital, where it is relevant to my taking part in this research. I give permission for these individuals to access my records. | |
| 4. I agree to take part in this clinical treatment trial. | |
| 5. I agree to the collection, laboratory tests and storage for future analysis of the samples from the surface my eye infection to understand the disease as described above. | |
| 6. I agree for the photographs of the front of my eye to be used in the publication or report released on the study, and for teaching purposes, including on the internet. | |
| 7. I understand that data about/from me/the participant may be shared via a public data repository or by sharing directly with other researchers, and that I will not be identifiable from this information. | |

____________________ ____________________ ____________

Name of Participant *(printed)* Signature/Thumbprint Date

____________________ ____________________ ____________

Name of Person taking consent Signature Date

The participant is unable to sign. As a witness, I confirm that all the information about the study was given and the participant consented to taking part.

____________________ ____________________ ____________

Name of Impartial Witness *(if required)* Signature Date

*1 copy for participant; 1 copy for Principal Investigator; 1 copy to be kept with hospital notes*

For peer review only

## Appendix 2: Monitoring for Harm

### Definitions

**Adverse Event (AE):** any untoward medical occurrence in a patient or clinical trial subject administered a medicinal product and which does not necessarily have a causal relationship with this treatment. *An AE can therefore be any unfavourable and unintended sign (including an abnormal laboratory finding), symptom, or disease temporally associated with the use of an investigational medicinal product (IMP), whether or not considered related to the IMP.*

**Adverse Reaction (AR):** all untoward and unintended responses to an IMP related to any dose administered. *All AEs judged by either the reporting investigator or the sponsor as having reasonable causal relationship to a medicinal product qualify as adverse reactions. The expression reasonable causal relationship means to convey in general that there is evidence or argument to suggest a causal relationship.*

**Unexpected Adverse Reaction:** an AR, the nature or severity of which is not consistent with the applicable product information (e.g. investigator's brochure for an unapproved investigational product or summary of product characteristics (SmPC) for an authorised product*). When the outcome of the adverse reaction is not consistent with the applicable product information this adverse reaction should be considered as unexpected. Side effects documented in the SmPC which occur in a more severe form than anticipated are also considered to be unexpected.*

**Serious Adverse Event (SAE)** or **Serious Adverse Reaction:** any untoward medical occurrence or effect that at any dose

- **Results in death**
- **Is life-threatening** – *refers to an event in which the subject was at risk of death at the time of the event; it does not refer to an event which hypothetically might have caused death if it were more severe*
- **Requires hospitalisation, or prolongation of existing inpatients' hospitalisation**
- **Results in persistent or significant disability or incapacity**
- **Is a congenital anomaly or birth defect**

Medical judgement should be exercised in deciding whether an AE/AR is serious in other situations. Important AE/ARs that are not immediately life-threatening or do not result in death or hospitalisation but may jeopardise the subject or may require intervention to prevent one of the other outcomes listed in the definition above, should also be considered serious.

**Suspected Unexpected Serious Adverse Reaction (SUSAR):** any suspected adverse reaction related to an IMP that is both unexpected and serious.

### Causality

Most adverse events and adverse drug reactions that occur in this study, whether they are serious or not, will be expected treatment-related toxicities due to the drugs used in this study. The assignment of the causality should be made by the investigator responsible for the care of the participant using the definitions in the table below.

| Relationship | Description |
|---|---|
| **Unrelated** | There is no evidence of any causal relationship |
| **Unlikely** | There is little evidence to suggest there is a causal relationship (e.g. the event did not occur within a reasonable time after administration of the trial medication). There is another reasonable explanation for the event (e.g. the participant's clinical condition, other concomitant treatment). |
| **Possible** | There is some evidence to suggest a causal relationship (e.g. because the event occurs within a reasonable time after administration of the trial medication). However, the influence of other factors may have contributed to the event (e.g. the participant's clinical condition, other concomitant treatments). |
| **Probable** | There is evidence to suggest a causal relationship and the influence of other factors is unlikely. |

For peer review only

| | |
|---|---|
| **Definitely** | There is clear evidence to suggest a causal relationship and other possible contributing factors can be ruled out. |
| **Not assessable** | There is insufficient or incomplete evidence to make a clinical judgement of the causal relationship. |

If any doubt about the causality exists the local investigator should inform the study coordination centre who will notify the Chief Investigators. The pharmaceutical companies and/or other clinicians may be asked to advise in some cases.

In the case of discrepant views on causality between the investigator and others, all parties will discuss the case. In the event that no agreement is made, the MHRA will be informed of both points of view.

## Reporting Procedures

All adverse events should be reported. Depending on the nature of the event the reporting procedures below should be followed. Any questions concerning adverse event reporting should be directed to the study coordination centre in the first instance. A flowchart is given below (Figure 5) to aid in the reporting procedures and the reporting form is shown in (Data Collection Appendix).

***Figure 5: Adverse event reporting flow-chart***

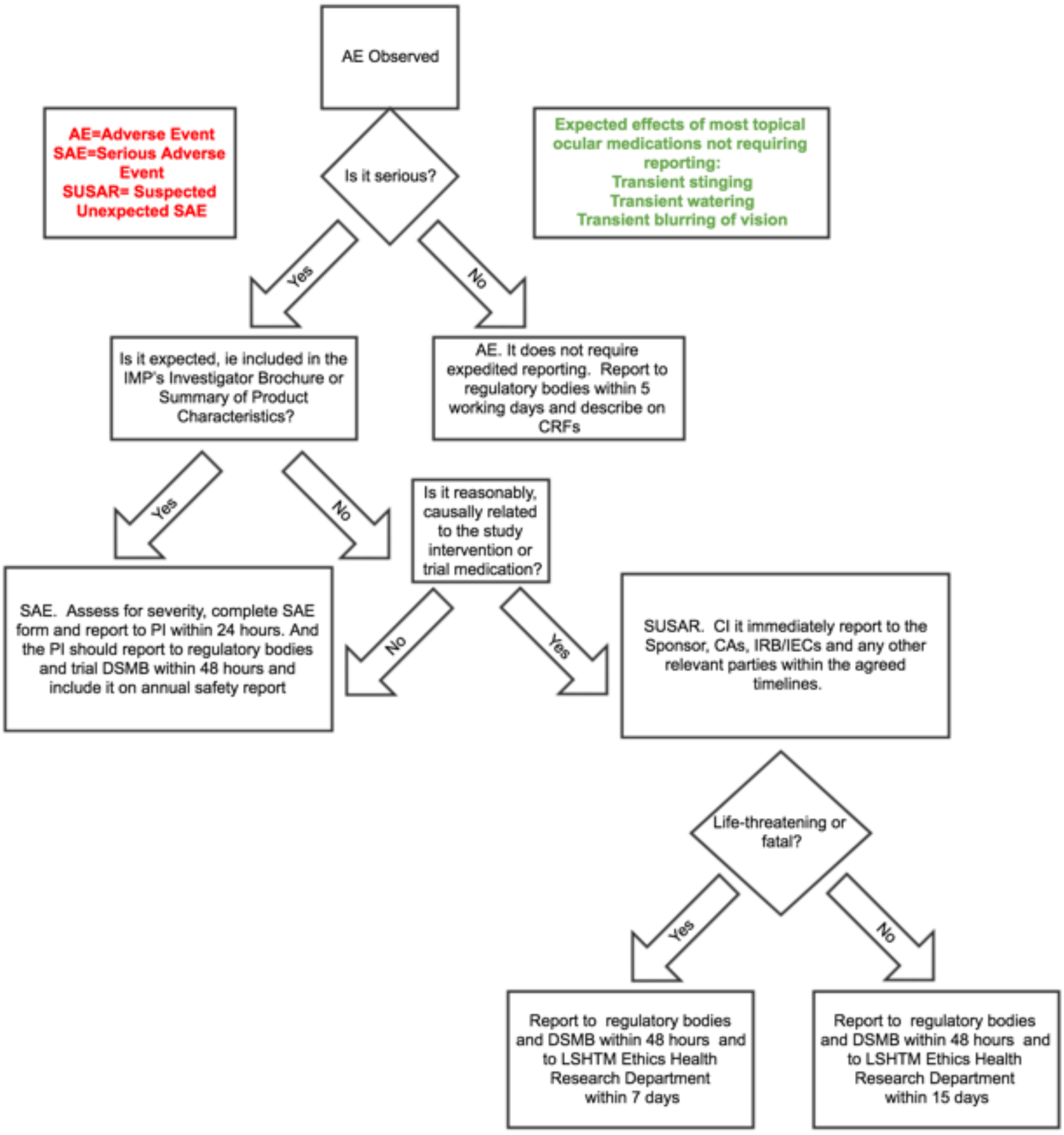

**Non-serious Adverse Reactions (ARs)/Adverse Events (AEs)**
All such events, whether expected or not, should be recorded in the toxicity section of the relevant case report form and sent to the study coordination centre, regulatory bodies and the trial DSMB within 5 working days from the day of occurrence.

**Serious Adverse Reactions (SARs)/Serious Adverse Events (SAEs)**
Fatal or life threatening Serious Adverse Events (SAEs), Serious Adverse Reactions (SARs) and Suspected Unexpected Serious Adverse Reactions (SUSARs) should be reported to the PI on the day that the local site is aware of the event. The SAE form asks for nature of event, date of onset, severity, corrective therapies given, outcome and causality (i.e. unrelated, unlikely, possible, probably, definitely). All SAEs or SARs whether expected, suspected or unexpected should be reported to regulatory bodies and the trial DSMB within 48 hours of occurrence. The responsible investigator should sign the causality of the event. Additional information should be sent within 5 days if the reaction has not resolved at the time of reporting. All investigators will be informed of all SARs occurring throughout the study.

**SAEs**
A SAE form should be completed and faxed to the study coordination centre for all SAEs within 24 hours. However, relapse and death due to a known pre-existing condition and hospitalisations for elective treatment of a pre-existing condition do not need reporting as SAEs.

**SUSARs**
In the case of serious, unexpected and related adverse events, the staff at the site should:

Complete the SAE case report form & send it immediately (within 24 hours, preferably by fax), signed and dated to the study coordination centre together with relevant treatment forms and anonymised copies of all relevant investigations.

**Or**

Contact the study coordination centre by phone and then send the completed SAE form to the study coordination centre within the following 24 hours as above.

The study coordination centre will notify all SUSARs occurring during the study within 48 hours to the Nepal Department of Drug Administration, and the trial DSMB. SUSARs will be reported to LSHTM Ethics Committee according to the following timelines; fatal and life-threatening within 7 days of notification and non-life threatening within 15 days. All investigators will be informed of all SUSARs occurring throughout the study.

Local investigators should report any SUSARs and /or SAEs as required by their Local Research Ethics Committee and/or Research & Development Office.

**Adverse Event Reporting for this Trial**
**Non-serious Ocular: narrative form not required**
- ☐ Local allergic reaction (conjunctiva, lid)
- ☐ Intraocular pressure ≥35 mmHg for 1 week despite therapy
- ☐ Progressive corneal thinning, ≤50% of enrolment thickness
- ☐ Other (specify)

**Serious Ocular: (1) report within 24hrs (2) Serious Adverse Event (SAE) Form required**
- ☐ Endophthalmitis
- ☐ Other serious ocular event thought to be related to the study drug, requiring non-elective hospitalization, loss of function, or systemic treatment (specify)

**Serious Non-ocular: (1) report within 24hrs (2) Serious Adverse Event (SAE) Form required**
- ☐ Death
- ☐ Non-elective surgery, hospitalization, or loss of function
- ☐ Myocardial infarction or stroke
- ☐ Systemic allergic reaction

For peer review only

## Appendix 3: Biological Specimens

The project will equip and staff an ocular microbiology laboratory at the hospital. The microbiology analysis will involve the following investigations:

1) **Microscopy** will be performed of the slides using Gram stain, KOH and calcofluor white.

2) **Culture** will be performed using a variety of media inoculated with corneal scrape samples in the clinic: blood agar, chocolate agar, brain-heart infusion broth (incubated for 2-7 days at 37°C) and Sabouraud agar (incubated for 7 days at 30°C). Culture isolates will be identified using standard microbiological techniques. Fungal and bacterial isolates will be stored frozen for long-term reference and subsequent additional analysis.

3) **Antibiotic susceptibility testing** of bacteria culture isolates will be performed using standard disc diffusion techniques in each of the laboratories.

4) **Antifungal susceptibility testing** of fungal isolates will be performed at KCMC, Tanzania . Isolates from Uganda and Nepal will be periodically transferred to KCMC, under a Material Transfer Agreement. They will be re-cultured and tested using a dilution series method for four drugs: Natamycin, Chlorhexidine, Voriconazole and Amphotericin-B. The technique has been developed at Radboud University, The Netherlands.

**PCR for microorganisms**

DNA from the dry swab will be extracted using the QIAamp mini kit (Qiagen) with bead-beating. Samples will be tested for the presence of bacterial (16rRNA), fungal (ITS1) and specific viral (HSV1&2) DNA by quantitative PCR. This PCR work will be performed at KCMC, Tanzania. Samples from Uganda and Nepal will be periodically transferred to KCMC for testing, under a Material Transfer Agreement. The performance of PCR as a diagnostic tool will be evaluated against a composite reference standard for positive cases. Cases will be considered positive if the pathogen is detected by either smear microscopy, IVCM or culture.

**Point of care tests for fungal infection**

Point-of-care tests for fungal keratitis would be very helpful. Molecular tests are generally more sensitive than antigen-based assays. Isothermal nucleic amplification techniques offer the sensitivity of PCR amplification without the need for specialized equipment. A result may be obtained within 30 minutes. Promising molecular approaches include isothermal amplification (Loop-mediated isothermal amplification (LAMP), Recombinase polymerase amplification (RPA)), associated with lateral flow assays for amplicon detection. Several assays have been developed that detect fungi, however, these have not been evaluated in fungal keratitis. The second ulcer swab will be used to test and develop isothermal molecular assays in comparison with the standard microbiological approaches described above. Results will be evaluated against a composite reference standard for fungal diagnosis as described above.

For peer review only

**Fungal DNA sequencing**

It is not always possible to determine the species of a fungus from classical microbiological techniques. It is now common to use fungal DNA sequencing for species-level diagnosis for some genera, including *Fusarium spp.*, which is the commonest fungal genus reported for MK in the study settings. We will use sequencing of the fungal ITS and TEF1$\alpha$ regions for this.[1] DNA will be extracted from the cultured fungal isolate when available. If there no fungus is cultured but the PCR is positive, then this PCR product will be used for sequencing. The DNA will be extracted from the isolates in KCMC, Tanzania. The extracted DNA will be sent under a Material Transfer Agreement to the Center of Expertise in Mycology Radboudumc/CWZ, Radboud University Nijmegen Medical Center and the CBS Fungal Biodiversity Centre, Utrecht, the Netherlands for the specialised sequencing and identification work.

**Corneal gene expression**

Three prime RNAseq (3'RNAseq) generates one read per transcript and enables strand-specific, highly multiplexed sequencing of poor concentration or quality samples (as low as 100pg total RNA input) for gene expression analysis. We have previously used this method for gene expression profiling of human conjunctival swabs from a trachoma-endemic area. RNA will be extracted from corneal swabs or scrapes using Norgen RNA purification kits. 3'RNAseq (Lexogen) will be performed on normalised RNA extracts from 500 Tanzanian and Ugandan participants with confirmed fungal keratitis. Pooled libraries will be sequenced on a HiSeq (Illumina). Transcriptomic profiles will be analysed for differences between controls and mild, moderate and severe FK cases using DEseq2, and between participants with good versus poor visual outcomes. A small number of healthy corneal tissue controls will be derived from the residual ring of tissue remaining after corneal graft surgery (ethics application for control tissue to be submitted separately).

**Human Genetics**

DNA will be extracted from buccal brush samples using QIAmp DNA mini kits (Qiagen). Samples from 500 confirmed fungal keratitis participants from Nepal and 500 matched controls will be run on immune-focussed Infinium ImmunoArray-24 BeadChips (Illumina) covering >240,000 genetic polymorphisms (including copy number variants, germline variants, insertions-deletions, single nucleotide polymorphisms (SNPs) and structural variants) at UCL genomics centre. In addition to SNP testing, two-field resolution Human Leukocyte Antigen (HLA) allele typing will be performed in all 1000 samples. Allele level HLA typing cannot currently be determined from SNP data but the HLA genes are primary immune response genes that have a role in many diseases and warrant independent study. Data will be analysed to determine sources of immunogenetic variation associated with fungal keratitis. These data will be linked to gene expression data from the same individuals to determine functional effects of genetic variation associated with fungal keratitis.

For peer review only

**HIV testing**

As outlined above, patients with microbial keratitis will be offered counselling and testing for HIV infection. This will be done through the established services in each of the hospitals. Appropriate onward referrals will be made if a test is found to be positive.

**Blood glucose testing**

Patients will have capillary blood glucose (CBG) testing done at presentation. Patients with an elevated CBG (>6.1mmol) will be referred to the hospital physicians for assessment and formal diagnosis of impaired glucose tolerance or diabetes mellitus. This level is considered a suitable cut-off to detect individuals with diabetes and has been validated in a south-Asian population.[2]

**References:**

1 Schoch CL, Seifert KA, Huhndorf S, *et al.* Nuclear ribosomal internal transcribed spacer (ITS) region as a universal DNA barcode marker for Fungi. *Proc Natl Acad Sci USA* 2012;**109**:6241–6. doi:10.1073/pnas.1117018109

2 Somannavar S, Ganesan A, Deepa M, *et al.* Random capillary blood glucose cut points for diabetes and pre-diabetes derived from community-based opportunistic screening in India. *Diabetes Care* 2009;**32**:641–3. doi:10.2337/dc08-0403

For peer review only

## Appendix 4: SPIRIT Checklist for Topical chlorhexidine 0.2% versus topical natamycin 5% for fungal keratitis in Nepal randomised clinical trial

| Section / Item | Item No. | Description | Location Reference |
|---|---|---|---|
| **Administrative information** | | | |
| Title | 1 | Descriptive title identifying the study design, population, interventions, and, if applicable, trial acronym | Page 1 of study protocol |
| Trial registration | 2a | Trial identifier and registry name. If not yet registered, name of intended registry | ISRCTN14332621; pre-results |
| | 2b | All items from the World Health Organization Trial Registration Data Set | ISRCTN14332621 Study Registry |
| Protocol Version | 3 | Date and version identifier | Every page of study protocol |
| Funding | 4 | Sources and types of financial, material, and other support 5a Names, affiliations, and roles of protocol contributors | Section 5.3 of study protocol, page 25<br>Page 17 of Manuscript |
| Roles and Responsibilities | 5a | Names, affiliations, and roles of protocol contributors | Page 1 of study protocol<br>Page 1 of Manuscript |
| | 5b | Name and contact information for the trial sponsor | Page 1 of study protocol<br>Page 1 of Manuscript |
| | 5c | Role of study sponsor and funders, if any, in study design; collection, management, analysis, and interpretation of data; writing of the report; and the decision to submit the report for publication, including whether they will have ultimate authority over any of these activities | Page 1 of study protocol and Section 5.2 of study protocol, page 25.<br><br>Page 17 of Manuscript |
| | 5d | Composition, roles, and responsibilities of the coordinating centre, steering committee, endpoint adjudication committee, data management team, and other individuals or groups overseeing the trial, if applicable (see Item 21a for data monitoring committee) | Section 5.1, page 24 of study protocol<br><br>Section 6, page 25 of study protocol<br><br>Page 11 of Manuscript |
| **Introduction** | | | |
| Background and rationale | 6a | Description of research question and justification for undertaking the trial, including summary of relevant studies (published and unpublished) examining benefits and harms for each intervention | Section 1, pages 6-9 of study protocol<br><br>Pages 4-5 of Manuscript |
| | 6b | Explanation for choice of comparators | Sections 1.3 – 1.4, pages 7-9 of study protocol<br>Pages 4-5 of Manuscript |

For peer review only

| | | | |
|---|---|---|---|
| Objectives | 7 | Specific objectives or hypotheses | Section 3, page 11 of study protocol<br><br>Page 5 of Manuscript |
| Trial design | 8 | Description of trial design including type of trial (eg, parallel group, crossover, factorial, single group), allocation ratio, and framework (eg, superiority, equivalence, noninferiority, exploratory) | Section 4, pages 12-24 of study protocol<br><br>Page 6 of Manuscript |
| **Methods: Participants, interventions, and outcomes** | | | |
| Study setting | 9 | Description of study settings (eg, community clinic, academic hospital) and list of countries where data will be collected. Reference to where list of study sites can be obtained | Section 4.3, page 13 of study protocol<br><br>Page 6 of Manuscript |
| Eligibility criteria | 10 | Inclusion and exclusion criteria for participants. If applicable, eligibility criteria for study centres and individuals who will perform the interventions (eg, surgeons, psychotherapists) | Section 4.4, page 13 of study protocol<br><br>Page 7 of Manuscript and Table 1 of Manuscript |
| Interventions | 11a | Interventions for each group with sufficient detail to allow replication, including how and when they will be administered | Section 4.7, page 17 of study protocol<br><br>Pages 8-9 of Manuscript |
| | 11b | Criteria for discontinuing or modifying allocated interventions for a given trial participant (eg, drug dose change in response to harms, participant request, or improving/worsening disease) | Page 11 of Manuscript |
| | 11c | Strategies to improve adherence to intervention protocols, and any procedures for monitoring adherence (eg, drug tablet return, laboratory tests) | Section 4.9, page 19 of study protocol<br><br>Pages 10-11 of Manuscript |
| | 11d | Relevant concomitant care and interventions that are permitted or prohibited during the trial | Section 4.7, pages 17-18 of study protocol<br>Pages 9-10 of Manuscript |
| Outcomes | 12 | Primary, secondary, and other outcomes, including the specific measurement variable (eg, systolic blood pressure), analysis metric (eg, change from baseline, final value, time to event), method of aggregation (eg, median, proportion), and time point for each outcome. Explanation of the clinical relevance of chosen efficacy and harm outcomes is strongly recommended | Section 4.11, page 22 of study protocol<br><br>Pages 10-11 of Manuscript |

For peer review only

| | | | |
|---|---|---|---|
| Participant timeline | 13 | Time schedule of enrolment, interventions (including any run-ins and washouts), assessments, and visits for participants. A schematic diagram is highly recommended (see Figure) | Table 3, page 19 of study protocol<br><br>Table 4 of Manuscript |
| Sample size | 14 | Estimated number of participants needed to achieve study objectives and how it was determined, including clinical and statistical assumptions supporting any sample size calculations | Section 4.12, page 22 of study protocol<br><br>Pages 12 -13 of Manuscript |
| Recruitment | 15 | Strategies for achieving adequate participant enrolment to reach target sample size | Section 4.5, page 14 of study protocol<br><br>Page 7 of Manuscript |
| **Methods: Assignment of interventions (for controlled trials)** | | | |
| Allocation: sequence generation | 16a | Method of generating the allocation sequence (eg, computer- generated random numbers), and list of any factors for stratification. To reduce predictability of a random sequence, details of any planned restriction (eg, blocking) should be provided in a separate document that is unavailable to those who enrol participants or assign interventions | Section 4.8, pages 18-19 of study protocol<br><br>Pages 7-8 of Manuscript |
| Allocation: concealment mechanism | 16b | Mechanism of implementing the allocation sequence (eg, central telephone; sequentially numbered, opaque, sealed envelopes), describing any steps to conceal the sequence until interventions are assigned | |
| Allocation: Implementation | 16c | Who will generate the allocation sequence, who will enrol participants, and who will assign participants to interventions | |
| Blinding (masking) | 17a | Who will be blinded after assignment to interventions (eg, trial participants, care providers, outcome assessors, data analysts), and how | Section 4.8, pages 18-19 of study protocol<br><br>Page 8 of Manuscript |
| | 17b | If blinded, circumstances under which unblinding is permissible, and procedure for revealing a participant's allocated intervention during the trial | Page 8 of Manuscript |
| **Methods: Data collection, management, and analysis** | | | |
| | 18a | Plans for assessment and collection of outcome, baseline, and other trial data, including any | Section 4.14, page 23 of study protocol |

For peer review only

| | | | |
|---|---|---|---|
| Data collection methods | | related processes to promote data quality (eg, duplicate measurements, training of assessors) and a description of study instruments (eg, questionnaires, laboratory tests) along with their reliability and validity, if known. Reference to where data collection forms can be found, if not in the protocol | Page 12 of Manuscript |
| | 18b | Plans to promote participant retention and complete follow-up, including list of any outcome data to be collected for participants who discontinue or deviate from intervention protocols | Page 12 of Manuscript |
| Data management | 19 | Plans for data entry, coding, security, and storage, including any related processes to promote data quality (eg, double data entry; range checks for data values). Reference to where details of data management procedures can be found, if not in the protocol | Section 4.14, page 23 of study protocol<br><br>Page 12 of Manuscript |
| Statistical methods | 20a | Statistical methods for analysing primary and secondary outcomes. Reference to where other details of the statistical analysis plan can be found, if not in the protocol | Section 4.13, pages 22-23 of study protocol<br><br>Pages 12-15 of Manuscript |
| | 20b | Methods for any additional analyses (eg, subgroup and adjusted analyses) | |
| | 20c | Definition of analysis population relating to protocol non-adherence (eg, as randomised analysis), and any statistical methods to handle missing data (eg, multiple imputation) | |
| **Methods: Monitoring** | | | |
| Data Monitoring | 21a | Composition of data monitoring committee (DMC); summary of its role and reporting structure; statement of whether it is independent from the sponsor and competing interests; and reference to where further details about its charter can be found, if not in the protocol. Alternatively, an explanation of why a DMC is not needed | Section 6, page 25 of study protocol<br><br>Page 12 of Manuscript |
| | 21b | Description of any interim analyses and stopping guidelines, including who will have access to | Section 4.13, page 23 of study protocol |

For peer review only

| | | | |
|---|---|---|---|
| | | these interim results and make the final decision to terminate the trial | Page 15 of Manuscript |
| Harms | 22 | Plans for collecting, assessing, reporting, and managing solicited and spontaneously reported adverse events and other unintended effects of trial interventions or trial conduct | Section 7, pages 26-28 of study protocol<br><br>Page 12 of Manuscript<br><br>Annex 2 |
| Auditing | 23 | Frequency and procedures for auditing trial conduct, if any, and whether the process will be independent from investigators and the sponsor | Section 6, page 25 of study protocol<br><br>Page 12 of Manuscript |
| **Ethics and Dissemination** | | | |
| Research ethics approval | 24 | Plans for seeking research ethics committee/institutional review board (REC/IRB) approval | Section 4.15, page 24 of study protocol<br>Page 15 of Manuscript |
| Protocol amendments | 25 | Plans for communicating important protocol modifications (eg, changes to eligibility criteria, outcomes, analyses) to relevant parties (eg, investigators, REC/IRBs, trial participants, trial registries, journals, regulators) | Section 4.15, page 24 of study protocol<br>Page 15 of Manuscript |
| Consent or assent | 26a | Who will obtain informed consent or assent from potential trial participants or authorised surrogates, and how (see Item 32) | Section 4.5, page 14 of study protocol<br><br>Page 7 of Manuscript<br><br>Appendix 1: Consent Forms |
| | 26b | Additional consent provisions for collection and use of participant data and biological specimens in ancillary studies, if applicable | |
| Confidentiality | 27 | How personal information about potential and enrolled participants will be collected, shared, and maintained in order to protect confidentiality before, during, and after the trial | Page 12 of Manuscript |
| Declaration of interests | 28 | Financial and other competing interests for principal investigators for the overall trial and each study site | Page 18 of submitted manuscript |
| Access to data | 29 | Statement of who will have access to the final trial dataset, and disclosure of contractual agreements that limit such access for investigators | Page 12 of Manuscript |
| Ancillary and post-trial care | 30 | Provisions, if any, for ancillary and post-trial care, and for compensation to those who suffer harm from trial participation | Page 9 of Manuscript |
| Dissemination policy | 31a | Plans for investigators and sponsor to communicate trial results to participants, healthcare | Section 10, page 33 of study protocol |

For peer review only

| | | | |
|---|---|---|---|
| | | professionals, the public, and other relevant groups (eg, via publication, reporting in results databases, or other data sharing arrangements), including any publication restrictions | Pages 15-16 of Manuscript |
| | 31b | Authorship eligibility guidelines and any intended use of professional writers | Page 24 of Manuscript |
| | 31c | Plans, if any, for granting public access to the full protocol, participant- level dataset, and statistical code | Page 24 of Manuscript |
| **Appendices** | | | |
| Informed consent materials | 32 | Model consent form and other related documentation given to participants and authorised surrogates | Appendix 1 of Manuscript |
| Biological specimens | 33 | Plans for collection, laboratory evaluation, and storage of biological specimens for genetic or molecular analysis in the current trial and for future use in ancillary studies, if applicable | Appendix 3 of Manuscript |

For peer review only
